# Small Molecule CD38 Inhibitors: Synthesis of 8-Amino-*N*1-Inosine 5′-Monophosphate, Analogues and Early Structure-Activity Relationship

**DOI:** 10.3390/molecules26237165

**Published:** 2021-11-26

**Authors:** Joanna M. Watt, Richard Graeff, Barry V. L. Potter

**Affiliations:** 1 Medicinal Chemistry & Drug Discovery, Department of Pharmacology, University of Oxford, Mansfield Road, Oxford, OX1 3QT, UK; 2 Wolfson Laboratory of Medicinal Chemistry, Department of Pharmacy and Pharmacology, University of Bath, Claverton Down, Bath, BA2 7AY, UK; 3 Department of Physiology, University of Hong Kong, Hong Kong, China

## Abstract

Although a monoclonal antibody targeting the multifunctional ectoenzyme CD38 is an FDA approved drug, few small molecule inhibitors exist for this enzyme that catalyzes *inter alia* the formation and metabolism of the *N*1-ribosylated, Ca^2+^-mobilizing, second messenger cyclic adenosine 5′-diphosphoribose (cADPR). *N*1-Inosine 5′-monophosphate (*N*1-IMP) is a fragment directly related to cADPR. 8-Substituted-*N*1-IMP derivatives, prepared by degradation of cyclic parent compounds, inhibit CD38-mediated cADPR hydrolysis more efficiently than related cyclic analogues, making them attractive for inhibitor development. A total synthesis of the *N*1-IMP scaffold from adenine was designed and a small initial compound series facilitated early delineation of structure-activity parameters, with analogues evaluated for inhibition of CD38-mediated hydrolysis of cADPR. The 5′-phosphate group proved essential for useful activity, but substitution of this group with a sulfonamide bioisostere was not fruitful. 8-NH_2_-*N*1-IMP proved the most potent inhibitor (IC_50_ = 7.6 μM) and importantly HPLC studies showed this ligand to be cleaved at high CD38 concentrations, confirming its access to the CD38 catalytic machinery. Such truncated ligands further demonstrate the potential of our fragment approach.

## Introduction

**Figure 1:**
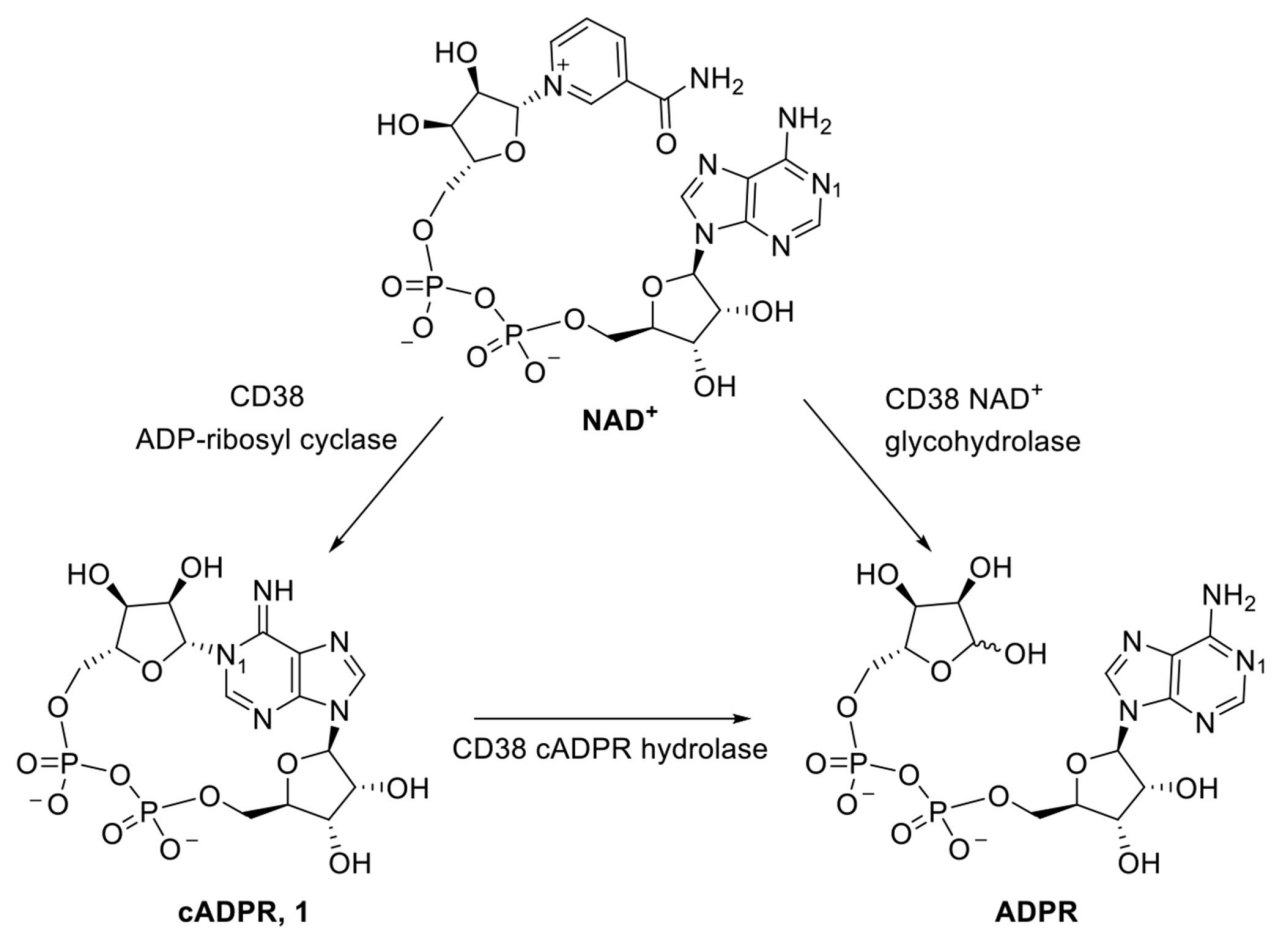
Conversion of NAD^+^ into cADPR and ADPR.

The second messenger cyclic ADP-ribose (cADPR, **1**, Figure 1) mobilizes intracellular Ca^2+^ in numerous cell types^1^. cADPR is synthesized enzymatically from nicotinamide adenine dinucleotide (NAD^+^) through ADP-ribosyl cyclases and is hydrolyzed at the *N*1-glycosidic linkage to give ADP-ribose (ADPR) both chemically and under physiological conditions ^2–4^. cADPR chemistry and the cADPR/Ca^2+^ signalling system have been reviewed ^5–11^.

The multifunctional ectoenzyme human CD38 is primarily an NAD^+^ glycohydrolase (NADase) and transforms NAD^+^ into ADPR (Figure 1), but it can also produce a small amount of cADPR through its cyclase activity and convert cADPR into ADPR through its hydrolase activity ^12–13^. It catalyzes the biosynthesis of two calcium-mobilizing second messengers cADPR and nicotinamide adenine dinucleotide phosphate (NAADP).

CD38 Has relevance to a range of diseases, *e.g*. it is a marker of AIDS progression and a negative prognostic marker of chronic lymphocytic leukaemia. A recent review categorized the enzyme as a druggable target, at least for human cancers^14^. It has also been shown to be influential for social behaviour in mice^15^ and plays a key role in age-related NAD^+^ decline. NAD^+^ metabolism is implicated in the ageing process and in the pathogenesis of several diseases. CD38 inhibition can decrease NADase activity and boost cellular NAD^+^ levels and such a therapy could be used to promote increases in longevity and healthspan in models of ageing and age-related disease.^14^ Therefore, there is significant wide interest to identify CD38 inhibitors and provide structural clues for design of potential drug candidates.

The greatest therapeutic success so far has been in multiple myeloma using CD38 inhibitors as an antibody-based therapy to target white blood cells in the bone marrow that cause the disease and where CD38 is found on the cell surface. Darzalex (daratumumab), an FDA-approved CD38 inhibitor for mono- and combination therapy of multiple myeloma, binds to CD38, blocks the growth of the cells and induces their death. Several other antibody therapies are currently being evaluated in clinical trials, for example Isatuximab, GBR 1342, TAK-079 and TAK-169,^16^ but relatively few small molecule CD38 inhibitors have been reported to date and there is a need to identify lead structures.

Inhibitors of the CD38 NAD^+^ glycohydrolase activity have mainly been investigated, the best being covalent mechanism-based agents that modify the active site. For example, nicotinamide ribose derivatives derived from NAD^+^ exhibit K_*i*_ values in the nanomolar range.^17,18^ Metabolically stable nicotinamide-based analogues can block endogenous CD38 activity.^19^ A non-hydrolyzable NAD^+^ analogue is a weak micromolar competitive inhibitor.^19^ Membrane permeable analogues are low mM inhibitors and could relax agonist-induced muscle contraction.^20^ NAD^+^ analogues with ribose, nucleobase, or pyrophosphate modifications have been explored.^21^ Others have explored non-nucleotide compounds and non-covalent compounds *via* screening methodologies.^22^ Screening yielded a compound that after optimization afforded a non-covalent CD38 NADase inhibitor with an IC_50_ of 4.7 μM. Low micromolar concentrations of flavonoids inhibit CD38. ^23^ A recent study reported the first small molecule allosteric modulator LX102.^24^ The structure of the CD38 catalytic domain and mechanism of cADPR breakdown have recently been elucidated crystallographically using covalent inhibitors. ^25,26^ Glu-226 is the catalytic residue and mutation eliminates activity. ^27^ Glu-146 is critical to regulate the multi-functionality of CD38-mediated NAD^+^ hydrolysis, the ADP-ribosyl cyclase and cADPR hydrolysis activities. ^27–29^

**Figure 2:**
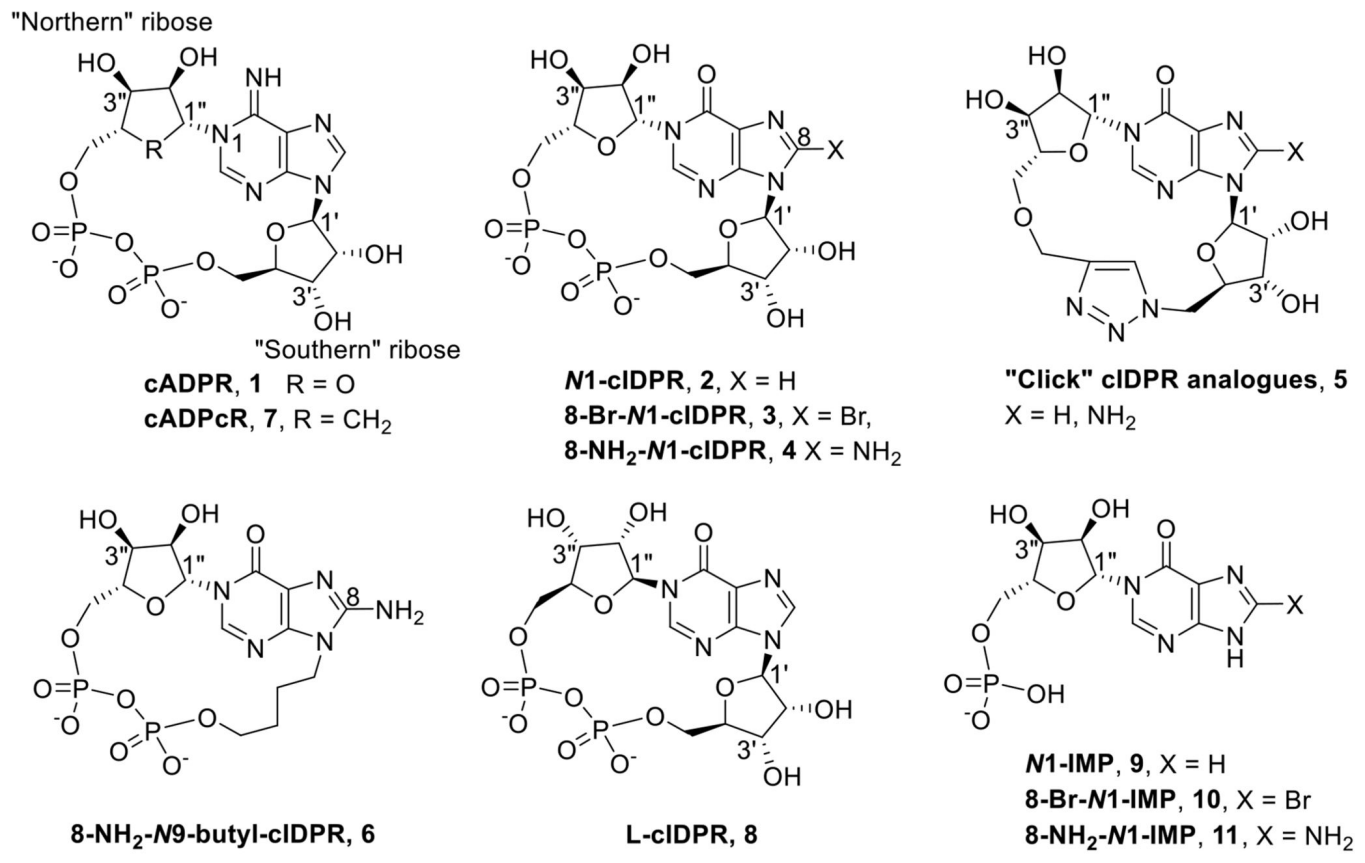
Structure and nomenclature of cADPR and previous analogues. NB the “northern” and “southern” riboses of the cyclic analogues are distinguished by adopting prime (′) and double prime (″) notation respectively for their sugar carbons.

We previously designed the hydrolysis-resistant cADPR analogue, cyclic inosine 5′-diphosphoribose (*N*1-cIDPR, **2**, Figure 2) ^30,31^ in which an oxo group at position 6 replaces the amino group. cADPR hydrolysis by CD38 is inhibited with an IC_50_ of 276 μM and in T-cells *N*1-cIDPR induces Ca^2+^ release almost indistinguishably to that induced by cADPR. ^30,32^ We also described the first total synthesis of the membrane permeant, hydrolytically stable, analogue 8 bromo-cIDPR (**3**) *via* regio- and stereoselective *N*1-ribosylation of protected 8-bromoinosine. ^33^ A crystal structure of the ligand with wild-type CD38 ^34^ showed *N*1-cIDPR to bind in the active site, close to catalytic Glu-226 with the two hydroxyl groups of the “northern” ribose forming hydrogen bonds. This work facilitates, at least in principle, structure-based design of novel CD38 inhibitors using the *N*1-cIDPR template. In another approach to more drug-like inhibitors we deleted the pyrophosphate group of the macrocycle using a “click” approach without serious loss of activity (**5**, Figure 2). ^35^

Analogs based on the *N*1-cIDPR template replaced the “southern” *N*9-ribose, with a butyl chain illustrating the nonessential nature of the “southern” ribose for binding ^36^ and 8-amino-*N*9-butyl-cIDPR (**6**) compared to the best non-covalent CD38 inhibitors (IC_50_=3.3 μM). Crystallographic analysis of the complex with CD38 unexpectedly revealed an *N*1-hydrolyzed ligand in the active site and ligand cleavage at high protein concentrations was confirmed. We described X-ray crystal structures of CD38 in complex with two non-hydrolyzable inhibitors, an 8-substituted *N*1-cIDPR analogue^37^ and cADP carbocyclic ribose (cADPcR, **7**, Figure 2) ^38^ and the elucidation of a preliminary SAR for inhibitors. ^39^

More recently, we exploited the cIDPR template to generate CD38 inhibitors *via* total synthesis. In the first example of a sugar hybrid cIDPR analogue, L-cIDPR (**8**), the natural “northern” *N*1-linked D-ribose of cADPR was replaced by an L-ribose ^40^ and other work has demonstrated the existence of conformers in these macrocycles. ^41^

From a comparison of the *N*1-cIDPR and cADPcR complexes with wild-type CD38 it was clear that the “northern” ribose part of the cyclic dinucleotide (ribose and/or carbocycle) is more important in binding than the “southern” part. ^39^ The “northern” ribose monophosphate motif of *N*1-cIDPR and the carbocyclic ribose monophosphate of cADPcR overlap with the rest of the ligand accommodated more flexibly. This implied that perhaps non-cyclic simple fragments of the macrocycle could maintain key interactions with wild-type CD38 and might inhibit the enzyme.

**Scheme 1:**
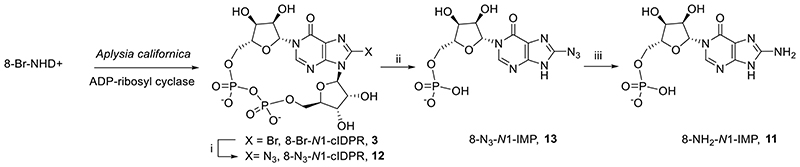
Previous preparation of *N*1-IMP compounds. Reagents and conditions: i) NaN_3_, ii) 0.2 M HCl; iii) dithiothreitol.

A small series of *N*1-hypoxanthine ribose 5′-monophosphate fragments (*N*1-IMPs, **9**-**11**), derived from careful degradation of the parent cyclic compound ^39,42^ were indeed inhibitors of CD38-catalyzed cADPR hydrolysis (Scheme 1). Moreover, 8-amino *N*1-IMP (**11**) showed promise, being considerably better than its cyclic counterpart (7.6 μM *cf*. 8-NH_2_-*N*1-cIDPR (**4**) at 56 μM) and this was explored and rationalised in a preliminary fashion through docking experiments.^39^ The reduced complexity and lower molecular weight of such fragments make them attractive as a starting point for further inhibitor design. 8-NH_2_-*N*1-IMP is amongst the best small non-covalent molecule inhibitors of CD38 activity reported so far; thus, its further development to design agents for pharmacological intervention is desirable and a straightforward synthesis is required.

We now report the synthesis of 8-NH_2_-*N*1-IMP (**11**) and a small focused SAR study to examine obvious points of substitution and clarify the importance of the “northern” ribose phosphate group motif for cIDPR-based inhibitors. Analogues were evaluated for their inhibition of CD38-mediated hydrolysis of cADPR.

## Results and Discussion

Initially, fragments **14**-**17** were designed based on the cIDPR structure with only the pyrophosphate deleted.

**Scheme 2:**
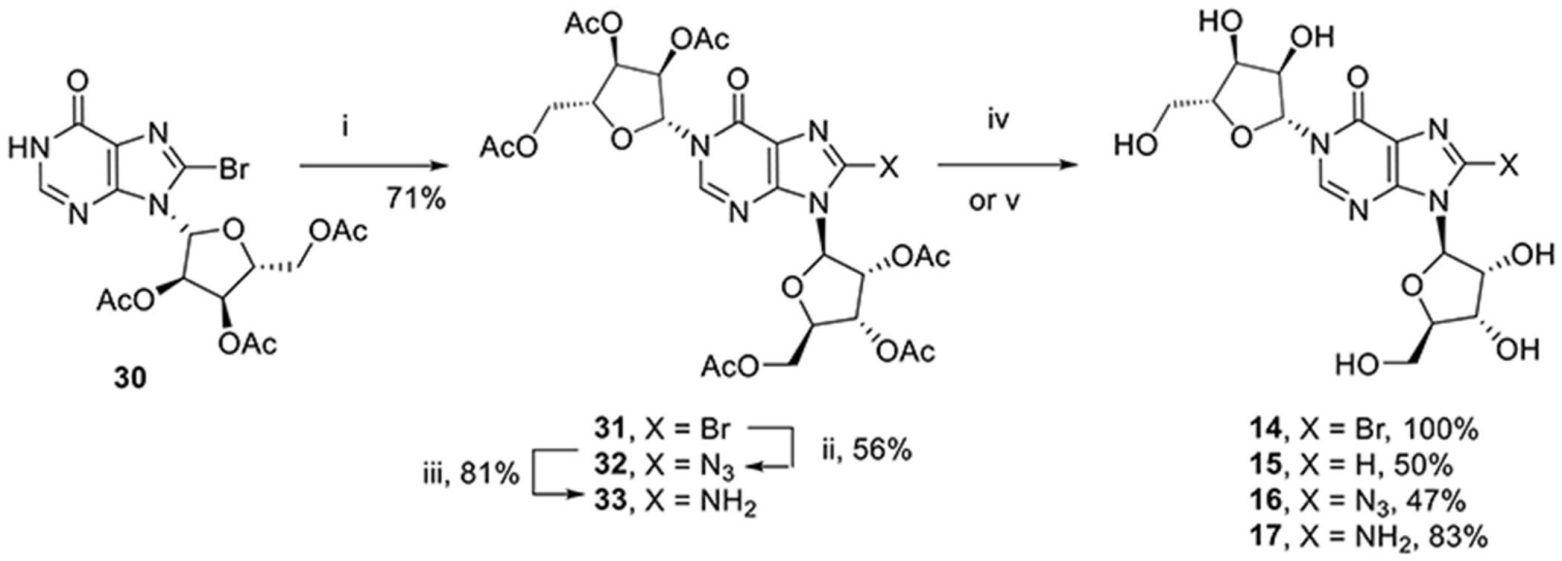
Synthesis of *N*1-ribosyl inosine analogues. Reagents and conditions i)a) DBU, MeCN; b) tetra-acetyl-D-ribose, TMSOTf; ii) NaN_3_, DMF, 70 °C; iii) H_2_, Pd(C), EtOH; iv) NH_3_ (g), MeOH; v) H_2_, Pd(C), NaHCO_3_, EtOH.

Triacetyl protected 8-bromoinosine **30** was *N*1-ribosylated under standard conditions^33^ to generate protected *N*1-ribosyl-8-bromoinosine **31** (Scheme 2). Sequential substitution with sodium azide and reduction with palladium under an atmosphere of hydrogen afforded the protected 8-azido (**32**) and 8-amino (**33**) analogues respectively. All three analogues were separately treated with methanolic ammonia to afford the corresponding *N*1-ribosyl-8-X-inosine analogues (**14**, **16**-**17**). *N*1-ribosyl inosine (**15**) was prepared directly from **31** upon treatment with palladium under an atmosphere of hydrogen.

**Scheme 3:**
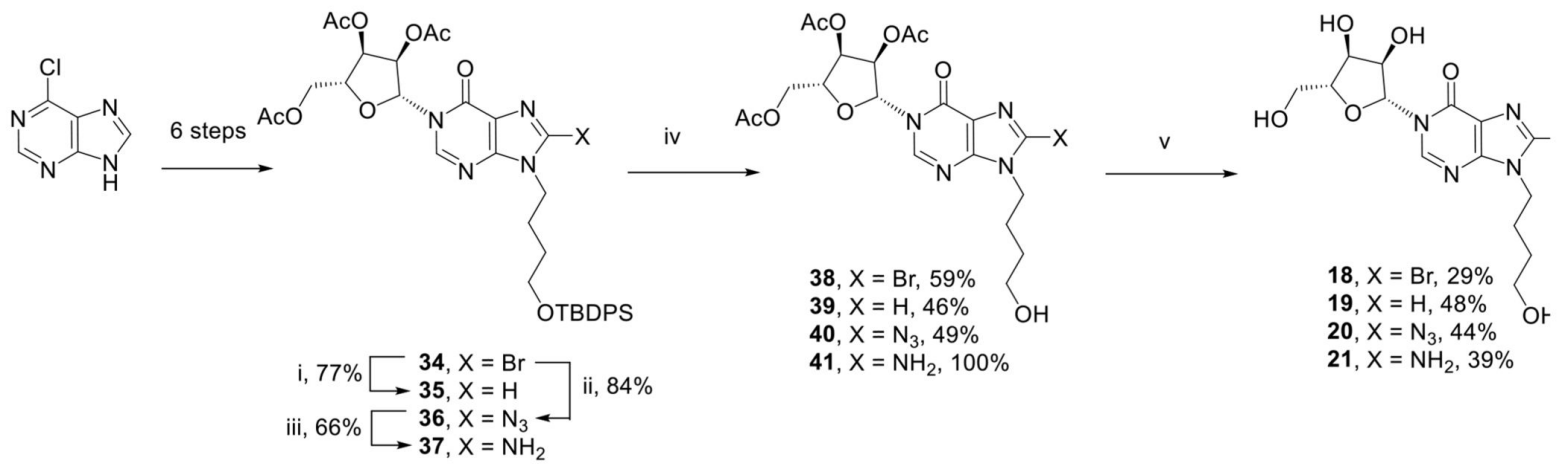
Synthesis of *N*9-hydroxybutyl-*N*1-inosine analogues. Reagents and conditions i) H_2_, Pd(C), NaHCO_3_, EtOH; ii) NaN_3_, DMF, 70 °C; iii) H_2_, Pd(C), EtOH; iv) TBAF.3H_2_O, AcOH, DMF; v) NH_3_ (g), MeOH.

We next prepared fragments with an *N*9-butyl linker in place of the “southern” ribose (*N*9-hydroxybutyl-*N*1-inosine derivatives, **18**-**21**, Scheme 3). This substitution in related cyclic analogues^36^ showed improved inhibition of CD38-mediated hydrolysis of cADPR.

8-Bromo-*N*9-(4-hydroxybutyl)-*N*1-inosine (**34**, Scheme 3) was prepared from 6-chloropurine, as previously described.^36^ Briefly, an *N*9-hydroxybutyl chain was introduced onto 6-chloropurine, that was converted in four steps to the protected 8-bromohypoxanthine. Treatment with DBU followed by TMSOTf and 1,2,3,5-tetra-*O*-acetyl-*β*-D-ribofuranose generated the *N*1-ribosyl scaffold **34**. To generate three further 8-substituted analogues, **34**, was treated with palladium on carbon under an atmosphere of hydrogen to generate the 8-H analogue **35**. 8-Bromo **34** was substitued in the 8-position using sodium azide in DMF at 70 °C to prepare 8-azido analogue **36**. Finally, 8-azido analogue **36** was reduced using palladium on charcoal under an atmosphere of hydrogen to prepare 8-amino analogue **37** (Scheme 3). All four protected analogues (**34**-**37**) were deprotected in a two stage process. First the *N*9-hydroxybutyl was revealed by treatment with neutral TBAF in DMF to prepare **38**-**41**, then the acetyl protecting groups were removed from the *N*1-ribose using saturated methanolic ammonia to prepare the final compounds (8-Br **18**, 8-H **19**, 8-N_3_ **20**, 8-NH_2_ **21**).

**Scheme 4:**
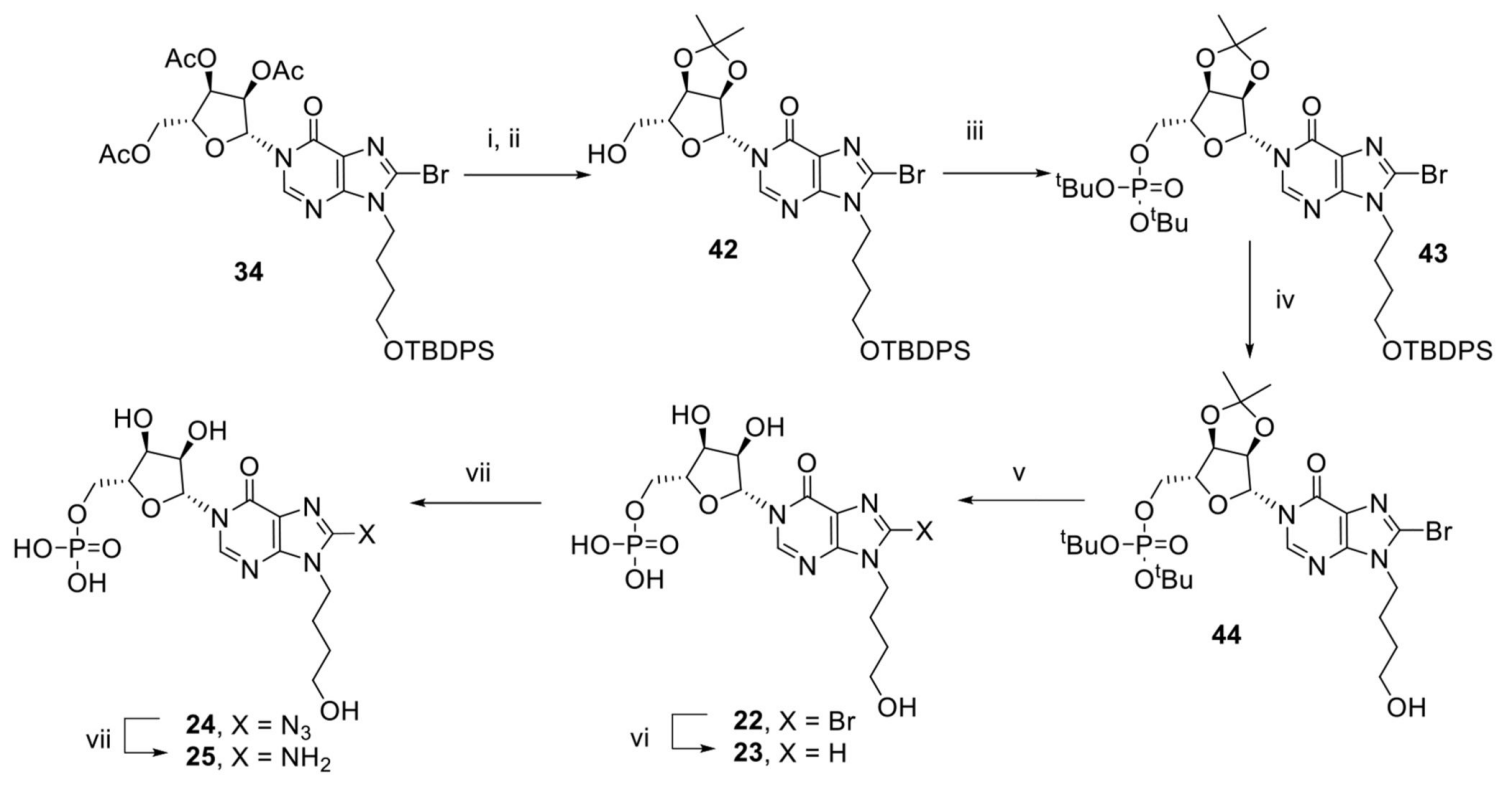
Synthesis of *N*9-(4-hydroxybutyl)-*N*1-IMP analogues. Reagents and conditions i) NH_3_, MeOH; ii) 2,2-dimethoxypropane, acetone, *p*TsOH; iii)a) ^*i*^Pr_2_NP(O^*t*^Bu)_2_, 5-Ph-1*H*-tetrazole, DCM; b) H_2_O_2_, Et_3_N; iv) TBAF.3H_2_O, AcOH, DMF; v) aq. TFA; vi) H_2_, Pd(C), NaHCO_3_, EtOH-H_2_O (1:2 v/v); vii) TMS-N_3_, DMF; viii) dithiothreitol, 0.05 M TEAB.

To determine the importance of the 5′-*O*-phosphate group, *N*9-hydroxybutyl-*N*1-inosine derivatives (**22**-**25**) were also prepared (Scheme 4). 8-Bromo-*N*9-(4-hydroxybutyl)-*N*1-inosine (**34**) was deprotected and reprotected as the isopropylidene ketal **42** in preparation for introduction of the protected 5-*O*-phosphate to afford **43**. Attempts to introduce different 8-substituents to the hypoxanthine ring at this stage were not successful, presumably due to steric interference from the adjacent *N*9-butyl chain and bulky TBDPS group. Sequential deprotection of the TBDPS silyl ether with TBAF to afford **44**, followed by deprotection of both phosphate esters and the isopropylidene ketal using aqueous TFA furnished 8-bromo-*N*9-hydroxybutyl-*N*1-IMP **22**. The 8-bromo substituent was removed by hydrogenation to afford **23** or nucleophilic substitution with TMS-N_3_ to afford **24**, followed by reduction with dithiothreitol to afford **25**.

**Scheme 5:**
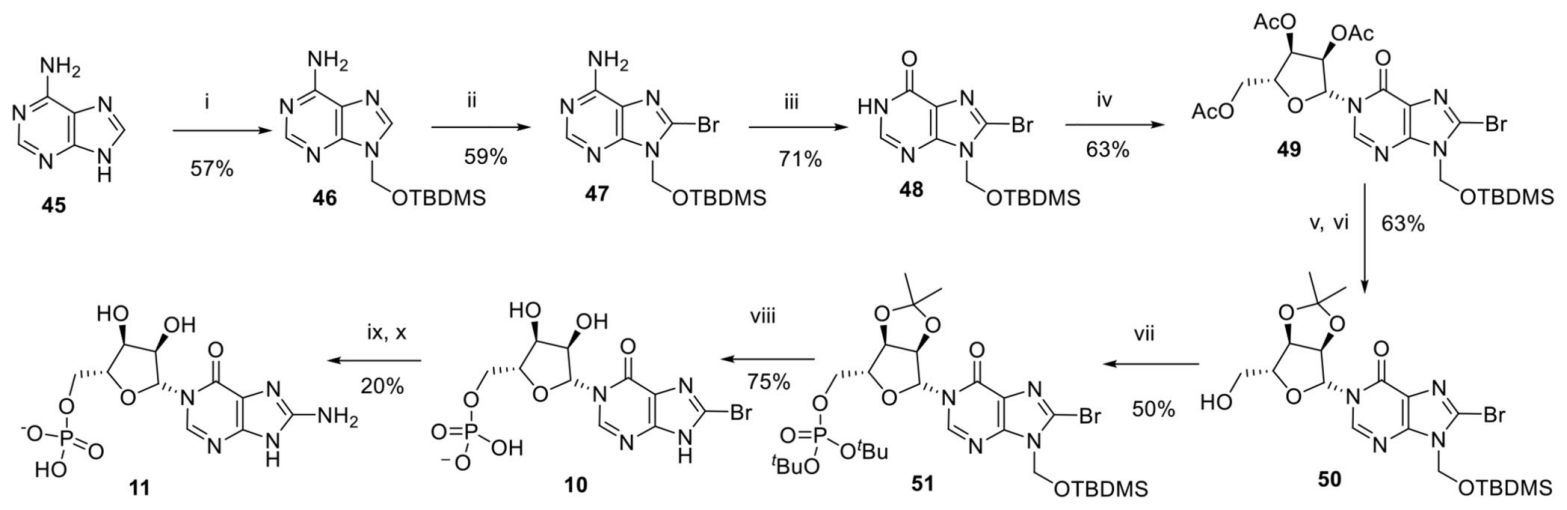
Preparation of 8-NH_2_-*N*1-IMP *via* a total synthetic route. Reagents and conditions i)a) Formaldehyde, 0.1 N NaOH, H_2_O; b) TBDMS-OTf, pyridine, 0 °C; ii)a) DIPA, *n*BuLi, THF, -78 °C; b) Br_2_; iii) NaNO_2_, AcOH-H_2_O; iv)a) DBU, DCM; b) tetra-acetyl-D-ribose then TMS-OTf; v) NH_3_, MeOH; vi) 2,2-dimethoxypropane, acetone, *p*TsOH; vii)a) ^*i*^Pr_2_NP(O^*t*^Bu)_2_, 5-Ph-1*H*-tetrazole, DCM; b) H_2_O_2_, Et_3_N; viii) aq. TFA; ix) TMS-N_3_, DMF; x) dithiothreitol, 0.05 M TEAB

We previously synthesized 8-NH_2_-*N*1-IMP (**11**) by destruction of 8-N_3_-cIDPR (**12**, Scheme 2) through acid catalyzed hydrolysis at elevated temperature.^39^ This route is however inefficient and thus we sought an alternative v*ia* total synthesis. Starting from adenine **45**, the *tert*-butyldimethylsilyloxymethyl group^43^ was introduced in a two-step procedure. In contrast to other *N*9 protecting groups, such as benzyl, benzoyl or acetyl, this generates an organically-soluble product that is considerably easier to handle. The *N*9-protected adenine **46** was then prepared for a regio- and stereoselective *N*1-glycosylation by introduction of a bromine group at *C*8 (**47**), followed by treatment with sodium nitrite to effect conversion from adenine to hypoxanthine base (**48**). *N*1-glycosylation was effected by treatment of **48** with 1,8-diazabicyclo[5.4.0]undec-7-ene (DBU), followed by trimethylsilyl triflate (TMSOTf) and 1,2,3,5-tetra-*O*-acetyl-*β*-D-ribofuranose to afford **49**. The three acetyl protecting groups were removed using methanolic ammonia and exchanged for a 2′,3′-*O*-isopropylidene group by treatment with 2,2-dimethoxypropane and acetone under acidic conditions to afford **50**. The protected precursor **50** was phosphitylated at the free 5-OH using di-*tert*-butyl protected phosphoramidite, followed by oxidation to the corresponding phosphate using hydrogen peroxide and triethylamine to afford **51**. A convenient global deprotection of the *tert*-butyldimethylsilyloxymethyl, isopropylidene ketal and two *tert*-butyl phosphate esters using 50% aqueous TFA generated **10**, the *N*1-IMP scaffold with an 8-bromo substituent. This could be conveniently manipulated to generate 8-NH_2_-*N*1-IMP **11** by sequential treatment with TMS-N_3_ and dithiothreitol (Scheme 5). Attempts to convert the 8-Br substituent to the 8-N_3_ analogue at an earlier stage in the synthesis were unsuccessful as the *N*7-*tert*-butyldimethylsilyloxymethyl protecting group was cleaved concurrently.

**Scheme 6:**
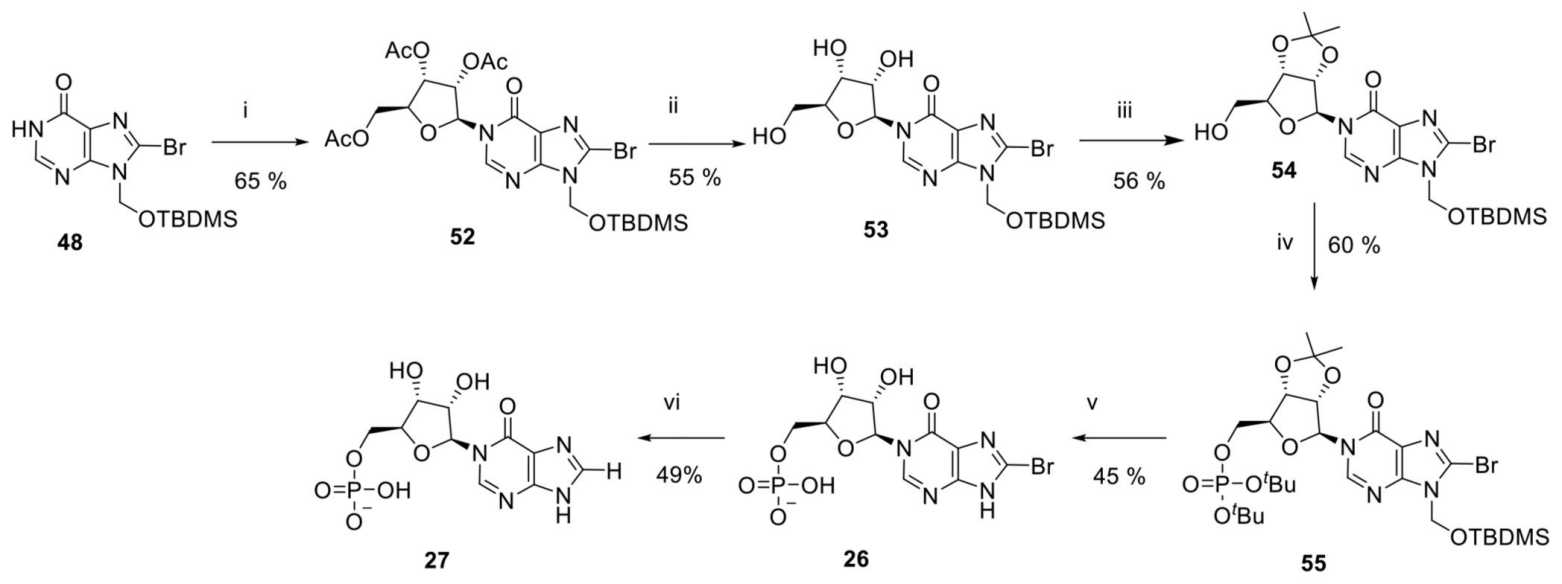
Synthesis of L-ribose-*N*1-IMP analogues. Reagents and conditions i)a) DBU, DCM; b) tetra-acetyl-L-ribose then TMS-OTf;ii) NH_3_, MeOH; iii) 2,2-dimethoxypropane, acetone, *p*TsOH; iv)a) ^*i*^Pr_2_NP(O^*t*^Bu)_2_, 5-Ph-1*H*-tetrazole, DCM; b) H_2_O_2_, Et_3_N; v) aq. TFA; vi) H_2_, Pd(C), NaHCO_3_, EtOH-H_2_O (1:2 v/v).

We next sought to explore the SAR of these more accessible, less negatively charged molecules. Following on from our earlier interesting results with inhibition of CD38 from compounds with an L-configuration “northern” ribose,^40^ we synthesied fragments with an L-ribose (Scheme 6). Briefly, *N*1-glycosylation of **48** was effected by treatment with DBU, TMSOTf and 1,2,3,5-tetra-*O*-acetyl-β-L-ribofuranose to afford **52**. Treatment with methanolic ammonia removed the three acetyl groups (**53**), followed by introduction of a 2′,3′-*O*-isopropylidene ketal to afford **54**. The protected precursor was phosphitylated at the free 5-OH and oxidized using the methods described above to afford **55**. Global deprotection using 50% aqueous TFA generated **26**, the L-*N*1-IMP scaffold with an 8-bromo substituent. The 8-bromo substituent was reduced to generate L-*N*1-IMP **27** using hydrogenation with palladium on carbon.

**Scheme 7:**
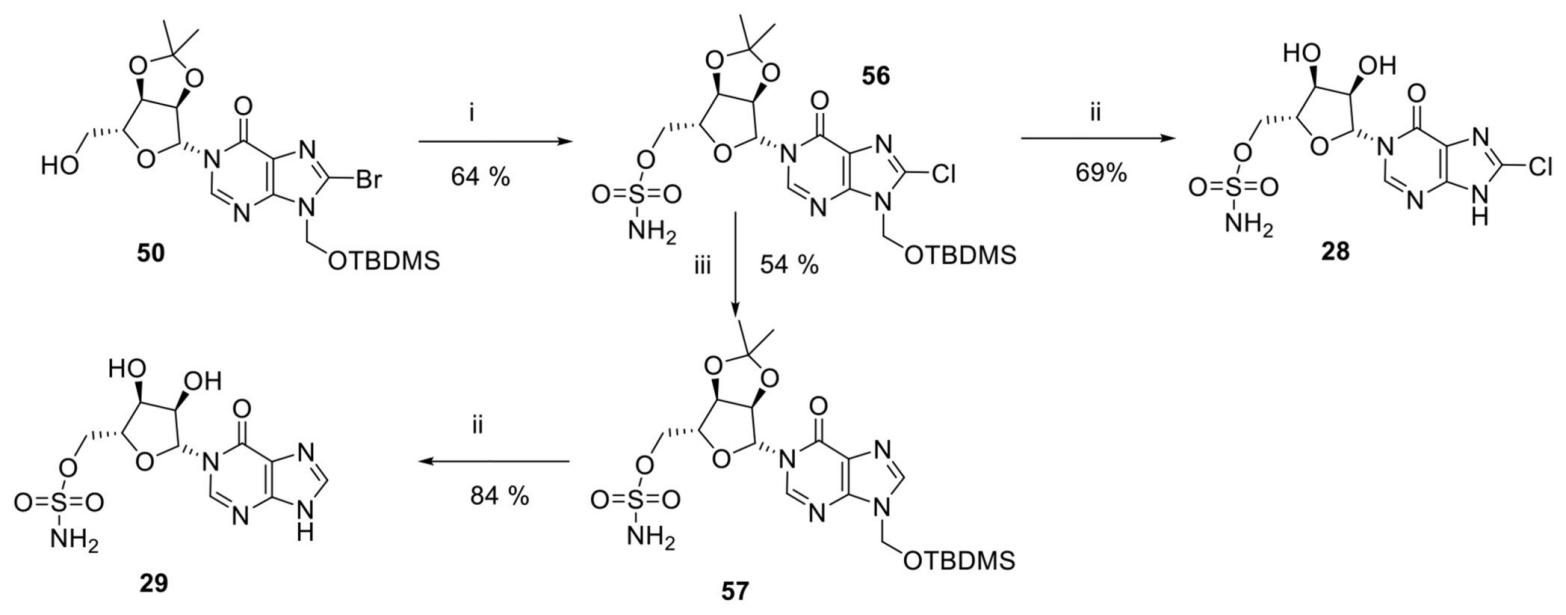
Synthesis of 5′-*O*-sulfonamyl-*N*1-inosine analogues. Reagents and conditions i) H_2_NSO_2_Cl in toluene (0.44 M), TEA, DCM; ii) aq. TFA; iii) H_2_, Pd(C), NaHCO_3_, EtOH.

We next explored the potential for *N*1-phosphate replacement with a bioisostere (Scheme 7). Phosphate bioisosteres present binding partners without negative charge, which is more attractive for drug design.^43^

*N*1-(*β*-D-Ribofuranosyl)-*N*9-*tert*-butyldimethylsilyloxymethyl-8-bromohypoxanthine (**50**) was treated with triethylamine followed by addition of sulfamoyl chloride. In addition to the desired introduction of a 5-*O*-sulfonamide group, the 8-bromo substituent was also substituted by an 8-chloro substituent in the reaction mixture, confirmed by MS (ES^+^ 588.13 and 590.13, 3:1) to afford **56**. Deprotection of the *N*9-protecting group with aqueous TFA gave the 8-chloro sulfonamide analogue **28**. Removal of the 8-chloro substituent by treatment with palladium on carbon under an atmosphere of hydrogen gave the *N*9-protected parent analogue **57**, which was then deprotected using aqueous TFA to give sulfonamide analogue **29**.

### Fragments as Inhibitors of cADPR Hydrolysis

All fragments were tested as inhibitors of CD38-catalyzed cADPR hydrolysis (Table).

Analogues with an *N*1-ribosyl group (**14**-**17**) were poor inhibitors of CD38. The 8-Br (**14**) and 8-N_3_ (**16**) analogues showed no activity and the 8-H (**15**) and 8-NH_2_ (**17**) analogues both only showed low mM inhibitory activity. The 8-bromo analogue **18** was insoluble in the assay medium, suggesting that the combination of the *N*9-hydroxybutyl group and the hydrophobic 8-bromine reduced the polarity of the fragment compared to the 8-H, -N_3_ and -NH_2_ analogues. *N*9-Hydroxybutyl-*N*1-inosines (**19**-**21**) were low mM inhibitors, possibly showing a marginal improvement compared to the *N*1-ribosyl-inosine analogues (**14**-**17**). Taken together, the poor inhibitory activity of analogues **14**-**21** suggests that the *N*1-ribose-5′-phosphate group is an essential feature for inhibitory activity.

The *N*9-hydroxybutyl-*N*1-IMPs (**23**-**25**) showed improved inhibitory activity compared to their non-phosphorylated *N*9-hydroxybutyl counterparts (**18**-**21**), with the most active being 8-N_3_ analogue **24** (IC_50_ = 201 μM). *N*9-hydroxybutyl-*N*1-IMPs (**23**-**25**) showed a 40-fold reduction in activity compared to the parent *N*1-IMPs (**9**-**11**). This is the opposite trend observed with the cyclic analogues in previous studies^36^, perhaps due to the unconstrained nature of the hydrophobic butyl chain in this case, compared to in a restricted cyclic analogue.

The two analogues with an L-ribose as the *N*1-ribose configuration (**26**, **27**) showed contrasting activity. While the activity of 8-Br analogue **26** was similar to the 8-Br *N*1-IMP analogue **10**, the 8-H analogue **27** showed a 30-fold reduction in activity compared to its D-ribose counterpart **9** (IC_50_ = 460 and 14 μM repectively). In other studies, where the L-ribose was constrained as part of a cyclic analogue, the L-ribose substitution highlighted differences in binding activity that were attributed to likely different binding modes.^40^ For fragments such as **26** and **27**, however, there would be free rotation around the *N*1-ribosyl bond and a smaller overall ligand to fit into the binding pocket.

CD38 is predominantly an ectoenzyme, but to a small degree its catalytic site can also face the intracellular environment, *eg* Type III CD38 has its *C*-terminal facing intracellularly, CD38 is present in the nucleus and mitochondrial membrane and a soluble form of CD38 is likely present in the cytoplasm^14^. Thus, approaches to neutralise the mono-phosphate charges of inhibitors could be useful for wider targeting of the enzyme. Attempted phosphate replacement with a sulfonamide bioisostere (Scheme 7), however, did not generate CD38 inhibitors, as neither of the two analogues (**28**, **29**) showed any activity. For further development there are obviously many further phosphate bioisteres that could be explored, as well as perhaps more importantly alternative methods to mask phosphate negative charges, such as acetoxymethyl-esters and the Protide approach, using groups that may be cleaved intracellularly.^43^

### Ligand hydrolysis by CD38

**Figure 4:**
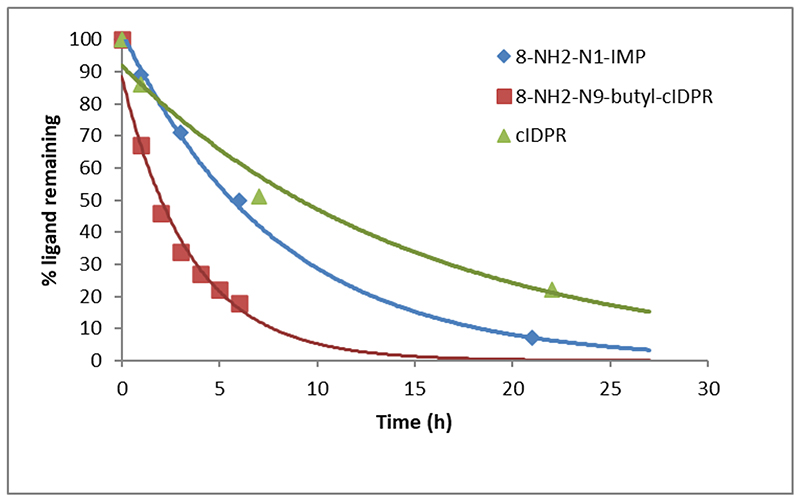
Hydrolysis of 8-NH_2_-*N*1-IMP by high concentrations of CD38

Previous studies demonstrated that cADPR analogues inhibiting CD38-mediated hydrolysis could be turned over by the high concentrations of CD38 catalytic domain (shCD38) present in crystallography media.^36^ Indeed, 8-NH_2_-*N*9-butyl-cIDPR (**6**, Figure 2) was captured as the hydrolyzed product in the crystal structure with shCD38. Demonstration of hydrolysis by shCD38 using HPLC suggests that the fragment is indeed binding to the cADPR pocket, probably in an orientation that places the *N*1-ribosyl bond within reach of the catalytic residue. Incubation of **11**, 8-NH_2_-*N*1-IMP (1 mM final concentration) with 4 mg/mL shCD38 was monitored using RP-HPLC. The peak corresponding to 8-NH_2_-*N*1-IMP (R_T_ = 10.5 min) reduced in intensity over time, alongside the appearance of a new peak (R_T_ = 2.0 min) that was characteristic of an 8-amino substituted hypoxanthine analog (See Supplementary Information, Figure S1). No change in the original peak was observed in a parallel control experiment containing no shCD38 (data not shown). 8-NH_2_-*N*1-IMP (**11**) was hydrolyzed more rapidly than cIDPR (**2**), but more slowly than 8-NH_2_-*N*9-butyl-cIDPR (**6**) (Figure 4).

Both 8-NH_2_-substituted analogues are more potent inhibitors of CD38-mediated hydrolysis than cIDPR (**2**) (IC_50_ values of 7.6 and 3.3 μM compared to 276 μM). Perhaps most surprising is that the small fragment, 8-NH_2_-*N*1-IMP (**11**) is hydrolyzed at the *N*1-ribosyl bond, suggesting that it not only binds in the active site of CD38 but also that it orientates itself to bind with the *N*1-ribosyl bond acessible to the catalytic residue. This would seem more likely for the larger cyclic ligands and adds further weight to the argument that the “northern” ribose makes key interactions in the CD38 binding site.^36, 40^.

## Conclusions

Five fragment scaffolds were prepared, each with multiple 8-substitutions. *N*1-ribosyl-inosine derivatives **14-17** and *N*9-Hydroxybutyl-*N*1-inosine derivatives **18-21** are non-phosphorylated analogues that retain the key “northern” ribose motif. These analogues illustrate the importance of the 5′-phosphate group on the “northern” ribose for CD38 inhibitory activity. Introduction of the 5′-phosphate group to in *N*9-hydroxybutyl-*N*1-IMP analogues **22-25** shows some improvement in activity; however, the unconstrained *N*9-butyl chain appeared to be detrimental, compared to its effect in cyclic analogues.^36^ The promising fragment 8-NH_2_-*N*1-IMP (**11**) was prepared *via* total synthesis for the first time, which affords a route to generate this analogue in more significant amounts (compared alternatively to the previously reported degradation of the cyclic parent analogue) and to access other related analogues for SAR studies. To illustrate the utility of this new synthetic route, L-ribose (**26**-**27**) and sulphonamide (**28**-**29**) analogues were prepared. In summary, this work illustrates the potential for design of much simpler and mono-phosphorylated CD38 inhibitors, through a key structural motif derived from from its macrocyclic pyrophosphate ligand, that could be worthy of future optimisation and development. Importantly, their continuing, albeit weak, substrate activity implies that such compounds bind closely mimicking the relevant part of the natural ligand, which should aid structure-based design strategies. CD38 while generally an ectoenzyme, does also exist inside cells, so the reduction of the inhibitor class to a simple monophosphate derivative as here makes available well-established prodrug strategies that should improve inhibitor access.

## Experimental

### General

All reagents and solvents were of commercial quality and were used without further purification, unless described otherwise. Unless otherwise stated, all reactions were carried out under an inert atmosphere of argon. ^1^H, ^13^C, and ^31^P-NMR spectra were collected on a Varian Mercury 400 MHz or Bruker Avance III 500 MHz spectrometer. All ^1^H and ^13^C NMR assignments are based on gCOSY, gHMBC, gHSQC, and DEPT-135 experiments. Abbreviations for splitting patterns are as follows: br, broad; s, singlet; d, doublet; t, triplet; m, multiplet. Coupling constants are given in Hertz (Hz). High resolution time-of-flight mass spectra were obtained on a Bruker Daltonics micrOTOF mass spectrometer using electrospray ionization (ESI). The purity of new tested compounds was determined to be ≥95% by analytical HPLC (See Supplementary Information). Analytical HPLC analyses were carried out on a Waters 2695 Alliance module equipped with a Waters 2996 photodiode array detector (210–350 nm). The chromatographic system consisted of a Hichrom Guard column for HPLC and a Phenomenex Synergi 4 μm MAX-RP 80A column (150 mm × 4.60 mm), with elution at 1 mL/min with either a) ion-pair buffer: 0.17% (m/v) cetrimide and 45% (v/v) phosphate buffer (pH 6.4) in MeOH or b) a gradient of 0.05M TEAB:MeCN (95:5 → 35:65 v/v). Synthetic phosphates were assayed and quantified by the Ames phosphate test.^44^ Non-phosphate final compounds were quantified by quantitative ^1^H-NMR.

#### Total synthesis of 8-NH_2_-*N*1-IMP (11)

##### *N*9-*tert*-Butyldimethylsilyloxymethyl-adenine^45^ (46)

Prepared from adenine following the method of Lang et al.^45^

##### *N*9-*tert*-Butyldimethylsilyloxymethyl-8-bromoadenine (47)

To diisopropylamine (2.93 mL, 20.94 mmol) in THF (10 mL) at -78 °C was added *n*-butyllithium (8.55 mL, 2.5 M solution, 21.37 mmol), dropwise. After 1 h, a solution of *N*9-*tert*-Butyldimethylsilyloxymethyl-adenine (**46**, 1.17 g, 4.19 mmol) in THF (40 mL) was added dropwise and stirring continued for a further 1 h. Br_2_ (837 μL, 16.76 mmol) was added dropwise and the solution allowed to warm to rt and stirred for 16 h. The reaction was quenched by addition of NaOAc/AcOH (pH 4, 1N aq., 2mL) and all solvents evaporated. The residue was taken up in DCM:H_2_O (1:1 v/v, 100 mL), the organic layer separated, washed with brine, dried (Na_2_SO_4_) and evaporated to dryness. The crude material was purified by column chromatography on silica gel eluting with DCM:Acetone (1:0 → 0:1 v/v) to afford the *title compound* (879 mg, 59%) as an amorphous cream solid; *R_f_* = 0.59 (DCM/Acetone 1:1 v/v); ^1^H-NMR (400 MHz, CDCl_3_) δ 8.32 (s, 1H, H-2), 5.94 (br s, 2H, NH_2_), 5.71 (s, 2H, CH_2_OTBDMS), 0.86 (s, 9H) 0.10 (s, 6H) (15H, TBDMS); ^13^C-NMR (100 MHz, CDCl_3_) δ 154.1, 153.2, 151.03, 127.3, 119.7, 67.0, 25.5 (3C), 18.0 and -5.3 (2C); HRMS (ES^+^) calcd for C_12_H_21_N_5_OSi^79^Br 358.0693 (M+H)^+^ found 358.0699, calcd for C_12_H_21_N_5_OSi^81^Br 360.0678 (M+H)^+^ found 360.0691.

##### *N*9-*tert*-Butyldimethylsilyloxymethyl-8-bromohypoxanthine (48)

*N*9-*tert*-Butyldimethylsilyloxymethyl-8-bromoadenine (**47**, 600 mg, 1.67 mmol) was taken up in acetic acid-water (46 mL, 20:3 v/v). NaNO_3_ (1.39 g, 20.09 mmol) was added in one portion and the resulting solution stirred at 50 °C for 16 h. All solvents were evaporated and the residue partitioned between DCM and H_2_O, and the organic layer washed with NaHCO_3_ (sat. aq.), then brine, dried (Na_2_SO_4_) and evaporated to dryness. The residue was purified by column chromatography on silica gel eluting with DCM/Acetone (1:0 → 0:1 v/v) to afford the title compound (430 mg, 71%) as a pale orange amorphous solid; *R_f_* = 0.57 (DCM/Acetone 1:1 v/v) ; ^1^H-NMR (400 MHz, CDCl_3_) δ 13.15 (br s, 1H, NH), 8.31 (s, 1H, H-2), 5.71 (s, 1H), 5.70 (s, 1H) (2H, CH_2_OTBDMS), 0.87 (s, 9H) 0.12 (s, 6H) (15H, TBDMS); ^13^C-NMR (100 MHz, CDCl_3_) δ 158.0, 150.2, 145.8, 126.4, 124.6, 67.5, 25.5 (3C), 18.0 and -5.3 (2C); HRMS (ES^+^) calcd for C_12_H_20_N_4_O_2_Si^79^Br 359.0539 (M+H)^+^ found 359.0544, calcd for C_12_H_20_N_4_O_2_Si^81^Br 361.0519 (M+H)^+^ found 361.0536.

##### *N*1-(2′,3′,5′-Tri-*O*-acetyl-β-D-ribofuranosyl)-*N*9-*tert*-butyldimethylsilyloxymethyl-8-bromohypoxanthine (49)

To *N*9-*tert*-butyldimethylsilyloxymethyl-8-bromohypoxanthine (**48**, 480 mg, 1.336 mmol) in DCM (5 mL) was added DBU (599 μL, 4.008 mmol). After 30 min, 1,2,3,5-tetra-*O*-acetyl-β-D-ribofuranose (468 mg, 1.47 mmol) was added and the solution cooled to −78 °C. Trimethylsilyl trifluoromethanesulfonate (967 μL, 5.344 mmol) was added dropwise and the solution stirred for a further 45 min before warming to rt. After 2 h, NaHCO_3_ (satd aq) was added and the crude material extracted into DCM (×3). The combined organic fractions were dried (Na_2_SO_4_), and solvent was evaporated under reduced pressure. The residue was purified by column chromatography on silica gel eluting with PE/EtOAc (1:0 → 0:1 v/v) to afford the *title compound* (517 mg, 63%) as a colourless glass; *R_f_* = 0.64 (PE:EtOAc 1:3 v/v) ;^1^H-NMR (400 MHz, CDCl_3_) δ 8.20 (s, 1H, H-2), 6.35 (d, *J* = 4.5 Hz, 1H, H-1′), 5.64 (d, *J* = 9.8 Hz, 1H), 5.62 (d, *J* = 9.8 Hz, 1H) (2H, CH_2_OTBDMS), 5.46-5.41 (m, 2H, H-2′, H-3′), 4.43-4.33 (m, 3H, H-4′, 2 × H-5′), 2.15 (s, 3H, AcetylCH_3_), 2.10 (s, 3H, AcetylCH_3_), 2.06 (s, 3H, AcetylCH_3_), 0.86 (s, 9H) 0.12 (s, 3H) and 0.11 (s, 3H) (15H, TBDMS); ^13^C-NMR (100 MHz, CDCl_3_) δ 170.2, 169.6 (2C), 154.7, 148.4, 144.5, 126.1, 123.9, 87.4, 80.4, 74.2, 70.1, 67.3, 62.9, 25.4 (3C), 20.7, 20.44, 20.38, 18.0, -5.2, -5.3; HRMS (ES^+^) calcd for C_23_H_34_N_4_O_9_Si^79^Br 617.1273 (M+H)^+^ found 617.1281, calcd for C_23_H_34_N_4_O_9_Si^81^Br 619.1252 (M+H)^+^ found 619.1241.

##### *N*1-(2′,3′-*O*-Isopropylidene-β-D-ribofuranosyl)-*N*9-*tert*-butyldimethylsilyloxymethyl-8-bromohypoxanthine (50)

*N*1-(2′,3′,5′-Tri-*O*-acetyl-β-D-ribofuranosyl)-*N*9-*tert*-butyldimethylsilyl oxymethyl-8-bromohypoxanthine (**49**, 370 mg, 0.599 mmol) was taken up in MeOH (5.0 mL) in a pressure tube. The solution was cooled to 0 °C in an ice-water bath and NH_3_ (g) bubbled through the solution to saturation. The tube was sealed and the resulting solution stirred at rt. When complete by TLC, the solvents were removed by evaporation under reduced pressure and the residue was purified by column chromatography on silica gel eluting with DCM/Acetone (1:0 → 0:1 v/v) to afford *N*1-(β-D-Ribofuranosyl)-*N*9-*tert*-butyldimethylsilyloxymethyl-8-bromohypoxanthine as an amorphous white solid, *R_f_* = 0.21 (DCM:Acetone 1:1 v/v), which was used directly in the next step. HRMS (ES^+^) calcd for C_17_H_27_N_4_O_6_Si^79^BrNa 513.0775 (M+Na)^+^ found 513.0780, calcd for C_17_H_27_N_4_O_6_Si^81^BrNa 515.0755 (M+Na)^+^ found 515.0758.

To crude *N*1-(β-D-ribofuranosyl)-*N*9-*tert*-butyldimethylsilyloxymethyl-8-bromohypoxanthine in 2,2-dimethoxypropane-acetone (1:4 v/v, 5 mL) was added *para*-toluenesulfonic acid (114 mg, 0.599 mmol). After 1h, DCM and NaHCO_3_ (satd. aq.) were added and the aqueous phase extracted with DCM (×3). The combined organic extracts were evaporated to dryness and purified by column chromatography on silica gel eluting with PE/EtOAc (1:0 → 0:1 v/v) to afford the *title compound* (200 mg, 63% over two steps) as a colourless glass. *R_f_* = 0.65 (PE:EtOAc 1:3 v/v); ^1^H-NMR (400 MHz, CDCl_3_) δ 8.05 (s, 1H, H-2), 5.73 (d, *J* = 2.8 Hz, 1H, H-1′), 5.64 (d, *J* = 9.8 Hz, 1H), 5.62 (d, *J* = 9.8 Hz, 1H) (2H, CH_2_OTBDMS), 5.27 (dd, *J* = 6.5, 2.8 Hz, 1H, H-2′), 5.12 (dd, *J* = 6.5, 3.5 Hz, 1H, H-3′), 4.34 (ddd, *J* = 6.1, 5.6, 3.5 Hz, 1H, H-4′), 3.91 (ddd, *J* = 11.9, 5.6, 3.3 Hz, 1H, H-5′_a_), 3.83 (ddd, *J* = 11.9, 7.8, 6.1 Hz, 1H, H-5′_b_), 3.40 (dd, *J* = 7.8, 3.3 Hz, 1H, 5-OH), 1.57 (s, 3H, CHCH_3_), 1.34 (s, 3H, CHCH_3_), 0.86 (s, 9H) 0.11 (s, 3H) and 0.10 (s, 3H) (15H, TBDMS); ^13^C-NMR (100 MHz, CDCl_3_) δ 155.2, 148.8, 146.9, 126.5, 124.6, 114.2, 96.9, 88.0, 83.5, 80.6, 67.4, 62.8, 27.2, 25.4, 25.1, 17.9, -5.2 (2C); HRMS (ES^+^) calcd for C_20_H_32_N_4_O_6_Si^79^Br 531.1269 (M+H)^+^ found 531.1248, calcd for C_20_H_32_N_4_O_6_Si^81^Br 533.1249 (M+H)^+^ found 533.1246.

##### *N*1-[2′,3′-*O*-Isopropylidene-5′-*O*-(di-*tert*-butyl)-phosphoryl-β-D-ribofuranosyl]-*N*9-*tert*-butyldimethylsilyloxymethyl-8-bromohypoxanthine (51)

To *N*1-(2′,3′-*O*-isopropylidene-β-D-ribofuranosyl)-*N*9-*tert*-butyldimethylsilyloxymethyl-8-bromohypoxanthine (**50**, 80 mg, 0.151 mmol) in DCM (0.8 mL) was added 5-phenyl-1*H*-tetrazole (44 mg, 0.301 mmol) and the solution cooled to 0 °C. Di-*tert*-butyl *N,N*-diisopropylphosphoramidite (71 μL, 0.227 mmol) was added dropwise and the solution stirred at rt until phosphitylation was complete by TLC. After cooling to 0 °C, triethylamine (126 μL, 0.906 mmol) and H_2_O_2_ (30% in H_2_O, 33 μL, 0.375 mmol) were added and the solution stirred at rt until oxidation was complete. The reaction was diluted with DCM and washed with NaHCO_3_ (satd. aq.), dried over Na_2_SO_4_ and purified by column chromatography on silica gel eluting with PE/EtOAc (1:0 → 0:1 v/v + 0.5% pyridine in each solvent) to afford the *title compound* (55 mg, 50%) as a colourless glass; *R_f_* = 0.46 (PE:EtOAc 1:3 v/v) – note there is no change in retention time compared to starting material (after oxidation); ^1^H-NMR (500 MHz, CDCl_3_) δ 8.00 (s, 1H, H-2), 5.90 (d, *J* = 1.8 Hz, 1H, H-1′), 5.54 (d, *J* = 10.4 Hz, 1H), 5.52 (d, *J* = 10.4 Hz, 1H) (2H, CH_2_OTBDMS), 4.96 (dd, *J* = 6.4, 1.8 Hz, 1H, H-2′), 4.88 (dd, *J* = 6.4, 4.1 Hz, 1H, H-3′), 4.32 (ddd, *J* = 6.5, 5.9, 4.1 Hz, 1H, H-4′), 4.14 (ddd, *J* = 11.2, 6.5, 4.2 Hz, 1H, H-5′_a_), 4.08 (ddd, *J* = 11.2, 6.8, 5.9 Hz, 1H, H-5′_b_), 1.47 (s, 3H, CHCH_3_), 1.354 (s, 9H, O^*t*^Bu), 1.346 (s, 9H, O^*t*^Bu), 1.23 (s, 3H, CHCH_3_), 0.76 (s, 9H) 0.01 (s, 3H) and 0.00 (s, 3H) (15H, TBDMS); ^13^C-NMR (125MHz, CDCl_3_) δ 154.8, 148.7, 146.2, 126.1, 124.4, 114.4, 94.1, 86.8 (d, *J* = 7.6 Hz), 85.1, 82.7 (d, *J* = 7.5 Hz), 81.3, 67.4, 66.4 (d, *J* = 6.3), 29.84 (3C, d, *J* = 4.1 Hz), 29.81 (3C, d, *J* = 3.7 Hz), 27.2, 25.5 (3C), 25.3, 18.0, -5.2 (2C); HRMS (ES^+^) calcd for C_28_H_48_N_4_O_9_Si^79^BrNa 745.2004 (M+Na)^+^ found 745.1990, calcd for C_28_H_48_N_4_O_9_Si ^81^BrNa 747.1983 (M+Na)^+^ found 747.1964.

##### *N*1-(5′-*O*-Phosphoryl-β-D-ribofuranosyl)-8-bromohypoxanthine (8-Br-*N*1-IMP, 10)

*N*1-[2′,3′-*O*-Isopropylidene-5′-*O*-(di-*tert*-butyl)-phosphoryl-β-D-ribofuranosyl]-*N*9-*tert*-butyldimethylsilyloxymethyl-8-bromohypoxanthine (**51**, 55 mg, 76 μmol) was treated with TFA (50% aq., 4 mL) for 16h. All solvents were evaporated and the residue evaporated from MeOH × 3 to give the *title compound* (24 mg, 75%). ^1^H-NMR and MS data was as previously described^39^ and it was used in the next step without further purification.

##### *N*1-(5′-*O*-Phosphoryl-β-D-ribofuranosyl)-8-aminohypoxanthine (8-NH_2_-*N*1-IMP, 11)

To *N*1-(5′-*O-*phosphoryl-β-D-ribofuranosyl)-8-bromohypoxanthine (**10**, 24 mg, 56 μmol) in DMF (1 mL) was added TMSN_3_ (50 μL) and the solution stirred at 70 °C for 16h. All solvents were evaporated and the crude *N*1-(5′-*O*-Phosphoryl-β-D-ribofuranosyl)-8-azidohypoxanthine (8-N_3_-*N*1-IMP) product used directly in the following step.

To crude *N*1-(5′-*O*-phosphoryl-β-D-ribofuranosyl)-8-azidohypoxanthine in TEAB (0.05 M, 5 mL) was added dithiothreitol (5 mg, 32 μmol) and the solution stirred at rt for 16h before purification by semipreparative HPLC (1.1 cm × 25 cm C18 column), eluting with acetonitrile/0.1 M TEAB (1:19 → 13:7 v/v) over 25 min. Fractions were analyzed by analytical HPLC and appropriate fractions collected and evaporated under vacuum to give the *title compound* (5.3 mg, 20%). ^1^H-NMR (500 MHz, D_2_O) δ 8.42 (s, 1H, H-2), 6.32 (d, *J* = 3.3 Hz, 1H, H-1′), 4.31-4.35 (m, 2H, H-2′ and H-3′), 4.24 (brs, 1H, H-4′), 4.15-4.18 (m, 1H, H-5′_a_) and 4.07-4.03 (m, 1H, H-5′_b_). ^13^C-NMR (125 MHz, D_2_O) δ 161.4 (C-6), 155.4 (C-4), 152.7 (C-5), 143.7 (C-2), 112.2 (C-8), 89.1 (C-1′), 83.0 (d, *J* = 8.9 Hz, C-4′), 75.0 (C-2′), 69.0 (C-3′) and 63.6 (d, *J* = 4.5 Hz, C-5′). ^31^P-NMR (202 MHz, D_2_O) δ 0.36. HRMS (ES^−^) calcd for C_10_H_13_N_5_O_8_P 362.0507 (M−H)^−^ found 362.0516.

#### *N*1-Ribosyl-inosine analogues (14-17)

##### *N*1-(2″,3″,5″-Tri-*O*-acetyl-β-D-ribofuranosyl)-2′,3′,5′-tri-*O*-acetyl-8-bromoinosine (31)

2′,3′,5′-tri-*O-*acetyl-8-bromoinosine^33^ (**30**, 615 mg, 1.30 mmol) was taken up in DCM (6 mL) and DBU (583 μL, 3.90 mmol) added. After 30 min, 1,2,3,5-tetra-*O*-acetyl-β-D-ribofuranose (455 mg, 1.43 mmol) was added and the solution cooled to −78 °C. Trimethylsilyl trifluoromethanesulfonate (941 μL, 5.20 mmol) was added dropwise and the solution stirred for a further 45 min before warming to rt. After 1 h, NaHCO_3_ (satd aq) was added and the crude material extracted into DCM (×3). The combined organic fractions were dried (Na_2_SO_4_), and solvent was evaporated under reduced pressure. The residue was purified by column chromatography on silica gel eluting with DCM/Acetone (1:0 → 4:1 v/v) to afford the title compound (678 mg, 71%) as a colorless glass: *R_f_* = 0.69 (DCM:Acetone 3:1 v/v); ^1^H-NMR (400 MHz, CDCl_3_) δ 8.28(s, 1H, 2-H), 6.38 (d, *J* = 4.6 Hz, 1H), 6.20 (dd, *J* = 6.0, 4.7, Hz, 1H), 6.10 (d, *J* = 4.7 Hz, 1H), 5.79 (app. t, *J* = 5.8 Hz, 1H), 5.51-5.44 (m, 2H), 4.48-4.33 (m, 6H), 2.20 (s, 3H), 2.16 (s, 3H), 2.14 (s, 3H), 2.13 (s, 3H), 2.11 (s, 3H), 2.07 (s, 3H); ^13^C-NMR (100 MHz, CDCl_3_) δ 170.4, 170.3, 169.52, 169.47, 169.39, 169.29, 154.5, 148.1, 144.6, 126.3, 125.1, 88.7, 87.5, 80.3, 80.08, 74.2, 72.2, 70.3, 70.1, 63.1, 62.9, 20.8, 20.6, 20.48, 20.46, 20.39, 20.37; HRMS (ES^+^) calcd for C_15_H_20_N_4_O_9_^79^Br 479.0408 (M+H)^+^ found 479.0401, calcd for C_15_H_20_N_4_O_9_^81^Br 481.0388 (M+H)^+^ found 481.0379.

##### *N*1-(β-D-Ribofuranosyl)-8-bromoinosine (8-Br-*N*1-ribosyl-IMP, 14)

*N*1-(2″,3″,5″-Tri-*O*-acetyl-β-D-ribofuranosyl)-2′,3′,5′-tri-*O*-acetyl-8-bromoinosine (**31**, 107 mg, 0.146 mmol) was taken up in MeOH (2.0 mL) in a pressure tube. The solution was cooled to 0 °C in an ice-water bath and NH_3_ (g) bubbled through the solution to saturation. The tube was sealed and the resulting solution stirred at rt. When complete by TLC, the precipitate was removed by filtration and dried under vacuum to yield the *title compound* (70 mg, 100%) as a white amorphous solid; ^1^H-NMR (400 MHz, CD_3_OD) δ 8.89 (s, 1H, 2-H), 6.26 (d, *J* = 2.8 Hz, 1H), 6.10 (d, *J* = 6.4 Hz, 1H), 5.11 (dd, *J* = 6.4, 5.7, Hz, 1H), 4.46 (dd, *J* = 5.4, 2.8 Hz, 1H), 4.35-4.30 (m, 2H), 4.20-4.16 (m, 2H), 4.01 (dd, *J* = 12.3, 2.5 Hz, 1H), 3.91 (dd, *J* = 12.4, 3.3 Hz, 1H), 3.86 (dd, *J* = 12.3, 2.8 Hz, 1H), 3.79 (dd, *J* = 12.4, 4.2 Hz, 1H); ^13^C-NMR (100 MHz, CD_3_OD) δ 156.9, 149.7, 147.0, 128.5, 125.8, 92.9, 91.6, 88.2, 86.3, 76.9, 74.0, 72.4, 70.5, 63.7, 61.6; HRMS (ES^+^) calcd for C_15_H_20_N_4_O_9_^79^Br 479.0408 (M+H)^+^ found 479.0410, calcd for C_15_H_20_N_4_O_9_^81^Br 481.0388 (M+H)^+^ found 481.0401.

##### *N*1-(β-D-Ribofuranosyl)-inosine (*N*1-ribosyl-IMP, 15)

*N*1-(2″,3″,5″-Tri-*O*-acetyl-β-D-ribofuranosyl)-2′,3′,5′-tri-*O*-acetyl-8-bromoinosine (**31**, 330 mg, 0.451 mmol), NaHCO_3_ (42 mg, 0.496 mmol) and Pd/C (45 mg, 45 μmol) were taken up in EtOH (6 mL) and the solution degassed with argon before being placed under an atmosphere of H_2_ for 12 h. The catalyst was removed by filtration and the resulting solution purified by column chromatography on silica gel eluting with DCM/Acetone (1:0 → 0:1 v/v) to afford the *title compound* (90 mg, 50%) as a clear glass; *R_f_* = 0.27 (DCM:Acetone 4:1 v/v); ^1^H-NMR (500 MHz, CD_3_OD) δ 8.82 (s, 1H), 8.34 (s, 1H), 6.22 (d, *J* = 3.0 Hz, 1H), 6.00 (d, *J* = 5.7 Hz, 1H), 4.61 (dd, *J* = 5.7, 5.4 Hz, 1H), 4.31 (dd, *J* = 5.0, 3.7 Hz, 1H), 4.28-4.24 (m, 2H), 4.14-4.09 (m, 2H), 3.94 (dd, *J* = 12.3, 2.5 Hz, 1H), 3.85 (dd, *J* = 12.4, 3.0 Hz, 1H), 3.79 (dd, *J* = 12.4, 2.9 Hz, 1H), 3.74 (dd, *J* = 12.3, 3.3 Hz, 1H); ^13^C-NMR (125 MHz, CD_3_OD) δ 158.0, 148.7, 147.0, 141.4, 125.0, 91.4, 90.6, 87.6, 86.3, 76.9, 76.2, 72.1, 70.6, 63.0, 61.7; HRMS (ES^+^) calcd for C_15_H_21_N_4_O_9_ 401.1303 (M+H)^+^ found 401.1297.

##### *N*1-(2″,3″,5″-Tri-*O*-acetyl-β-D-ribofuranosyl)-2′,3′,5′-tri-*O*-acetyl-8-azidoinosine (32)

To *N*1-(2″,3″,5″-tri-*O*-acetyl-β-D-ribofuranosyl)-2′,3′,5′-tri-*O*-acetyl-8-bromoinosine (**31**, 240 mg, 0.328 mmol) in DMF (2.4 mL) was added NaN_3_ (68 mg, 1.05 mmol) and the resulting solution stirred at 70 °C in the dark for 72h. The solution was evaporated to dryness and the residue taken up in DCM, washed with H_2_O, dried over Na_2_SO_4_ and purified by column chromatography on silica gel eluting with PE/EtOAc (1:0 → 0:1 v/v) to afford the *title compound* (128 mg, 56%) as a clear glass; *R_f_* = 0.53 (PE:EtOAc 1:3 v/v); ^1^H-NMR (400 MHz, CDCl_3_) δ 8.16 (s, 1H, 2-H), 6.30 (d, *J* = 4.5 Hz, 1H), 6.02 (dd, *J* = 5.8, 4.9 Hz, 1H), 5.90 (d, *J* = 4.9 Hz, 1H), 5.67 (app. t, *J* = 5.8 Hz, 1H), 5.47 (dd, *J* = 5.9, 4.5 Hz, 1H), 5.43 (dd, *J* = 5.8, 5.0 Hz, 1H), 4.42-4.22 (m, 6H), 2.14 (s, 3H), 2.10 (s, 3H), 2.09 (s, 3H), 2.064 (s, 3H), 2.061 (s, 3H), 2.03 (s, 3H); ^13^C-NMR (100 MHz, CDCl_3_) δ 170.3, 170.2, 169.5, 169.4, 169.3, 169.2, 154.7, 146.9, 144.8, 143.8, 122.5, 87.9, 85.6, 80.3, 79.8, 74.2, 71.9, 70.2, 70.1, 63.0, 62.9, 20.7, 20.6, 20.4 (2C), 20.32, 20.31; HRMS (ES^+^) calcd for C_27_H_31_N_7_O_15_Na 716.1770 (M+Na)^+^ found 716.1758.

##### *N*1-(β-D-ribofuranosyl)-8-azidoinosine (8-N_3_-*N*1-ribosyl-IMP, 16)

*N*1-(2″,3″,5′-Tri-*O*-acetyl-β-D-ribofuranosyl)-2′,3′,5′-tri-*O*-acetyl-8-azidoinosine (**32**, 50 mg, 72 μmol) was taken up in MeOH (1.5 mL) in a pressure tube. The solution was cooled to 0 °C in an ice-water bath and NH_3_ (g) bubbled through the solution to saturation. The tube was sealed and the resulting solution stirred at rt. When complete by TLC, the solvents were removed by evaporation under reduced pressure and co-evaporation with MeOH 5 mL ×2). The residue was purified by column chromatography on silica gel eluting with DCM/MeOH (1:0 → 4:1 v/v + 0.5% pyridine) to afford the *title compound* (15 mg, 47%) as a clear glass; *R_f_* = 0.23 (DCM:MeOH 8:2 v/v); ^1^H-NMR (500 MHz, D_2_O) δ 8.50 (s, 1H, 2-H), 6.08 (d, *J* = 3.0 Hz, 1H), 5.80 (d, *J* = 6.2 Hz, 1H), 4.83 (dd, *J* = 6.2, 5.8, Hz, 1H), 4.38 (dd, *J* = 5.8, 3.6 Hz, 1H), 4.34 (dd, *J* = 5.2, 3.0 Hz, 1H), 4.22 (dd, *J* = 7.0, 5.2 Hz, 1H), 4.14-4.10 (m, 2H), 3.93 (dd, *J* = 12.9, 2.7 Hz, 1H), 3.82-3.77 (m, 2H), 3.75 (dd, *J* = 12.7, 4.6 Hz, 1H); ^13^C-NMR (125 MHz, D_2_O) δ 156.1, 147.3, 146.4, 144.4, 121.4, 90.4, 87.7, 85.6, 83.9, 74.7, 72.4, 70.3, 68.7, 61.6, 60.2; HRMS (ES^+^) calcd for C_15_H_20_N_7_O_9_ 442.1317 (M+H)^+^ found 442.1329.

##### *N*1-(2″,3″,5″-Tri-*O*-acetyl-β-D-ribofuranosyl)-2′,3′,5′-tri-*O*-acetyl-8-aminoinosine (33)

*N*1-(2″,3″,5″-Tri-*O*-acetyl-β-D-ribofuranosyl)-2′,3′,5′-tri-*O*-acetyl-8-azidoinosine (**32**, 65 mg, 87 μmol) and Pd/C (9 mg, 9 μmol) were taken up in EtOH (2 mL) and the solution degassed with argon before being placed under an atmosphere of H_2_ for 12 h. The catalyst was removed by filtration and the resulting solution purified by column chromatography on silica gel eluting with DCM/Acetone (1:0 → 0:1 v/v) to afford the title compound (51 mg, 81%) as a clear glass; *R_f_* = 0.48 (DCM:Acetone 1:1 v/v); ^1^H-NMR (400 MHz, CDCl_3_) δ 8.03 (s, 1H, 2-H), 6.29 (d, *J* = 4.3 Hz, 1H), 6.08 (d, *J* = 6.4 Hz, 1H), 5.84 (dd, *J* = 6.4, 6.2 Hz, 1H), 5.61 (br s, 2H, NH_2_), 5.53-5.49 (m, 2H), 5.45 (dd, *J* = 5.7, 5.5 Hz, 1H), 4.51 (dd, *J* = 12.1, 3.9 Hz, 1H), 4.44-4.28 (m, 5H), 2.15 (s, 3H), 2.12 (s, 3H), 2.11 (s, 3H), 2.10 (s, 3H), 2.09 (s, 3H), 2.06 (s, 3H); ^13^C-NMR (100 MHz, CDCl_3_) δ 170.3, 170.2, 169.50, 169.47, 169.46, 169.41, 154.9, 151.0, 145.9, 141.7, 121.4, 88.0, 84.8, 80.5, 79.9, 74.2, 70.9, 70.2, 70.0, 63.0, 62.9, 20.7, 20.6, 20.5, 20.39, 20.38, 20.3; HRMS (ES^+^) calcd for C_27_H_34_N_5_O_15_ 668.2046 (M+H)^+^ found 668.2021.

##### *N*1-(β-D-ribofuranosyl)-8-aminoinosine (8-NH_2_-*N*1-ribosyl-IMP, 17)

*N*1-(2″,3″,5″-Tri-*O*-acetyl-β-D-ribofuranosyl)-2′,3′,5′-tri-*O*-acetyl-8-aminoinosine (**33**, 50 mg, 69 μmol) was taken up in MeOH (2 mL) in a pressure tube. The solution was cooled to 0 °C in an ice-water bath and NH_3_ (g) bubbled through the solution to saturation. The tube was sealed and the resulting solution stirred at rt. When complete by TLC, the solvents were removed by evaporation under reduced pressure and co-evaporation with MeOH (5 mL ×2). The residue was purified by column chromatography on silica gel eluting with DCM/MeOH (1:0 → 7:3 v/v + 0.5% pyridine) to afford the *title compound* (24 mg, 83%) as a clear glass; *R_f_* = 0.38 (DCM:MeOH 7:3 v/v + 0.5% pyridine); ^1^H-NMR (400 MHz, CD_3_OD) δ 8.67 (s, 1H, 2-H), 6.34 (d, *J* = 3.3 Hz, 1H), 6.09 (d, *J* = 7.3 Hz, 1H), 4.81 (dd, *J* = 7.3, 5.6, Hz, 1H), 4.38-4.30 (m, 3H), 4.20-4.15 (m, 2H), 4.00 (dd, *J* = 12.3, 2.5 Hz, 1H), 3.92-3.84 (m, 3H); ^13^C-NMR (100 MHz, CD_3_OD) δ 156.6, 153.5, 148.2, 144.1, 120.6, 91.8, 88.9, 87.7, 86.3, 76.9, 72.9, 72.6, 70.6, 62.9, 61.8; HRMS (ES^+^) calcd for C_15_H_21_N_5_O_9_Na 438.1231 (M+Na)^+^ found 438.1246.

#### Total synthesis of *N*9-(4-hydroxybutyl)-*N*1-Inosine analogues (18-21)

*N*1-(2′,3′,5′-Tri-*O*-acetyl-β-D-ribofuranosyl)**-** *N*9-(4-(*tert*-butyldiphenylsilyl)oxybutyl)-8-bromohypoxanthine (**34**) was prepared as described previously.^36^

##### *N*1-(2′,3′,5′-Tri-*O*-acetyl-β-D-ribofuranosyl)-*N*9-(4-(*tert*-butyldiphenylsilyl)oxybutyl)-hypoxanthine (35)

*N*1-(2′,3′,5′-Tri-*O*-acetyl-β-D-ribofuranosyl)-*N*9-(4-(*tert*-butyldiphenylsilyl)oxybutyl)-8-bromohypoxanthine (**34**, 140mg, 0.179 mmol), NaHCO_3_ (75 mg, 0.895 mmol) and Pd/C (2 mg, 2 μmol) were taken up in EtOH (3 mL) and the solution degassed with argon before being placed under an atmosphere of H_2_ for 12 h. The catalyst was removed by filtration and the resulting solution purified by column chromatography on silica gel eluting with PE/EtOAc (1:0 → 0:1 v/v) to afford the *title compound* (95 mg, 77%) as a clear glass; *R_f_* = 0.60 (PE:EtOAc 1:3 v/v); ^1^H-NMR (400 MHz, CDCl_3_) δ 8.15 (s, 1H), 7.68 (s, 1H), 7.63-7.61 (m, 4H), 7.42-7.33 (m, 6H)(10H, TBDPS), 6.39 (d, *J* = 4.7 Hz, 1H, H-1′), 5.51-5.45 (m, 2H, H-2′, H-3′), 4.44-4.36 (m, 3H, H-4′, 2 × H-5′), 4.13 (t, *J* = 7.2 Hz, 2H, CH_2_), 3.69 (t, *J* = 6.0 Hz, 2H, CH_2_), 2.14 (s, 3H), 2.11 (s, 3H), 2.06 (s, 3H)(3 × CH_3_), 2.02-1.93 (m, 2H, CH_2_), 1.64-1.52 (m, 2H, CH_2_), 1.03 (s, 9H, TBDPS); ^13^C-NMR (100 MHz, CDCl_3_) δ 170.2, 169.53, 169.51, 156.0, 147.4, 143.8, 140.0, 135.5 (4C), 133.6 (2C), 129.6 (2C), 127.6 (4C), 123.9, 87.3, 80.1, 74.2, 70.2, 63.0, 62.9, 43.9, 29.3, 26.83, 26.80 (3C), 20.7, 20.42, 20.37, 19.1; HRMS (ES^+^) calcd for C_36_H_44_N_4_O_9_SiNa 727.2770 (M+H)^+^ found 727.2741.

##### *N*1-(2′,3′,5′-Tri-*O*-acetyl-β-D-ribofuranosyl)-*N*9-(4-hydroxybutyl)-hypoxanthine (39)

To TBAF (128 mg, 0.404 mmol) in DMF (0.5 mL) was added AcOH (24 μL, 0.425 mmol). After 30 min, the solution was cooled to 0 °C and a solution of *N*1-(2′,3′,5′-Tri-*O*-acetyl-β-D-ribofuranosyl)-*N*9-(4-(*tert*-butyldiphenylsilyl)oxybutyl)-hypoxanthine (**35**, 95 mg, 0.135 mmol) in DMF (1.0 mL) added dropwise. After 6h stirring at rt, NH_4_Cl (satd. aq.) was added and the solution extracted with DCM × 3. The combined organic layers were dried over Na_2_SO_4_ and purified by column chromatography on silica gel eluting with DCM/MeOH (1:0 → 4:1 v/v) to afford the *title compound* (29 mg, 46%) as a clear glass; *R_f_* = 0.41 (DCM:MeOH 9:1 v/v); ^1^H-NMR (400 MHz, CDCl_3_) δ 8.17 (s, 1H), 7.76 (s, 1H), 6.36 (d, *J* = 4.6 Hz, 1H, H-1′), 5.49 (dd, *J* = 5.8, 4.6 Hz, 1H, H-2′), 5.46 (dd, *J* = 5.8, 5.1 Hz, 1H, H-3′), 4.43-4.35 (m, 3H, H-4′, 2 × H-5′), 4.21 (t, *J* = 7.2 Hz, 2H, CH_2_), 3.69 (t, *J* = 6.1 Hz, 2H, CH_2_), 2.16 (s, 3H), 2.09 (s, 3H), 2.07 (s, 3H)(3 × CH_3_), 2.02-1.91 (m, 2H, CH_2_) and 1.63-1.54 (m, 2H, CH_2_); ^13^C-NMR (125 MHz, CDCl_3_) δ 170.4, 169.70, 169.66, 156.0, 147.5, 144.0, 140.3, 123.9, 87.6, 80.1, 74.2, 70.2, 63.0, 61.9, 44.0, 29.2, 27.1, 20.9, 20.52, 20.49; HRMS (ES^+^) calcd for C_20_H_27_N_4_O_9_ 467.1773 (M+H)^+^ found 467.1787.

##### *N*1-(β-D-Ribofuranosyl)-*N*9-(4-hydroxybutyl)-hypoxanthine (*N*9-hydroxybutyl-*N*1-Inosine, 19)

*N*1-(2′,3′,5′-Tri-*O*-acetyl-β-D-ribofuranosyl)-*N*9-(4-hydroxybutyl)-hypoxanthine (**39**, 25 mg, 54 μmol) was taken up in MeOH (2.0 mL) in a pressure tube. The solution was cooled to 0 °C in an ice-water bath and NH_3_ (g) bubbled through the solution to saturation. The tube was sealed and the resulting solution stirred at rt. When complete by TLC, the solvents were removed by evaporation under reduced pressure and co-evaporation with MeOH (5 mL ×2). The residue was purified by column chromatography on silica gel eluting with DCM/MeOH (1:0 → 3:1 v/v) to afford the *title compound* (8.7 mg, 48%) as a clear glass; *R_f_* = 0.19 (DCM:MeOH 4:1 v/v); ^1^H-NMR (500 MHz, D_2_O) δ 8.48 (s, 1H), 7.96 (s, 1H), 6.04 (d, *J* = 3.3 Hz, 1H, H-1′), 4.28 (dd, *J* = 5.2, 3.3 Hz, 1H, H-2′), 4.17 (dd, *J* = 6.5, 5.2 Hz, 1H, H-3′), 4.11 (t, *J* = 7.1 Hz, 2H, CH_2_), 4.06 (ddd, *J* = 6.5, 4.1, 2.7 Hz, 1H, H-4′), 3.86 (dd, *J* = 12.9, 2.7 Hz, 1H, H-5′_a_), 3.72 (dd, *J* = 12.9, 2.7 Hz, 1H, H-5′_b_), 3.43 (t, *J* = 6.5 Hz, 2H, CH_2_), 1.76 (dt, *J* = 7.6, 7.1 Hz, 2H, CH_2_) and 1.37 (dt, *J* = 7.6, 6.5 Hz, 2H, CH_2_); ^13^C-NMR (125 MHz, D_2_O) δ 157.5, 147.7, 145.0, 142.5, 122.6, 90.1, 83.9, 74.7, 68.8, 60.9, 60.2, 44.0, 28.3, 25.9; HRMS (ES^+^) calcd for C_14_H_20_N_4_O_6_Na 363.1275 (M+Na)^+^ found 363.1276.

##### *N*1-(2′,3′,5′-Tri-*O*-acetyl-β-D-ribofuranosyl)-*N*9-(4-hydroxybutyl)-8-bromohypoxanthine (38)

To TBAF (121 mg, 0.384 mmol) in DMF (0.5 mL) was added AcOH (23 μL, 0.402 mmol). After 30 min, the solution was cooled to 0 °C and a solution of *N*1-(2′,3′,5′-Tri-*O*-acetyl-β-D-ribofuranosyl)**-** *N*9-(4-(*tert*-butyldiphenylsilyl)oxybutyl)-8-bromohypoxanthine (**34**, 100 mg, 0.128 mmol) in DMF (1.0 mL) added dropwise. After 6h stirring at rt, NH_4_Cl (satd. aq.) was added and the solution extracted with DCM × 3. The combined organic layers were dried over Na_2_SO_4_ and purified by column chromatography on silica gel eluting with DCM/MeOH (1:0 → 4:1 v/v) to afford the *title compound* (41 mg, 59%) as a clear glass; *R_f_* = 0.10 (PE:EtOAc 1:3 v/v); ^1^H-NMR (400 MHz, CDCl_3_) δ 8.16 (s, 1H), 6.35 (d, *J* = 4.6 Hz, 1H, H-1′), 5.46 (dd, *J* = 4.6, 2.4 Hz, 1H, H-2′), 5.43 (dd, *J* = 5.8, 2.4 Hz, 1H, H-3′), 4.43-4.34 (m, 3H, H-4′, 2 × H-5′), 4.21 (t, *J* = 7.2 Hz, 2H, CH_2_), 3.69 (t, *J* = 6.2 Hz, 2H, CH_2_), 2.15 (s, 3H), 2.10 (s, 3H), 2.07 (s, 3H)(3 × CH_3_), 1.96-1.88 (m, 2H, CH_2_) and 1.62-1.55 (m, 2H, CH_2_); ^13^C-NMR (100 MHz, CDCl_3_) δ 170.2, 169.6, 169.5, 154.7, 148.7, 144.2, 126.1, 124.0, 87.4, 80.1, 74.1, 70.1, 62.9, 62.0, 44.6, 29.1, 26.2, 20.7, 20.41, 20.35; HRMS (ES^+^) calcd for C_20_H_25_N_4_O_9_Na^79^Br 567.0697 (M+Na)^+^ found 567.0688; calcd for C_20_H_25_N_4_O_9_Na^81^Br 569.0677 (M+Na)^+^ found 569.0656.

##### *N*1-(β-D-Ribofuranosyl)-*N*9-(4-hydroxybutyl)-8-bromohypoxanthine (8-Br-*N*9-hydroxybutyl-*N*1-Inosine, 18)

*N*1-(2′,3′,5′-Tri-*O*-acetyl-β-D-ribofuranosyl)-*N*9-(4-hydroxybutyl)-8-bromohypoxanthine (**38**, 40 mg, 73 μmol) was taken up in MeOH (1.5 mL) in a pressure tube. The solution was cooled to 0 °C in an ice-water bath and NH_3_ (g) bubbled through the solution to saturation. The tube was sealed and the resulting solution stirred at rt. When complete by TLC, the solvents were removed by evaporation under reduced pressure and co-evaporation with MeOH (5 mL ×2). The residue was purified by column chromatography on silica gel eluting with DCM/MeOH (1:0 → 3:1 v/v) to afford the *title compound* (9 mg, 29%) as a clear glass; *R_f_* = 0.12 (DCM:MeOH 9:1 v/v); ^1^H-NMR (400 MHz, CD_3_OD) δ 8.86 (s, 1H), 6.26 (d, *J* = 3.0 Hz, 1H, H-1′), 4.36-4.31 (m, 4H, H-2′, H-3′, CH_2_), 4.17 (ddd, *J* = 5.4, 2.9, 2.6 Hz, 1H, H-4′), 4.01 (dd, *J* = 12.3, 2.6 Hz, 1H, H-5′_a_), 3.87 (dd, *J* = 12.3, 2.9 Hz, 1H, H-5′_b_), 3.65 (t, *J* = 6.3 Hz, 2H, CH_2_), 1.98 (dt, *J* = 7.5, 7.1 Hz, 2H, CH_2_) and 1.62 (dt, *J* = 7.5, 6.3 Hz, 2H, CH_2_); ^13^C-NMR (100 MHz, CD_3_OD) δ 157.0, 150.6, 147.2, 127.7, 124.6, 91.5, 86.3, 76.9, 70.6, 62.2, 61.7, 45.8, 30.5, 27.2; HRMS (ES^+^) calcd for C_14_H_19_N_4_O_6_Na^79^Br 441.0380 (M+Na)^+^ found 441.0395; calcd for C_14_H_19_N_4_O_6_Na^81^Br 443.0360 (M+Na)^+^ found 443.0382.

##### *N*1-(2′,3′,5′-Tri-*O*-acetyl-β-D-ribofuranosyl)-*N*9-(4-(*tert*-butyldiphenylsilyl)oxybutyl)-8-azidohypoxanthine (36)

To *N*1-(2′,3′,5′-tri-*O*-acetyl-β-D-ribofuranosyl)**-** *N*9-(4-(*tert*-butyldiphenylsilyl)oxybutyl)-8-bromohypoxanthine (**34**, 400 mg, 0.510 mmol) in DMF (4.0 mL) was added NaN_3_ (106 mg, 1.633 mmol) and the resulting solution stirred at 70 °C in the dark for 72h. The solution was evaporated to dryness and the residue taken up in DCM, washed with H_2_O, dried over Na_2_SO_4_ and purified by column chromatography on silica gel eluting with PE/EtOAc (1:0 → 0:1 v/v) to afford the *title compound* (321 mg, 84%) as a clear glass; *R_f_* = 0.73 (PE:EtOAc 1:3 v/v) – note there is no change in retention time compared to the starting material; ^1^H-NMR (400 MHz, CDCl_3_) δ 8.09 (s, 1H), 7.64-7.62 (m, 4H), 7.42-7.34 (m, 6H)(10H, TBDPS), 6.37 (d, *J* = 4.4 Hz, 1H, H-1′), 5.50-5.45 (m, 2H, H-2′, H-3′), 4.44-4.36 (m, 3H, H-4′, 2 × H-5′), 3.97 (t, *J* = 7.2 Hz, 2H, CH_2_), 3.68 (t, *J* = 6.1 Hz, 2H, CH_2_), 2.13 (s, 3H), 2.12 (s, 3H), 2.08 (s, 3H)(3 × CH_3_), 1.96-1.85 (m, 2H, CH_2_), 1.56-1.49 (m, 2H, CH_2_), 1.03 (s, 9H, TBDPS); ^13^C-NMR (100 MHz, CDCl_3_) δ 170.1, 169.53, 169.52, 154.9, 147.5, 145.0, 143.2, 135.5 (4C), 133.7 (2C), 129.6 (2C), 127.6 (4C), 121.7, 87.6, 80.2, 74.3, 70.3, 63.1, 62.8, 42.4, 29.2, 26.8 (3C), 25.9, 20.7, 20.43, 20.36, 19.1; HRMS (ES^+^) calcd for C_36_H_43_N_7_O_9_SiNa 768.2784 (M+H)^+^ found 768.2761.

##### *N*1-(2′,3′,5′-Tri-*O*-acetyl-β-D-ribofuranosyl)-*N*9-(4-hydroxybutyl)-8-azidohypoxanthine (40)

To TBAF (114 mg, 0.362 mmol) in DMF (0.5 mL) was added AcOH (22 μL, 0.380 mmol). After 30 min, the solution was cooled to 0 °C and a solution of *N*1-(2′,3′,5′-Tri-*O*-acetyl-β-D-ribofuranosyl)-*N*9-(4-(*tert*-butyldiphenylsilyl)oxybutyl)-8-azidohypoxanthine (**36**, 90 mg, 0.121 mmol) in DMF (1.0 mL) added dropwise. After 6h stirring at rt, NH_4_Cl (satd. aq.) was added and the solution extracted with DCM × 3. The combined organic layers were dried over Na_2_SO_4_ and purified by column chromatography on silica gel eluting with DCM/MeOH (1:0 → 4:1 v/v) to afford the *title compound* (30 mg, 49%) as a clear glass; *R_f_* = 0.41 (DCM:MeOH 9:1 v/v); ^1^H-NMR (400 MHz, CDCl_3_) δ 8.11 (s, 1H), 6.34 (d, *J* = 4.5 Hz, 1H, H-1′), 5.48 (dd, *J* = 5.9, 4.6 Hz, 1H, H-2′), 5.45 (dd, *J* = 5.9, 4.7 Hz, 1H, H-3′), 4.43-4.35 (m, 3H, H-4′, 2 × H-5′), 4.02 (t, *J* = 7.1 Hz, 2H, CH_2_), 3.67 (t, *J* = 6.3 Hz, 2H, CH_2_), 2.15 (s, 3H), 2.11 (s, 3H), 2.07 (s, 3H)(3 × CH_3_), 1.91-1.83 (m, 2H, CH_2_) and 1.58-1.51 (m, 2H, CH_2_); ^13^C-NMR (100 MHz, CDCl_3_) δ 170.2, 169.6, 169.5, 154.9, 147.5, 145.0, 143.4, 121.7, 87.7, 80.2, 74.2, 70.2, 63.1, 62.0, 42.4, 29.1, 26.0, 20.7, 20.42, 20.36; HRMS (ES^+^) calcd for C_20_H_26_N_7_O_9_ 508.1787 (M+H)^+^ found 508.1799.

##### *N*1-(β-D-Ribofuranosyl)-*N*9-(4-hydroxybutyl)-8-azidohypoxanthine (8-N_3_-*N*9-hydroxybutyl-*N*1-Inosine, 20)

*N*1-(2′,3′,5′-Tri-*O*-acetyl-β-D-ribofuranosyl)-*N*9-(4-hydroxybutyl)-8-azidohypoxanthine (**40**, 30 mg, 59 μmol) was taken up in MeOH (1.5 mL) in a pressure tube. The solution was cooled to 0 °C in an ice-water bath and NH_3_ (g) bubbled through the solution to saturation. The tube was sealed and the resulting solution stirred at rt. When complete by TLC, the solvents were removed by evaporation under reduced pressure and co-evaporation with MeOH (5 mL ×2). The residue was purified by column chromatography on silica gel eluting with DCM/MeOH (1:0 → 4:1 v/v) to afford the *title compound* (10 mg, 44%) as a clear glass; *R_f_* = 0.11 (DCM:MeOH 9:1 v/v); ^1^H-NMR (400 MHz, CD_3_OD) δ 8.80 (s, 1H), 6.27 (d, *J* = 3.1 Hz, 1H, H-1′), 4.36-4.31 (m, 2H, H-2′, H-3′), 4.17 (ddd, *J* = 5.5, 2.9, 2.3 Hz, 1H, H-4′), 4.13 (t, *J* = 7.1 Hz, 2H, CH_2_), 4.01 (dd, *J* = 12.3, 2.6 Hz, 1H, H-5′_a_), 3.87 (dd, *J* = 12.3, 2.9 Hz, 1H, H-5′_b_), 3.63 (t, *J* = 6.4 Hz, 2H, CH_2_), 1.93 (dt, *J* = 7.3, 7.1 Hz, 2H, CH_2_) and 1.58 (dt, *J* = 7.3, 6.4 Hz, 2H, CH_2_); ^13^C-NMR (125 MHz, CD_3_OD) δ 157.3, 149.5, 146.7, 146.2, 122.4, 91.6, 86.3, 76.9, 70.6, 62.1, 61.7, 43.5, 30.5, 26.9; HRMS (ES^+^) calcd for C_14_H_19_N_7_O_6_Na 404.1289 (M+Na)^+^ found 404.1293.

##### *N*1-(2′,3′,5′-Tri-*O*-acetyl-β-D-ribofuranosyl)-*N*9-(4-(*tert*-butyldiphenylsilyl)oxybutyl)-8-aminohypoxanthine (37)

*N*1-(2′,3′,5′-Tri-*O*-acetyl-β-D-ribofuranosyl)-*N*9-(4-(*tert*-butyldiphenylsilyl)oxybutyl)-8-azidohypoxanthine (**36**, 110mg, 0.147 mmol) and Pd/C (9 mg, 9 μmol) were taken up in EtOH (3 mL) and the solution degassed with argon before being placed under an atmosphere of H_2_ for 12 h. The catalyst was removed by filtration and the resulting solution purified by column chromatography on silica gel eluting with DCM/Acetone (1:0 → 0:1 v/v) to afford the title compound (70 mg, 66%) as a clear glass; *R_f_* = 0.60 (DCM:Acetone 1:1 v/v); ^1^H-NMR (500 MHz, CDCl_3_) δ 8.03 (s, 1H), 7.65-7.63 (m, 4H), 7.44-7.36 (m, 6H)(10H, TBDPS), 6.40 (d, *J* = 4.7 Hz, 1H, H-1′), 5.50 (dd, 1H, *J* = 5.7, 4.7, H-2′), 5.47-5.45 (m, 1H, H-3′), 4.84 (brs, 2H, NH_2_), 4.44-4.36 (m, 3H, H-4′, 2 × H-5′), 3.98 (dt, *J* = 7.1, 1.5 Hz, 2H, CH_2_), 3.68 (m, 2H, CH_2_), 2.15 (s, 3H), 2.11 (s, 3H), 2.09 (s, 3H)(3 × CH_3_), 1.92-1.88 (m, 2H, CH_2_), 1.61-1.56 (m, 2H, CH_2_), 1.05 (s, 9H, TBDPS); ^13^C-NMR (125 MHz, CDCl_3_) δ 170.3, 169.63, 169.61, 155.1, 150.7, 146.7, 141.4, 135.5 (4C), 133.5 (2C), 129.8 (2C), 127.7 (4C), 120.6, 87.3, 80.0, 74.3, 70.2, 64.3, 63.3, 63.1, 41.7, 29.0, 26.9 (3C), 25.9, 20.8, 20.52, 20.49, 19.2; HRMS (ES^+^) calcd for C_36_H_45_N_5_O_9_SiNa 742.2879 (M+Na)^+^ found 742.2850.

##### *N*1-(2′,3′,5′-Tri-*O*-acetyl-β-D-ribofuranosyl)-*N*9-(4-hydroxybutyl)-8-aminohypoxanthine (41)

To TBAF (92 mg, 0.292 mmol) in DMF (0.5 mL) was added AcOH (18 μL, 0.306 mmol). After 30 min, the solution was cooled to 0 °C and a solution of *N*1-(2′,3′,5′-Tri-*O*-acetyl-β-D-ribofuranosyl)-*N*9-(4-(*tert*-butyldiphenylsilyl)oxybutyl)-8-aminohypoxanthine (**37**, 70 mg, 0.0972 mmol) in DMF (0.5 mL) added dropwise. After 6h stirring at rt, NH_4_Cl (satd. aq.) was added and the solution extracted with DCM × 3. The combined organic layers were dried over Na_2_SO_4_ and purified by column chromatography on silica gel eluting with DCM/MeOH (1:0 → 4:1 v/v) to afford the *title compound* (47 mg, 100%) as a clear glass; *R_f_* = 0.47 (DCM:MeOH 9:1 v/v); ^1^H-NMR (400 MHz, CDCl_3_) δ 7.99 (s, 1H), 6.24 (d, *J* = 4.0 Hz, 1H, H-1′), 6.16 (br s, 2H, NH_2_), 5.55 (dd, *J* = 5.8, 4.0 Hz, 1H, H-2′), 5.45 (dd, *J* = 5.8, 5.7 Hz, 1H, H-3′), 4.43-4.31 (m, 3H, H-4′, 2 × H-5′), 4.03 (t, *J* = 7.0 Hz, 2H, CH_2_), 3.69 (t, *J* = 5.8 Hz, 2H, CH_2_), 2.12 (s, 3H), 2.08 (s, 6H)(3 × CH_3_), 1.91-1.82 (m, 2H, CH_2_) and 1.58-1.51 (m, 2H, CH_2_); ^13^C-NMR (100 MHz, CDCl_3_) δ 170.4, 169.61, 169.59, 154.9, 152.4, 146.8, 141.1, 120.5, 88.4, 79.7, 74.1, 70.0, 62.9, 62.0, 41.5, 28.4, 26.1, 20.7, 20.41 (2C); HRMS (ES^+^) calcd for C_20_H_28_N_5_O_9_ 482.1882 (M+H)^+^ found 482.1891.

##### *N*1-(β-D-Ribofuranosyl)-*N*9-(4-hydroxybutyl)-8-aminohypoxanthine (8-NH_2_-*N*9-hydroxybutyl-*N*1-Inosine, 21)

*N*1-(2′,3′,5′-Tri-*O*-acetyl-β-D-ribofuranosyl)-*N*9-(4-hydroxybutyl)-8-aminohypoxanthine (**41**, 45 mg, 93 μmol) was taken up in MeOH (1.5 mL) in a pressure tube. The solution was cooled to 0 °C in an ice-water bath and NH_3_ (g) bubbled through the solution to saturation. The tube was sealed and the resulting solution stirred at rt. When complete by TLC, the solvents were removed by evaporation under reduced pressure and co-evaporation with MeOH (5 mL ×2). The residue was purified by column chromatography on silica gel eluting with DCM/MeOH (1:0 → 4:1 v/v + 0.5% pyridine) to afford the *title compound* (13 mg, 39%) as a clear glass; *R_f_* = 0.47 (DCM:MeOH 7:3 v/v); ^1^H-NMR (400 MHz, CD_3_OD) δ 8.63 (s, 1H), 6.25 (d, *J* = 3.4 Hz, 1H, H-1′), 4.37 (dd, *J* = 5.2, 3.4 Hz, 1H, H-2′), 4.33 (dd, *J* = 5.7, 5.2 Hz, 1H, H-3′), 4.16 (ddd, *J* = 5.7, 3.0, 2.5 Hz, 1H, H-4′), 4.12 (t, *J* = 7.2 Hz, 2H, CH_2_), 4.00 (dd, *J* = 12.3, 2.5 Hz, 1H, H-5′_a_), 3.86 (dd, *J* = 12.3, 3.0 Hz, 1H, H-5′_b_), 3.65 (t, *J* = 6.4 Hz, 2H, CH_2_), 1.90 (dt, *J* = 7.5, 7.2 Hz, 2H, CH_2_) and 1.61 (dt, *J* = 7.5, 6.4 Hz, 2H, CH_2_); ^13^C-NMR (125 MHz, CD_3_OD) δ 156.9, 154.1, 148.6, 143.8, 121.2, 91.7, 86.2, 76.8, 70.7, 62.4, 61.9, 42.4, 30.4, 26.5; HRMS (ES^+^) calcd for C_14_H_22_N_5_O_6_ 356.1565 (M+H)^+^ found 356.1561.

#### Total synthesis of *N*9-(4-hydroxybutyl)-*N*1-IMP analogues (22-25)

***N*1-[2′,3′-*O*-Isopropylidine-5′-*O*-(di-*tert-*butyl)-phosphoryl-β-D-ribofuranosyl]-*N*9-(4-hydroxybutyl)-8-bromohypoxanthine (44)** was prepared in 10 steps from 6-chloropurine as described previously.^36^

##### *N*1-(5′-*O*-Phosphoryl-β-D-ribofuranosyl)-*N*9-(4-hydroxybutyl)-8-bromohypoxanthine (22)

*N*1-[2′,3′-*O*-Isopropylidine-5′-*O*-(di-*tert-*butyl)-phosphoryl-β-D-ribofuranosyl]-*N*9-(4-hydroxybutyl)-8-bromohypoxanthine (**44**, 50 mg, 77 μmol) was stirred at 0 °C in TFA (50% aq., 2mL). Solvents were evaporated to dryness and the residue evaporated with MeOH (× 3) before purification by semipreparative HPLC (1.1 cm × 25 cm C18 column), eluting with acetonitrile/0.1 M TEAB (1:19 → 13:7 v/v) over 25 min. Fractions were analyzed by analytical HPLC and appropriate fractions collected and evaporated under vacuum to give the *title compound* (33 mg, 87%); UV (H_2_O, pH 7), λmax = 257 nm (ε = 14 700); ^1^H-NMR (500 MHz, D_2_O) δ 8.64 (s, 1H, H-2), 6.10 (d, *J* = 2.4 Hz, 1H, H-1′), 4.31-4.27 (m, 2H, H-2′ and H-3′), 4.21-4.18 (m, 1H, H-4′), 4.11-4.06 (m, 3H, H-5′_a_, CH_2_), 3.95-3.92 (m, 1H, H-5′_b_), 3.45 (t, *J* = 6.5 Hz, 2H, CH_2_), 1.72 (dt, *J* = 7.6, 7.3 Hz, 2H, CH_2_) and 1.41 (dt, *J* = 8.3, 6.5 Hz, 2H, CH_2_); ^13^C-NMR (125 MHz, D_2_O) δ 156.1, 148.7, 145.3, 128.2, 122.8, 89.1 (C-1′), 83.4 (d, *J* = 8.9 Hz, C-4′), 75.2 (C-2′), 68.8 (C-3′), 62.7 (d, *J* = 3.8 Hz, C-5′), 60.9, 44.8, 28.3, 25.3 (4 × CH_2_); ^31^P-NMR (202 MHz, D_2_O) δ 2.49. HRMS (ES^−^) calcd for C_14_H_19_N_4_O_9_P^79^Br 497.0079 (M−H)^−^ found 497.0073, calcd for C_14_H_19_N_4_O_9_P^81^Br 499.0058 (M−H)^−^ found 499.0072.

##### *N*1-(5′-*O*-Phosphoryl-β-D-ribofuranosyl)-*N*9-(4-hydroxybutyl)hypoxanthine (23)

*N*1-(5′-*O*-Phosphoryl-β-D-ribofuranosyl)-*N*9-(4-hydroxybutyl)-8-bromohypoxanthine (**22**, 38 mg, 77 μmol), NaHCO_3_ (20 mg, 0.23 mmol) and Pd/C (5 mg) were taken up in MilliQ-EtOH (2:1 v/v, 3 mL) and the solution degassed with argon before being placed under an atmosphere of H_2_ for 12 h. The catalyst was removed by filtration and the resulting solution purified by semipreparative HPLC (1.1 cm × 25 cm C18 column), eluting with acetonitrile/0.1 M TEAB (1:19 → 13:7 v/v) over 25 min. Fractions were analyzed by analytical HPLC and appropriate fractions collected and evaporated under vacuum to give the *title compound* (25 mg, 78%); UV (H_2_O, pH 7), λmax = 252 nm (ε = 10 300); ^1^H-NMR (500 MHz, D_2_O) δ 8.52 (s, 1H, H-2), 7.96 (s, 1H, H-8), 6.11 (d, *J* = 2.3 Hz, 1H, H-1′), 4.30-4.27 (m, 2H, H-2′ and H-3′), 4.22-4.19 (m, 1H, H-4′), 4.13-4.16 (m, 1H, H-5′_a_), 4.10 (t, *J* = 7.1 Hz, 2H, CH_2_), 4.02-3.98 (m, 1H, H-5′_b_), 3.44 (t, *J* = 6.5 Hz, 2H, CH_2_), 1.76 (dt, *J* = 7.6, 7.1 Hz, 2H, CH_2_) and 1.38 (dt, *J* = 8.1, 6.5 Hz, 2H, CH_2_); ^13^C-NMR (125 MHz, D_2_O) δ 157.4, 147.5, 144.7, 142.4, 122.3, 89.1 (C-1′), 82.9 (d, *J* = 8.8 Hz, C-4′), 75.1 (C-2′), 68.8 (C-3′), 63.4 (d, *J* = 4.1 Hz, C-5′), 60.9, 44.0, 28.3, 25.9 (4 × CH_2_); ^31^P-NMR (202 MHz, D_2_O) δ 0.49. HRMS (ES^−^) calcd for C_14_H_20_N_4_O_9_P 419.0973 (M−H)^−^ found 419.0958.

##### *N*1-(5′-*O*-Phosphoryl-β-D-ribofuranosyl)-*N*9-(4-hydroxybutyl)-8-azidohypoxanthine (24)

*N*1-(5′-*O-*phosphoryl-β-D-ribofuranosyl)-*N*9-(4-hydroxybutyl)-8-bromohypoxanthine (**22**, 20 mg, 40 μmol) was evaporated from DMF (2 × 2 mL), taken up in DMF (1 mL) and TMSN_3_ (53 μL, 401 μL) added. After 16 h at 70 °C in the dark, all solvents were evaporated and the residue purified by semi preparative HPLC (1.1 cm × 25 cm C18 column), eluting with acetonitrile/0.1 M TEAB (1:19 → 13:7 v/v) over 25 min. Fractions were analyzed by analytical HPLC and appropriate fractions collected and evaporated under vacuum to give the *title compound* (5.2 mg, 28%); UV (H_2_O, pH 7), λmax = 277 nm (ε = 15 600); ^1^H-NMR (500 MHz, D_2_O) δ 8.59 (s, 1H, H-2), 6.18 (d, *J* = 2.2 Hz, 1H, H-1′), 4.35-4.31 (m, 2H, H-2′ and H-3′), 4.27-4.24 (m, 1H, H-4′), 4.19-4.15 (m, 1H, H-5′_a_), 4.06-4.03 (m, 1H, H-5′_b_), 3.96 (t, *J* = 7.0 Hz, 2H, CH_2_), 3.50 (t, *J* = 6.6 Hz, 2H, CH_2_), 1.74 (dt, *J* = 7.5, 7.0 Hz, 2H, CH_2_) and 1.43 (dt, *J* = 8.1, 6.6 Hz, 2H, CH_2_); ^13^C-NMR (125 MHz, D_2_O) δ 156.2, 147.7, 146.6, 144.2, 120.5, 89.2 (C-1′), 83.0 (d, *J* = 8.9 Hz, C-4′), 75.2 (C-2′), 68.7 (C-3′), 63.2 (d, *J* = 4.6 Hz, C-5′), 60.9, 42.4, 28.3, 25.0 (4 × CH_2_); ^31^P-NMR (202 MHz, D_2_O) δ 0.97. HRMS (ES^−^) calcd for C_14_H_19_N_7_O_9_P 460.0987 (M−H)^−^ found 460.0969.

##### *N*1-(5′-*O*-Phosphoryl-β-D-ribofuranosyl)-*N*9-(4-hydroxybutyl)-8-aminohypoxanthine (25)

To *N*1-(5′-*O*-phosphoryl-β-D-ribofuranosyl)-*N*9-(4-hydroxybutyl)-8-azidohypoxanthine (**24**, 18 mg, 39 μmol) in TEAB (0.05 M, 5 mL) was added dithiothreitol (5 mg, 32 μmol). After 16h, the reaction was complete by HPLC (λ_max_ 277 → 262) and was purified by semipreparative HPLC (1.1 cm × 25 cm C18 column), eluting with acetonitrile/0.1 M TEAB (1:19 → 13:7 v/v) over 25 min. Fractions were analyzed by analytical HPLC and appropriate fractions collected and evaporated under vacuum to give the *title compound* (9.6 mg, 57%); UV (H_2_O, pH 7), λmax = 265 nm (ε = 25 400); H-NMR (500 MHz, D_2_O) δ 8.46 (s, 1H, H-2), 6.12 (d, *J* = 2.4 Hz, 1H, H-1′), 4.29-4.26 (m, 2H, H-2′ and H-3′), 4.18-4.16 (m, 1H, H-4′), 4.09-4.06 (m, 1H, H-5′_a_), 3.96-3.93 (m, 1H, H-5′_b_), 3.87 (t, *J* = 7.0 Hz, 2H, CH_2_), 3.42 (t, *J* = 6.4 Hz, 2H, CH_2_), 1.65 (dt, *J* = 7.5, 7.0 Hz, 2H, CH_2_) and 1.38 (dt, *J* = 8.1, 6.4 Hz, 2H, CH_2_); ^13^C-NMR (125 MHz, D_2_O) δ 155.8, 152.9, 147.3, 142.3, 119.3, 89.1 (C-1′), 83.1 (d, *J* = 8.8 Hz, C-4′), 75.2 (C-2′), 68.8 (C-3′), 62.8 (d, *J* = 4.3 Hz, C-5′), 61.1, 41.5, 28.4, 24.5 (4 × CH_2_); ^31^P-NMR (202 MHz, D_2_O) δ 2.03; HRMS (ES^−^) calcd for C_14_H_21_N_5_O_9_P 434.1082 (M−H)^−^ found 434.1063.

#### Total synthesis of L-*N*1-IMP analogues (26-27)

##### *N*1-(2′,3′,5′-Tri-*O*-acetyl-β-L-ribofuranosyl)-*N*9-*tert*-butyldimethylsilyloxymethyl-8-bromohypoxanthine (52)

To *N*9-*tert*-butyldimethylsilyloxymethyl-8-bromohypoxanthine (**48**, 430 mg, 1.197 mmol) in DCM (4.5 mL) was added DBU (537 μL, 3.590 mmol). After 30 min, 1,2,3,5-tetra-*O*-acetyl-β-L-ribofuranose (419 mg, 1.317 mmol) was added and the solution cooled to −78 °C. Trimethylsilyl trifluoromethanesulfonate (867 μL, 4.788 mmol) was added dropwise and the solution stirred for a further 45 min before warming to rt. After 2 h, NaHCO_3_ (satd aq) was added and the crude material extracted into DCM (×3). The combined organic fractions were dried (Na_2_SO_4_), and solvent was evaporated under reduced pressure. The residue was purified by column chromatography on silica gel eluting with PE/EtOAc (1:0 → 0:1 v/v) to afford the title compound (481 mg, 65%) as a colourless glass; *R_f_* = 0.48 (PE:EtOAc 1:3 v/v); ^1^H-NMR (400 MHz, CDCl_3_) δ 8.20 (s, 1H, H-2), 6.35 (d, *J* = 4.6 Hz, 1H, H-1′), 5.64 (d, *J* = 9.8 Hz, 1H), 5.61 (d, *J* = 9.8 Hz, 1H) (2H, CH_2_OTBDMS), 5.46-5.40 (m, 2H, H-2′, H-3′), 4.43-4.32 (m, 3H, H-4′, 2 × H-5′), 2.13 (s, 3H, AcetylCH_3_), 2.09 (s, 3H, AcetylCH_3_), 2.05 (s, 3H, AcetylCH_3_), 0.85 (s, 9H) 0.11 (s, 3H) and 0.10 (s, 3H) (15H, TBDMS); ^13^C-NMR (100 MHz, CDCl_3_) δ 170.2, 169.5 (2C), 154.7, 148.4, 144.5, 126.1, 123.9, 87.4, 80.1, 74.2, 70.1, 67.3, 62.9, 25.5 (3C), 20.7, 20.43, 20.37, 18.0, -5.26 and -5.27; HRMS (ES^+^) calcd for C_23_H_34_N_4_O_9_Si^79^Br 617.1273 (M+H)^+^ found 617.1298, calcd for C_23_H_34_N_4_O_9_Si^81^Br 619.1252 (M+H)^+^ found 619.1298.

##### *N*1-(2′,3′-*O*-Isopropylidene-β-L-ribofuranosyl)-*N*9-*tert*-butyldimethylsilyloxymethyl-8-bromohypoxanthine (54)

*N*1-(2′,3′,5′-Tri-*O*-acetyl-β-L-ribofuranosyl)-*N*9-*tert*-butyldimethylsilyloxymethyl-8-bromohypoxanthine (**52**, 470 mg, 0.761 mmol) was taken up in MeOH (5.0 mL) in a pressure tube. The solution was cooled to 0 °C in an ice-water bath and NH_3_ (g) bubbled through the solution to saturation. The tube was sealed and the resulting solution stirred at rt. When complete by TLC, the solvents were removed by evaporation under reduced pressure and the residue was purified by column chromatography on silica gel eluting with DCM/Acetone (1:0 → 0:1 v/v) to afford the deacetylated intermediate **53** (205 mg, 55%) as an amorphous white solid, *R_f_* = 0.34 (EtOAc), which was used directly in the next step.

To *N*1-(β-L-ribofuranosyl)-*N*9-*tert*-butyldimethylsilyloxymethyl-8-bromohypoxanthine (**53**, 170 mg, 0.346 mmol) in 2,2-dimethoxypropane-acetone (1:4 v/v, 10 mL) was added *para*-toluenesulfonic acid (66 mg, 0.346 mmol). After 1h, DCM and NaHCO_3_ (satd. aq.) were added and the aqueous phase extracted with DCM (×3). The combined organic extracts were evaporated to dryness, taken up in MeOH (5 mL) and treated with pre-washed DOWEX^®^ H^+^ resin for 30 min. The resin was removed by filtration and the filtrate purified by column chromatography on silica gel eluting with PE/EtOAc (1:0 → 0:1 v/v) to afford the *title compound* (103 mg, 56%) as a colorless glass; *R_f_* = 0.71 (EtOAc); ^1^H-NMR (500 MHz, CDCl_3_) δ 8.03 (s, 1H, 2-H), 5.71 (d, *J* = 2.9 Hz, 1H, H-1′), 5.66 (d, *J* = 9.9 Hz, 1H), 5.63 (d, *J* = 9.9 Hz, 1H) (2H, CH_2_OTBDMS), 5.31 (dd, *J* = 6.5, 2.9 Hz, 1H, H-2′), 5.15 (dd, *J* = 6.5, 3.6 Hz, 1H, H-3′), 4.36 (ddd, *J* = 6.0, 3.7, 3.6 Hz, 1H, H-4′), 3.93 (ddd, *J* = 12.0, 6.0, 3.1 Hz, 1H, H-5′_a_), 3.84 (ddd, *J* = 12.0, 8.2, 3.7 Hz, 1H, H-5′_b_), 3.31 (dd, *J* = 8.2, 3.1, 5′-OH), 1.59 (s, 3H, CCH_3_), 1.36 (s, 3H, CCH_3_), 0.87 (s, 9H), 0.12 (s, 3H), 0.11 (s, 3H) (15H, TBDMS); ^13^C-NMR (125 MHz, CDCl_3_) δ 155.3 (C-6), 148.9 (C-4), 146.9 (C-2), 126.6 (C-8), 124.8 (C-5), 114.3 (C(CH_3_)_2_), 97.2 (C-1′), 88.1 (C-4′), 83.5 (C-2′), 80.7 (C-3′), 67.5 (CH_2_OTBDMS), 62.9 (C-5′), 27.3 (CCH_3_), 25.5 (3C, SiC(CH_3_)_3_), 25.2 (CCH_3_), 18.1 (SiC(CH_3_)_3_), -5.2 (2C, Si(CH_3_)_2_); HRMS (ES^+^) calcd for C_20_H_32_N_4_O_6_^79^BrSi 531.1275 (M+H)^+^ found 531.1288, calcd for C_20_H_32_N_4_O_6_^79^BrSi 533.1254 (M+H)^+^ found 533.1249.

##### *N*1-[2′,3′-*O*-Isopropylidene-5′-*O*-(di-*tert*-butyl)-phosphoryl-β-L-ribofuranosyl]-*N*9-*tert*-butyldimethylsilyloxymethyl-8-bromohypoxanthine (55)

To *N*1-(2′,3′-*O*-isopropylidene-β-L-ribofuranosyl)-*N*9-*tert*-butyldimethylsilyloxymethyl-8-bromohypoxanthine (**54**, 100 mg, 0.188 mmol) in DCM (1.0 mL) was added 5-phenyl-1*H*-tetrazole (55 mg, 0.376 mmol) and the solution cooled to 0 °C. Di-*tert*-butyl *N,N-*diisopropylphosphoramidite (89 μL, 0.282 mmol) was added dropwise and the solution stirred at rt until phosphitylation was complete by TLC. After cooling to 0 °C, triethylamine (157 μL, 1.128 mmol) and H_2_O_2_ (30% in H_2_O, 48 μL, 0.470 mmol) were added and the solution stirred at rt until oxidation was complete. The reaction was diluted with DCM and washed with NaHCO_3_ (satd. aq.), dried over Na_2_SO_4_ and purified by column chromatography on silica gel eluting with PE/EtOAc (1:0 → 0:1 v/v + 0.5% pyridine in each solvent) to afford the *title compound* (82 mg, 60%) as a colourless glass; *R_f_* = 0.37 (PE:EtOAc 1:3 v/v); ^1^H-NMR (500 MHz, CDCl_3_) δ 8.10 (s, 1H, 2-H), 6.01 (d, *J* = 2.0 Hz, 1H, H-1′), 5.65 (d, *J* = 9.9 Hz, 1H), 5.63 (d, *J* = 9.9 Hz, 1H) (2H, CH_2_OTBDMS), 5.07 (dd, *J* = 6.4, 2.0 Hz, 1H, H-2′), 4.99 (dd, *J* = 6.4, 4.0 Hz, 1H, H-3′), 4.42 (ddd, *J* = 5.8, 4.1, 4.0 Hz, 1H, H-4′), 4.25 (ddd, *J* = 12.9, 6.5, 4.1 Hz, 1H, H-5′_a_), 4.18 (ddd, *J* = 12.9, 7.2, 5.8 Hz, 1H, H-5′_b_), 1.58 (s, 3H, CCH_3_), 1.47 (s, 9H, O^*t*^Bu), 1.46 (s, 9H, O^*t*^Bu), 1.34 (s, 3H, CCH_3_), 0.87 (s, 9H), 0.12 (s, 3H), 0.11 (s, 3H) (15H, TBDMS); ^13^C-NMR (125 MHz, CDCl_3_) δ 154.7, 148.7, 146.2, 126.1, 124.4, 114.4 (C(CH_3_)_2_), 94.1 (C-1′), 86.8 (d, *J* = 7.8 Hz, C-4′), 85.1 (C-2′), 82.7 (d, *J* = 7.1 Hz, 2C, 2 × POC(CH_3_)_3_), 81.3 (C-3′), 67.3 (CH_2_OTBDMS), 66.4 (d, *J* = 6.3 Hz, C-5′), 29.81 (d, *J* = 4.3 Hz, 3C, POC(CH_3_)_3_), 29.78 (d, *J* = 4.2 Hz, 3C, POC(CH_3_)_3_), 27.1 (CCH_3_), 25.5 (3C, SiC(CH_3_)_3_), 25.3 (CCH_3_), 18.0 (SiC(CH_3_)_3_), -5.2 (2C, Si(CH_3_)_2_); ^31^P-NMR (202 MHz, CDCl_3_) δ -10.10; HRMS (ES^+^) calcd for C_28_H_48_N_4_O_9_P^79^BrSiNa 745.2010 (M+Na)^+^ found 745.2040, calcd for C_28_H_48_N_4_O_9_P^81^BrSiNa 747.1989 (M+Na)^+^ found 747.2045.

##### *N*1-(5′-*O*-Phosphoryl-β-L-ribofuranosyl)-8-bromohypoxanthine (8-Br-L-*N*1-IMP, 26)

*N*1-[2′,3′-*O*Isopropylidene-5′-*O*-(di-*tert*-butyl)-phosphoryl-β-L-ribofuranosyl]-*N*9-*tert*-butyldimethylsilyloxymethyl-8-bromohypoxanthine (**55**, 55 mg, 76 μmol) was treated with TFA (50% aq., 4 mL) for 16h. All solvents were evaporated and the residue evaporated from MeOH × 3 before purification by semipreparative HPLC (1.1 cm × 25 cm C18 column), eluting with acetonitrile/0.1 M TEAB (1:19 → 13:7 v/v) over 25 min. Fractions were analyzed by analytical HPLC and appropriate fractions collected and evaporated under vacuum to give the *title compound* (14.4 mg, 45%); UV (H_2_O, pH 7), λmax = 261 nm (ε = 15,900); ^1^H-NMR (500 MHz, D_2_O) δ 8.49 (s, 1H, 2-H), 6.22 (d, *J* = 4.2 Hz, 1H, H-1′), 4.39 (dd, *J* = 5.3, 4.4 Hz, 1H, H-2′), 4.35 (dd, *J* = 5.3, 5,2 Hz, 1H, H-3′), 4.27-4.21 (m, 1H, H-4′), 4.07-4.03 (m, 1H, H-5′_a_) and 3.99-3.94 (m, 1H, H-5′_b_); ^13^C-NMR (125 MHz, D_2_O) δ 157.3, 156.4, 142.5, 136.3, 123.8, 88.4 (C-1′), 83.5 (d, *J* = 8.5 Hz, C-4′), 75.0 (C-2′), 69.4 (C-3′) and 63.1 (d, *J* = 4.4 Hz, C-5′); ^31^P-NMR (202 MHz, D_2_O) δ 2.82; HRMS (ES^−^) calcd for C_10_H_11_N_4_O_8_P^79^Br 424.9503 (M−H)^−^ found 424.9524, calcd for C_10_H_11_N_4_O_8_P^81^Br 426.9478 (M−H)^−^ found 426.9503.

##### *N*1-(5′-*O*-Phosphoryl-β-L-ribofuranosyl)-hypoxanthine (L-*N*1-IMP, 27)

*N*1-(5′-*O*-Phosphoryl-β-L-ribofuranosyl)-8-bromohypoxanthine (**26**, 5.3 mg, 14 μmol), NaHCO_3_ (12 mg, 0.14 mmol) and Pd/C (5 mg) were taken up in MilliQ-EtOH (2:1 v/v, 3 mL) and the solution degassed with argon before being placed under an atmosphere of H_2_ for 16 h. The catalyst was removed by filtration through celite and the resulting solution purified by semipreparative HPLC (1.1 cm × 25 cm C18 column), eluting with acetonitrile/0.1 M TEAB (1:19 → 13:7 v/v) over 25 min. Fractions were analyzed by analytical HPLC and appropriate fractions collected and evaporated under vacuum to give the *title compound* (2.1 mg, 49%); UV (H_2_O, pH 7), λmax = 251 nm (ε = 11,500); ^1^H-NMR (500 MHz, D_2_O) δ 8.65 (s, 1H), 8.10 (s, 1H), 6.22 (d, *J* = 3.8 Hz, 1H, H-1′), 4.39 (dd, *J* = 5.3, 3.8 Hz, 1H, H-2′), 4.36 (dd, *J* = 5.5, 5,3 Hz, 1H, H-3′), 4.27-4.23 (m, 1H, H-4′), 4.16-4.10 (m, 1H, H-5′_a_) and 4.04-4.00 (m, 1H, H-5′_b_); ^13^C-NMR (125 MHz, D_2_O) δ 156.3, 144.7, 141.8, 88.9 (C-1′), 83.2 (d, *J* = 8.4 Hz, C-4′), 75.0 (C-2′), 68.9 (C-3′) and 63.0 (d, *J* = 3.4 Hz, C-5′); ^31^P-NMR (202 MHz, D_2_O) δ 1.71. HRMS (ES^−^) calcd for C_10_H_12_N_4_O_8_P 347.0398 (M−H)^−^ found 347.0409.

#### Total synthesis of pyrophosphate bioisostere analogues (28-29)

##### *N*1-(2′,3′-*O*-Isopropylidene-5′-*O*-sulfonamide-β-D-ribofuranosyl)-*N*9-*tert*-butyldimethylsilyloxymethyl-8-chlorohypoxanthine (56)

*N*1-(2′,3′-*O*-Isopropylidene-β-D-ribofuranosyl)-*N*9-*tert*-butyldimethylsilyloxymethyl-8-bromohypoxanthine (**50**, 150mg, 0.28 mmol) was taken up in DCM (1.5 mL) and cooled to 0 °C. Triethylamine (47 μL, 0.34 mmol) was added and the solution stirred for 30 min before dropwise addition of sulfamoyl chloride in toluene (1.28 mL, 0.56 mmol, 0.44 M solution). After 16h at rt, MeOH (1 mL) was added and all solvent evaporated under reduced pressure. The residue was purified by column chromatography on silica gel eluting with PE/EtOAc (1:0 → 0:1 v/v) to afford the *title compound* (102 mg, 64%) as a colourless glass; *R_f_* = 0.68 (PE:EtOAc 1:3 v/v); ^1^H-NMR (400 MHz, CDCl_3_) δ 8.02 (s, 1H, 2-H), 5.77 (d, *J* = 1.4 Hz, 1H, H-1′), 5.67 (d, *J* = 9.8 Hz, 1H), 5.64 (d, *J* = 9.8 Hz, 1H) (2H, CH_2_OTBDMS), 5.40 (br s, 2H, NH_2_), 5.25 (dd, *J* = 6.5, 1.4 Hz, 1H, H-2′), 5.08-5.05 (m, 1H, H-3′), 4.51-4.44 (m, 3H, H-4′, 2 × H-5′), 1.56 (s, 3H, CCH_3_), 1.34 (s, 3H, CCH_3_), 0.88 (s, 9H), 0.14 (s, 3H), 0.12 (s, 3H) (15H, TBDMS); ^13^C-NMR (100 MHz, CDCl_3_) δ 155.2, 148.4, 147.0, 138.2, 123.0, 114.5 (C(CH_3_)_2_), 96.7 (C-1′), 86.7 (C-4′), 84.5 (C-2′), 81.8 (C-3′), 69.8 (C-5′), 66.8 (CH_2_OTBDMS), 27.0 (CCH_3_), 25.5 (3C, SiC(CH_3_)_3_), 25.1 (CCH_3_), 18.0 (SiC(CH_3_)_3_), -5.3 (2C, Si(CH_3_)_2_); HRMS (ES^+^) calcd for C_20_H_32_N_5_O_8_SSi^35^ClNa 588.1322 (M+Na)^+^ found 588.1317, calcd for C_20_H_32_N_5_O_8_Ssi^37^ClNa 590.1294 (M+Na)^+^ found 590.1347.

##### *N*1-(5′-*O*-sulfonamide-β-D-ribofuranosyl)-8-chlorohypoxanthine (8-Cl-*N*1-IMS, 28)

*N*1-(2′,3′-*O*Isopropylidene-5′-*O*-sulfonamide-β-D-ribofuranosyl)-*N*9-*tert*-butyldimethylsilyloxymethyl-8-chlorohypoxanthine (**56**, 15mg, 25 μmol) was cooled to 0 °C and H_2_O (1 mL) then TFA (1 mL) added. The solution was allowed to warm to rt and stirred for 3 h. All solvent was evaporated and the residue co-evaporated with MeOH (×3). The residue was purified by column chromatography on silica gel eluting with DCM/MeOH (1:0 → 4:1 v/v) to afford the *title compound* (7 mg, 69%) as a colourless glass. The final compound was further purified by semipreparative HPLC (1.1 cm × 25 cm C18 column), eluting with acetonitrile/0.1 M TEAB (1:19 → 13:7 v/v) over 25 min. Fractions were analyzed by analytical HPLC and appropriate fractions collected and evaporated under vacuum; *R_f_* = 0.59 (DCM:MeOH 9:1 v/v); ^1^H-NMR (500 MHz, D_2_O) δ 8.13 (s, 1H, H-2), 6.05 (d, *J* = 2.2 Hz, 1H, H-1′), 4.47-4.44 (m, 1H, H-2′), 4.36-4.33 (m, 2H, H-3′, H-4′) and 4.29-4.26 (m, 2H, 2 × H-5); ^13^C-NMR (125 MHz, D_2_O) δ 156.2, 150.1, 142.3, 142.1, 123.4, 90.2, 80.9, 74.2, 68.9 and 68.5; HRMS (ES^−^) calcd for C_10_H_11_^35^Cl N_5_O_7_S 380.0062 (M−H)^−^ found 380.0099, calcd for C_10_H_11_^37^Cl N_5_O_7_S 382.0033 (M−H)^−^ found 382.0076.

##### *N*1-(2′,3′-*O*-Isopropylidene-5′-*O*-sulfonamide-β-D-ribofuranosyl)-*N*9-*tert*-butyldimethylsilyloxymethylhypoxanthine (57)

*N*1-(2′,3′-*O*-Isopropylidene-5′-*O*-sulfonamide-β-D-ribofuranosyl)-*N*9-*tert*-butyldimethylsilyloxymethyl-8-chlorohypoxanthine (**56**, 15mg, 25 μmol) was taken up in EtOH (1 mL). Pd/C (<1 mg, 10 mol %) and NaHCO_3_ (11 mg, 0.125 mmol) were added and the flask evacuated and purged with Argon (×3) before placing under an atmosphere of H_2_. After stirring for 16h, the suspension was filtered through cotton wool to remove the catalyst and all solvent evaporated under reduced pressure. The residue was purified by column chromatography on silica gel eluting with DCM/MeOH (1:0 → 4:1 v/v) to afford the *title compound* (7 mg, 54%) as a colourless glass; *R_f_* = 0.32 (PE:EtOAc 1:3 v/v); ^1^H-NMR (400 MHz, CDCl_3_) δ 8.00 (s, 1H, 2-H), 7.93 (s, 1H, 8-H), 5.78 (d, *J* = 1.3 Hz, 1H, H-1′), 5.69 (d, *J* = 9.6 Hz, 1H), 5.66 (d, *J* = 9.6 Hz, 1H) (2H, CH_2_OTBDMS), 5.43 (br s, 2H, NH_2_), 5.29 (dd, *J* = 6.4, 1.3 Hz, 1H, H-2′), 5.10 (dd, *J* = 6.4, 2.8 Hz, 1H, H-3′), 4.53-4.46 (m, 3H, H-4′, 2 × H-5′), 1.57 (s, 3H, CCH_3_), 1.35 (s, 3H, CCH_3_), 0.88 (s, 9H), 0.12 (s, 3H), 0.11 (s, 3H) (15H, TBDMS); ^13^C-NMR (125 MHz, CDCl_3_) δ 156.4, 147.4, 146.8, 140.4, 124.2, 114.5 (C(CH_3_)_2_), 96.8 (C-1′), 86.8 (C-4′), 84.6 (C-2′), 81.9 (C-3′), 69.9 (C-5′), 67.7 (CH_2_OTBDMS), 27.0 (CCH_3_), 25.5 (3C, SiC(CH_3_)_3_), 25.2 (CCH_3_), 18.0 (SiC(CH_3_)_3_), -5.2 (2C, Si(CH_3_)_2_); HRMS (ES^+^) calcd for C_20_H_34_N_5_O_8_SSi 532.1892 (M+Na)^+^ found 532.1909.

##### *N*1-(5′-*O*-sulfonamide-β-D-ribofuranosyl)-hypoxanthine (*N*1-IMS, 29)

*N*1-(2′,3′-*O*-Isopropylidene-5′-*O*-sulfonamide-β-D-ribofuranosyl)-*N*9-*tert*-butyldimethylsilyloxymethylhypoxanthine (**57**, 5.5 mg, 10 μmol) was cooled to 0 °C and H_2_O (1 mL) then TFA (1 mL) added. The solution was allowed to warm to rt and stirred for 3 h. All solvent was evaporated and the residue co-evaporated with MeOH (×3). The residue was purified by column chromatography on silica gel eluting with DCM/MeOH (1:0 → 4:1 v/v) to afford the *title compound* (3 mg, 84%) as a colourless glass; ^1^H-NMR (500 MHz, D_2_O) δ 8.32 (s, 1H), 8.18 (br s, 1H), 6.06 (d, *J* = 3.3 Hz, 1H), 4.48 (dd, *J* = 11.4, 1.9 Hz, 1H), 4.36 (dd, *J* = 11.4, 3.3 Hz, 1H), 4.33 (dd, *J* = 4.6, 3.5 Hz, 1H) and 4.29-4.25 (m, 2H); ^13^C-NMR (125 MHz, D_2_O) δ 155.7, 151.2, 145.0, 141.9, 117.7, 90.4, 81.1, 74.2, 68.8 and 68.3; HRMS (ES^−^) calcd for C_10_H_13_N_5_O_7_SNa 370.0428 (M−H)^−^ found 370.0437.

### Enzymatic Assay for cADPR Hydrolysis

The inhibition of cADPR hydrolysis by various concentrations of inhibitor (0–1 mM) was determined by incubating 1 mM cADPR with 2 mg/mL of CD38 for 10 min at 20–24uC in 25 mM sodium acetate, pH 4.5. The reaction was stopped by the addition of 150 mM HCl. The precipitated protein was filtered, and the pH was neutralized with Tris base. After diluting the mixture 20-fold, the concentration of the unhydrolyzed cADPR present in the diluted reaction mixture was assayed by the fluorimetric cycling assay as previously described.^46^

### HPLC studies

HPLC studies were carried out as previously described.^36^ Briefly, the solution containing shCD38 was adjusted to the desired concentration using Tris-HCl buffer (20 mM, pH 8) and 50 μL was added to the inhibitor (0.05 μmole) in an Eppendorf tube at room temperature. At a given time point, a sample of 5 μL was removed and diluted with 95 μL MilliQ water. 10 μL Of this sample was injected directly into the analytical HPLC system (see General Experimental), eluting at 1 mL/min with an isocratic ion-pair buffer: 0.17% (m/v) cetrimide and 45% (v/v) phosphate buffer (pH 6.4) in MeOH.

## Supplementary Material

The following are available online at www.mdpi.com, Figure S1: Hydrolysis of 8-NH_2_-*N*1-IMP by high concentrations of shCD38; ^1^H, ^13^C and ^31^P spectral data and HPLC profiles for novel compounds.

Supplementary Information

## Figures and Tables

**Figure 3:**
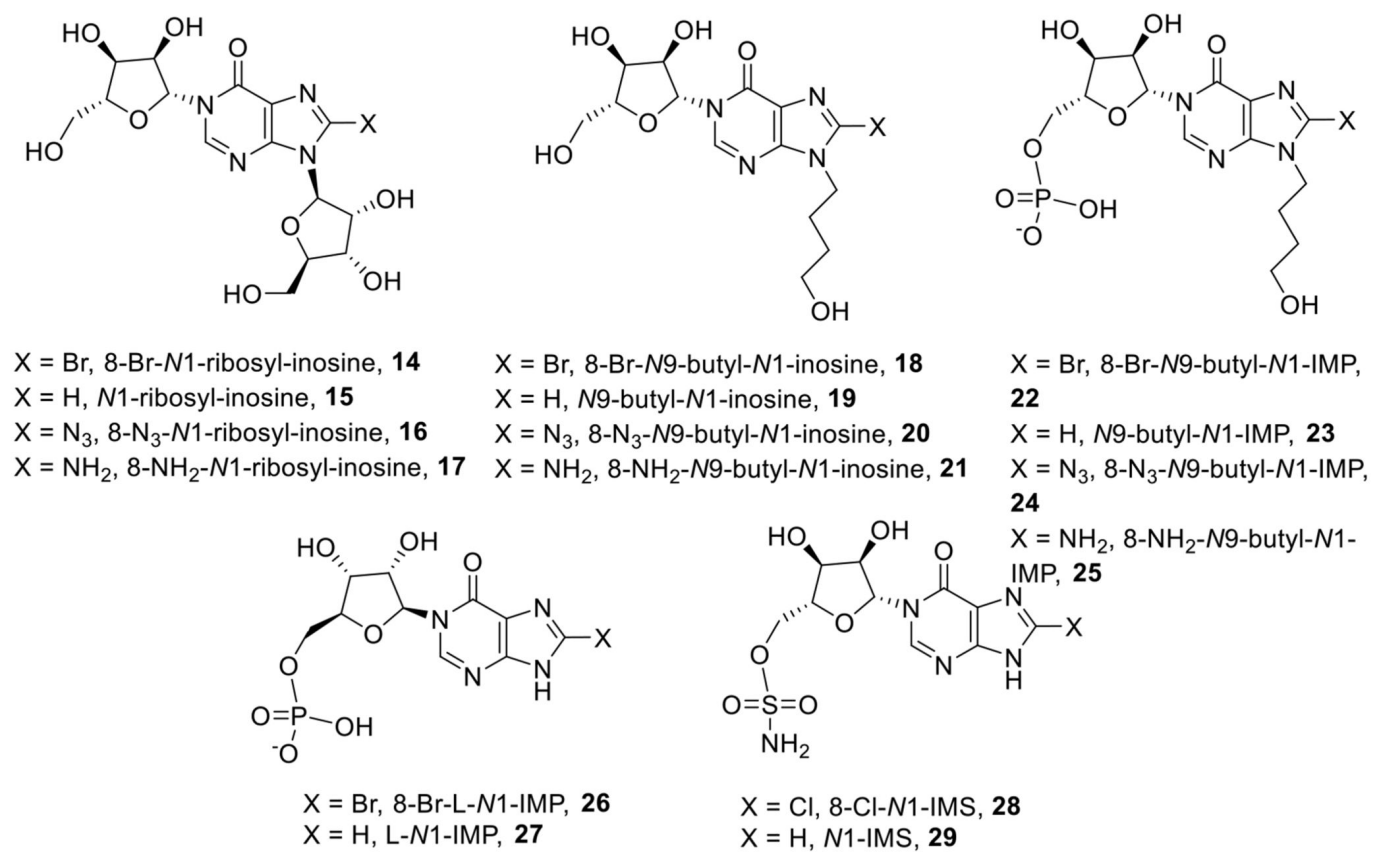
Synthetic analogues prepared in this study (IMP = Inosine 5′-monophosphate, IMS = Inosine 5′-monosulfamate)

**Table:** Summary of inhibitory activity (IC_50_, μM) of analogues * *n.d* = not determined. Blank cell = analogue not prepared. IC50 values >1000μM are approximate.

Compound	8-Br	8-H	8-N_3_	8-NH_2_	8-Cl
*N*1-ribosyl-inosine **14-17**	No activity	7500	No activity	7600	
*N*9-Hydroxybutyl-*N*1-inosine **18-21**	Insoluble	2300	9500	3300	
*N*9-Hydroxybutyl-*N*1-IMP **22-25** ^39^	*n.d*	757	201	332	
*N*1-IMP^1^ **9-11**	201 ± 41	14 ± 5	*n.d*	7.6 ± 0.2	
L-*N*1-IMP **26**, **27**	230 ±9	460			
*N*1-IMS **28**, **29**		No activity			No activity
