# Supplementary material for: Small Molecule CD38 Inhibitors: Synthesis of 8-Amino-N1-Inosine 5′-Monophosphate, Analogues and Early Structure-Activity Relationship
Source: Molecules. Author manuscript; Available in PMC 2022 Jan 4. (PMC8658804; doi:10.3390/molecules26237165)
Supplement: Supplementary Information [file EMS140042-supplement-Supplementary_Information.pdf]

Supplementary Information

Small Molecule CD38 Inhibitors: Synthesis of 8-Amino-*N*1-Inosine 5'-Monophosphate, Analogues and Early Structure-Activity Relationship

Joanna M. Watt[1,2], Richard Graeff[3] and Barry V. L. Potter[1]*

[1] Medicinal Chemistry & Drug Discovery, Department of Pharmacology, University of Oxford, Mansfield Road, Oxford, OX1 3QT, UK

[2] Wolfson Laboratory of Medicinal Chemistry, Department of Pharmacy and Pharmacology, University of Bath, Claverton Down, Bath, BA2 7AY, UK

[3] Department of Physiology, University of Hong Kong, Hong Kong, China

Contents

Figure S1: Hydrolysis of 8-NH_2-*N*1-IMP by high concentration of CD38 - HPLC data p2

^{1}H, ^{13}C-NMR and HPLC data p3-56

<u>**Figure S1: Hydrolysis of 8-NH$_2$-*N*1-IMP by high concentration of CD38 - HPLC data**</u>

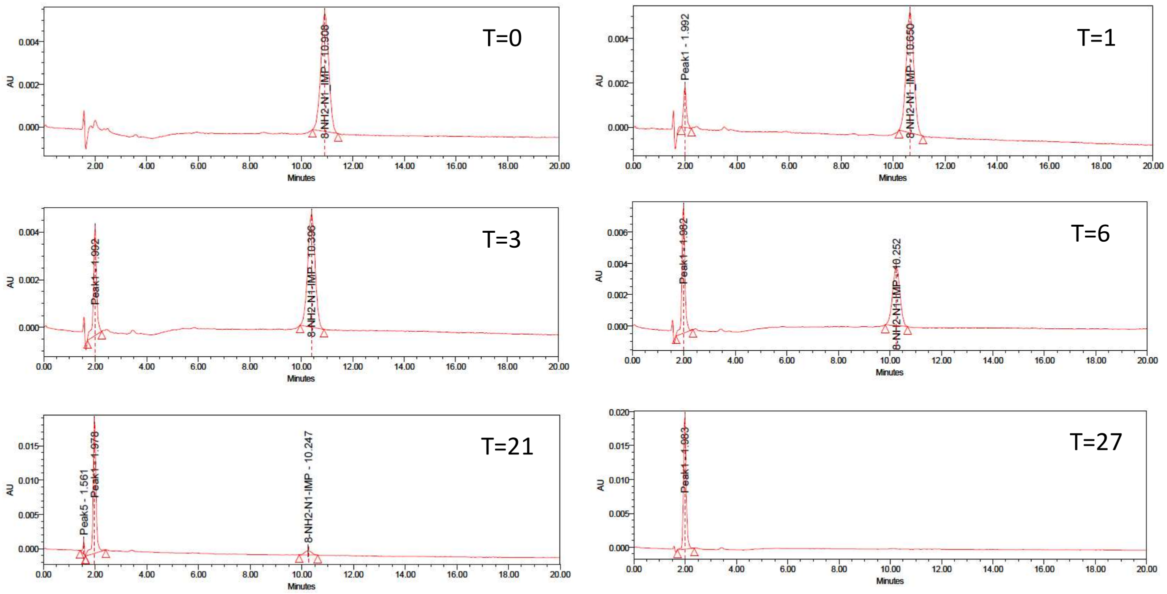

HPLC traces showing 8-NH$_2$-*N*1-IMP (**11**) is turned over by high concentrations of CD38. HPLC conditions: eluting at 1 mL/min with an isocratic ion-pair buffer: 0.17% (m/v) cetrimide and 45% (v/v) phosphate buffer (pH 6.4) in MeOH. Time (T) in hours from addition of CD38.

Synthesis of 8-NH$_2$-*N*1-IMP (11)

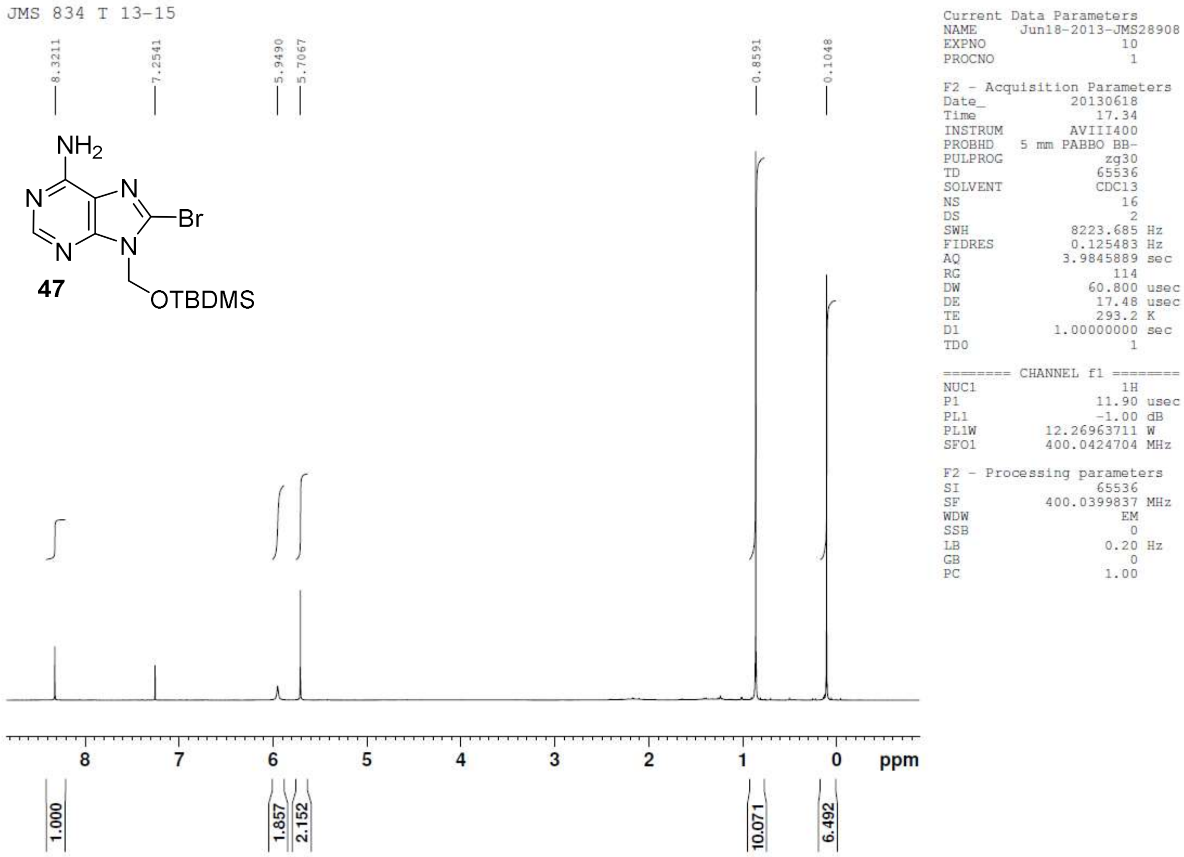

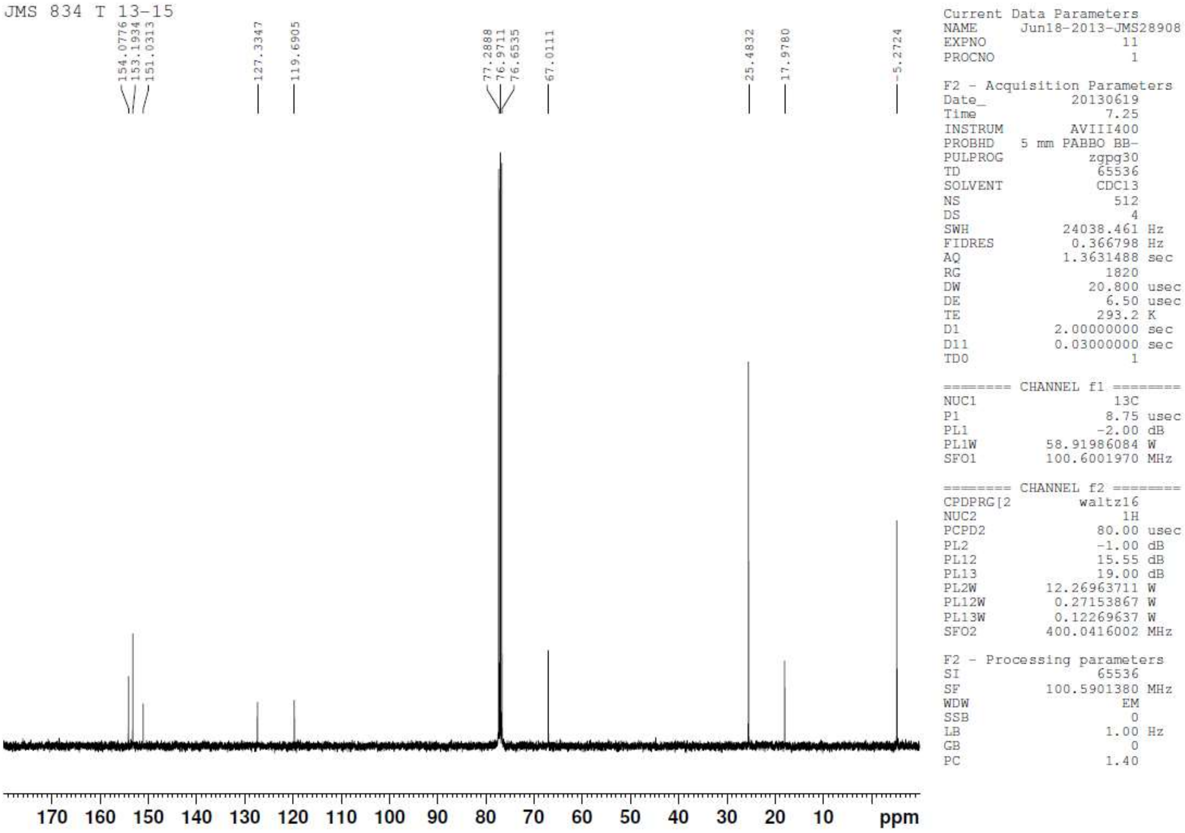

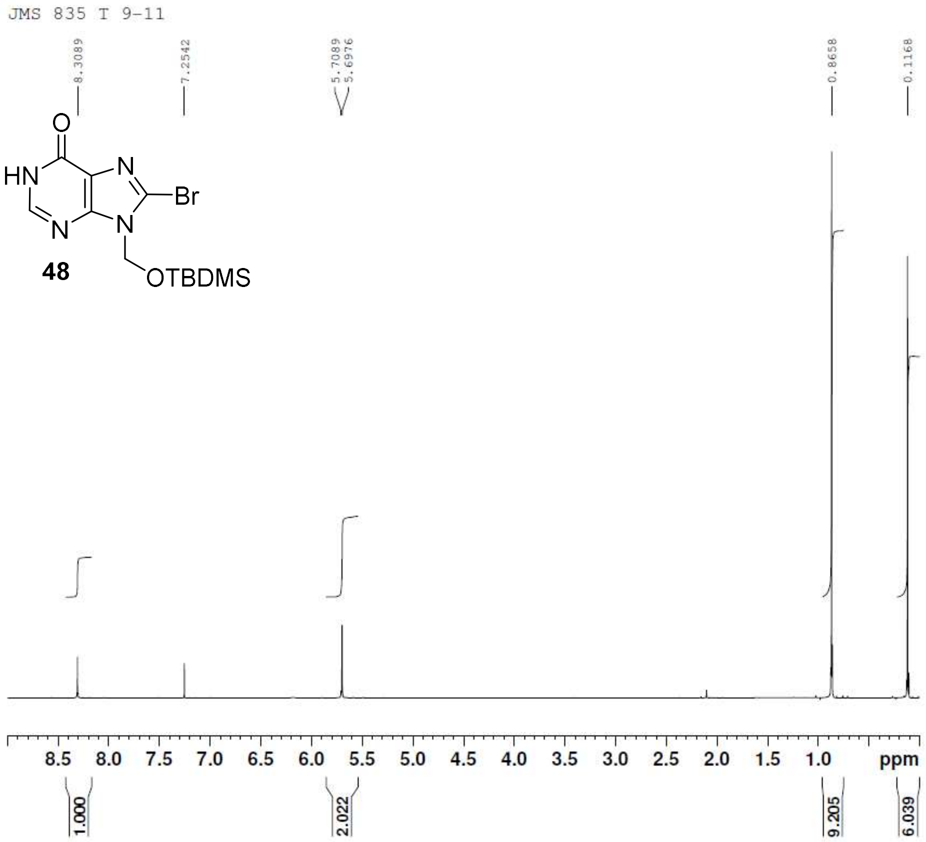


```
Current Data Parameters
NAME       Jun26-2013-JMS28987
EXPNO                   10
PROCNO                   1

F2 - Acquisition Parameters
Date_             20130626
Time                 10.30
INSTRUM           AVIII400
PROBHD   5 mm PABBO BB-
PULPROG               zg30
TD                   65536
SOLVENT              CDC13
NS                      16
DS                       2
SWH               8223.685 Hz
FIDRES            0.125483 Hz
AQ               3.9845889 sec
RG                    90.5
DW                  60.800 usec
DE                   17.48 usec
TE                   293.2 K
D1              1.00000000 sec
TD0                      1

======== CHANNEL f1 ========
NUC1                    1H
P1                   11.90 usec
PL1                  -1.00 dB
PL1W           12.26963711 W
SFO1           400.0424704 MHz

F2 - Processing parameters
SI                   65536
SF             400.0399837 MHz
WDW                     EM
SSB                      0
LB                    0.20 Hz
GB                       0
PC                    1.00
```

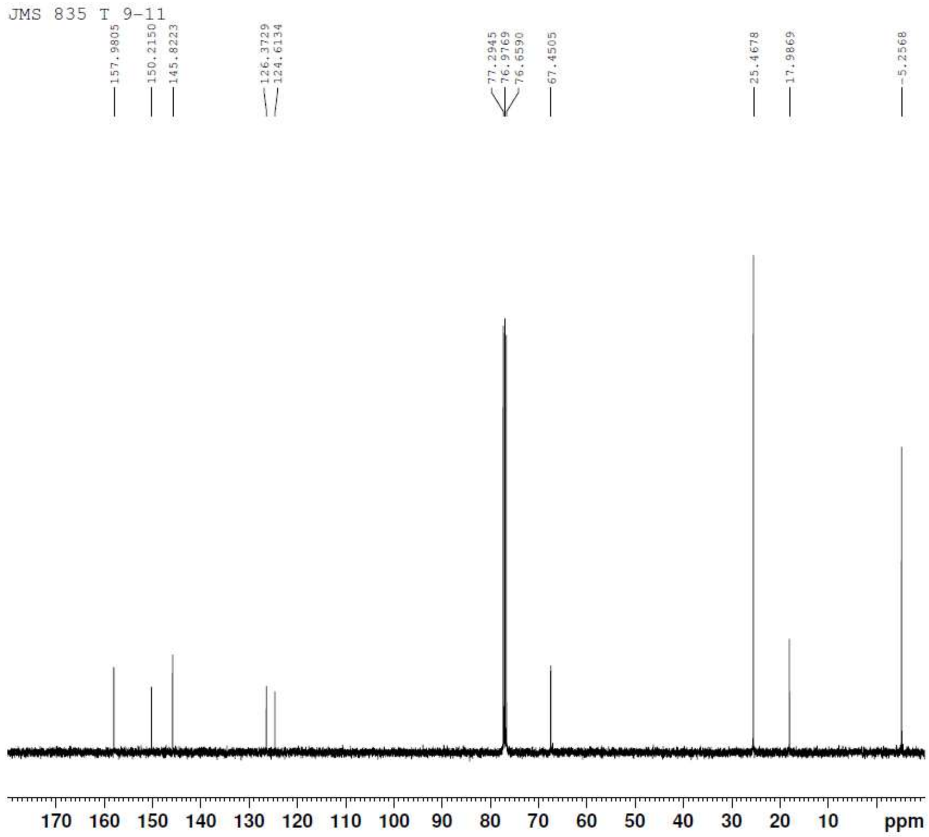


```
Current Data Parameters
NAME       Jun26-2013-JMS28987
EXPNO                   11
PROCNO                   1

F2 - Acquisition Parameters
Date_             20130626
Time                 10.39
INSTRUM           AVIII400
PROBHD   5 mm PABBO BB-
PULPROG             zgpg30
TD                   65536
SOLVENT              CDC13
NS                     256
DS                       4
SWH              24038.461 Hz
FIDRES            0.366798 Hz
AQ               1.3631488 sec
RG                    1820
DW                  20.800 usec
DE                    6.50 usec
TE                   293.2 K
D1              2.00000000 sec
D11             0.03000000 sec
TD0                      1

======== CHANNEL f1 ========
NUC1                   13C
P1                    8.75 usec
PL1                  -2.00 dB
PL1W           58.91986084 W
SFO1           100.6001970 MHz

======== CHANNEL f2 ========
CPDPRG[2           waltz16
NUC2                    1H
PCPD2                80.00 usec
PL2                  -1.00 dB
PL12                 15.55 dB
PL13                 19.00 dB
PL2W           12.26963711 W
PL12W           0.27153867 W
PL13W           0.12269637 W
SFO2           400.0416002 MHz

F2 - Processing parameters
SI                   65536
SF             100.5901380 MHz
WDW                     EM
SSB                      0
LB                    1.00 Hz
GB                       0
PC                    1.40
```

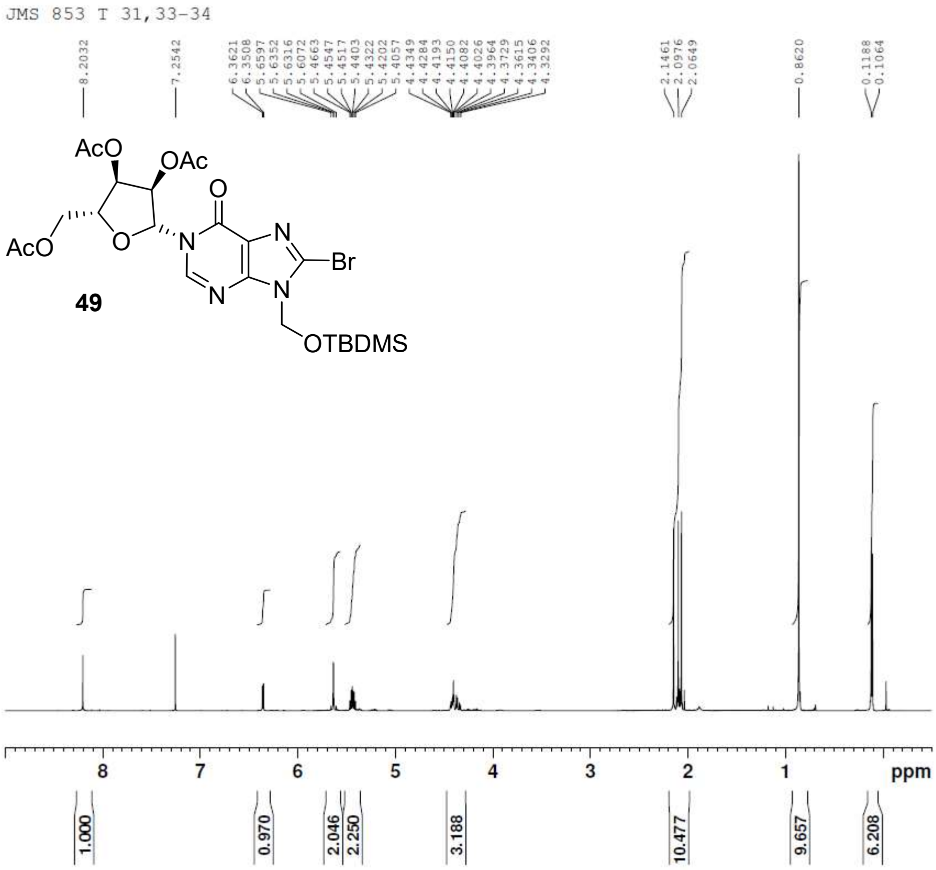

Current Data Parameters
NAME Aug01-2013-JMS29374
EXPNO 10
PROCNO 1

F2 - Acquisition Parameters
Date_ 20130801
Time 12.45
INSTRUM AVIII400
PROBHD 5 mm PABBO BB-
PULPROG zg30
TD 65536
SOLVENT CDCl3
NS 16
DS 2
SWH 8223.685 Hz
FIDRES 0.125483 Hz
AQ 3.9845889 sec
RG 64
DW 60.800 usec
DE 17.48 usec
TE 293.2 K
D1 1.00000000 sec
TD0 1

======== CHANNEL f1 ========
NUC1 1H
P1 11.90 usec
PL1 -1.00 dB
PL1W 12.26963711 W
SFO1 400.0424704 MHz

F2 - Processing parameters
SI 65536
SF 400.0399837 MHz
WDW EM
SSB 0
LB 0.20 Hz
GB 0
PC 1.00

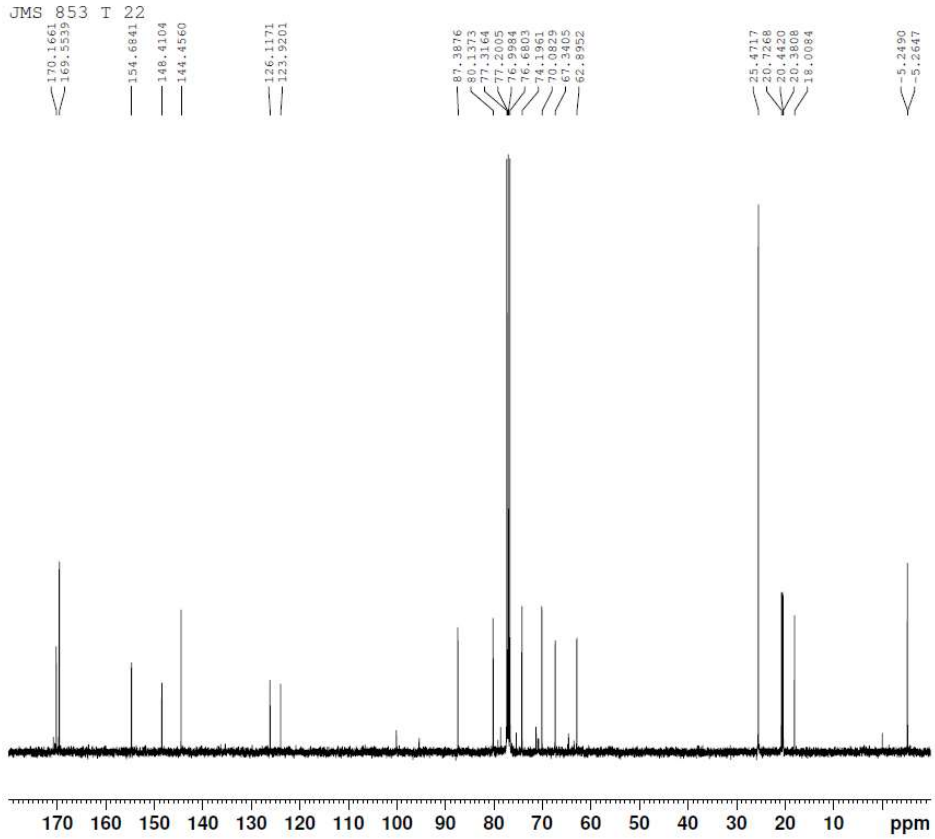

Current Data Parameters
NAME Jul31-2013-JMS29366
EXPNO 11
PROCNO 1

F2 - Acquisition Parameters
Date_ 20130801
Time 4.40
INSTRUM AVIII400
PROBHD 5 mm PABBO BB-
PULPROG zgpg30
TD 65536
SOLVENT CDCl3
NS 512
DS 4
SWH 24038.461 Hz
FIDRES 0.366798 Hz
AQ 1.3631488 sec
RG 1820
DW 20.800 usec
DE 6.50 usec
TE 293.2 K
D1 2.00000000 sec
D11 0.03000000 sec
TD0 1

======== CHANNEL f1 ========
NUC1 13C
P1 8.75 usec
PL1 -2.00 dB
PL1W 58.91986084 W
SFO1 100.6001970 MHz

======== CHANNEL f2 ========
CPDPRG[2 waltz16
NUC2 1H
PCPD2 80.00 usec
PL2 -1.00 dB
PL12 15.55 dB
PL13 19.00 dB
PL2W 12.26963711 W
PL12W 0.27153867 W
PL13W 0.12269637 W
SFO2 400.0416002 MHz

F2 - Processing parameters
SI 65536
SF 100.5901380 MHz
WDW EM
SSB 0
LB 1.00 Hz
GB 0
PC 1.40

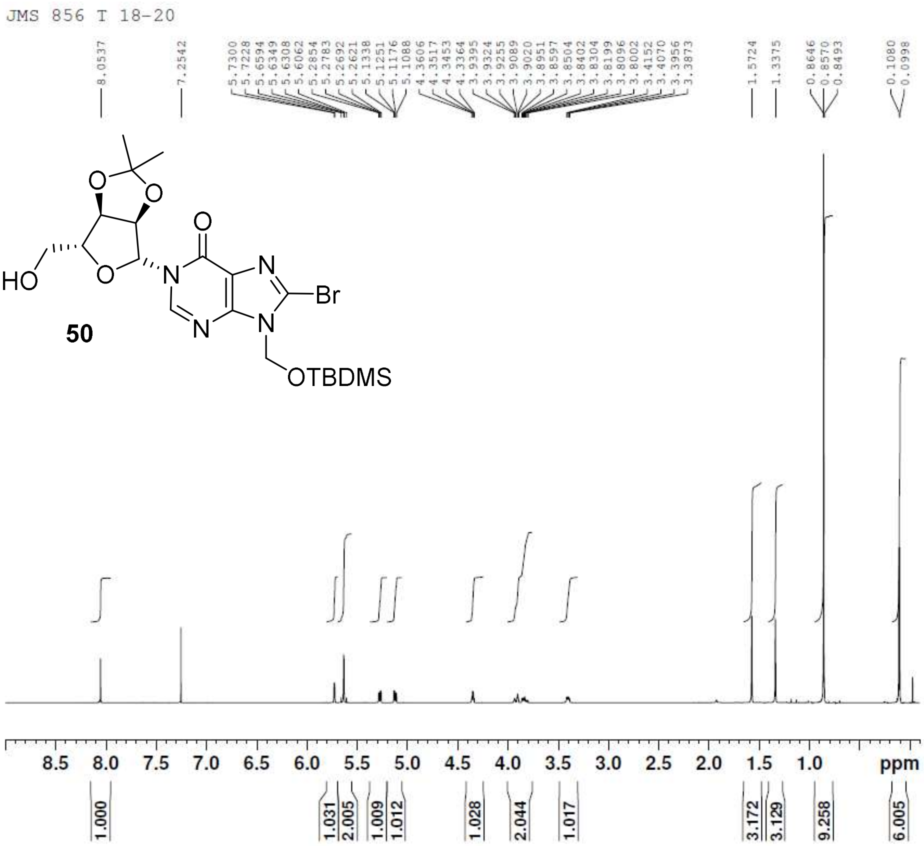


```
Current Data Parameters
NAME        Aug02-2013-JMS29398
EXPNO                        10
PROCNO                        1

F2 - Acquisition Parameters
Date_                  20130802
Time                      17.07
INSTRUM                AVIII400
PROBHD   5 mm PABBO BB-
PULPROG                    zg30
TD                        65536
SOLVENT                   CDCl3
NS                           16
DS                            2
SWH                 8223.685 Hz
FIDRES              0.125483 Hz
AQ                3.9845889 sec
RG                           64
DW                  60.800 usec
DE                   17.48 usec
TE                      293.2 K
D1               1.00000000 sec
TD0                           1

======== CHANNEL f1 ========
NUC1                         1H
P1                  11.90 usec
PL1                    -1.00 dB
PL1W            12.26963711 W
SFO1          400.0424704 MHz

F2 - Processing parameters
SI                        65536
SF            400.0399837 MHz
WDW                          EM
SSB                           0
LB                      0.20 Hz
GB                            0
PC                         1.00
```

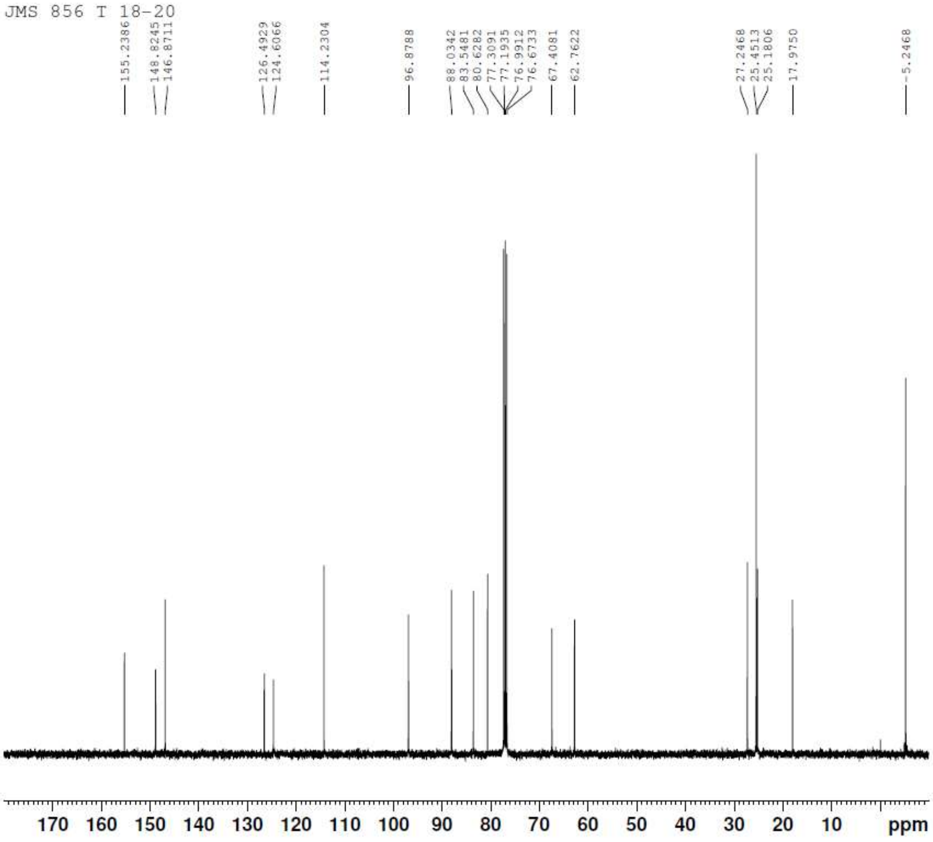


```
Current Data Parameters
NAME        Aug02-2013-JMS29398
EXPNO                        11
PROCNO                        1

F2 - Acquisition Parameters
Date_                  20130803
Time                       6.30
INSTRUM                AVIII400
PROBHD   5 mm PABBO BB-
PULPROG                  zgpg30
TD                        65536
SOLVENT                   CDCl3
NS                          512
DS                            4
SWH                24038.461 Hz
FIDRES              0.366798 Hz
AQ                1.3631488 sec
RG                         1820
DW                  20.800 usec
DE                    6.50 usec
TE                      293.2 K
D1               2.00000000 sec
D11              0.03000000 sec
TD0                           1

======== CHANNEL f1 ========
NUC1                        13C
P1                   8.75 usec
PL1                    -2.00 dB
PL1W            58.91986084 W
SFO1          100.6001970 MHz

======== CHANNEL f2 ========
CPDPRG[2                waltz16
NUC2                         1H
PCPD2               80.00 usec
PL2                    -1.00 dB
PL12                   15.55 dB
PL13                   19.00 dB
PL2W            12.26963711 W
PL12W            0.27153867 W
PL13W            0.12269637 W
SFO2          400.0416002 MHz

F2 - Processing parameters
SI                        65536
SF            100.5901380 MHz
WDW                          EM
SSB                           0
LB                      1.00 Hz
GB                            0
PC                         1.40
```

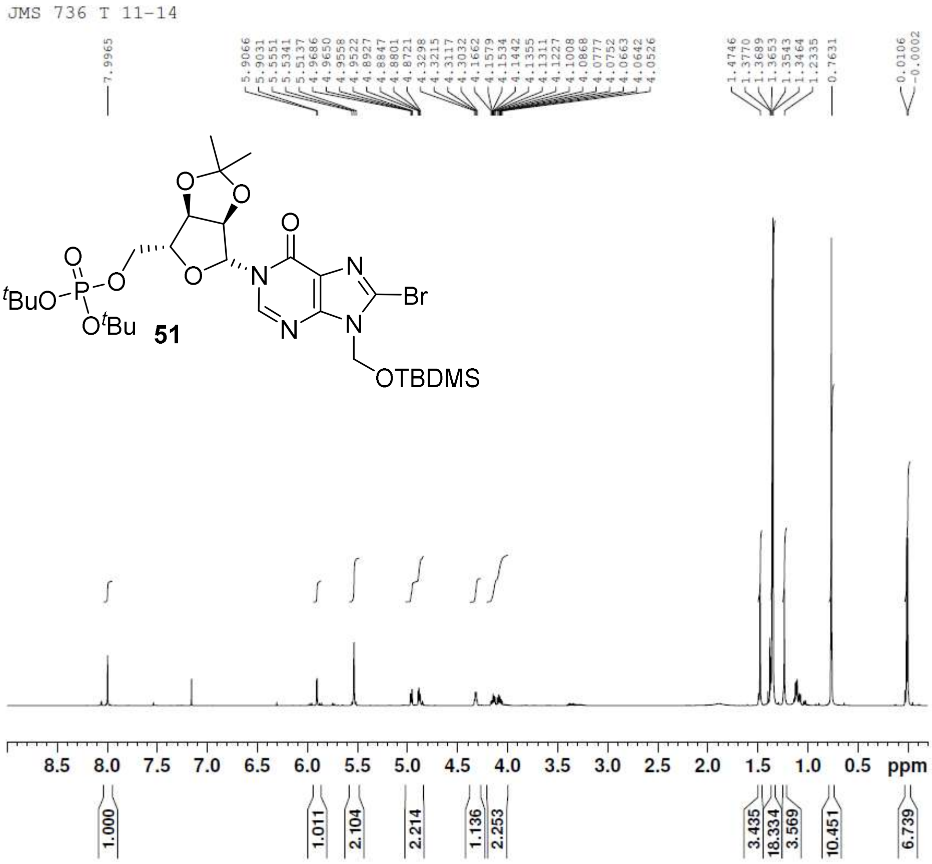


```
Current Data Parameters
NAME      Aug31-2012-JMS736
EXPNO                    10
PROCNO                    1

F2 - Acquisition Parameters
Date_              20120831
Time                  13.21
INSTRUM               spect
PROBHD   5 mm PABBO BB-
PULPROG                zg30
TD                    65536
SOLVENT               CDC13
NS                       16
DS                        2
SWH              10330.578 Hz
FIDRES            0.157632 Hz
AQ               3.1719425 sec
RG                       64
DW                   48.400 usec
DE                    13.94 usec
TE                    298.0 K
D1               1.00000000 sec
TD0                       1

======== CHANNEL f1 ========
NUC1                     1H
P1                    10.30 usec
PL1                   -0.12 dB
PL1W            19.35150909 W
SFO1            500.1330885 MHz

F2 - Processing parameters
SI                    32768
SF              500.1300644 MHz
WDW                      EM
SSB                       0
LB                     0.30 Hz
GB                        0
PC                     1.00
```

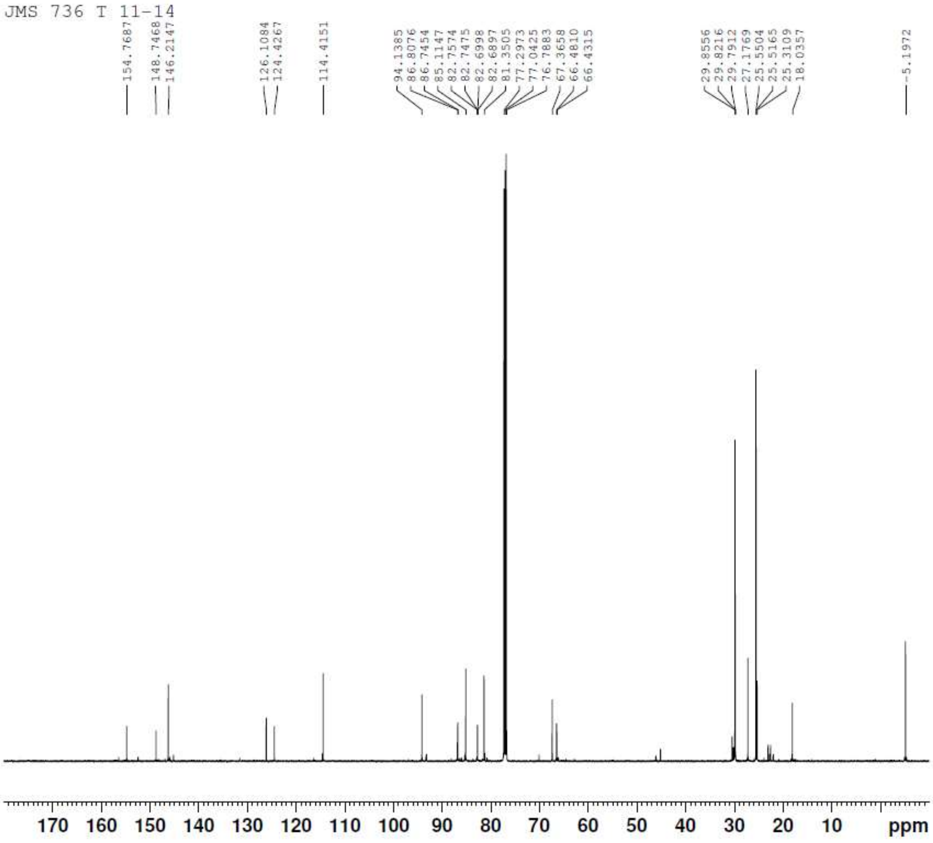


```
Current Data Parameters
NAME      Aug31-2012-JMS736
EXPNO                    12
PROCNO                    1

F2 - Acquisition Parameters
Date_              20120831
Time                  17.53
INSTRUM               spect
PROBHD   5 mm PABBO BB-
PULPROG              zgpg30
TD                    65536
SOLVENT               CDC13
NS                     5000
DS                        4
SWH              29761.904 Hz
FIDRES            0.454131 Hz
AQ               1.1010048 sec
RG                     2050
DW                   16.800 usec
DE                     8.43 usec
TE                    298.0 K
D1               2.00000000 sec
D11              0.03000000 sec
TD0                       1

======== CHANNEL f1 ========
NUC1                    13C
P1                     9.50 usec
PL1                   -0.51 dB
PL1W            99.92730713 W
SFO1            125.7703643 MHz

======== CHANNEL f2 ========
CPDPRG[2            waltz16
NUC2                     1H
PCPD2                 80.00 usec
PL2                   -0.12 dB
PL12                  17.94 dB
PL13                  21.00 dB
PL2W            19.35150909 W
PL12W            0.30249262 W
PL13W            0.14952536 W
SFO2            500.1320005 MHz

F2 - Processing parameters
SI                    32768
SF              125.7577890 MHz
WDW                      EM
SSB                       0
LB                     1.00 Hz
GB                        0
PC                     1.40
```

JMS 746 HPLC again

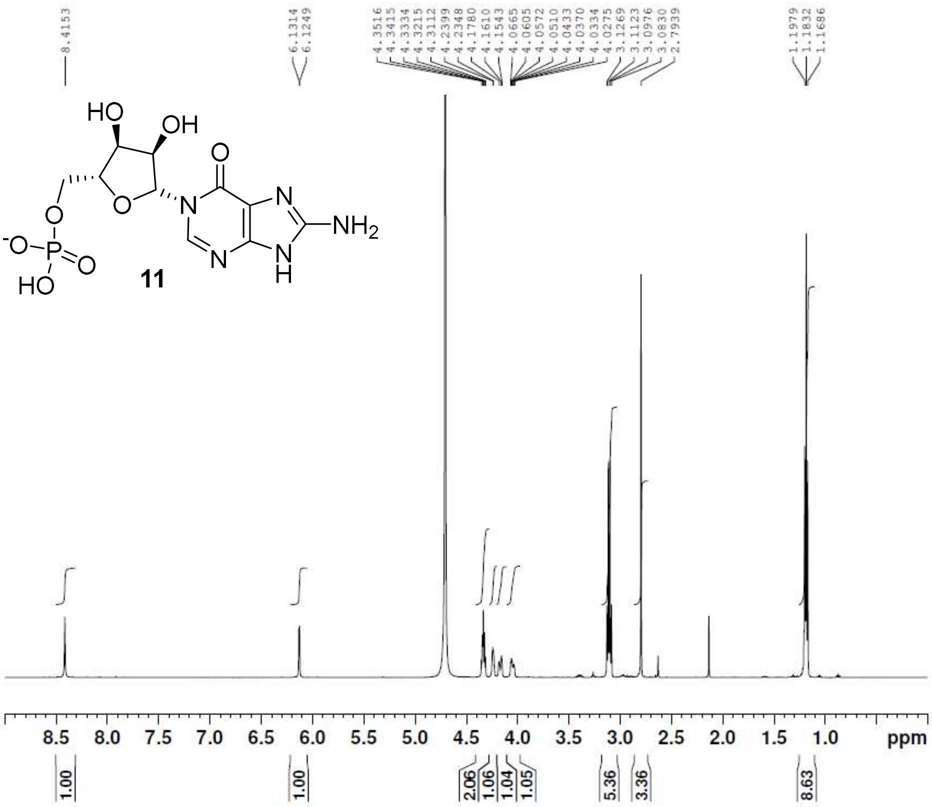

Current Data Parameters
NAME Oct24-2012-JMS 746
EXPNO 10
PROCNO 1

F2 - Acquisition Parameters
Date_ 20121024
Time 18.31
INSTRUM spect
PROBHD 5 mm PABBO BB-
PULPROG zg30
TD 65536
SOLVENT D2O
NS 128
DS 0
SWH 10330.578 Hz
FIDRES 0.157632 Hz
AQ 3.1719425 sec
RG 80.6
DW 48.400 usec
DE 6.50 usec
TE 297.9 K
D1 2.00000000 sec
TD0 1

======== CHANNEL f1 ========
NUC1 1H
P1 10.30 usec
PL1 -0.12 dB
PL1W 19.35150909 W
SFO1 500.1330885 MHz

F2 - Processing parameters
SI 32768
SF 500.1300000 MHz
WDW EM
SSB 0
LB 0.30 Hz
GB 0
PC 1.00

JMS 746 HPLC again

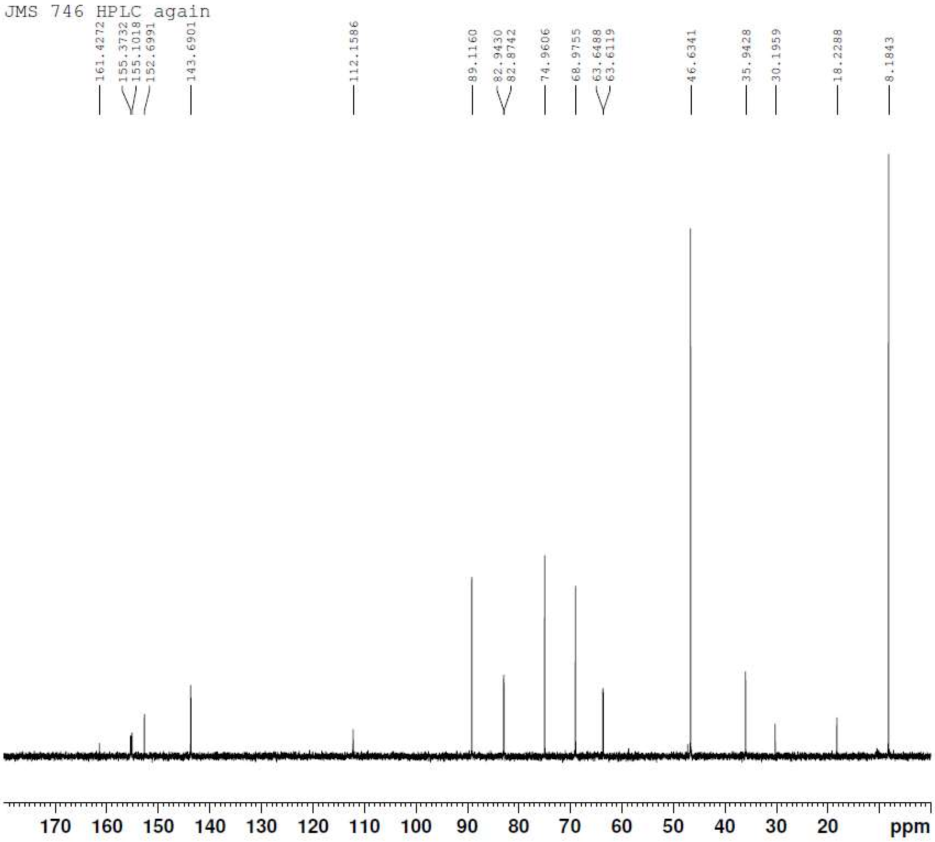

Current Data Parameters
NAME Oct24-2012-JMS 746
EXPNO 12
PROCNO 1

F2 - Acquisition Parameters
Date_ 20121025
Time 5.18
INSTRUM spect
PROBHD 5 mm PABBO BB-
PULPROG zgpg30
TD 65536
SOLVENT D2O
NS 5000
DS 4
SWH 29761.904 Hz
FIDRES 0.454131 Hz
AQ 1.1010048 sec
RG 2050
DW 16.800 usec
DE 8.43 usec
TE 298.0 K
D1 2.00000000 sec
D11 0.03000000 sec
TD0 1

======== CHANNEL f1 ========
NUC1 13C
P1 9.50 usec
PL1 -0.51 dB
PL1W 99.92730713 W
SFO1 125.7703643 MHz

======== CHANNEL f2 ========
CPDPRG[2 waltz16
NUC2 1H
PCPD2 80.00 usec
PL2 -0.12 dB
PL12 17.94 dB
PL13 21.00 dB
PL2W 19.35150909 W
PL12W 0.30249262 W
PL13W 0.14952536 W
SFO2 500.1320005 MHz

F2 - Processing parameters
SI 32768
SF 125.7577890 MHz
WDW EM
SSB 0
LB 1.00 Hz
GB 0
PC 1.40

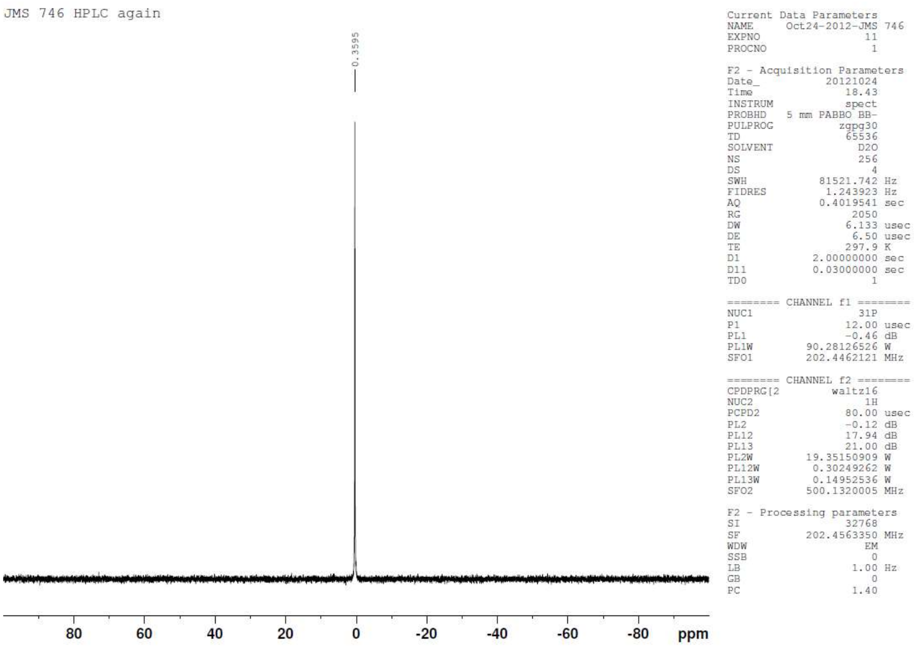

SAMPLE INFORMATION

Sample Name:	JMS 746 hplc	Acquired By:	Joanna
Sample Type:	Unknown	Date Acquired:	25/10/2012 2:59:32 PM
Vial:	2	Acq. Method Set:	isocratic new PDA Jo
Injection #:	1	Date Processed:	09/04/2021 3:09:01 PM
Injection Volume:	10.00 ul	Processing Method:	8_NH2_N1_IMP
Run Time:	15.0 Minutes	Channel Name:	Wvln Ch1
Sample Set Name:	JMS 3 deoxy and 746	Proc. Chnl. Descr.:	PDA 254.0 nm

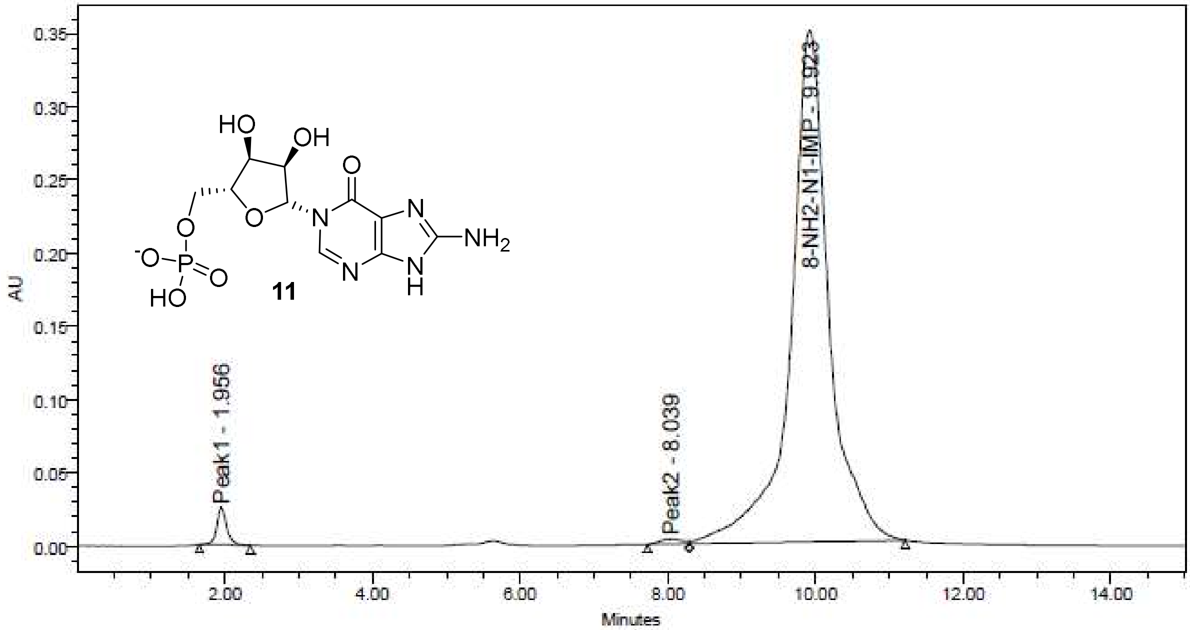

	Peak Name	RT	Area	% Area	Height
1	Peak1	1.956	237655	1.84	25525
2	Peak2	8.039	74802	0.58	3473
3	8-NH2-N1-IMP	9.923	12615673	97.58	348912

*N*1-Ribosyl-inosine analogues (14-17):

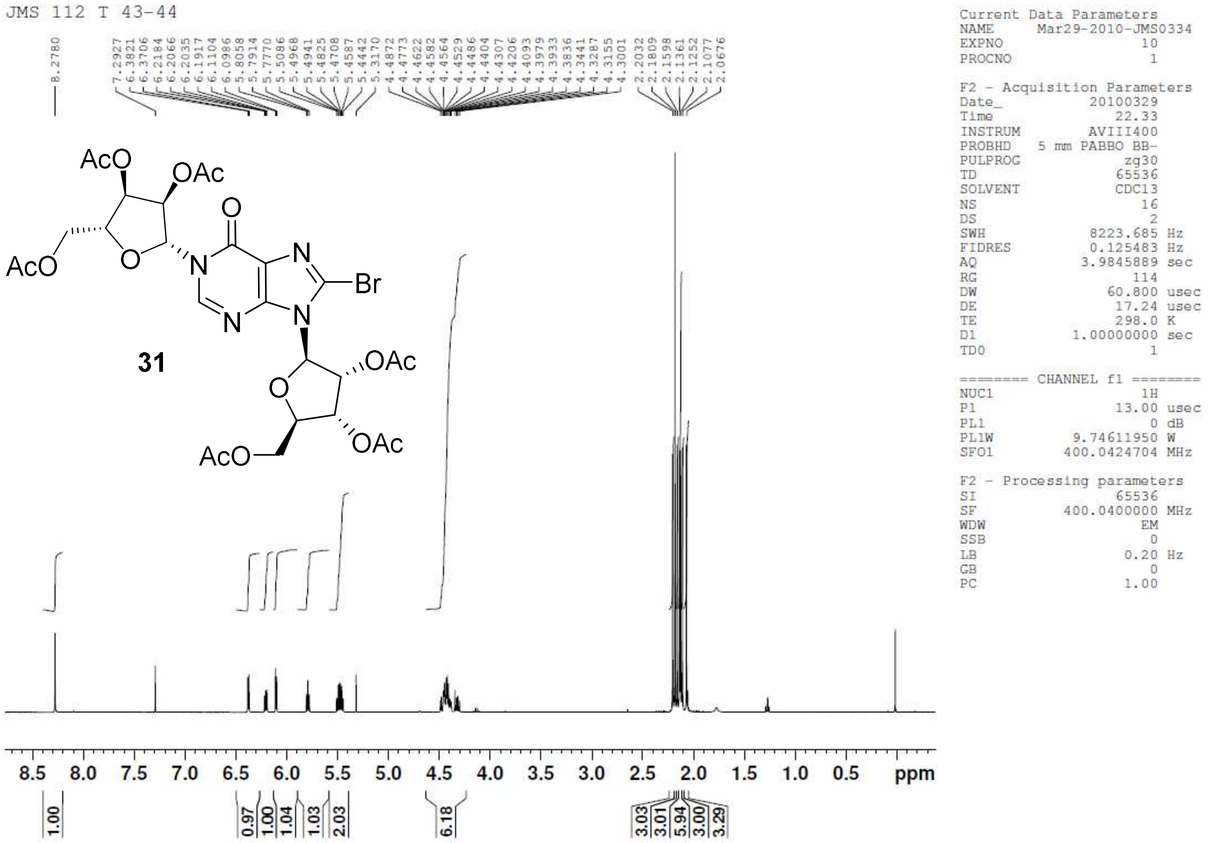

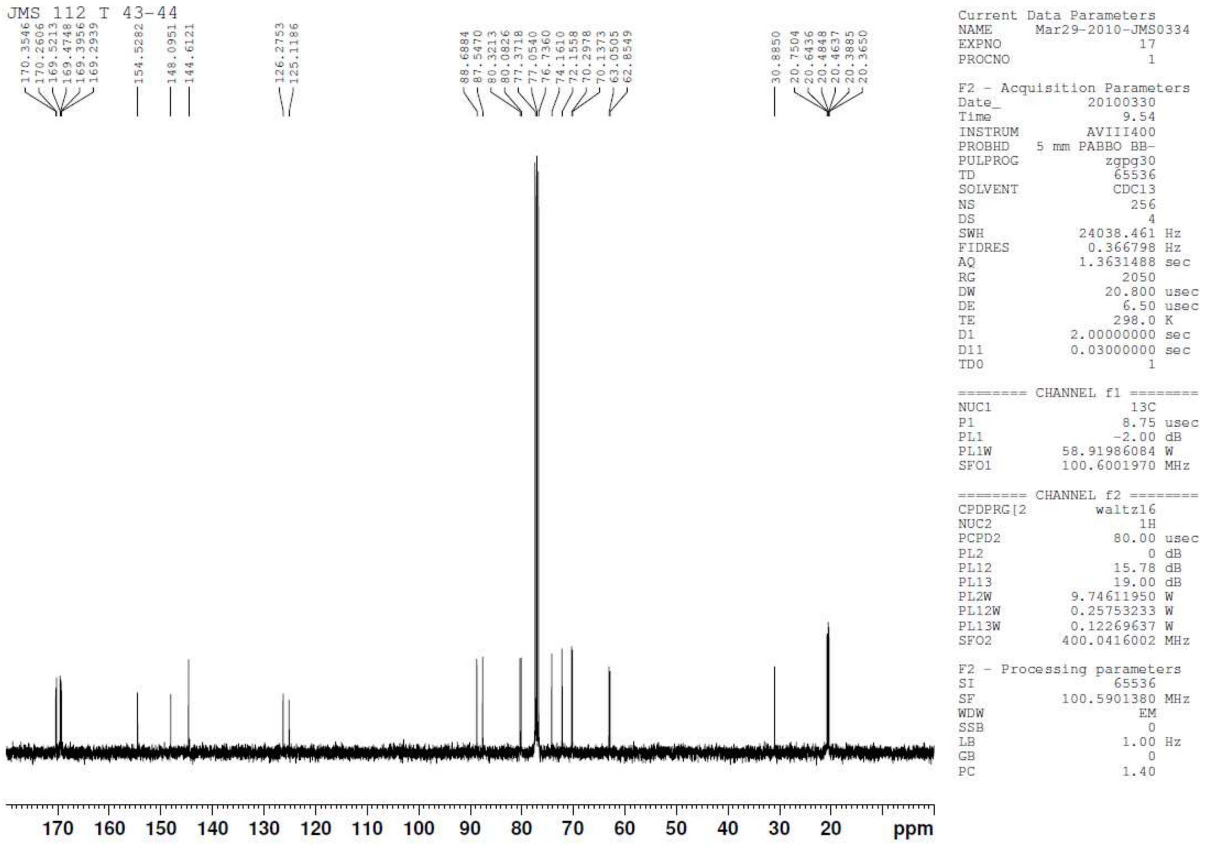

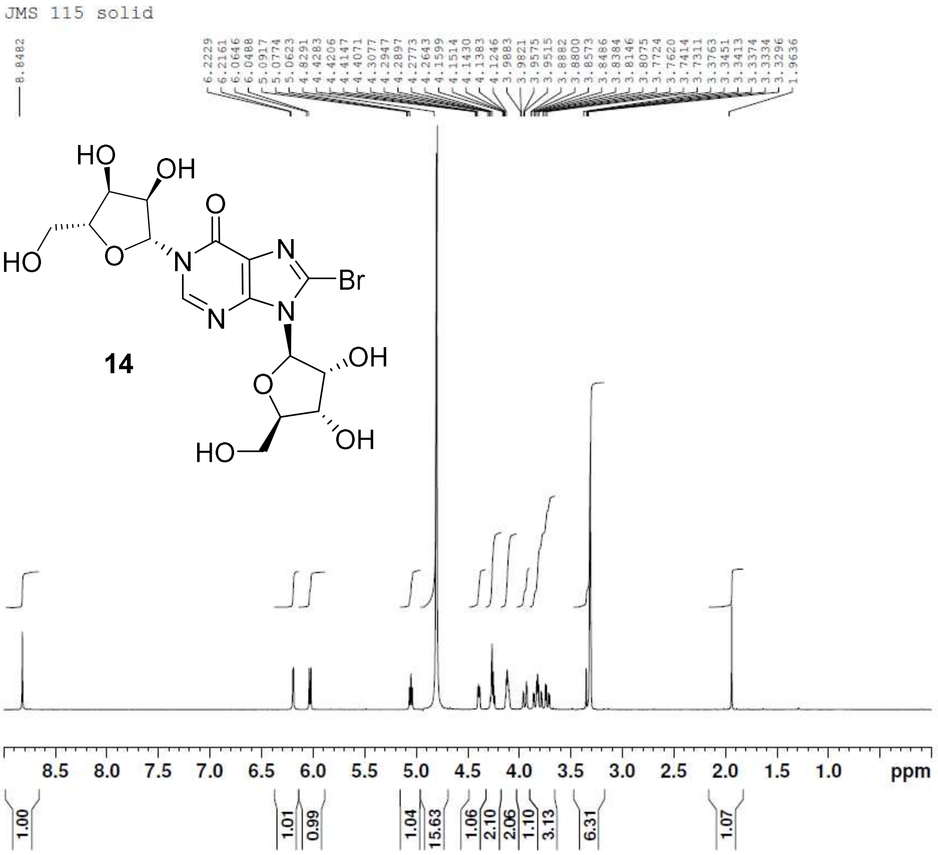

Current Data Parameters
NAME Mar31-2010-JMS0372
EXPNO 10
PROCNO 1

F2 - Acquisition Parameters
Date_ 20100331
Time 11.33
INSTRUM AVIII400
PROBHD 5 mm PABBO BB-
PULPROG zg30
TD 65536
SOLVENT MeOD
NS 16
DS 2
SWH 8223.685 Hz
FIDRES 0.125483 Hz
AQ 3.9845889 sec
RG 144
DW 60.800 usec
DE 6.50 usec
TE 298.0 K
D1 1.00000000 sec
TD0 1

======== CHANNEL f1 ========
NUC1 1H
P1 13.00 usec
PL1 0 dB
PL1W 9.74611950 W
SFO1 400.0424704 MHz

F2 - Processing parameters
SI 32768
SF 400.0400110 MHz
WDW EM
SSB 0
LB 0.30 Hz
GB 0
PC 1.00

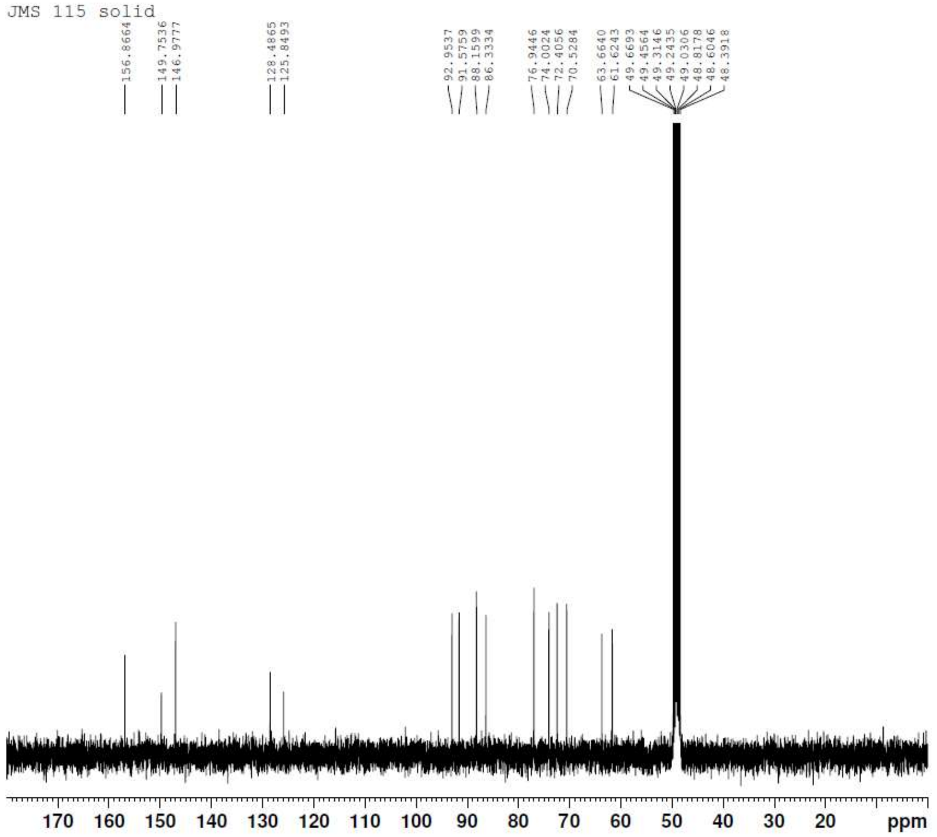

Current Data Parameters
NAME Mar31-2010-JMS0372
EXPNO 11
PROCNO 1

F2 - Acquisition Parameters
Date_ 20100331
Time 14.59
INSTRUM AVIII400
PROBHD 5 mm PABBO BB-
PULPROG zgpg30
TD 65536
SOLVENT MeOD
NS 512
DS 4
SWH 24038.461 Hz
FIDRES 0.366798 Hz
AQ 1.3631488 sec
RG 2050
DW 20.800 usec
DE 6.50 usec
TE 298.0 K
D1 2.00000000 sec
D11 0.03000000 sec
TD0 1

======== CHANNEL f1 ========
NUC1 13C
P1 8.75 usec
PL1 -2.00 dB
PL1W 58.91986084 W
SFO1 100.6001970 MHz

======== CHANNEL f2 ========
CPDPRG[2 waltz16
NUC2 1H
PCPD2 80.00 usec
PL2 0 dB
PL12 15.78 dB
PL13 19.00 dB
PL2W 9.74611950 W
PL12W 0.25753233 W
PL13W 0.12269637 W
SFO2 400.0416002 MHz

F2 - Processing parameters
SI 65536
SF 100.5899952 MHz
WDW EM
SSB 0
LB 1.00 Hz
GB 0
PC 1.40

SAMPLE INFORMATION

Sample Name:	JMS 115 solid	Acquired By:	Christelle
Sample Type:	Unknown	Date Acquired:	04/10/2011 3:37:32 PM
Vial:	6	Acq. Method Set:	RP18 LC
Injection #:	1	Date Processed:	05/10/2011 10:12:15 AM
Injection Volume:	10.00 ul	Processing Method:	JMS 115
Run Time:	20.0 Minutes	Channel Name:	WvlnCh1
Sample Set Name:	JMS 545 JMS 137 JMS 115	Proc. Chnl. Descr.:	PDA 262.1 nm

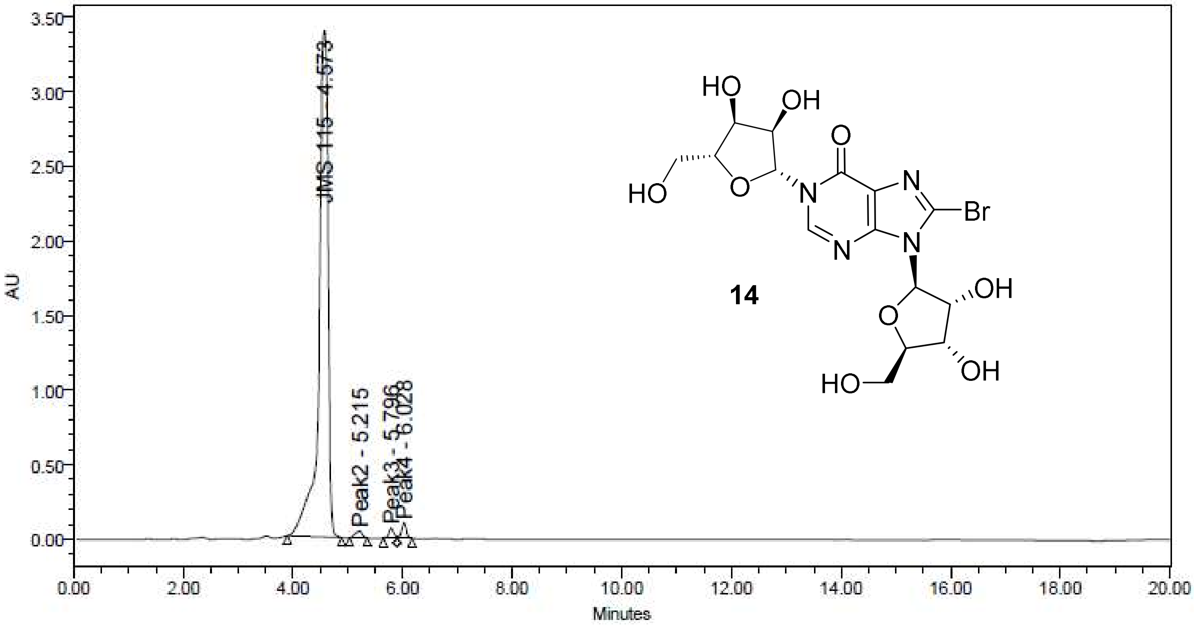

	Peak Name	RT	Area	% Area	Height
1	JMS 115	4.573	39715120	96.29	3391937
2	Peak2	5.215	419731	1.02	46549
3	Peak3	5.796	442067	1.07	65376
4	Peak4	6.028	668389	1.62	108342

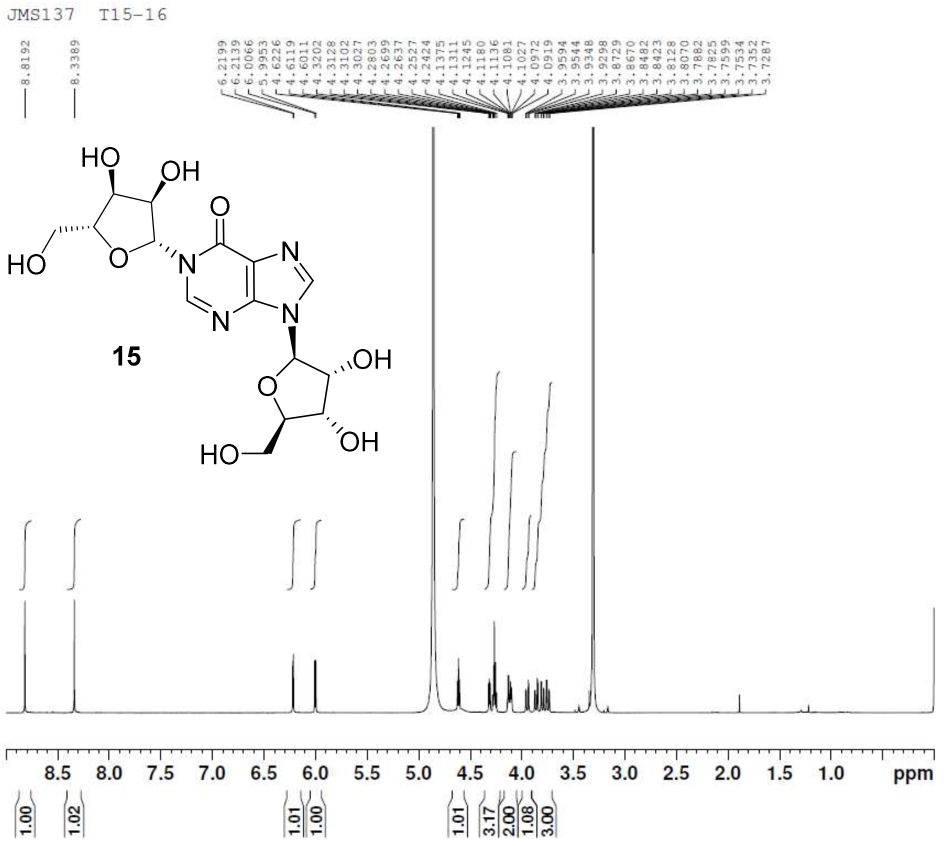

Current Data Parameters
NAME JMS137 T15-16
EXPNO 10
PROCNO 1

F2 - Acquisition Parameters
Date_ 20111007
Time 22.53
INSTRUM spect
PROBHD 5 mm PABBO BB-
PULPROG zg30
TD 65536
SOLVENT MeOD
NS 256
DS 2
SWH 10330.578 Hz
FIDRES 0.157632 Hz
AQ 3.1719425 sec
RG 228
DW 48.400 usec
DE 13.94 usec
TE 298.0 K
D1 1.00000000 sec
TD0 1

======== CHANNEL f1 ========
NUC1 1H
P1 10.30 usec
PL1 -0.12 dB
PL1W 19.35150909 W
SFO1 500.1330885 MHz

F2 - Processing parameters
SI 32768
SF 500.1300131 MHz
WDW EM
SSB 0
LB 0.30 Hz
GB 0
PC 1.00

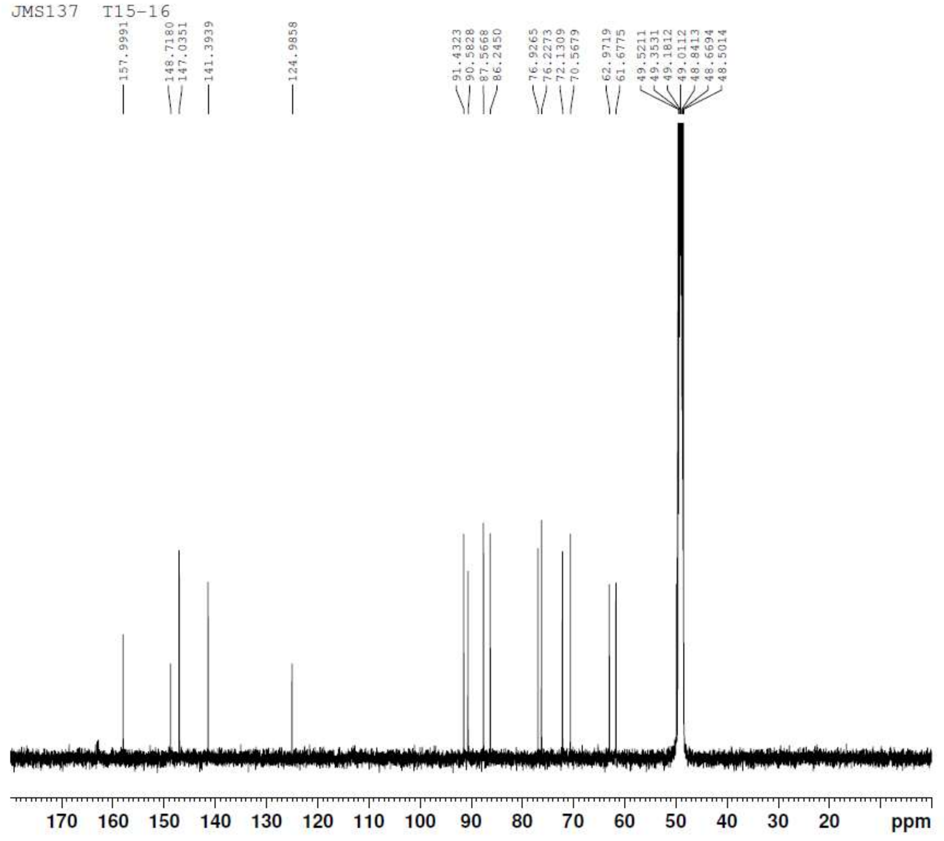

Current Data Parameters
NAME JMS137 T15-16
EXPNO 11
PROCNO 1

F2 - Acquisition Parameters
Date_ 20111010
Time 4.55
INSTRUM spect
PROBHD 5 mm PABBO BB-
PULPROG zgpg30
TD 65536
SOLVENT MeOD
NS 61440
DS 4
SWH 29761.904 Hz
FIDRES 0.454131 Hz
AQ 1.1010048 sec
RG 2050
DW 16.800 usec
DE 8.43 usec
TE 298.0 K
D1 2.00000000 sec
D11 0.03000000 sec
TD0 1

======== CHANNEL f1 ========
NUC1 13C
P1 9.50 usec
PL1 -0.51 dB
PL1W 99.92730713 W
SFO1 125.7703643 MHz

======== CHANNEL f2 ========
CPDPRG[2 waltz16
NUC2 1H
PCPD2 80.00 usec
PL2 -0.12 dB
PL12 17.94 dB
PL13 21.00 dB
PL2W 19.35150909 W
PL12W 0.30249262 W
PL13W 0.14952536 W
SFO2 500.1320005 MHz

F2 - Processing parameters
SI 32768
SF 125.7576104 MHz
WDW EM
SSB 0
LB 1.00 Hz
GB 0
PC 1.40

SAMPLE INFORMATION			
Sample Name:	JMS 137 HPLC	Acquired By:	Joanna
Sample Type:	Unknown	Date Acquired:	17/10/2011 3:27:24 PM
Vial:	26	Acq. Method Set:	RP18 LC
Injection #:	1	Date Processed:	17/10/2011 3:49:45 PM
Injection Volume:	10.00 ul	Processing Method:	JMS 137 Purified
Run Time:	20.0 Minutes	Channel Name:	WvlnCh1
Sample Set Name:	JMS 137 HPLC 2	Proc. Chnl. Descr.:	PDA 262.1 nm

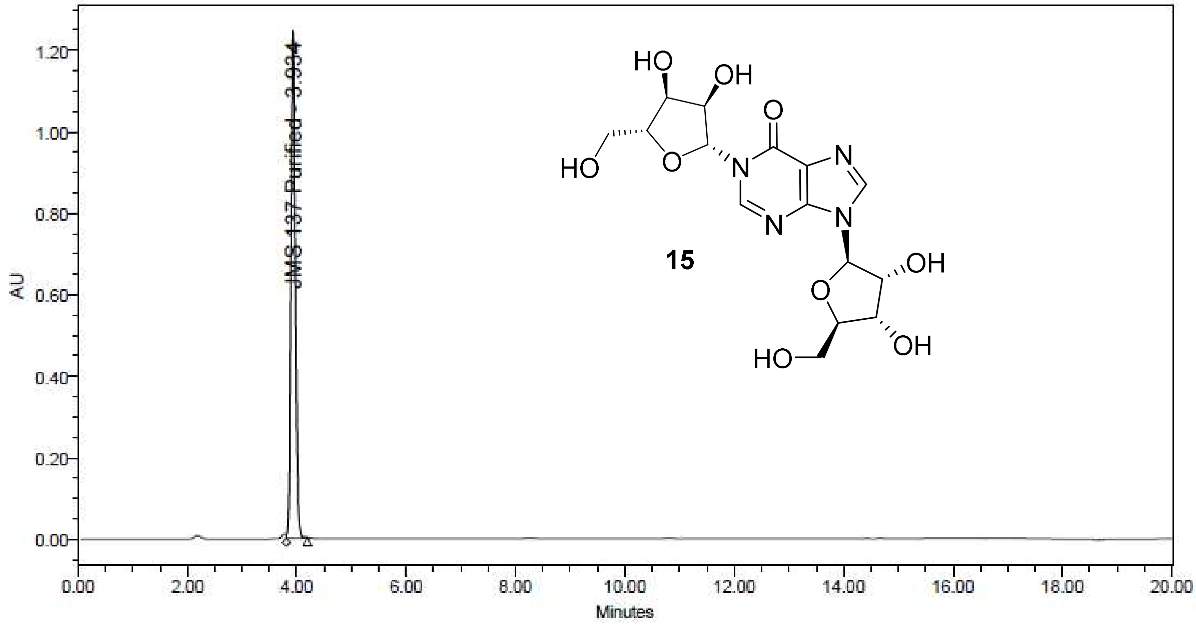

	Peak Name	RT	Area	% Area	Height
1	JMS 137 Purified	3.934	7025028	100.00	1244149

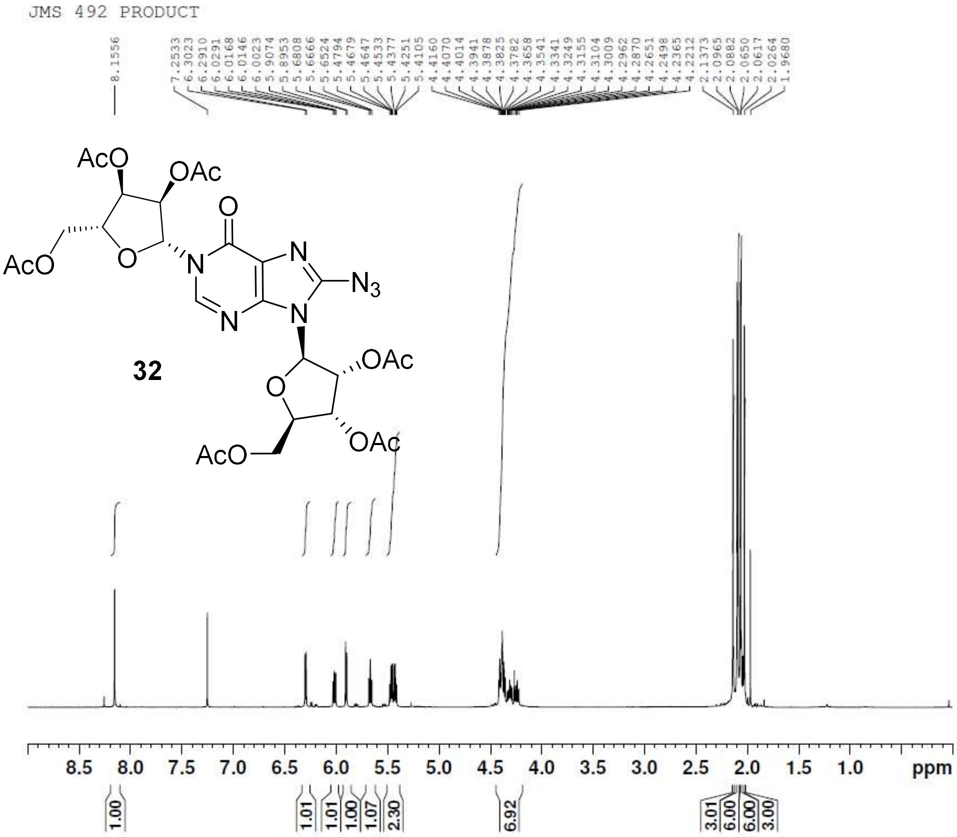


```
Current Data Parameters
NAME       Jul26-2011-JMS11649
EXPNO                   10
PROCNO                   1

F2 - Acquisition Parameters
Date_             20110726
Time                 17.25
INSTRUM           AVIII400
PROBHD   5 mm PABBO BB-
PULPROG               zg30
TD                   65536
SOLVENT              CDCl3
NS                      16
DS                       2
SWH               8223.685 Hz
FIDRES            0.125483 Hz
AQ               3.9845889 sec
RG                    71.8
DW                  60.800 usec
DE                   17.24 usec
TE                   298.0 K
D1              1.00000000 sec
TD0                      1

======== CHANNEL f1 ========
NUC1                    1H
P1                   13.00 usec
PL1                      0 dB
PL1W            9.74611950 W
SFO1           400.0424704 MHz

F2 - Processing parameters
SI                   65536
SF             400.0399837 MHz
WDW                     EM
SSB                      0
LB                    0.20 Hz
GB                       0
PC                    1.00
```

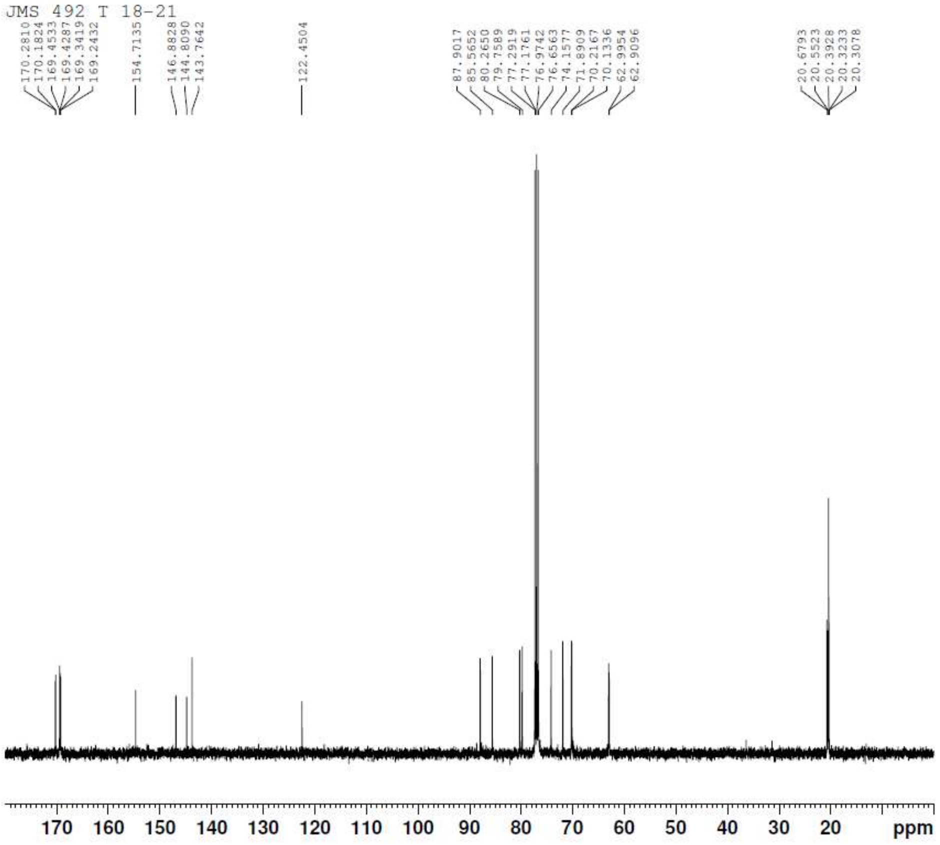


```
Current Data Parameters
NAME       Jul18-2011-JMS11462
EXPNO                   11
PROCNO                   1

F2 - Acquisition Parameters
Date_             20110719
Time                  0.59
INSTRUM           AVIII400
PROBHD   5 mm PABBO BB-
PULPROG            zgpg30
TD                   65536
SOLVENT              CDCl3
NS                     512
DS                       4
SWH              24038.461 Hz
FIDRES            0.366798 Hz
AQ               1.3631488 sec
RG                    2050
DW                  20.800 usec
DE                    6.50 usec
TE                   298.0 K
D1              2.00000000 sec
D11             0.03000000 sec
TD0                      1

======== CHANNEL f1 ========
NUC1                   13C
P1                    8.75 usec
PL1                  -2.00 dB
PL1W           58.91986084 W
SFO1           100.6001970 MHz

======== CHANNEL f2 ========
CPDPRG[2           waltz16
NUC2                    1H
PCPD2                80.00 usec
PL2                      0 dB
PL12                 15.78 dB
PL13                 19.00 dB
PL2W            9.74611950 W
PL12W           0.25753233 W
PL13W           0.12269637 W
SFO2           400.0416002 MHz

F2 - Processing parameters
SI                   65536
SF             100.5901380 MHz
WDW                     EM
SSB                      0
LB                    1.00 Hz
GB                       0
PC                    1.40
```

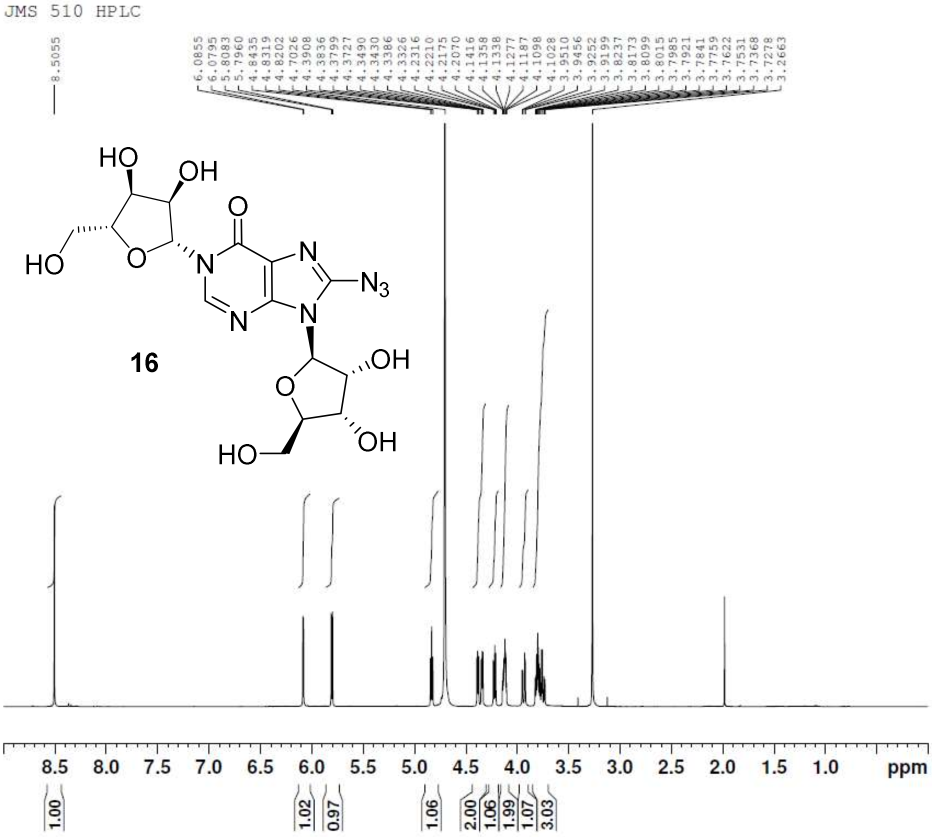

Current Data Parameters
NAME JMS510HPLC
EXPNO 10
PROCNO 1

F2 - Acquisition Parameters
Date_ 20111019
Time 9.17
INSTRUM spect
PROBHD 5 mm PABBO BB-
PULPROG zg30
TD 65536
SOLVENT D2O
NS 732
DS 2
SWH 10330.578 Hz
FIDRES 0.157632 Hz
AQ 3.1719425 sec
RG 128
DW 48.400 usec
DE 13.94 usec
TE 298.0 K
D1 1.00000000 sec
TD0 1

======== CHANNEL f1 ========
NUC1 1H
P1 10.30 usec
PL1 -0.12 dB
PL1W 19.35150909 W
SFO1 500.1330885 MHz

F2 - Processing parameters
SI 32768
SF 500.1300000 MHz
WDW EM
SSB 0
LB 0.30 Hz
GB 0
PC 1.00

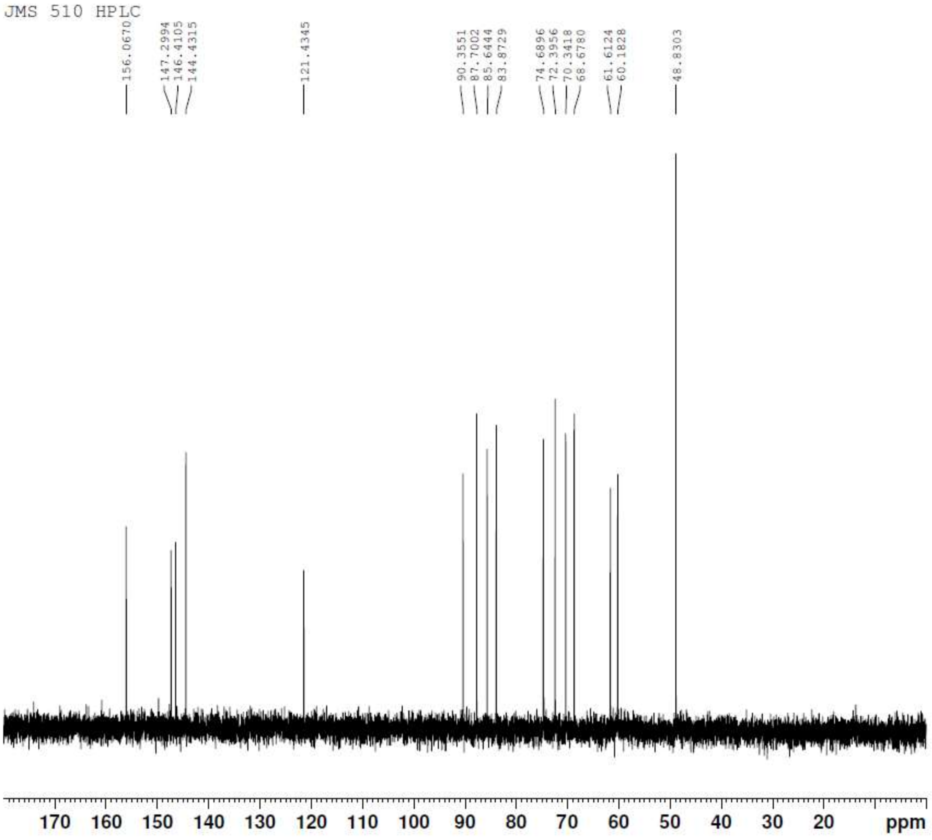

Current Data Parameters
NAME JMS510HPLC
EXPNO 11
PROCNO 1

F2 - Acquisition Parameters
Date_ 20111019
Time 10.10
INSTRUM spect
PROBHD 5 mm PABBO BB-
PULPROG zgpg30
TD 65536
SOLVENT D2O
NS 827
DS 4
SWH 29761.904 Hz
FIDRES 0.454131 Hz
AQ 1.1010048 sec
RG 2050
DW 16.800 usec
DE 8.43 usec
TE 298.2 K
D1 2.00000000 sec
D11 0.03000000 sec
TD0 1

======== CHANNEL f1 ========
NUC1 13C
P1 9.50 usec
PL1 -0.51 dB
PL1W 99.92730713 W
SFO1 125.7703643 MHz

======== CHANNEL f2 ========
CPDPRG[2 waltz16
NUC2 1H
PCPD2 80.00 usec
PL2 -0.12 dB
PL12 17.94 dB
PL13 21.00 dB
PL2W 19.35150909 W
PL12W 0.30249262 W
PL13W 0.14952536 W
SFO2 500.1320005 MHz

F2 - Processing parameters
SI 32768
SF 125.7577890 MHz
WDW EM
SSB 0
LB 1.00 Hz
GB 0
PC 1.40

SAMPLE INFORMATION

Sample Name:	JMS 510 HPLC	Acquired By:	Christelle
Sample Type:	Unknown	Date Acquired:	25/10/2011 5:14:47 PM
Vial:	1	Acq. Method Set:	RP18 LC
Injection #:	1	Date Processed:	28/10/2011 12:20:44 PM
Injection Volume:	10.00 ul	Processing Method:	JMS 510 HPLC
Run Time:	20.0 Minutes	Channel Name:	WvlnCh1
Sample Set Name:	JMS 510 HPLC	Proc. Chnl. Descr.:	PDA 262.1 nm

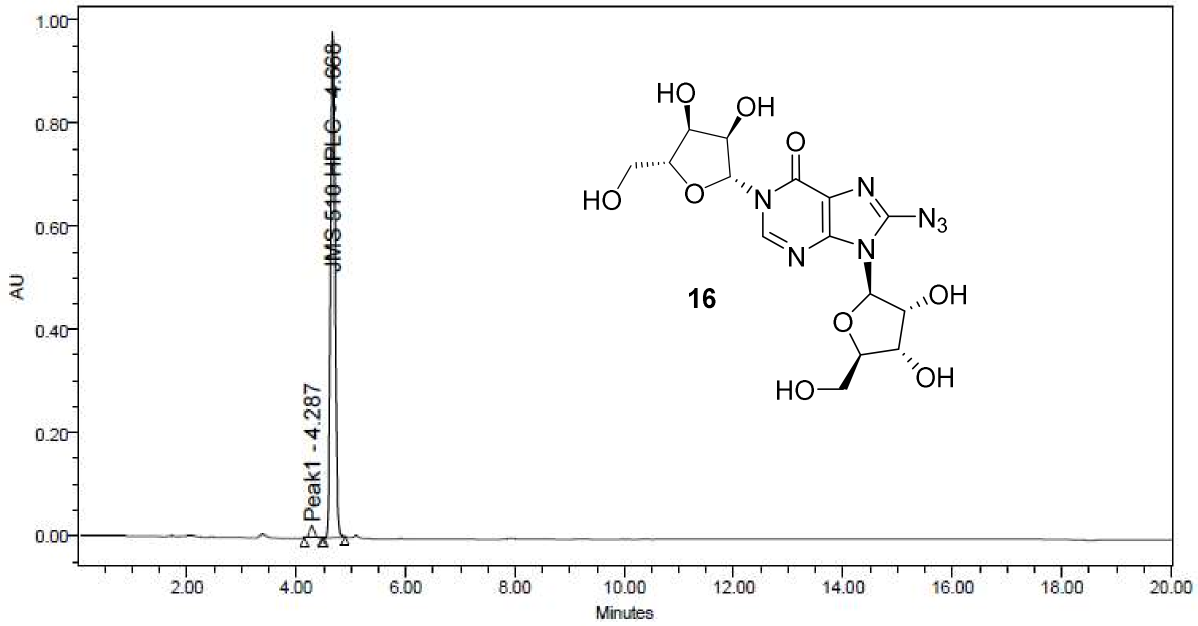

	Peak Name	RT	Area	% Area	Height
1	Peak1	4.287	143472	2.55	23047
2	JMS 510 HPLC	4.668	5486409	97.45	979291

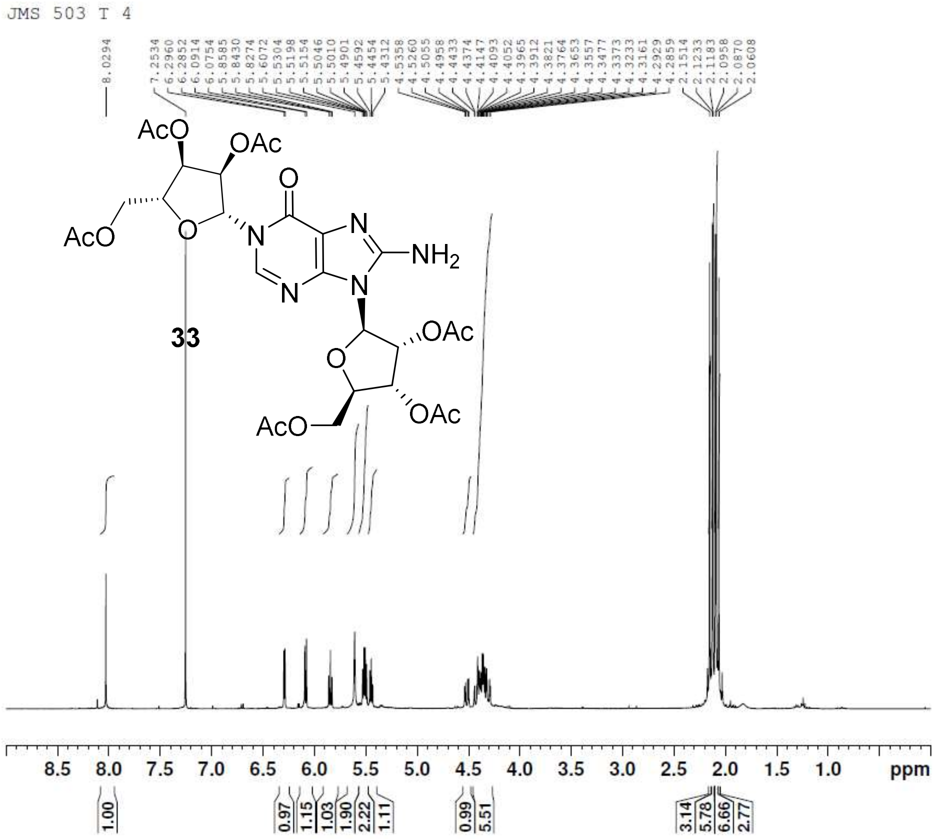

Current Data Parameters
NAME Jul26-2011-JMS11651
EXPNO 10
PROCNO 1

F2 - Acquisition Parameters
Date_ 20110726
Time 17.15
INSTRUM AVIII400
PROBHD 5 mm PABBO BB-
PULPROG zg30
TD 65536
SOLVENT CDCl3
NS 16
DS 2
SWH 8223.685 Hz
FIDRES 0.125483 Hz
AQ 3.9845889 sec
RG 144
DW 60.800 usec
DE 17.24 usec
TE 298.0 K
D1 1.00000000 sec
TD0 1

======== CHANNEL f1 ========
NUC1 1H
P1 13.00 usec
PL1 0 dB
PL1W 9.74611950 W
SFO1 400.0424704 MHz

F2 - Processing parameters
SI 65536
SF 400.0399837 MHz
WDW EM
SSB 0
LB 0.20 Hz
GB 0
PC 1.00

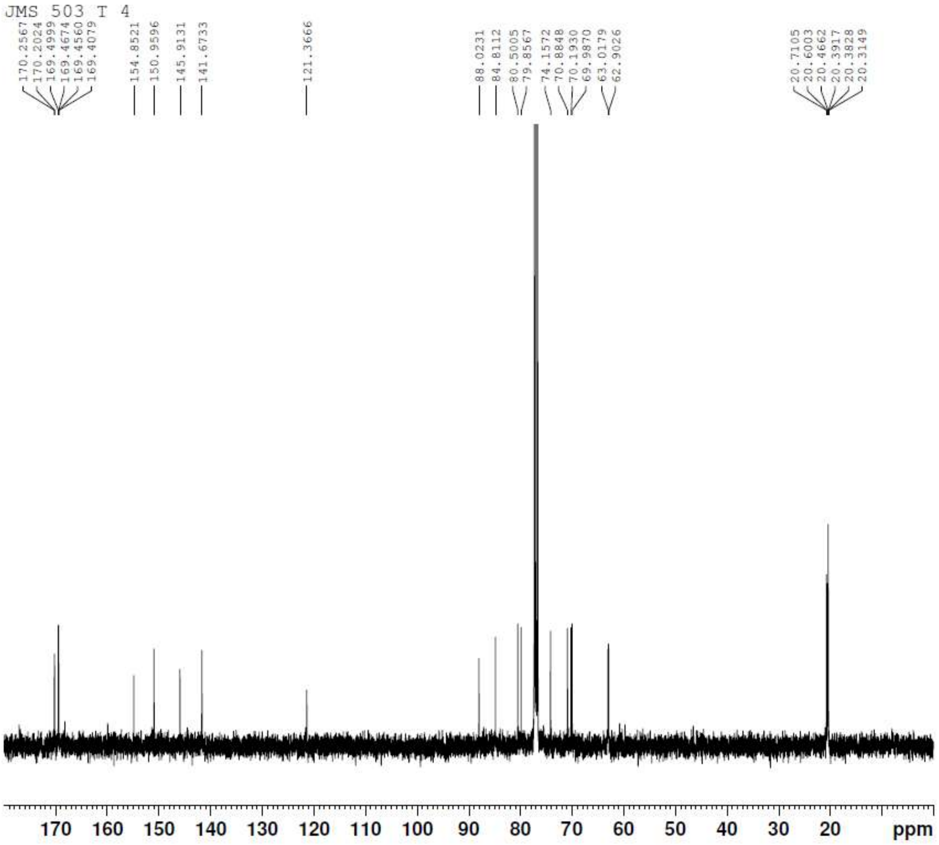

Current Data Parameters
NAME Jul26-2011-JMS11651
EXPNO 14
PROCNO 1

F2 - Acquisition Parameters
Date_ 20110726
Time 19.43
INSTRUM AVIII400
PROBHD 5 mm PABBO BB-
PULPROG zgpg30
TD 65536
SOLVENT CDCl3
NS 512
DS 4
SWH 24038.461 Hz
FIDRES 0.366798 Hz
AQ 1.3631488 sec
RG 2050
DW 20.800 usec
DE 6.50 usec
TE 298.0 K
D1 2.00000000 sec
D11 0.03000000 sec
TD0 1

======== CHANNEL f1 ========
NUC1 13C
P1 8.75 usec
PL1 -2.00 dB
PL1W 58.91986084 W
SFO1 100.6001970 MHz

======== CHANNEL f2 ========
CPDPRG[2 waltz16
NUC2 1H
PCPD2 80.00 usec
PL2 0 dB
PL12 15.78 dB
PL13 19.00 dB
PL2W 9.74611950 W
PL12W 0.25753233 W
PL13W 0.12269637 W
SFO2 400.0416002 MHz

F2 - Processing parameters
SI 65536
SF 100.5901380 MHz
WDW EM
SSB 0
LB 1.00 Hz
GB 0
PC 1.40

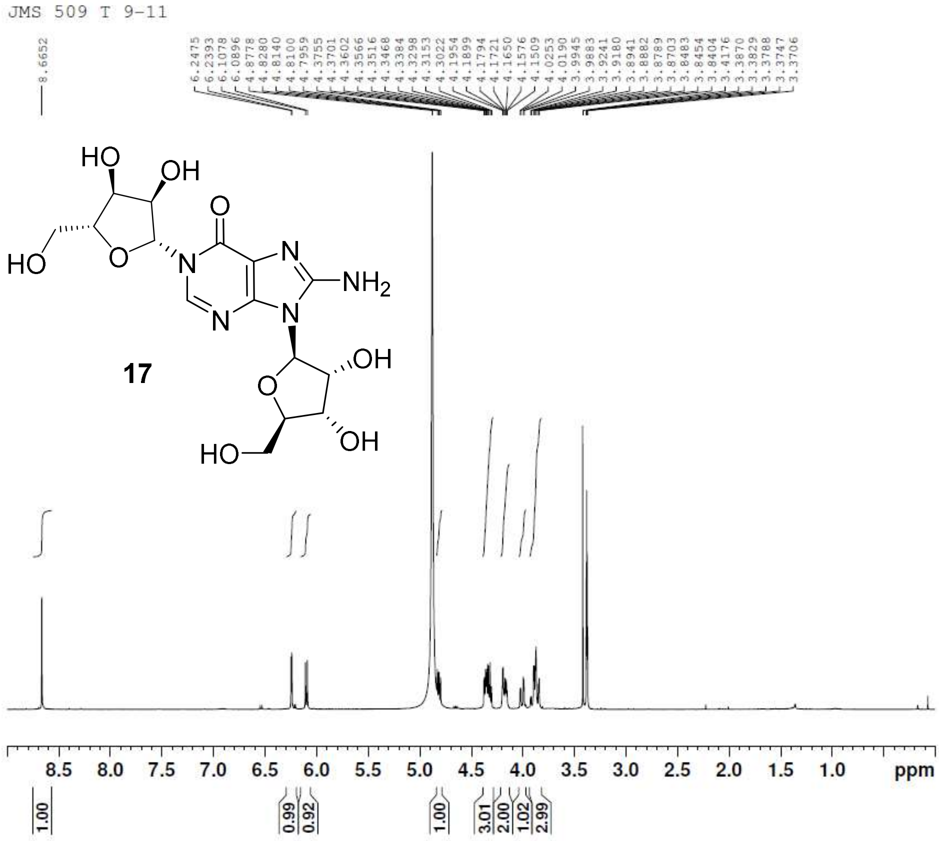


```
Current Data Parameters
NAME      Aug08-2011-JMS11943
EXPNO                      10
PROCNO                      1

F2 - Acquisition Parameters
Date_               20110808
Time                   16.58
INSTRUM             AVIII400
PROBHD   5 mm PABBO BB-
PULPROG                 zg30
TD                     65536
SOLVENT                 MeOD
NS                        16
DS                         2
SWH                 8223.685 Hz
FIDRES              0.125483 Hz
AQ                 3.9845889 sec
RG                       161
DW                    60.800 usec
DE                     17.24 usec
TE                     298.0 K
D1                1.00000000 sec
TD0                        1

======== CHANNEL f1 ========
NUC1                      1H
P1                     13.00 usec
PL1                        0 dB
PL1W              9.74611950 W
SFO1             400.0424704 MHz

F2 - Processing parameters
SI                     65536
SF               400.0399837 MHz
WDW                       EM
SSB                        0
LB                      0.20 Hz
GB                         0
PC                      1.00
```

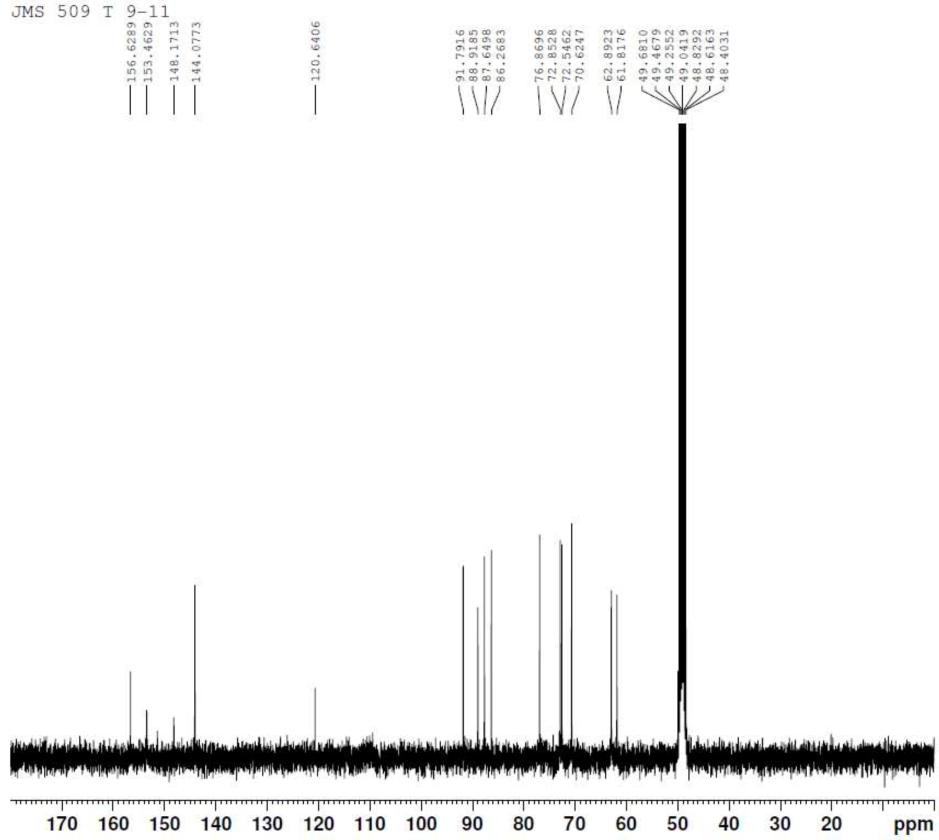


```
Current Data Parameters
NAME      Aug08-2011-JMS11943
EXPNO                      11
PROCNO                      1

F2 - Acquisition Parameters
Date_               20110809
Time                    5.47
INSTRUM             AVIII400
PROBHD   5 mm PABBO BB-
PULPROG               zgpg30
TD                     65536
SOLVENT                 MeOD
NS                      1024
DS                         4
SWH                24038.461 Hz
FIDRES              0.366798 Hz
AQ                 1.3631488 sec
RG                      2050
DW                    20.800 usec
DE                      6.50 usec
TE                     298.0 K
D1                2.00000000 sec
D11               0.03000000 sec
TD0                        1

======== CHANNEL f1 ========
NUC1                     13C
P1                      8.75 usec
PL1                    -2.00 dB
PL1W             58.91986084 W
SFO1             100.6001970 MHz

======== CHANNEL f2 ========
CPDPRG[2             waltz16
NUC2                      1H
PCPD2                  80.00 usec
PL2                        0 dB
PL12                   15.78 dB
PL13                   19.00 dB
PL2W              9.74611950 W
PL12W             0.25753233 W
PL13W             0.12269637 W
SFO2             400.0416002 MHz

F2 - Processing parameters
SI                     65536
SF               100.5899952 MHz
WDW                       EM
SSB                        0
LB                      1.00 Hz
GB                         0
PC                      1.40
```

SAMPLE INFORMATION

Sample Name:	JMS 509	Acquired By:	Joanna
Sample Type:	Unknown	Date Acquired:	17/08/2011 3:48:55 PM
Vial:	31	Acq. Method Set:	RP18 LC
Injection #:	1	Date Processed:	18/08/2011 5:45:27 PM
Injection Volume:	10.00 ul	Processing Method:	JMS 509
Run Time:	20.0 Minutes	Channel Name:	WvlnCh1
Sample Set Name:	JMS 509 510 519 533	Proc. Chnl. Descr.:	PDA 262.1 nm

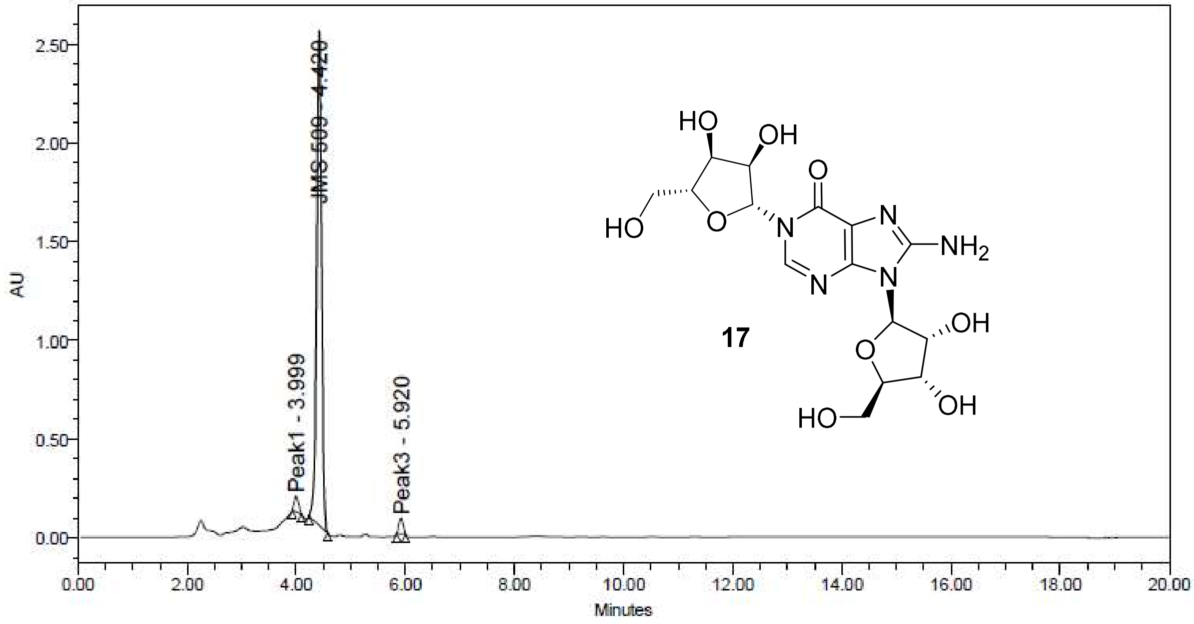

	Peak Name	RT	Area	% Area	Height
1	Peak1	3.999	428563	2.53	77203
2	JMS 509	4.420	16065701	95.02	2513493
3	Peak3	5.920	413227	2.44	80736

<u>**Total synthesis of *N*9-(4-hydroxybutyl)-*N*1-Inosine analogues (18-21)**</u>

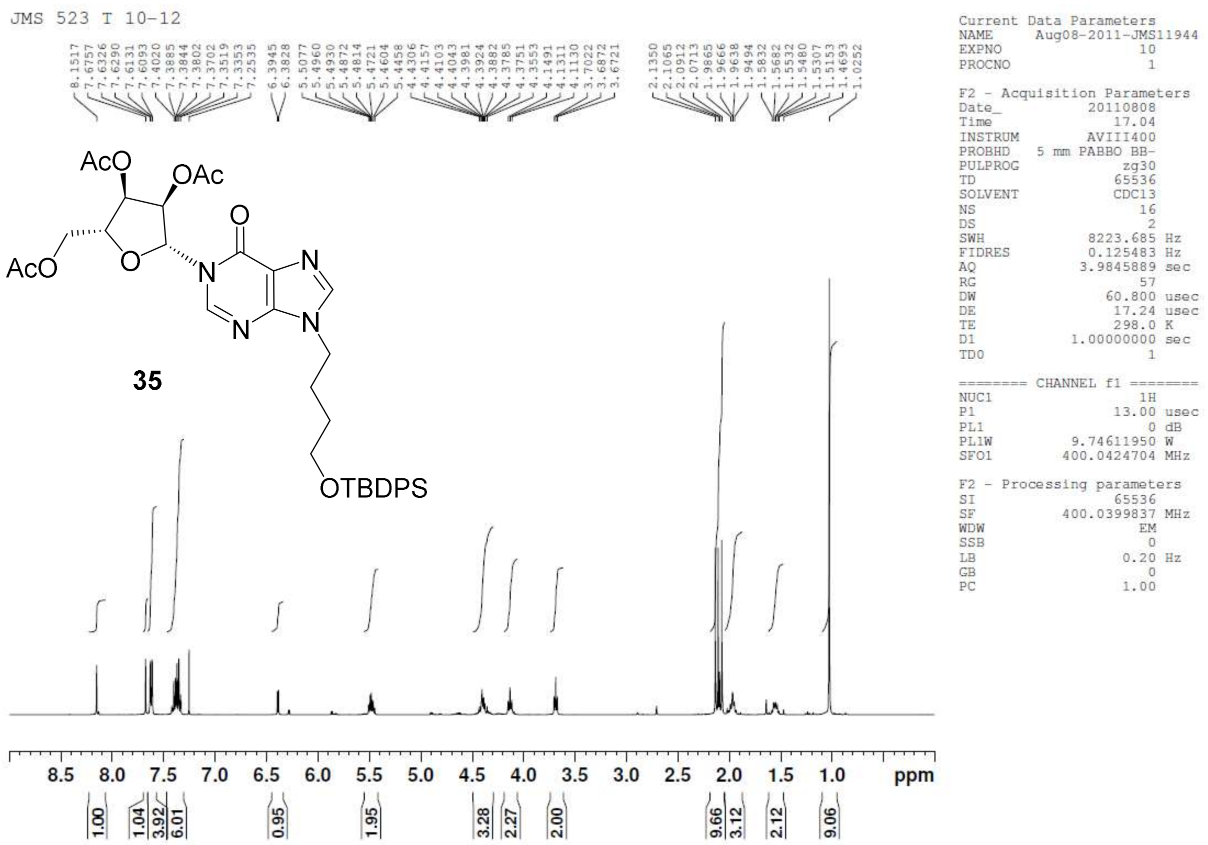

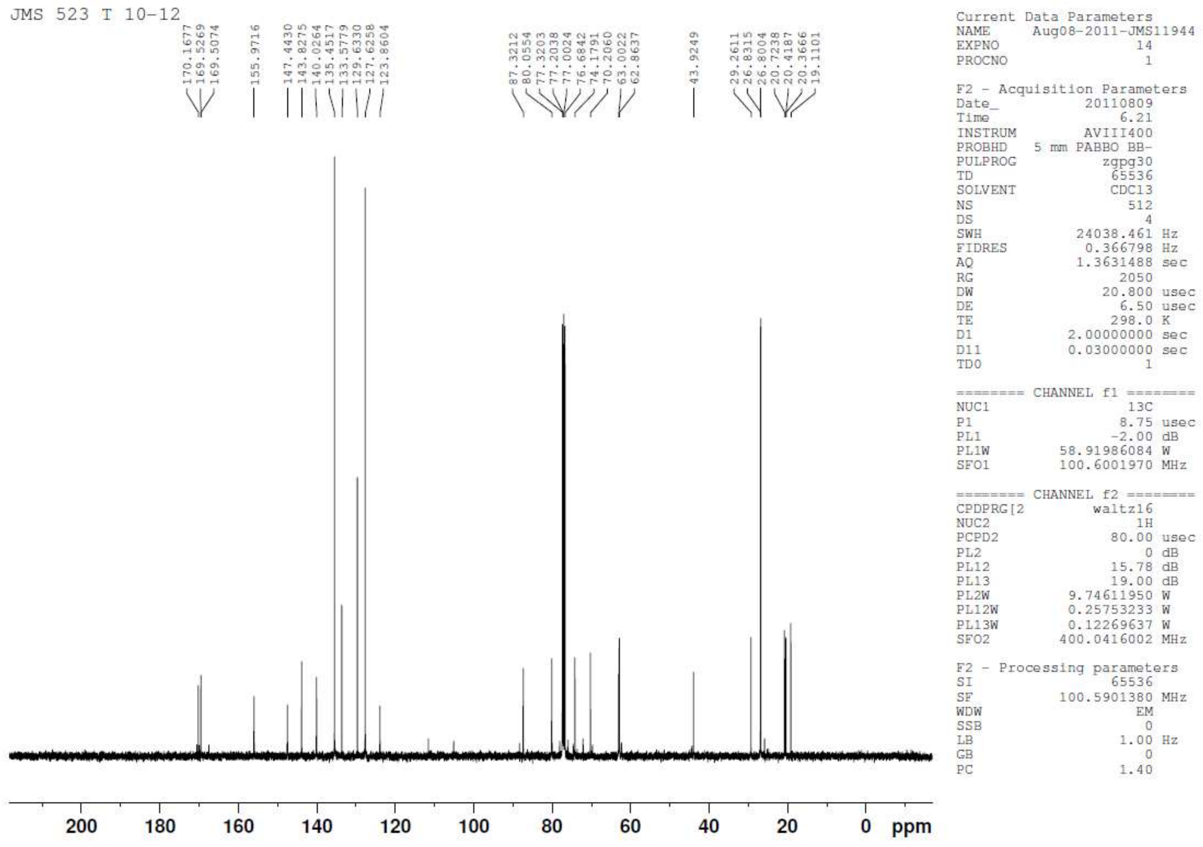

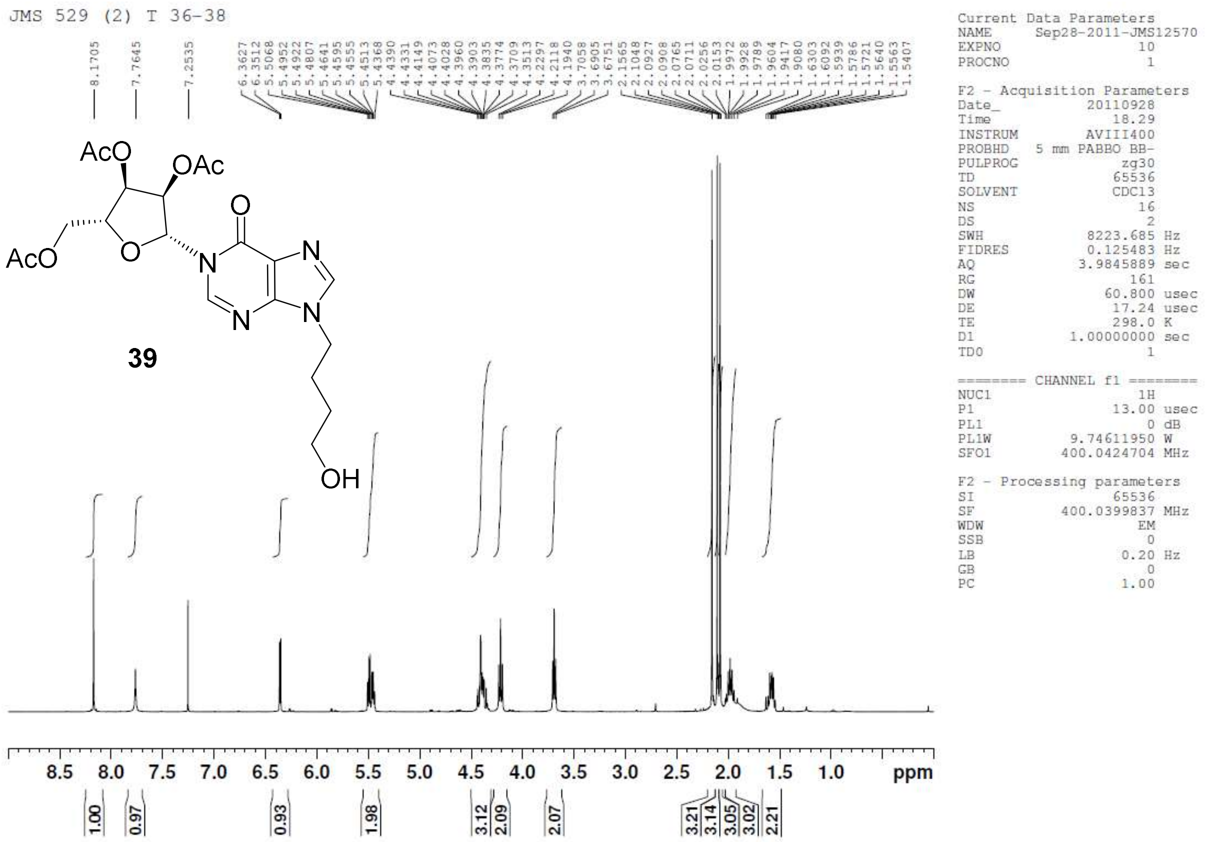
JMS 529 (2) T 36-38
8.1705 7.7645 7.2535 6.3627 6.3512 5.5068 5.4952 5.4922 5.4807 5.4641 5.4595 5.4555 5.4513 5.4368 4.4390 4.4331 4.4149 4.4073 4.4028 4.3960 4.3903 4.3835 4.3774 4.3709 4.3513 4.2297 4.2118 4.1940 3.7058 3.6905 3.6751 2.1565 2.1048 2.0927 2.0908 2.0765 2.0711 2.0256 2.0153 1.9972 1.9928 1.9789 1.9604 1.9417 1.9080 1.6303 1.6092 1.5939 1.5786 1.5721 1.5640 1.5563 1.5407
AcO
OAc
AcO
O
N
N
N
N
OH
39
8.5 8.0 7.5 7.0 6.5 6.0 5.5 5.0 4.5 4.0 3.5 3.0 2.5 2.0 1.5 1.0 ppm
1.00 0.97 0.93 1.98 3.12 2.09 2.07 3.21 3.14 3.05 3.02 2.21
Current Data Parameters
NAME Sep28-2011-JMS12570
EXPNO 10
PROCNO 1
F2 - Acquisition Parameters
Date_ 20110928
Time 18.29
INSTRUM AVIII400
PROBHD 5 mm PABBO BB-
PULPROG zg30
TD 65536
SOLVENT CDCl3
NS 16
DS 2
SWH 8223.685 Hz
FIDRES 0.125483 Hz
AQ 3.9845889 sec
RG 161
DW 60.800 usec
DE 17.24 usec
TE 298.0 K
D1 1.00000000 sec
TD0 1
======== CHANNEL f1 ========
NUC1 1H
P1 13.00 usec
PL1 0 dB
PL1W 9.74611950 W
SFO1 400.0424704 MHz
F2 - Processing parameters
SI 65536
SF 400.0399837 MHz
WDW EM
SSB 0
LB 0.20 Hz
GB 0
PC 1.00

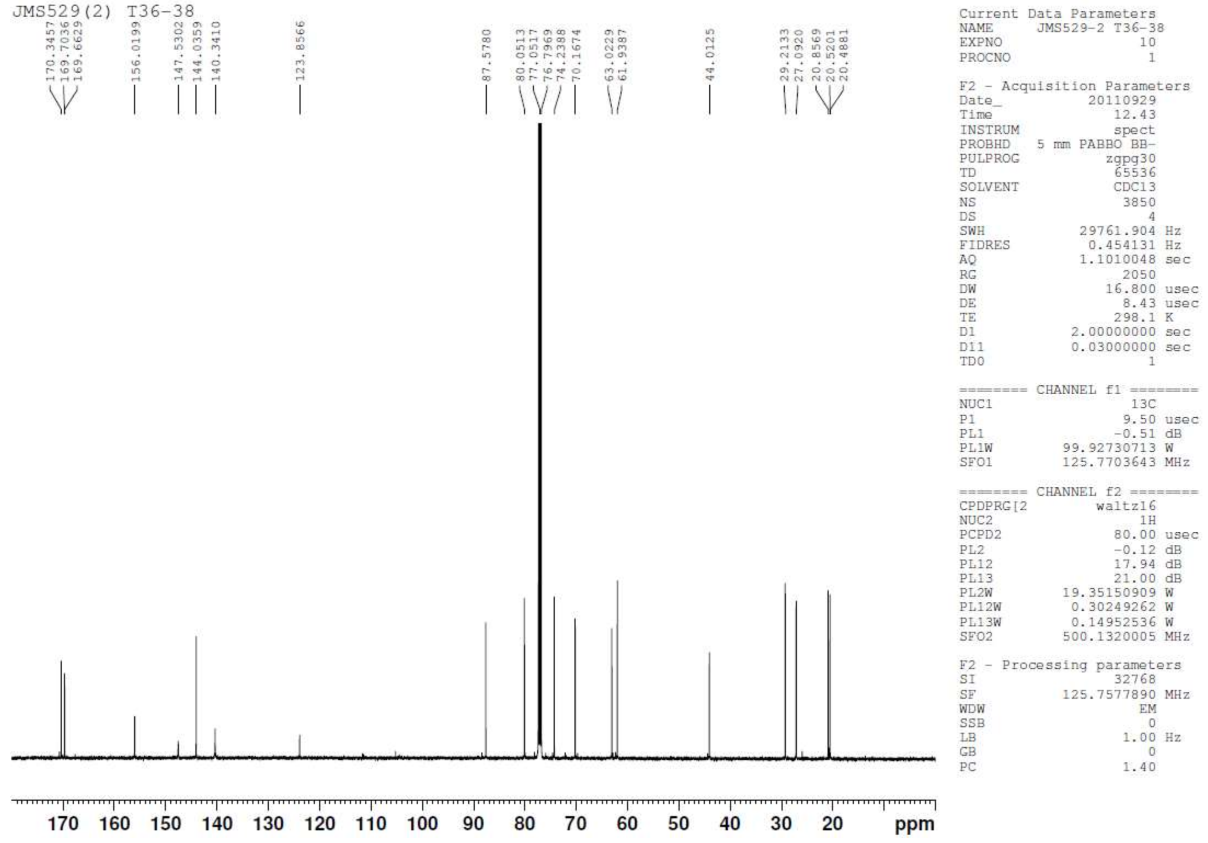
JMS529(2) T36-38
170.3457 169.7036 169.6629 156.0199 147.5302 144.0359 140.3410 123.8566 87.5780 80.0513 77.0517 76.7969 74.2388 70.1674 63.0229 61.9387 44.0125 29.2133 27.0920 20.8569 20.5201 20.4881
170 160 150 140 130 120 110 100 90 80 70 60 50 40 30 20 ppm
Current Data Parameters
NAME JMS529-2 T36-38
EXPNO 10
PROCNO 1
F2 - Acquisition Parameters
Date_ 20110929
Time 12.43
INSTRUM spect
PROBHD 5 mm PABBO BB-
PULPROG zgpg30
TD 65536
SOLVENT CDCl3
NS 3850
DS 4
SWH 29761.904 Hz
FIDRES 0.454131 Hz
AQ 1.1010048 sec
RG 2050
DW 16.800 usec
DE 8.43 usec
TE 298.1 K
D1 2.00000000 sec
D11 0.03000000 sec
TD0 1
======== CHANNEL f1 ========
NUC1 13C
P1 9.50 usec
PL1 -0.51 dB
PL1W 99.92730713 W
SFO1 125.7703643 MHz
======== CHANNEL f2 ========
CPDPRG[2 waltz16
NUC2 1H
PCPD2 80.00 usec
PL2 -0.12 dB
PL12 17.94 dB
PL13 21.00 dB
PL2W 19.35150909 W
PL12W 0.30249262 W
PL13W 0.14952536 W
SFO2 500.1320005 MHz
F2 - Processing parameters
SI 32768
SF 125.7577890 MHz
WDW EM
SSB 0
LB 1.00 Hz
GB 0
PC 1.40

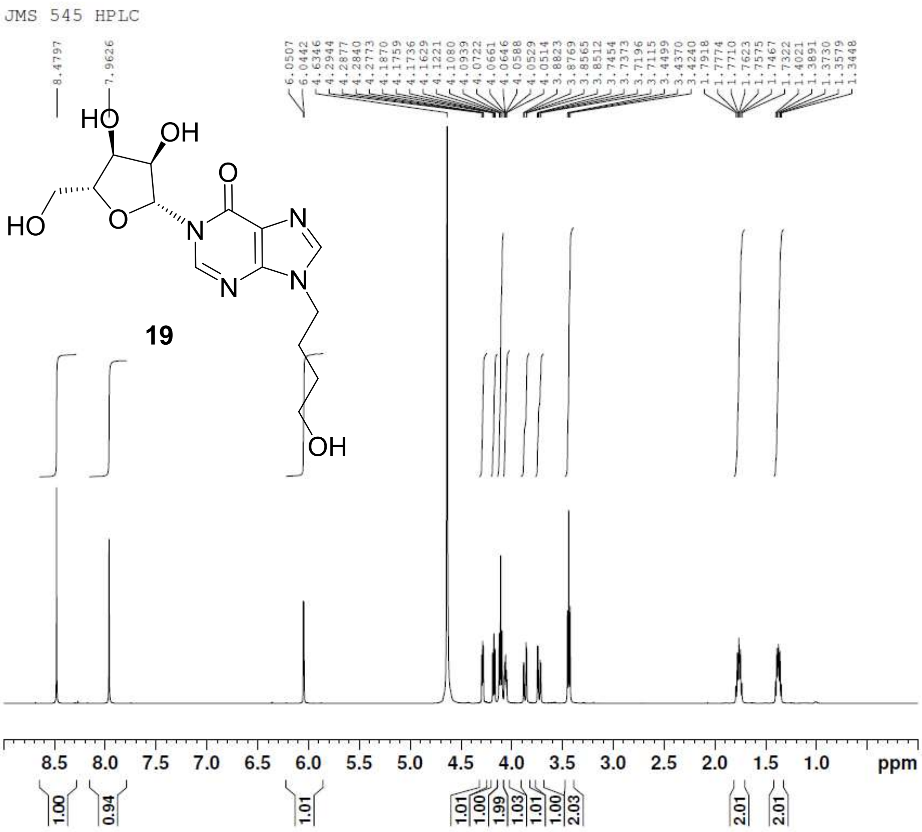

Current Data Parameters
NAME Oct24-2011-JMS545HPLC
EXPNO 10
PROCNO 1

F2 - Acquisition Parameters
Date_ 20111024
Time 19.16
INSTRUM spect
PROBHD 5 mm PABBO BB-
PULPROG zg30
TD 65536
SOLVENT D2O
NS 1600
DS 2
SWH 10330.578 Hz
FIDRES 0.157632 Hz
AQ 3.1719425 sec
RG 128
DW 48.400 usec
DE 13.94 usec
TE 297.9 K
D1 1.00000000 sec
TD0 1

======== CHANNEL f1 ========
NUC1 1H
P1 10.30 usec
PL1 -0.12 dB
PL1W 19.35150909 W
SFO1 500.1330885 MHz

F2 - Processing parameters
SI 32768
SF 500.1300345 MHz
WDW EM
SSB 0
LB 0.30 Hz
GB 0
PC 1.00

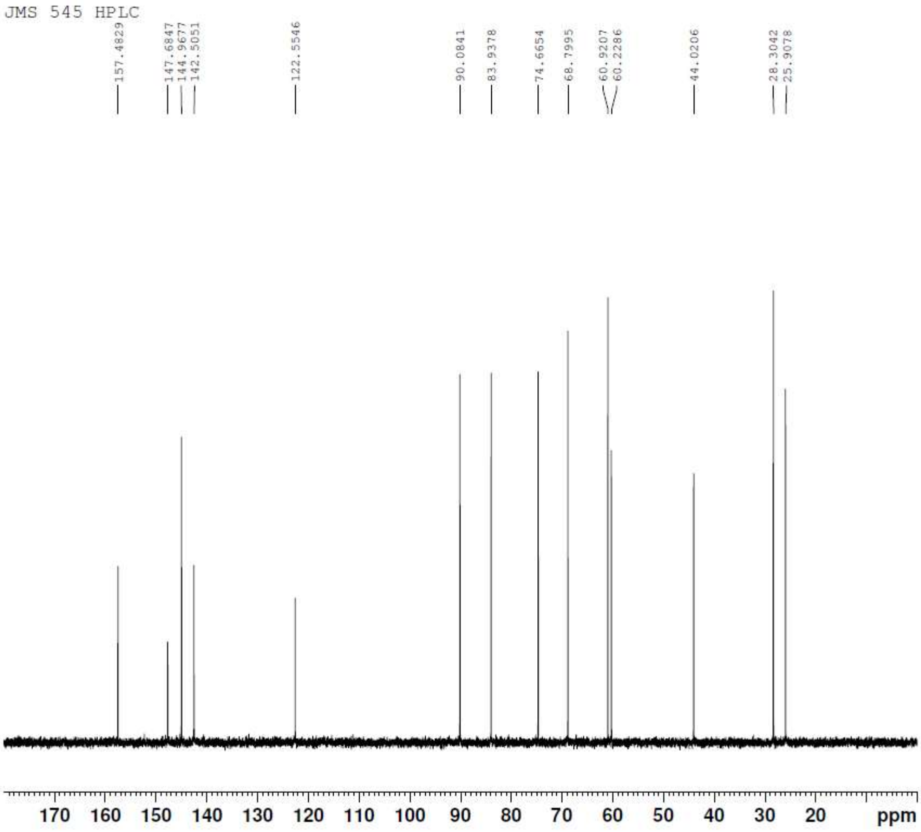

Current Data Parameters
NAME Oct24-2011-JMS545HPLC
EXPNO 11
PROCNO 1

F2 - Acquisition Parameters
Date_ 20111025
Time 6.10
INSTRUM spect
PROBHD 5 mm PABBO BB-
PULPROG zgpg30
TD 65536
SOLVENT D2O
NS 10240
DS 4
SWH 29761.904 Hz
FIDRES 0.454131 Hz
AQ 1.1010048 sec
RG 2050
DW 16.800 usec
DE 8.43 usec
TE 298.0 K
D1 2.00000000 sec
D11 0.03000000 sec
TD0 1

======== CHANNEL f1 ========
NUC1 13C
P1 9.50 usec
PL1 -0.51 dB
PL1W 99.92730713 W
SFO1 125.7703643 MHz

======== CHANNEL f2 ========
CPDPRG[2 waltz16
NUC2 1H
PCPD2 80.00 usec
PL2 -0.12 dB
PL12 17.94 dB
PL13 21.00 dB
PL2W 19.35150909 W
PL12W 0.30249262 W
PL13W 0.14952536 W
SFO2 500.1320005 MHz

F2 - Processing parameters
SI 32768
SF 125.7577890 MHz
WDW EM
SSB 0
LB 1.00 Hz
GB 0
PC 1.40

SAMPLE INFORMATION

Sample Name:	JMS 545 HPLC	Acquired By:	Christelle
Sample Type:	Unknown	Date Acquired:	25/10/2011 4:45:44 PM
Vial:	3	Acq. Method Set:	RP18 LC
Injection #:	1	Date Processed:	28/10/2011 12:25:15 PM
Injection Volume:	10.00 ul	Processing Method:	JMS 545 HPLC
Run Time:	20.0 Minutes	Channel Name:	WvlnCh1
Sample Set Name:	JMS 510 533 545 HPLC	Proc. Chnl. Descr.:	PDA 262.1 nm

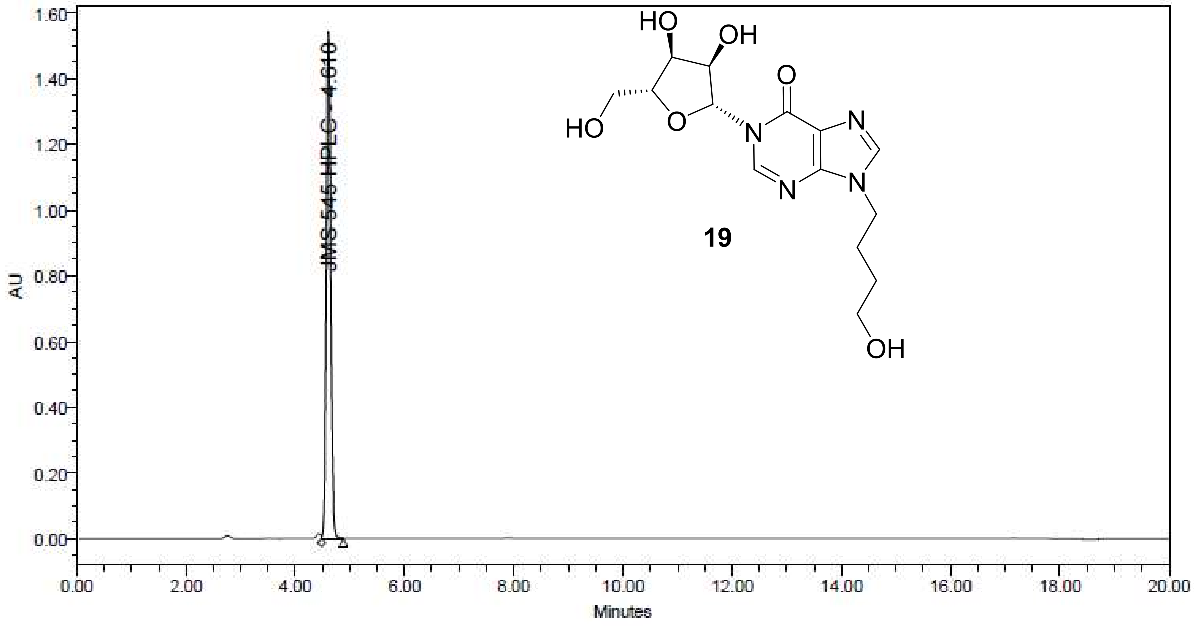

	Peak Name	RT	Area	% Area	Height
1	JMS 545 HPLC	4.610	8540932	100.00	1541489

JMS 522 T 8-10

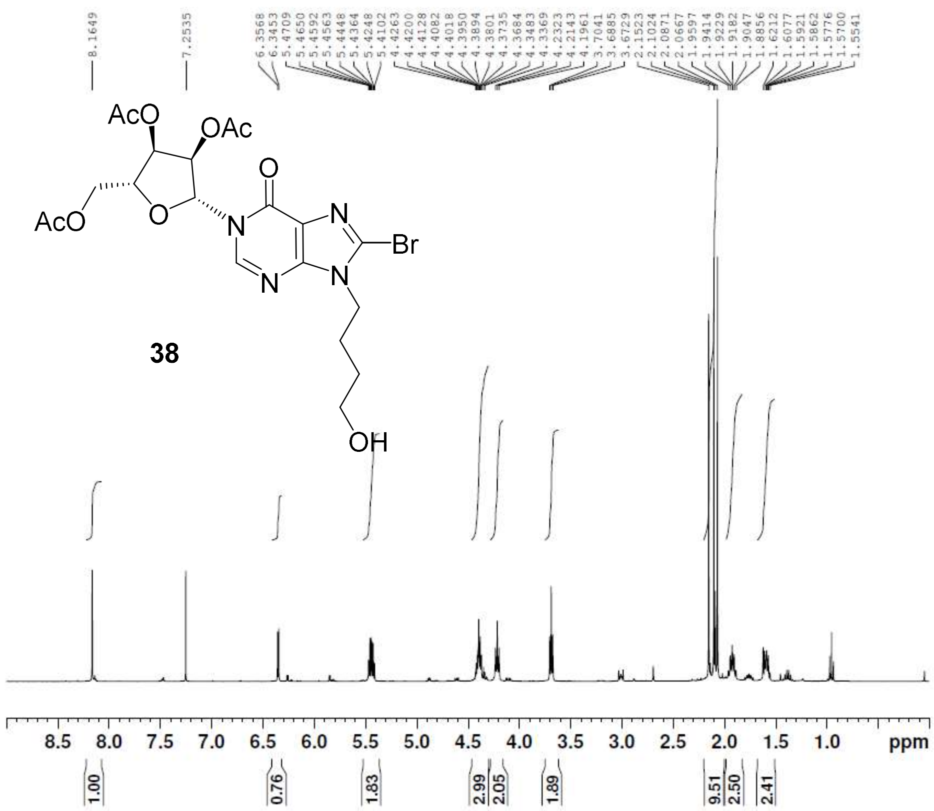

Current Data Parameters
NAME Aug08-2011-JMS11949
EXPNO 10
PROCNO 1

F2 - Acquisition Parameters
Date_ 20110809
Time 7.03
INSTRUM AVIII400
PROBHD 5 mm PABBO BB-
PULPROG zg30
TD 65536
SOLVENT CDCl3
NS 16
DS 2
SWH 8223.685 Hz
FIDRES 0.125483 Hz
AQ 3.9845889 sec
RG 128
DW 60.800 usec
DE 17.24 usec
TE 298.0 K
D1 1.00000000 sec
TD0 1

======== CHANNEL f1 ========
NUC1 1H
P1 13.00 usec
PL1 0 dB
PL1W 9.74611950 W
SFO1 400.0424704 MHz

F2 - Processing parameters
SI 65536
SF 400.0399837 MHz
WDW EM
SSB 0
LB 0.20 Hz
GB 0
PC 1.00

JMS 422 T 8-10

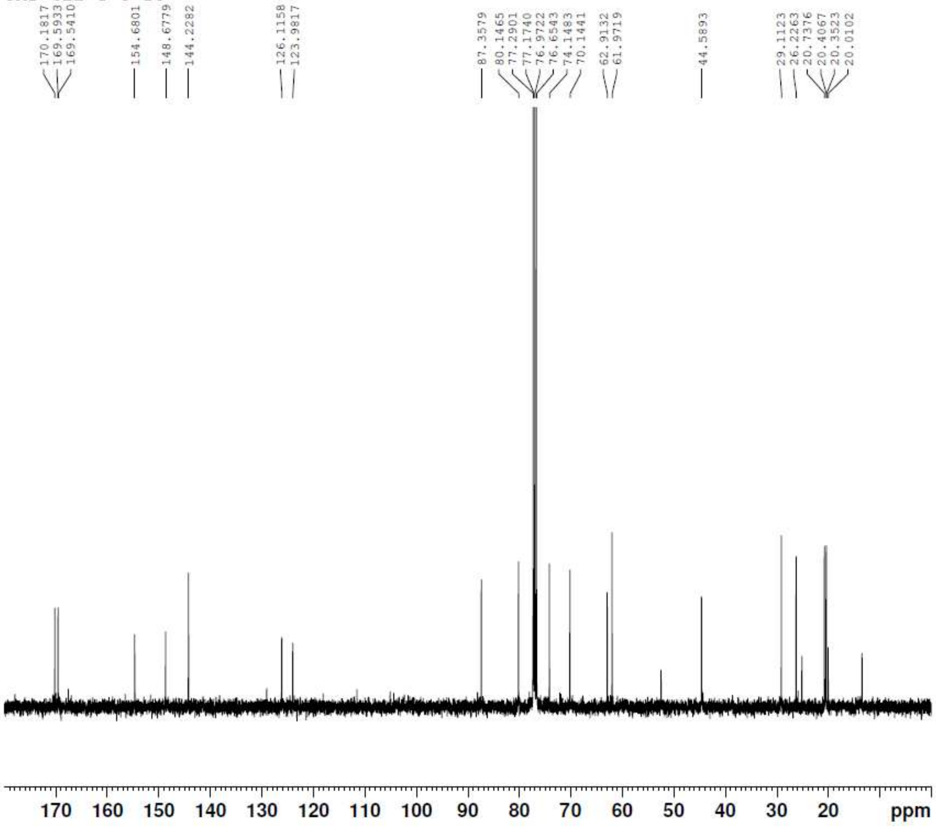

Current Data Parameters
NAME Aug08-2011-JMS11949
EXPNO 14
PROCNO 1

F2 - Acquisition Parameters
Date_ 20110809
Time 19.35
INSTRUM AVIII400
PROBHD 5 mm PABBO BB-
PULPROG zgpg30
TD 65536
SOLVENT CDCl3
NS 512
DS 4
SWH 24038.461 Hz
FIDRES 0.366798 Hz
AQ 1.3631488 sec
RG 2050
DW 20.800 usec
DE 6.50 usec
TE 298.0 K
D1 2.00000000 sec
D11 0.03000000 sec
TD0 1

======== CHANNEL f1 ========
NUC1 13C
P1 8.75 usec
PL1 -2.00 dB
PL1W 58.91986084 W
SFO1 100.6001970 MHz

======== CHANNEL f2 ========
CPDPRG[2 waltz16
NUC2 1H
PCPD2 80.00 usec
PL2 0 dB
PL12 15.78 dB
PL13 19.00 dB
PL2W 9.74611950 W
PL12W 0.25753233 W
PL13W 0.12269637 W
SFO2 400.0416002 MHz

F2 - Processing parameters
SI 65536
SF 100.5901380 MHz
WDW EM
SSB 0
LB 1.00 Hz
GB 0
PC 1.40

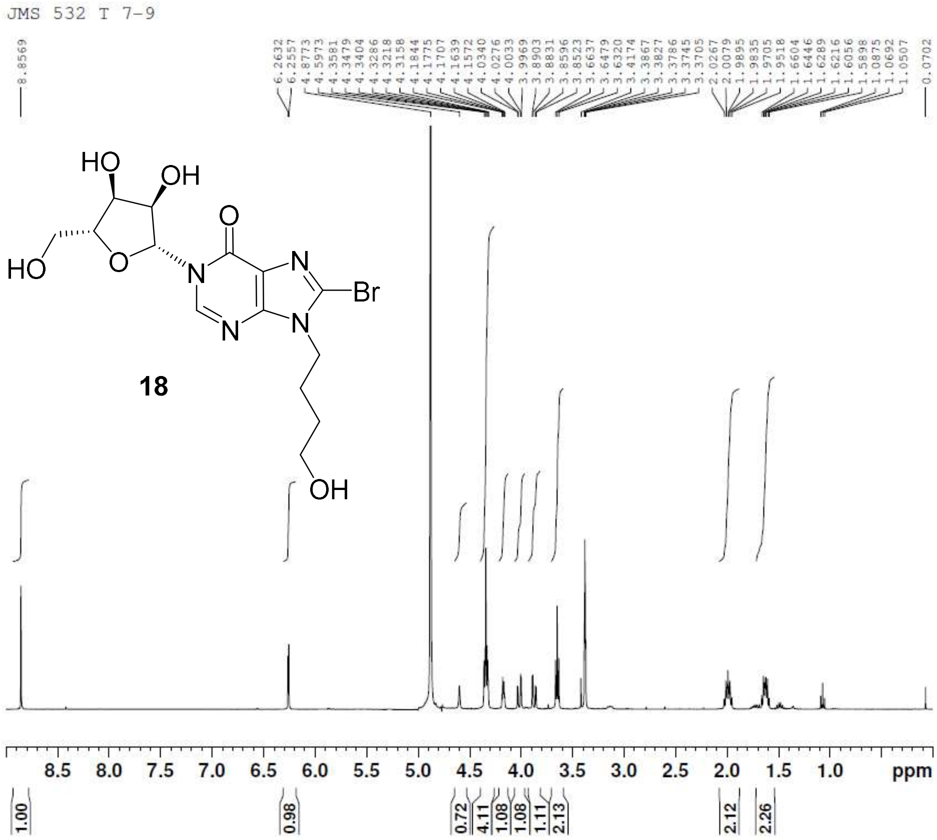

Current Data Parameters
NAME Aug12-2011-JMS10255
EXPNO 10
PROCNO 1

F2 - Acquisition Parameters
Date_ 20110812
Time 16.55
INSTRUM AVIII400
PROBHD 5 mm PABBO BB-
PULPROG zg30
TD 65536
SOLVENT MeOD
NS 16
DS 2
SWH 8223.685 Hz
FIDRES 0.125483 Hz
AQ 3.9845889 sec
RG 161
DW 60.800 usec
DE 17.24 usec
TE 298.0 K
D1 1.00000000 sec
TD0 1

======== CHANNEL f1 ========
NUC1 1H
P1 13.00 usec
PL1 0 dB
PL1W 9.74611950 W
SFO1 400.0424704 MHz

F2 - Processing parameters
SI 65536
SF 400.0399837 MHz
WDW EM
SSB 0
LB 0.20 Hz
GB 0
PC 1.00

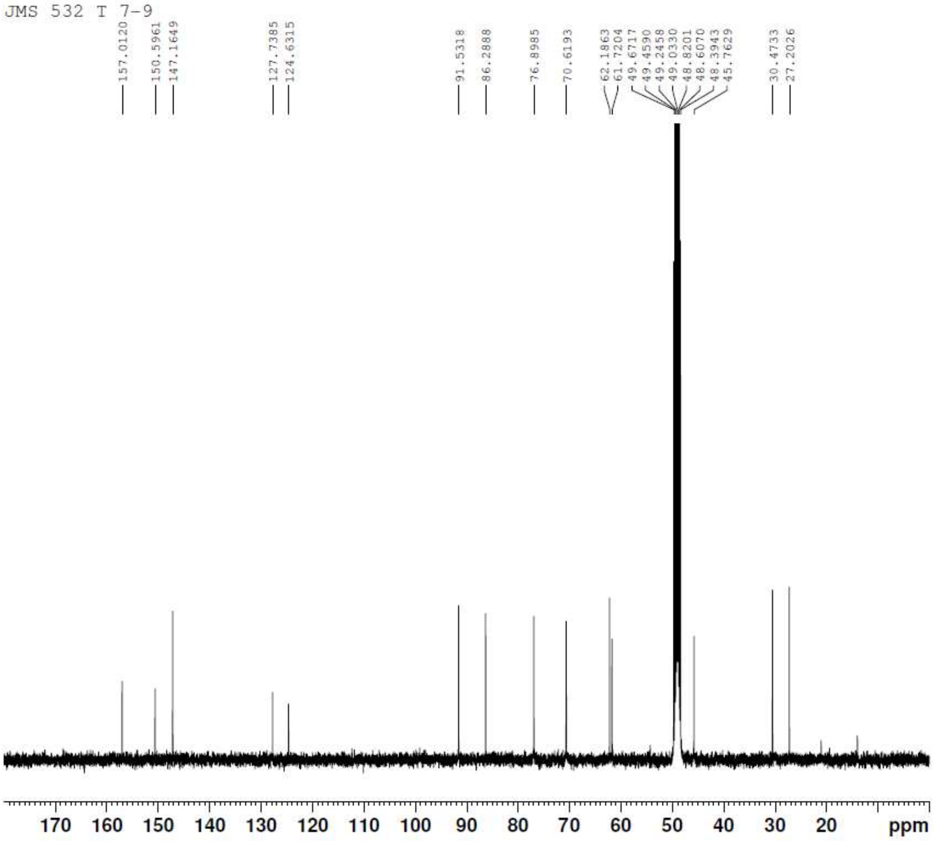

Current Data Parameters
NAME Aug12-2011-JMS10255
EXPNO 12
PROCNO 1

F2 - Acquisition Parameters
Date_ 20110813
Time 2.24
INSTRUM AVIII400
PROBHD 5 mm PABBO BB-
PULPROG zgpg30
TD 65536
SOLVENT MeOD
NS 2048
DS 4
SWH 24038.461 Hz
FIDRES 0.366798 Hz
AQ 1.3631488 sec
RG 1820
DW 20.800 usec
DE 6.50 usec
TE 298.0 K
D1 2.00000000 sec
D11 0.03000000 sec
TD0 1

======== CHANNEL f1 ========
NUC1 13C
P1 8.75 usec
PL1 -2.00 dB
PL1W 58.91986084 W
SFO1 100.6001970 MHz

======== CHANNEL f2 ========
CPDPRG[2 waltz16
NUC2 1H
PCPD2 80.00 usec
PL2 0 dB
PL12 15.78 dB
PL13 19.00 dB
PL2W 9.74611950 W
PL12W 0.25753233 W
PL13W 0.12269637 W
SFO2 400.0416002 MHz

F2 - Processing parameters
SI 65536
SF 100.5899952 MHz
WDW EM
SSB 0
LB 1.00 Hz
GB 0
PC 1.40

SAMPLE INFORMATION			
Sample Name:	JMS 532	Acquired By:	Joanna
Sample Type:	Unknown	Date Acquired:	18/08/2011 1:33:24 PM
Vial:	34	Acq. Method Set:	RP18 LC
Injection #:	1	Date Processed:	18/08/2011 5:00:47 PM
Injection Volume:	10.00 ul	Processing Method:	JMS 532
Run Time:	20.0 Minutes	Channel Name:	WvlnCh1
Sample Set Name:	JMS 509 510 519 532 533	Proc. Chnl. Descr.:	PDA 262.1 nm

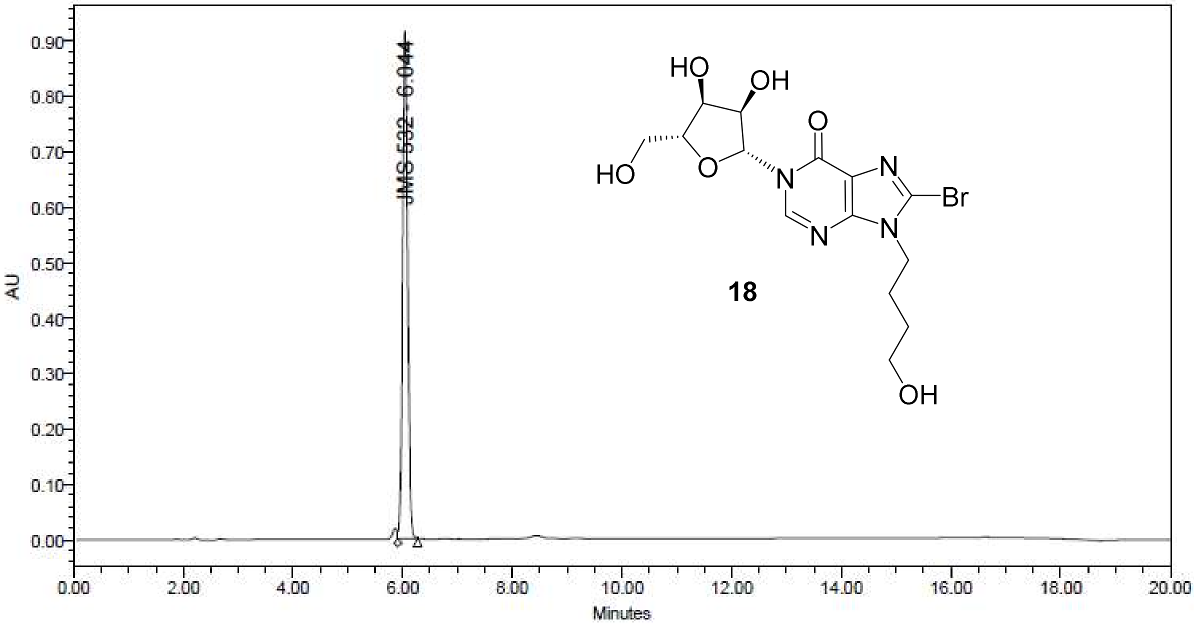

	Peak Name	RT	Area	% Area	Height
1	JMS 532	6.044	5950121	100.00	917702

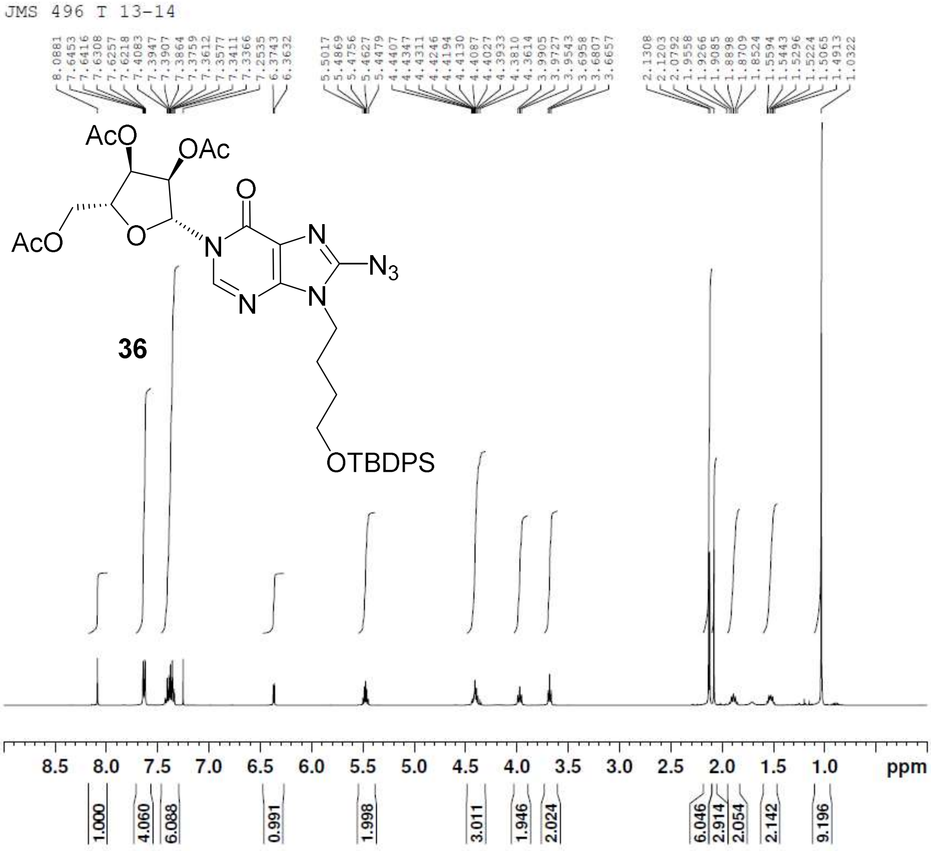

Current Data Parameters
NAME Aug03-2011-JMS11846
EXPNO 10
PROCNO 1

F2 - Acquisition Parameters
Date_ 20110803
Time 15.08
INSTRUM AVIII400
PROBHD 5 mm PABBO BB-
PULPROG zg30
TD 65536
SOLVENT CDCl3
NS 16
DS 2
SWH 8223.685 Hz
FIDRES 0.125483 Hz
AQ 3.9845889 sec
RG 101
DW 60.800 usec
DE 17.24 usec
TE 298.0 K
D1 1.00000000 sec
TD0 1

======== CHANNEL f1 ========
NUC1 1H
P1 13.00 usec
PL1 0 dB
PL1W 9.74611950 W
SFO1 400.0424704 MHz

F2 - Processing parameters
SI 65536
SF 400.0399837 MHz
WDW EM
SSB 0
LB 0.20 Hz
GB 0
PC 1.00

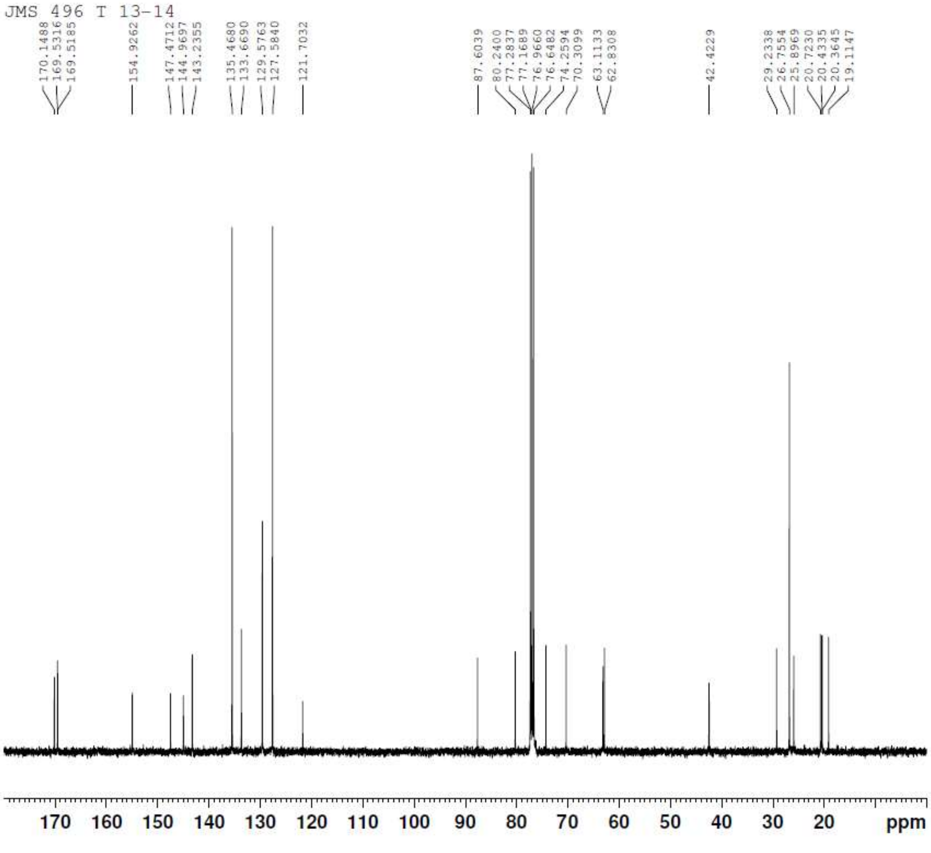

Current Data Parameters
NAME Aug03-2011-JMS11846
EXPNO 11
PROCNO 1

F2 - Acquisition Parameters
Date_ 20110803
Time 22.35
INSTRUM AVIII400
PROBHD 5 mm PABBO BB-
PULPROG zgpg30
TD 65536
SOLVENT CDCl3
NS 1024
DS 4
SWH 24038.461 Hz
FIDRES 0.366798 Hz
AQ 1.3631488 sec
RG 2050
DW 20.800 usec
DE 6.50 usec
TE 298.0 K
D1 2.00000000 sec
D11 0.03000000 sec
TD0 1

======== CHANNEL f1 ========
NUC1 13C
P1 8.75 usec
PL1 -2.00 dB
PL1W 58.91986084 W
SFO1 100.6001970 MHz

======== CHANNEL f2 ========
CPDPRG[2 waltz16
NUC2 1H
PCPD2 80.00 usec
PL2 0 dB
PL12 15.78 dB
PL13 19.00 dB
PL2W 9.74611950 W
PL12W 0.25753233 W
PL13W 0.12269637 W
SFO2 400.0416002 MHz

F2 - Processing parameters
SI 65536
SF 100.5901380 MHz
WDW EM
SSB 0
LB 1.00 Hz
GB 0
PC 1.40

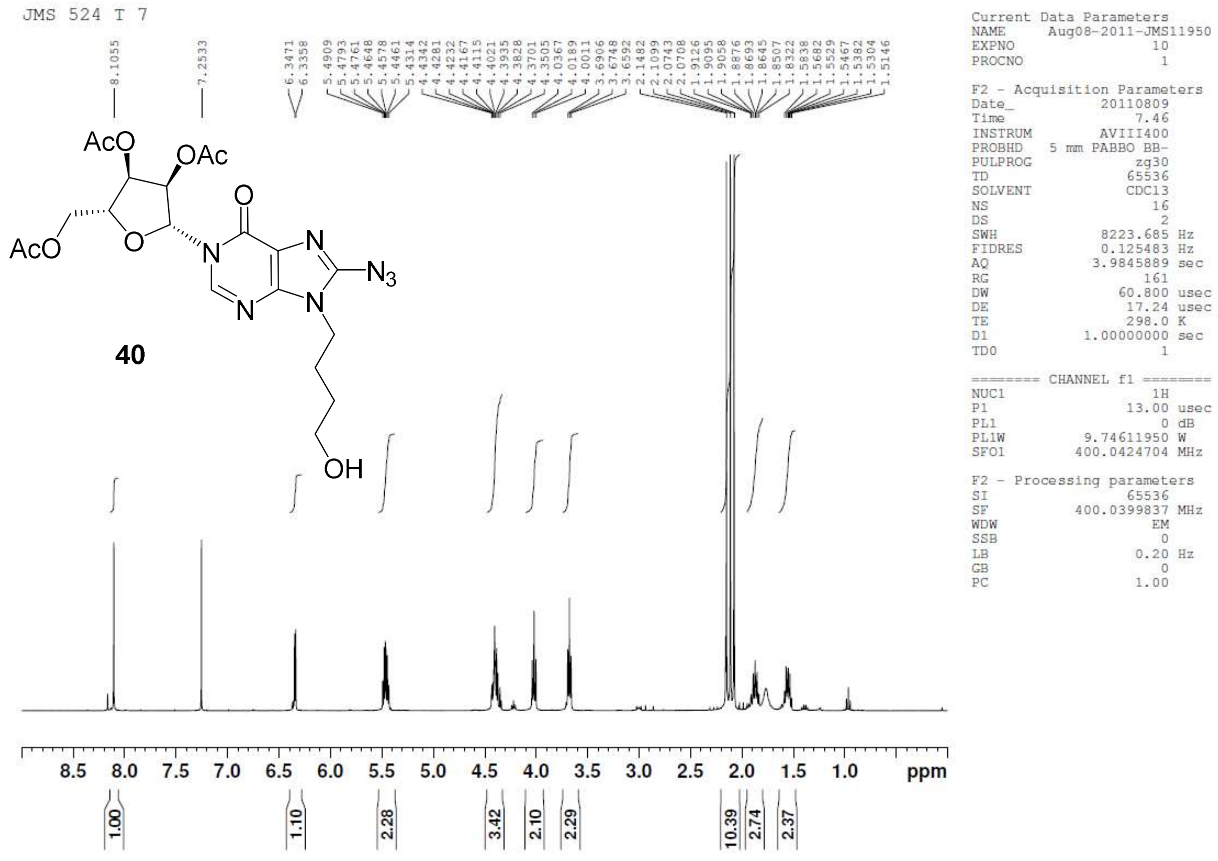
JMS 524 T 7
AcO
OAc
O
AcO
O
N
N
N
N
N3
OH
40
ppm
Current Data Parameters
NAME Aug08-2011-JMS11950
EXPNO 10
PROCNO 1
F2 - Acquisition Parameters
Date_ 20110809
Time 7.46
INSTRUM AVIII400
PROBHD 5 mm PABBO BB-
PULPROG zg30
TD 65536
SOLVENT CDCl3
NS 16
DS 2
SWH 8223.685 Hz
FIDRES 0.125483 Hz
AQ 3.9845889 sec
RG 161
DW 60.800 usec
DE 17.24 usec
TE 298.0 K
D1 1.00000000 sec
TD0 1
======== CHANNEL f1 ========
NUC1 1H
P1 13.00 usec
PL1 0 dB
PL1W 9.74611950 W
SFO1 400.0424704 MHz
F2 - Processing parameters
SI 65536
SF 400.0399837 MHz
WDW EM
SSB 0
LB 0.20 Hz
GB 0
PC 1.00

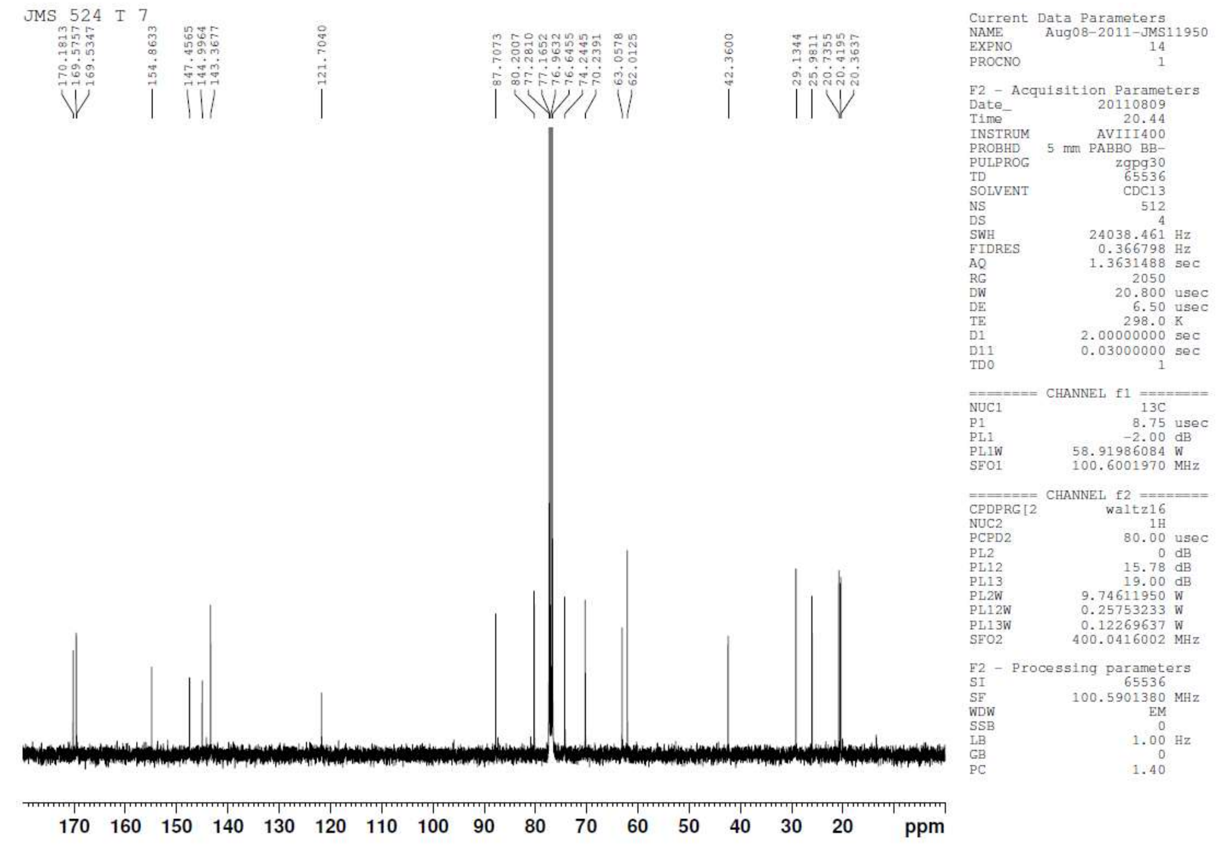
JMS 524 T 7
ppm
Current Data Parameters
NAME Aug08-2011-JMS11950
EXPNO 14
PROCNO 1
F2 - Acquisition Parameters
Date_ 20110809
Time 20.44
INSTRUM AVIII400
PROBHD 5 mm PABBO BB-
PULPROG zgpg30
TD 65536
SOLVENT CDCl3
NS 512
DS 4
SWH 24038.461 Hz
FIDRES 0.366798 Hz
AQ 1.3631488 sec
RG 2050
DW 20.800 usec
DE 6.50 usec
TE 298.0 K
D1 2.00000000 sec
D11 0.03000000 sec
TD0 1
======== CHANNEL f1 ========
NUC1 13C
P1 8.75 usec
PL1 -2.00 dB
PL1W 58.91986084 W
SFO1 100.6001970 MHz
======== CHANNEL f2 ========
CPDPRG[2 waltz16
NUC2 1H
PCPD2 80.00 usec
PL2 0 dB
PL12 15.78 dB
PL13 19.00 dB
PL2W 9.74611950 W
PL12W 0.25753233 W
PL13W 0.12269637 W
SFO2 400.0416002 MHz
F2 - Processing parameters
SI 65536
SF 100.5901380 MHz
WDW EM
SSB 0
LB 1.00 Hz
GB 0
PC 1.40

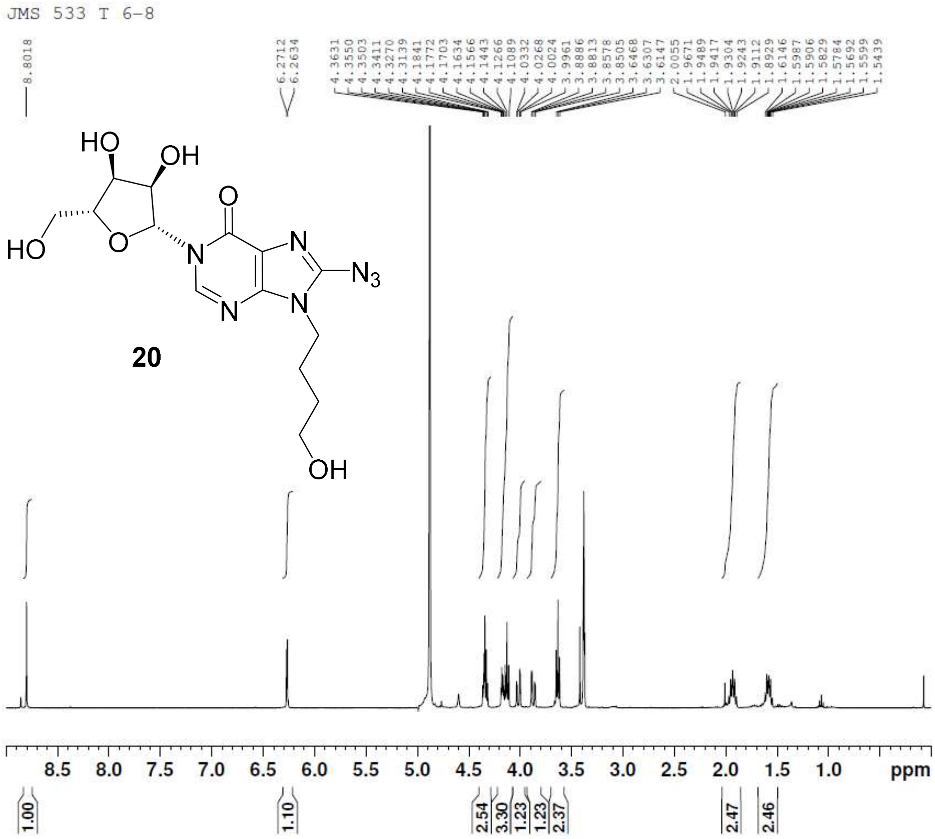


```
Current Data Parameters
NAME      Aug12-2011-JMS10256
EXPNO                    10
PROCNO                    1

F2 - Acquisition Parameters
Date_              20110812
Time                  17.06
INSTRUM            AVIII400
PROBHD     5 mm PABBO BB-
PULPROG                zg30
TD                    65536
SOLVENT                MeOD
NS                       16
DS                        2
SWH                8223.685 Hz
FIDRES             0.125483 Hz
AQ                3.9845889 sec
RG                      181
DW                   60.800 usec
DE                    17.24 usec
TE                    298.0 K
D1               1.00000000 sec
TD0                       1

======== CHANNEL f1 ========
NUC1                     1H
P1                    13.00 usec
PL1                       0 dB
PL1W             9.74611950 W
SFO1            400.0424704 MHz

F2 - Processing parameters
SI                    65536
SF              400.0399837 MHz
WDW                      EM
SSB                       0
LB                     0.20 Hz
GB                        0
PC                     1.00
```

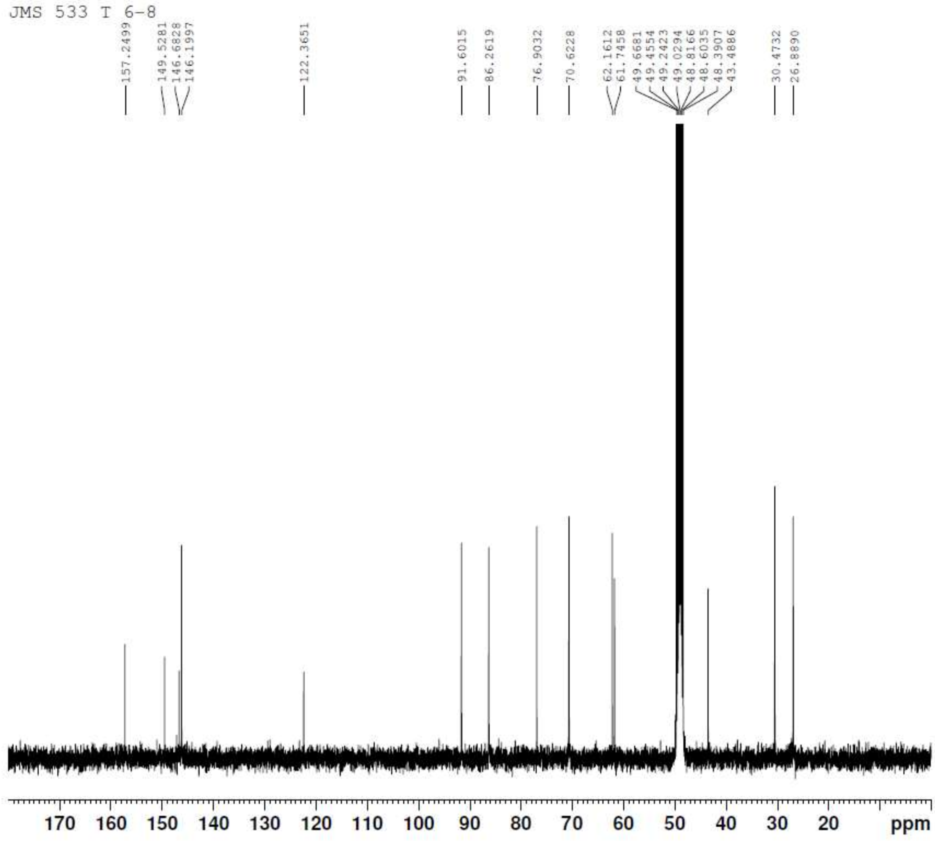


```
Current Data Parameters
NAME      Aug12-2011-JMS10256
EXPNO                    12
PROCNO                    1

F2 - Acquisition Parameters
Date_              20110813
Time                   5.33
INSTRUM            AVIII400
PROBHD     5 mm PABBO BB-
PULPROG              zgpg30
TD                    65536
SOLVENT                MeOD
NS                     2048
DS                        4
SWH               24038.461 Hz
FIDRES             0.366798 Hz
AQ                1.3631488 sec
RG                     2050
DW                   20.800 usec
DE                     6.50 usec
TE                    298.0 K
D1               2.00000000 sec
D11              0.03000000 sec
TD0                       1

======== CHANNEL f1 ========
NUC1                    13C
P1                     8.75 usec
PL1                   -2.00 dB
PL1W            58.91986084 W
SFO1            100.6001970 MHz

======== CHANNEL f2 ========
CPDPRG[2                waltz16
NUC2                     1H
PCPD2                 80.00 usec
PL2                       0 dB
PL12                  15.78 dB
PL13                  19.00 dB
PL2W             9.74611950 W
PL12W            0.25753233 W
PL13W            0.12269637 W
SFO2            400.0416002 MHz

F2 - Processing parameters
SI                    65536
SF              100.5899952 MHz
WDW                      EM
SSB                       0
LB                     1.00 Hz
GB                        0
PC                     1.40
```

SAMPLE INFORMATION

Sample Name:	JMS 533 HPLC	Acquired By:	Christelle
Sample Type:	Unknown	Date Acquired:	25/10/2011 4:24:49 PM
Vial:	2	Acq. Method Set:	RP18 LC
Injection #:	1	Date Processed:	28/10/2011 12:23:30 PM
Injection Volume:	10.00 ul	Processing Method:	JMS 533 HPLC
Run Time:	20.0 Minutes	Channel Name:	WvlnCh1
Sample Set Name:	JMS 510 533 545 HPLC	Proc. Chnl. Descr.:	PDA 262.1 nm

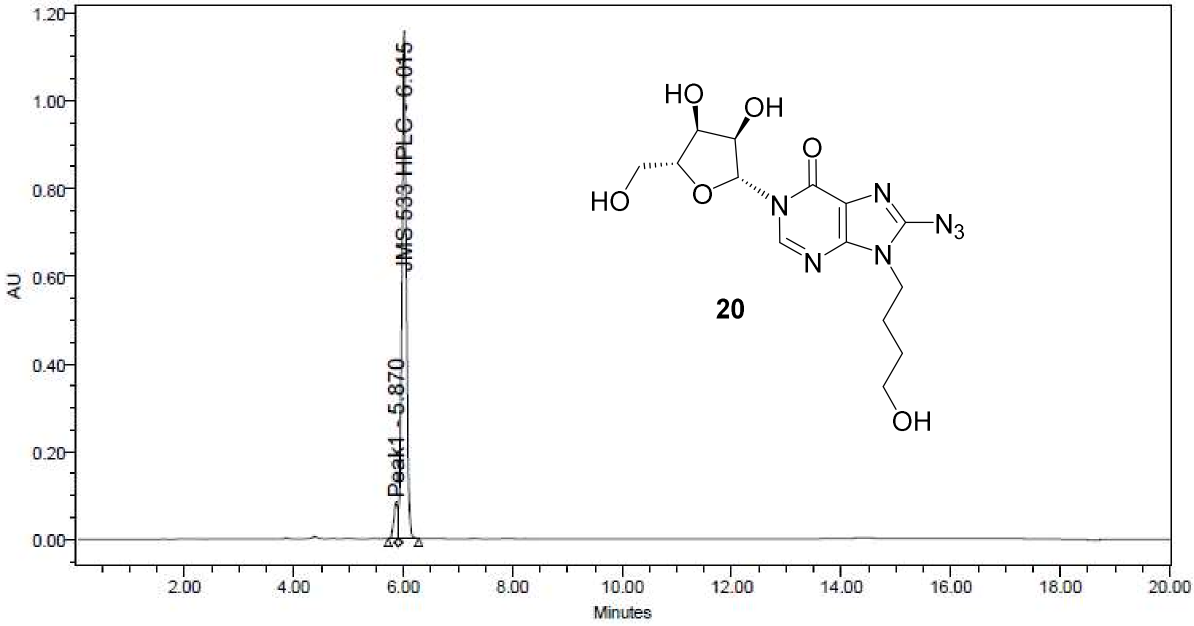

20

	Peak Name	RT	Area	% Area	Height
1	Peak1	5.870	424215	5.84	83250
2	JMS 533 HPLC	6.015	6835625	94.16	1157681

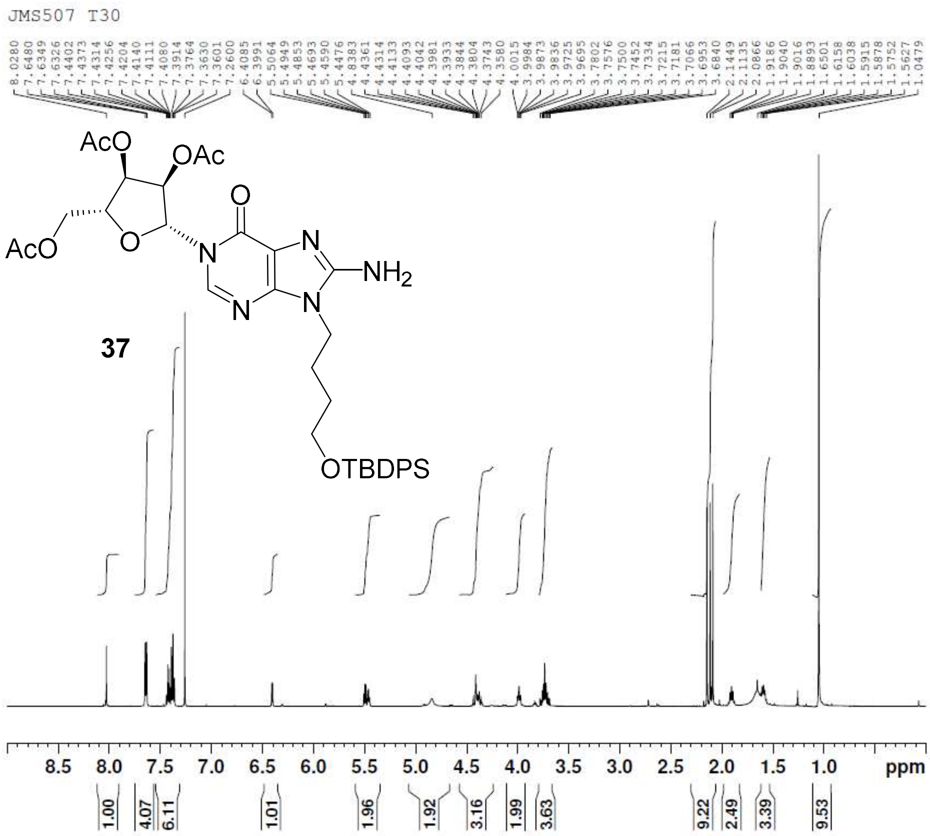

Current Data Parameters
NAME JMS507 T30
EXPNO 10
PROCNO 1

F2 - Acquisition Parameters
Date_ 20110802
Time 19.47
INSTRUM spect
PROBHD 5 mm PABBO BB-
PULPROG zg30
TD 65536
SOLVENT CDCl3
NS 16
DS 2
SWH 10330.578 Hz
FIDRES 0.157632 Hz
AQ 3.1719425 sec
RG 256
DW 48.400 usec
DE 13.94 usec
TE 297.9 K
D1 1.00000000 sec
TD0 1

======== CHANNEL f1 ========
NUC1 1H
P1 10.30 usec
PL1 -0.12 dB
PL1W 19.35150909 W
SFO1 500.1330885 MHz

F2 - Processing parameters
SI 32768
SF 500.1300132 MHz
WDW EM
SSB 0
LB 0.30 Hz
GB 0
PC 1.00

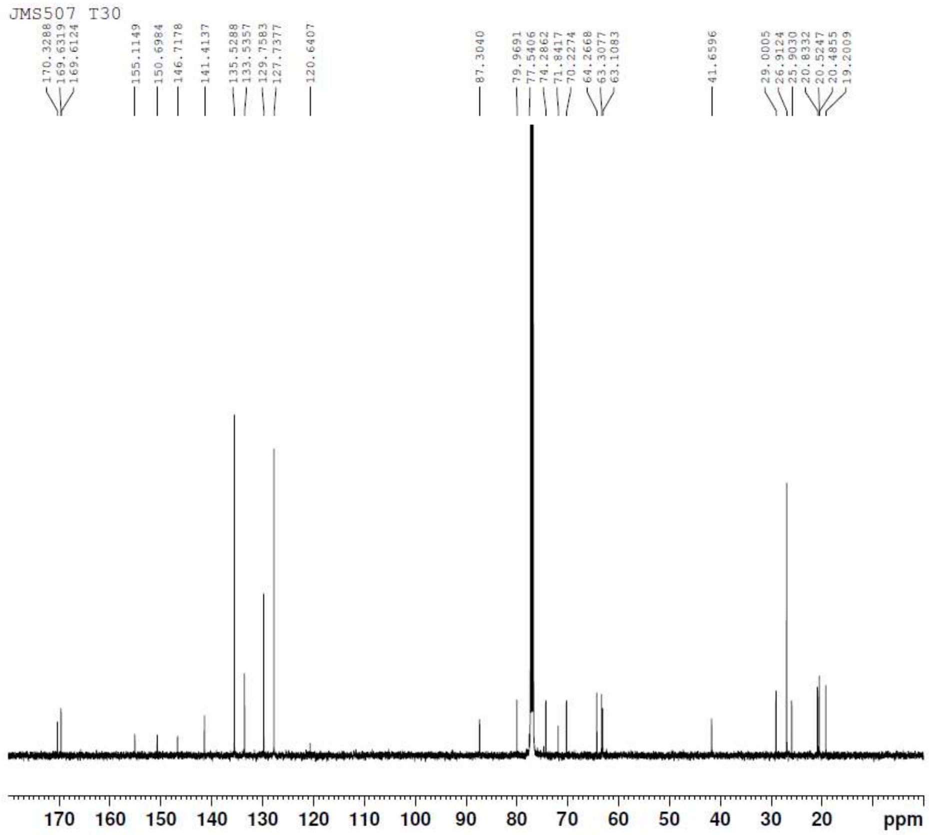

Current Data Parameters
NAME JMS507 T30
EXPNO 11
PROCNO 1

F2 - Acquisition Parameters
Date_ 20110803
Time 4.50
INSTRUM spect
PROBHD 5 mm PABBO BB-
PULPROG zgpg30
TD 65536
SOLVENT CDCl3
NS 10240
DS 4
SWH 29761.904 Hz
FIDRES 0.454131 Hz
AQ 1.1010048 sec
RG 2050
DW 16.800 usec
DE 8.43 usec
TE 298.0 K
D1 2.00000000 sec
D11 0.03000000 sec
TD0 1

======== CHANNEL f1 ========
NUC1 13C
P1 9.50 usec
PL1 -0.51 dB
PL1W 99.92730713 W
SFO1 125.7703643 MHz

======== CHANNEL f2 ========
CPDPRG[2 waltz16
NUC2 1H
PCPD2 80.00 usec
PL2 -0.12 dB
PL12 17.94 dB
PL13 21.00 dB
PL2W 19.35150909 W
PL12W 0.30249262 W
PL13W 0.14952536 W
SFO2 500.1320005 MHz

F2 - Processing parameters
SI 32768
SF 125.7577905 MHz
WDW EM
SSB 0
LB 1.00 Hz
GB 0
PC 1.40

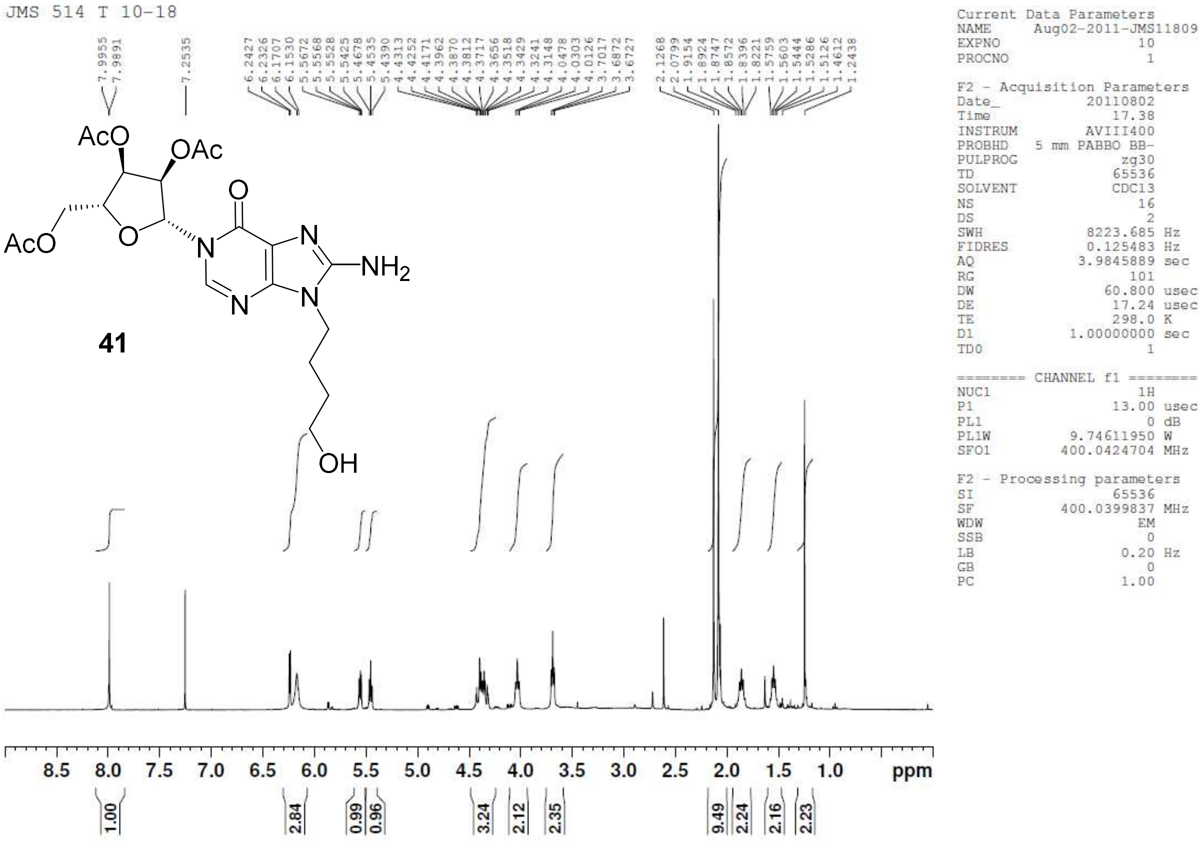
JMS 514 T 10-18
7.9955
7.9891
7.2535
6.2427 6.2326 6.1707 6.1530 5.5672 5.5568 5.5528 5.5425 5.4678 5.4535 5.4390 4.4313 4.4252 4.4171 4.3962 4.3870 4.3812 4.3717 4.3656 4.3518 4.3429 4.3241 4.3148 4.0478 4.0303 4.0126 3.7017 3.6872 3.6727
2.1268 2.0799 1.9154 1.8924 1.8747 1.8572 1.8396 1.8221 1.5759 1.5603 1.5444 1.5286 1.5126 1.4612 1.2438
AcO
OAc
O
N
NH2
AcO
O
N
N
N
41
OH
Current Data Parameters
NAME Aug02-2011-JMS11809
EXPNO 10
PROCNO 1
F2 - Acquisition Parameters
Date_ 20110802
Time 17.38
INSTRUM AVIII400
PROBHD 5 mm PABBO BB-
PULPROG zg30
TD 65536
SOLVENT CDCl3
NS 16
DS 2
SWH 8223.685 Hz
FIDRES 0.125483 Hz
AQ 3.9845889 sec
RG 101
DW 60.800 usec
DE 17.24 usec
TE 298.0 K
D1 1.00000000 sec
TD0 1
======== CHANNEL f1 ========
NUC1 1H
P1 13.00 usec
PL1 0 dB
PL1W 9.74611950 W
SFO1 400.0424704 MHz
F2 - Processing parameters
SI 65536
SF 400.0399837 MHz
WDW EM
SSB 0
LB 0.20 Hz
GB 0
PC 1.00
8.5 8.0 7.5 7.0 6.5 6.0 5.5 5.0 4.5 4.0 3.5 3.0 2.5 2.0 1.5 1.0 ppm
1.00
2.84
0.99
0.96
3.24
2.12
2.35
9.49
2.24
2.16
2.23

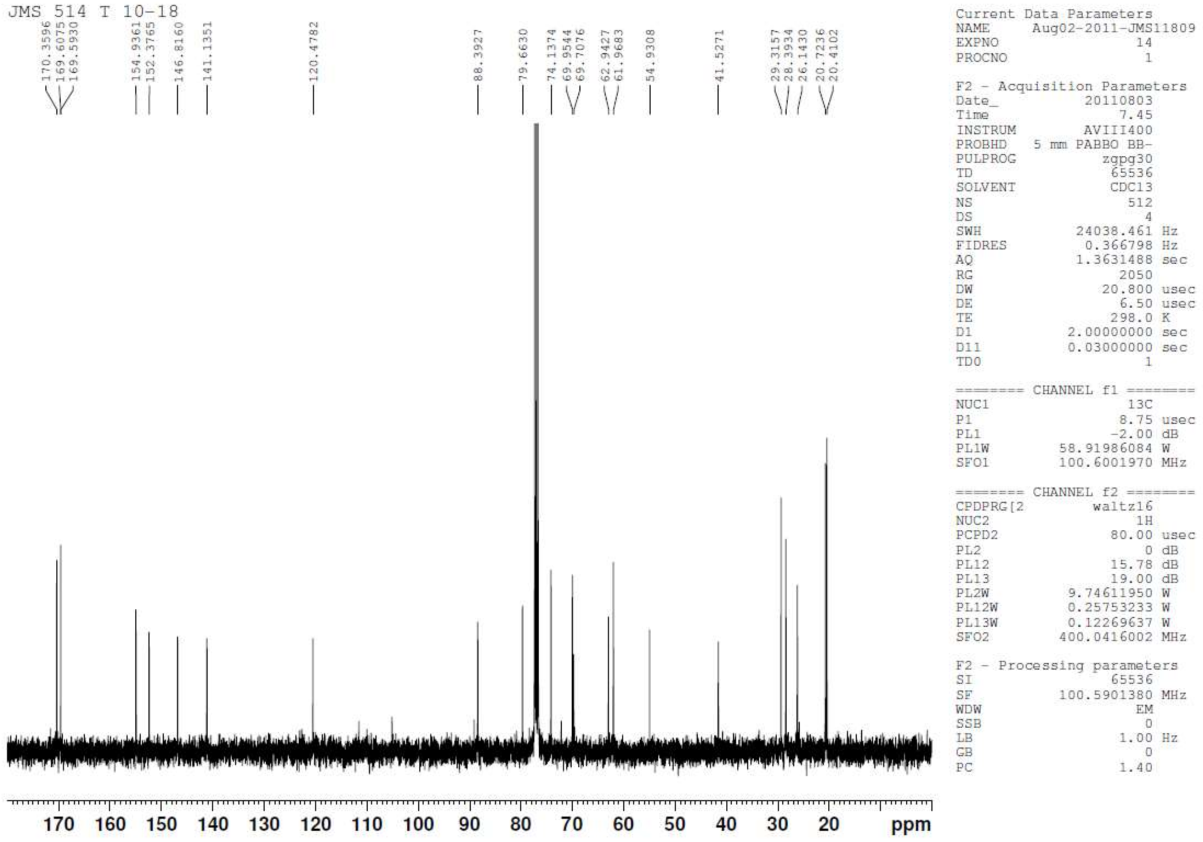
JMS 514 T 10-18
170.3596 169.6075 169.5930
154.9361 152.3765 146.8160 141.1351
120.4782
88.3927
79.6630 74.1374 69.9544 69.7076 62.9427 61.9683 54.9308
41.5271
29.3157 28.3934 26.1430 20.7236 20.4102
Current Data Parameters
NAME Aug02-2011-JMS11809
EXPNO 14
PROCNO 1
F2 - Acquisition Parameters
Date_ 20110803
Time 7.45
INSTRUM AVIII400
PROBHD 5 mm PABBO BB-
PULPROG zgpg30
TD 65536
SOLVENT CDCl3
NS 512
DS 4
SWH 24038.461 Hz
FIDRES 0.366798 Hz
AQ 1.3631488 sec
RG 2050
DW 20.800 usec
DE 6.50 usec
TE 298.0 K
D1 2.00000000 sec
D11 0.03000000 sec
TD0 1
======== CHANNEL f1 ========
NUC1 13C
P1 8.75 usec
PL1 -2.00 dB
PL1W 58.91986084 W
SFO1 100.6001970 MHz
======== CHANNEL f2 ========
CPDPRG[2 waltz16
NUC2 1H
PCPD2 80.00 usec
PL2 0 dB
PL12 15.78 dB
PL13 19.00 dB
PL2W 9.74611950 W
PL12W 0.25753233 W
PL13W 0.12269637 W
SFO2 400.0416002 MHz
F2 - Processing parameters
SI 65536
SF 100.5901380 MHz
WDW EM
SSB 0
LB 1.00 Hz
GB 0
PC 1.40
170 160 150 140 130 120 110 100 90 80 70 60 50 40 30 20 ppm

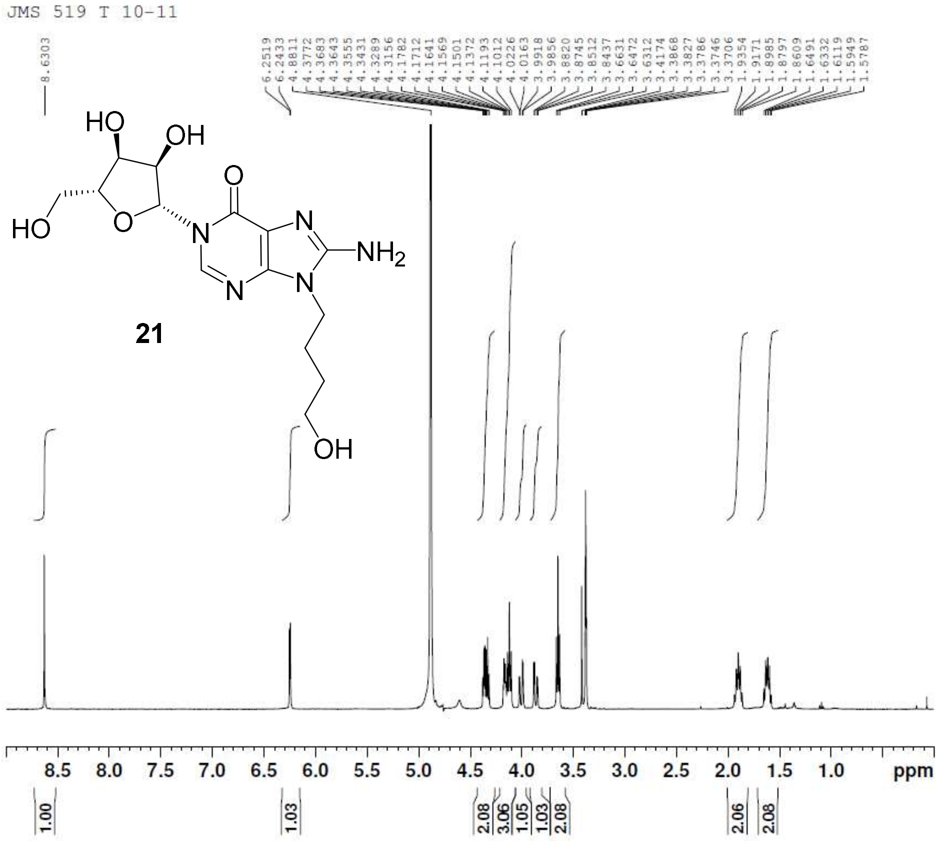


```
Current Data Parameters
NAME      Aug08-2011-JMS11948
EXPNO                   10
PROCNO                   1

F2 - Acquisition Parameters
Date_             20110809
Time                  8.02
INSTRUM           AVIII400
PROBHD   5 mm PABBO BB-
PULPROG               zg30
TD                   65536
SOLVENT               MeOD
NS                      16
DS                       2
SWH            8223.685 Hz
FIDRES         0.125483 Hz
AQ            3.9845889 sec
RG                     144
DW              60.800 usec
DE               17.24 usec
TE               298.0 K
D1          1.00000000 sec
TD0                      1

======== CHANNEL f1 ========
NUC1                    1H
P1              13.00 usec
PL1                   0 dB
PL1W        9.74611950 W
SFO1      400.0424704 MHz

F2 - Processing parameters
SI                   65536
SF        400.0399837 MHz
WDW                     EM
SSB                      0
LB                 0.20 Hz
GB                       0
PC                    1.00
```

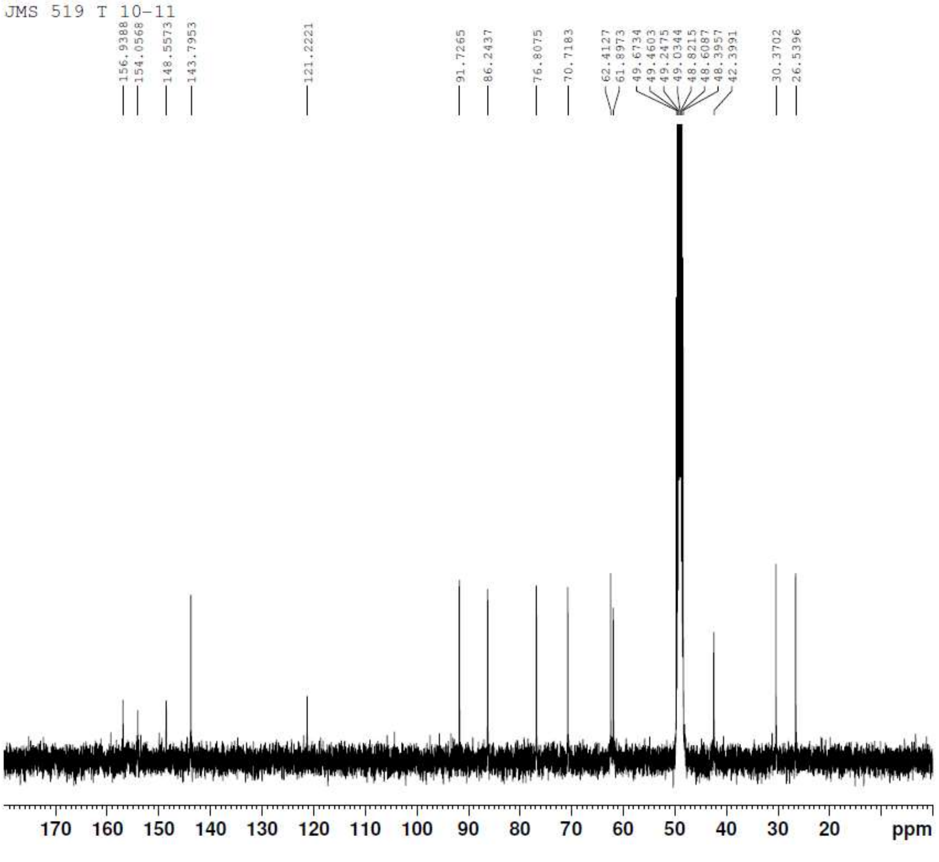


```
Current Data Parameters
NAME      Aug08-2011-JMS11948
EXPNO                   11
PROCNO                   1

F2 - Acquisition Parameters
Date_             20110809
Time                 22.22
INSTRUM           AVIII400
PROBHD   5 mm PABBO BB-
PULPROG             zgpg30
TD                   65536
SOLVENT               MeOD
NS                    1024
DS                       4
SWH           24038.461 Hz
FIDRES         0.366798 Hz
AQ            1.3631488 sec
RG                    1820
DW              20.800 usec
DE                6.50 usec
TE               298.0 K
D1          2.00000000 sec
D11         0.03000000 sec
TD0                      1

======== CHANNEL f1 ========
NUC1                   13C
P1               8.75 usec
PL1               -2.00 dB
PL1W       58.91986084 W
SFO1      100.6001970 MHz

======== CHANNEL f2 ========
CPDPRG[2           waltz16
NUC2                    1H
PCPD2           80.00 usec
PL2                   0 dB
PL12              15.78 dB
PL13              19.00 dB
PL2W        9.74611950 W
PL12W       0.25753233 W
PL13W       0.12269637 W
SFO2      400.0416602 MHz

F2 - Processing parameters
SI                   65536
SF        100.5899952 MHz
WDW                     EM
SSB                      0
LB                 1.00 Hz
GB                       0
PC                    1.40
```

SAMPLE INFORMATION			
Sample Name:	JMS 519	Acquired By:	Joanna
Sample Type:	Unknown	Date Acquired:	18/08/2011 1:12:30 PM
Vial:	33	Acq. Method Set:	RP18 LC
Injection #:	1	Date Processed:	18/08/2011 4:35:57 PM
Injection Volume:	10.00 ul	Processing Method:	JMS 519
Run Time:	20.0 Minutes	Channel Name:	WvlnCh1
Sample Set Name:	JMS 509 510 519 532 533	Proc. Chnl. Descr.:	PDA 262.1 nm

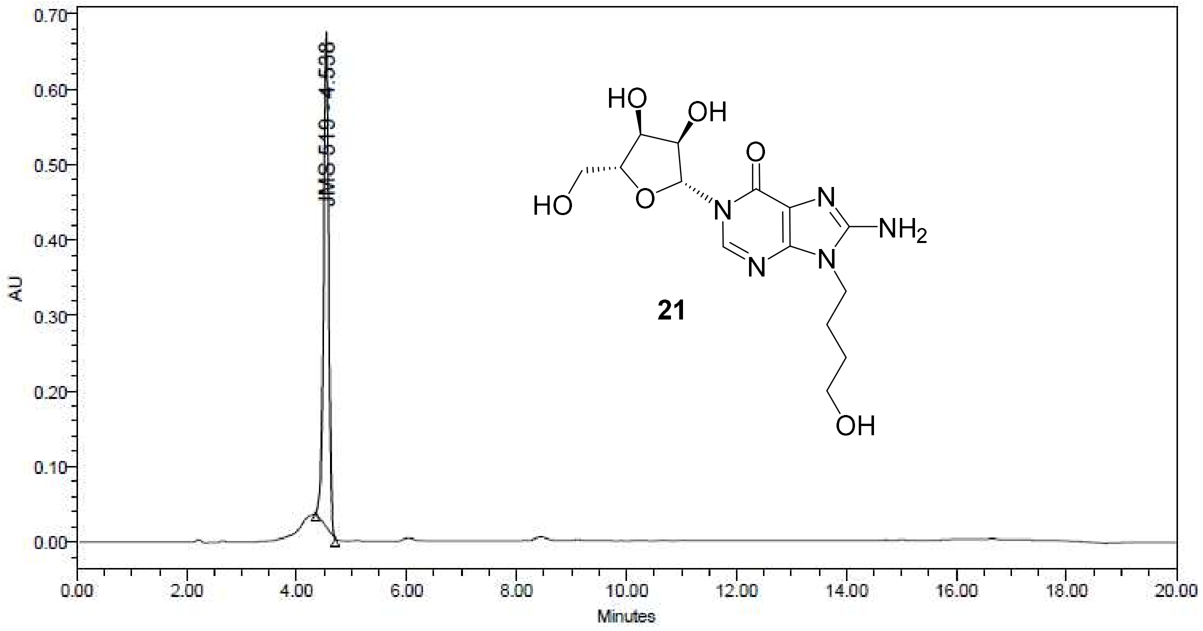

	Peak Name	RT	Area	% Area	Height
1	JMS 519	4.538	4357041	100.00	656981

<u>**Total synthesis of *N*9-(4-hydroxybutyl)-*N*1-IMP analogues (22-25)**</u>

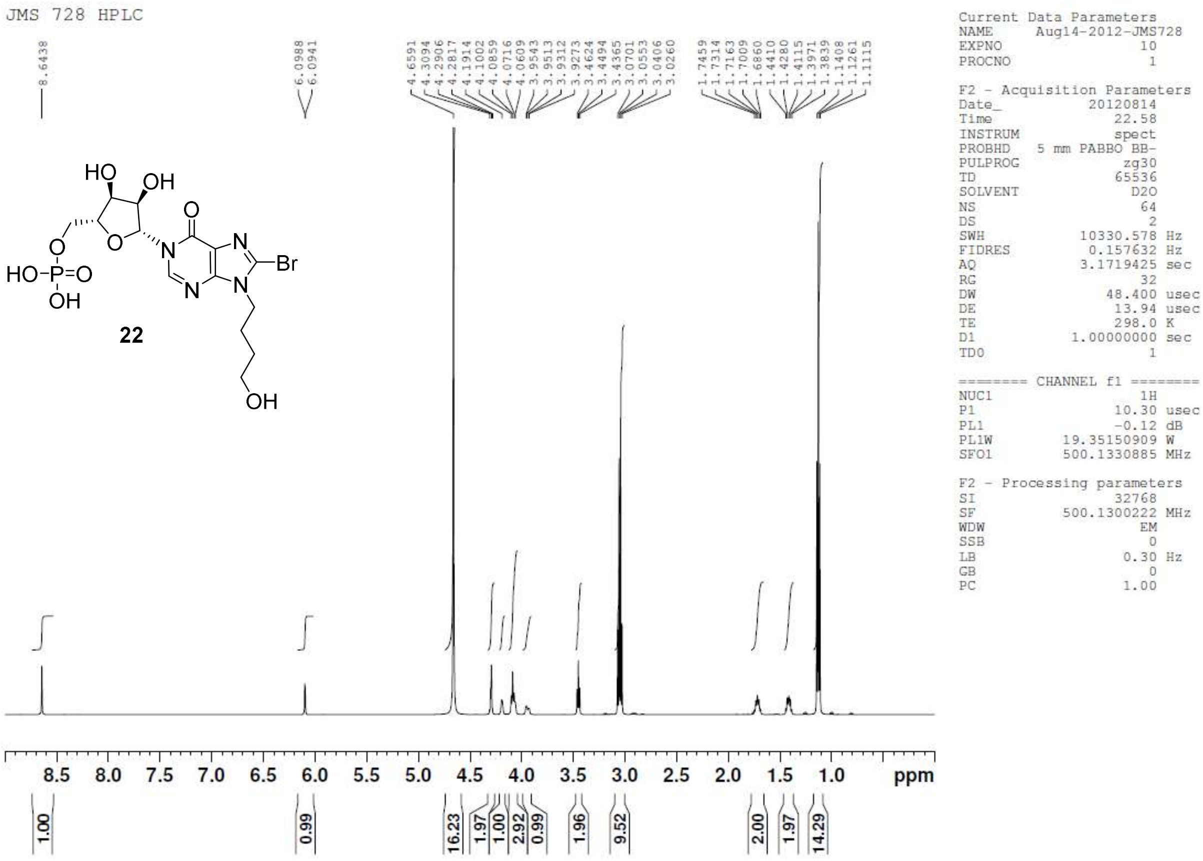

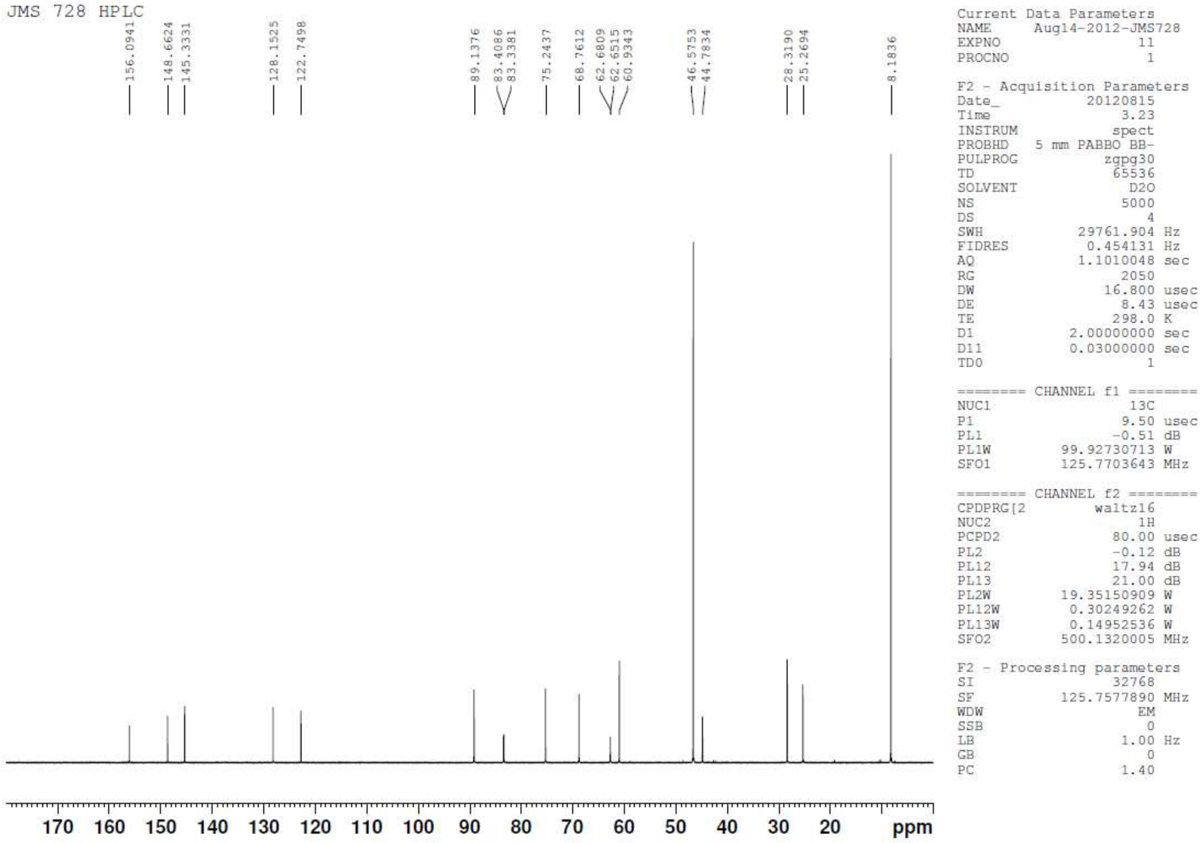

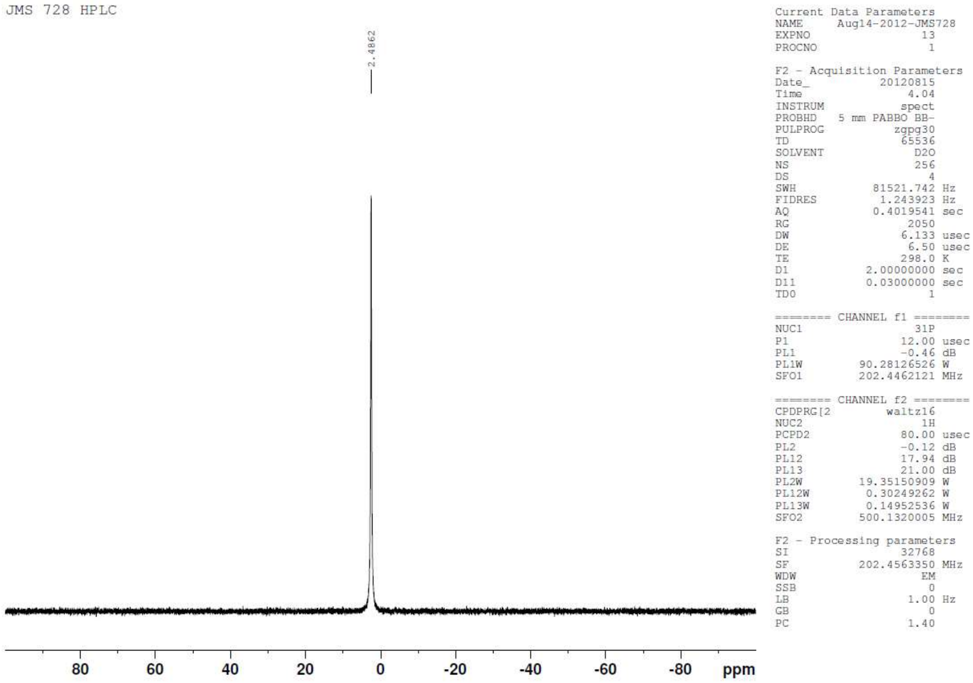

SAMPLE INFORMATION

Sample Name:	JMS 728 B4	Acquired By:	Joanna
Sample Type:	Unknown	Date Acquired:	02/08/2012 2:03:42 PM
Vial:	4	Acq. Method Set:	isocratic new PDA Jo
Injection #:	1	Date Processed:	11/09/2012 10:40:43 AM
Injection Volume:	10.00 ul	Processing Method:	JMS 728
Run Time:	12.0 Minutes	Channel Name:	Wvln Ch1
Sample Set Name:	JMS 728 HPLC B	Proc. Chnl. Descr.:	PDA 254.0 nm

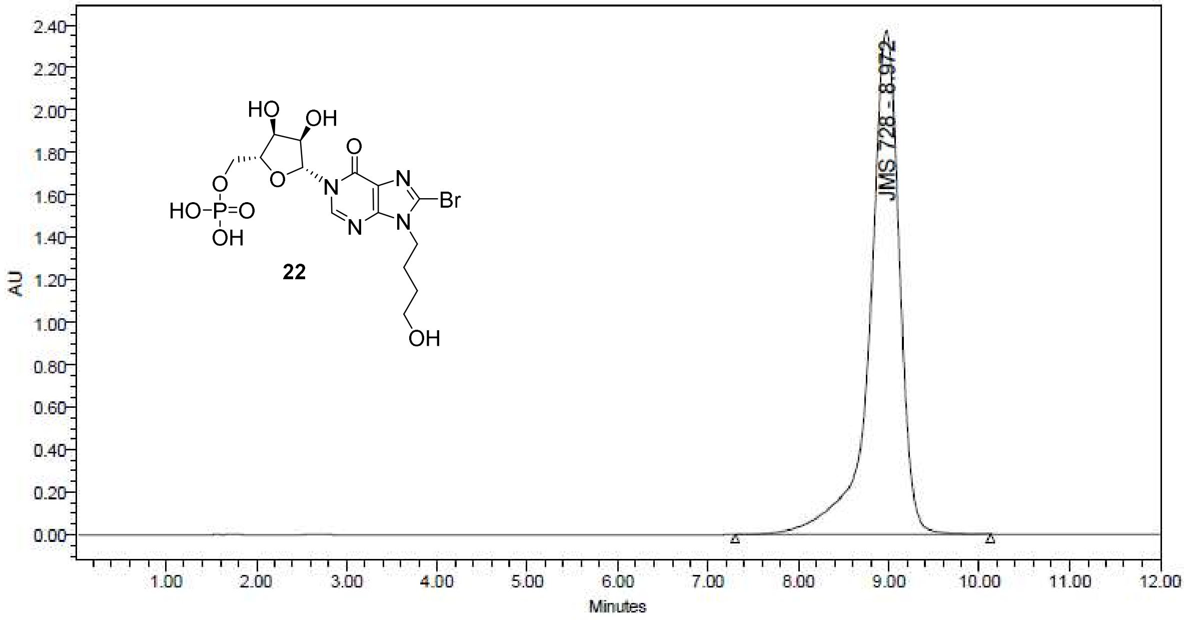

	Peak Name	RT	Area	% Area	Height
1	JMS 728	8.972	54319768	100.00	2372340

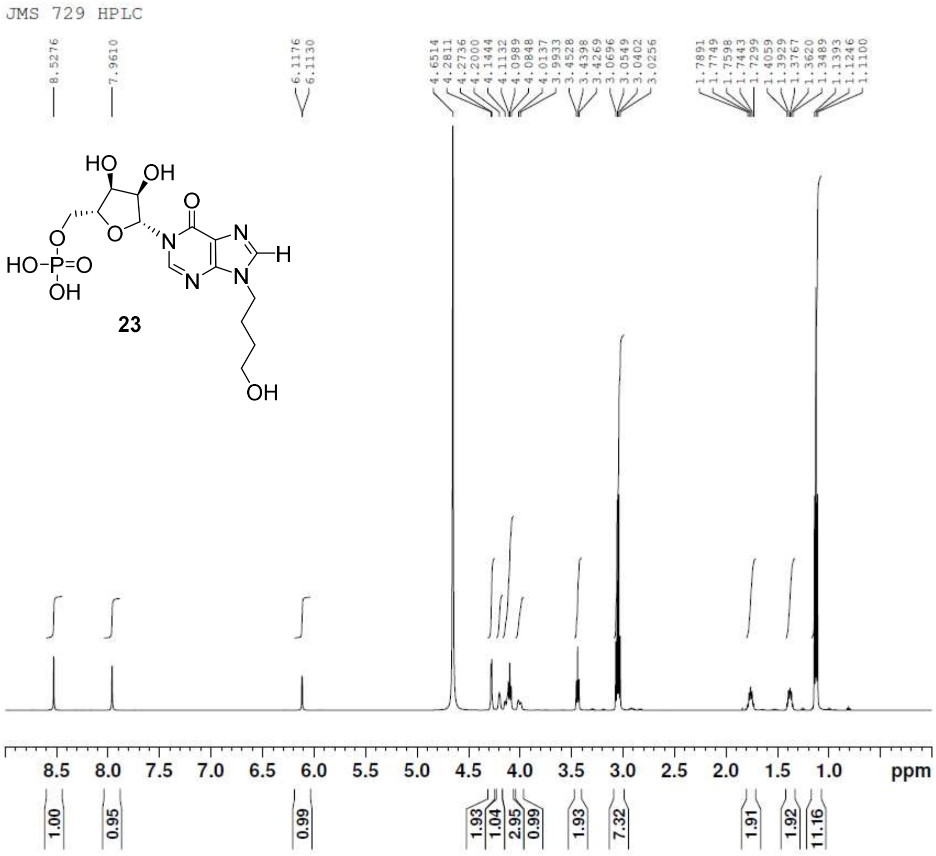


```
Current Data Parameters
NAME       Aug14-2012-JMS729
EXPNO                     10
PROCNO                     1

F2 - Acquisition Parameters
Date_               20120814
Time                   16.30
INSTRUM                spect
PROBHD     5 mm PABBO BB-
PULPROG                 zg30
TD                     65536
SOLVENT                  D2O
NS                        64
DS                         2
SWH               10330.578 Hz
FIDRES             0.157632 Hz
AQ                3.1719425 sec
RG                      50.8
DW                    48.400 usec
DE                     13.94 usec
TE                     298.0 K
D1               1.00000000 sec
TD0                        1

======== CHANNEL f1 ========
NUC1                      1H
P1                     10.30 usec
PL1                    -0.12 dB
PL1W             19.35150909 W
SFO1             500.1330885 MHz

F2 - Processing parameters
SI                     32768
SF               500.1300262 MHz
WDW                       EM
SSB                        0
LB                      0.30 Hz
GB                         0
PC                      1.00
```

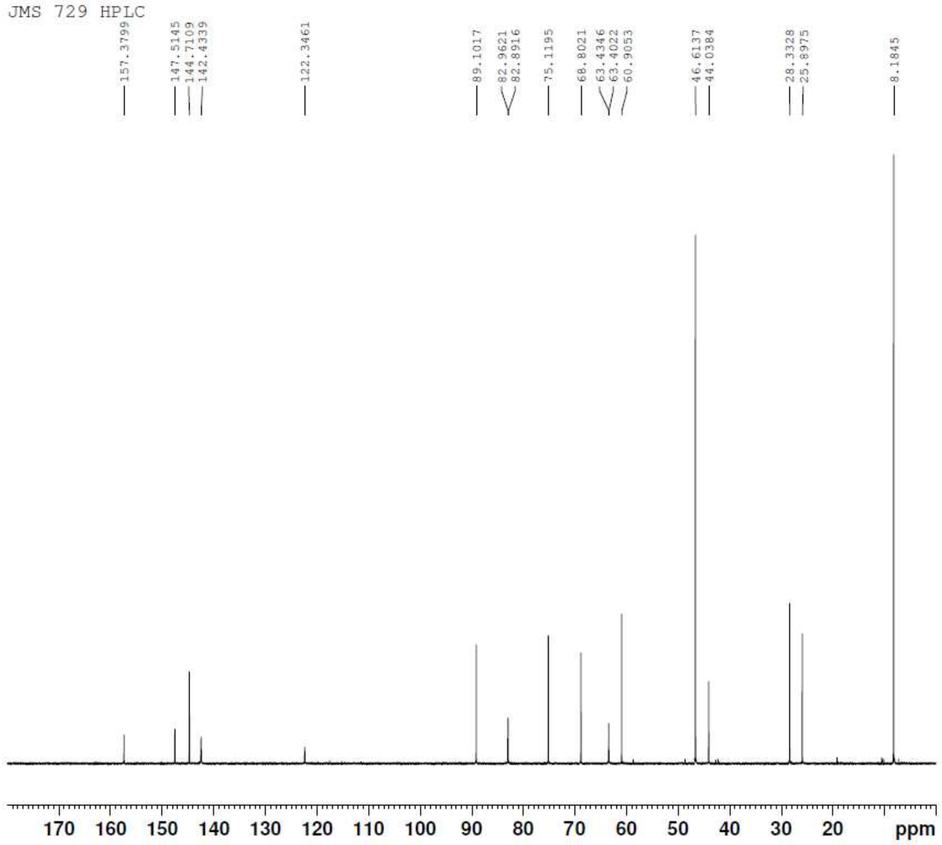


```
Current Data Parameters
NAME       Aug14-2012-JMS729
EXPNO                     11
PROCNO                     1

F2 - Acquisition Parameters
Date_               20120814
Time                   18.07
INSTRUM                spect
PROBHD     5 mm PABBO BB-
PULPROG               zgpg30
TD                     65536
SOLVENT                  D2O
NS                      5000
DS                         4
SWH               29761.904 Hz
FIDRES             0.454131 Hz
AQ                1.1010048 sec
RG                      2050
DW                    16.800 usec
DE                      8.43 usec
TE                     298.0 K
D1               2.00000000 sec
D11              0.03000000 sec
TD0                        1

======== CHANNEL f1 ========
NUC1                     13C
P1                      9.50 usec
PL1                    -0.51 dB
PL1W             99.92730713 W
SFO1             125.7703643 MHz

======== CHANNEL f2 ========
CPDPRG[2             waltz16
NUC2                      1H
PCPD2                  80.00 usec
PL2                    -0.12 dB
PL12                   17.94 dB
PL13                   21.00 dB
PL2W             19.35150909 W
PL12W             0.30249262 W
PL13W             0.14952536 W
SFO2             500.1320005 MHz

F2 - Processing parameters
SI                     32768
SF               125.7577890 MHz
WDW                       EM
SSB                        0
LB                      1.00 Hz
GB                         0
PC                      1.40
```

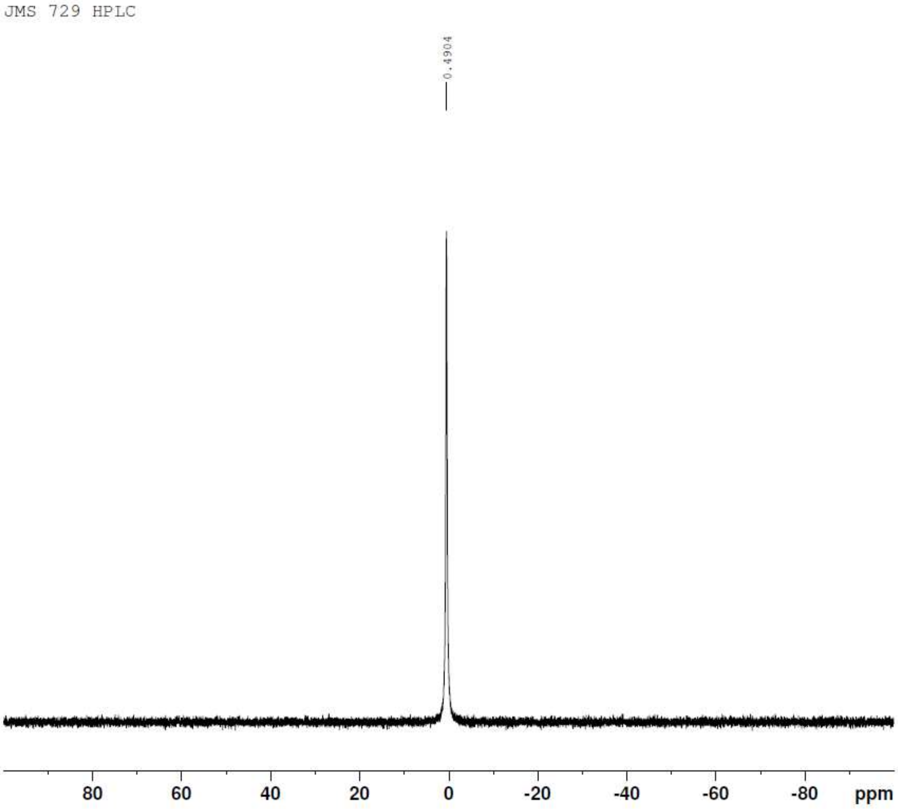


```
Current Data Parameters
NAME      Aug14-2012-JMS729
EXPNO                    13
PROCNO                    1

F2 - Acquisition Parameters
Date_              20120814
Time                  21.39
INSTRUM               spect
PROBHD   5 mm PABBO BB-
PULPROG             zgpg30
TD                   65536
SOLVENT                D2O
NS                     256
DS                       4
SWH            81521.742 Hz
FIDRES          1.243923 Hz
AQ             0.4019541 sec
RG                    2050
DW                   6.133 usec
DE                    6.50 usec
TE                   298.0 K
D1              2.00000000 sec
D11             0.03000000 sec
TD0                      1

======== CHANNEL f1 ========
NUC1                   31P
P1                   12.00 usec
PL1                  -0.46 dB
PL1W           90.28126526 W
SFO1           202.4462121 MHz

======== CHANNEL f2 ========
CPDPRG[2           waltz16
NUC2                    1H
PCPD2                80.00 usec
PL2                  -0.12 dB
PL12                 17.94 dB
PL13                 21.00 dB
PL2W           19.35150909 W
PL12W           0.30249262 W
PL13W           0.14952536 W
SFO2           500.1320005 MHz

F2 - Processing parameters
SI                   32768
SF             202.4563350 MHz
WDW                     EM
SSB                      0
LB                    1.00 Hz
GB                       0
PC                    1.40
```

SAMPLE INFORMATION

Sample Name:	JMS 729 D5	Acquired By:	Joanna
Sample Type:	Unknown	Date Acquired:	01/08/2012 6:46:31 PM
Vial:	54	Acq. Method Set:	isocratic new PDA Jo
Injection #:	1	Date Processed:	11/09/2012 10:41:43 AM
Injection Volume:	10.00 ul	Processing Method:	JMS 729
Run Time:	8.0 Minutes	Channel Name:	Wvln Ch1
Sample Set Name:	JMS 729 HPLC D	Proc. Chnl. Descr.:	PDA 254.0 nm

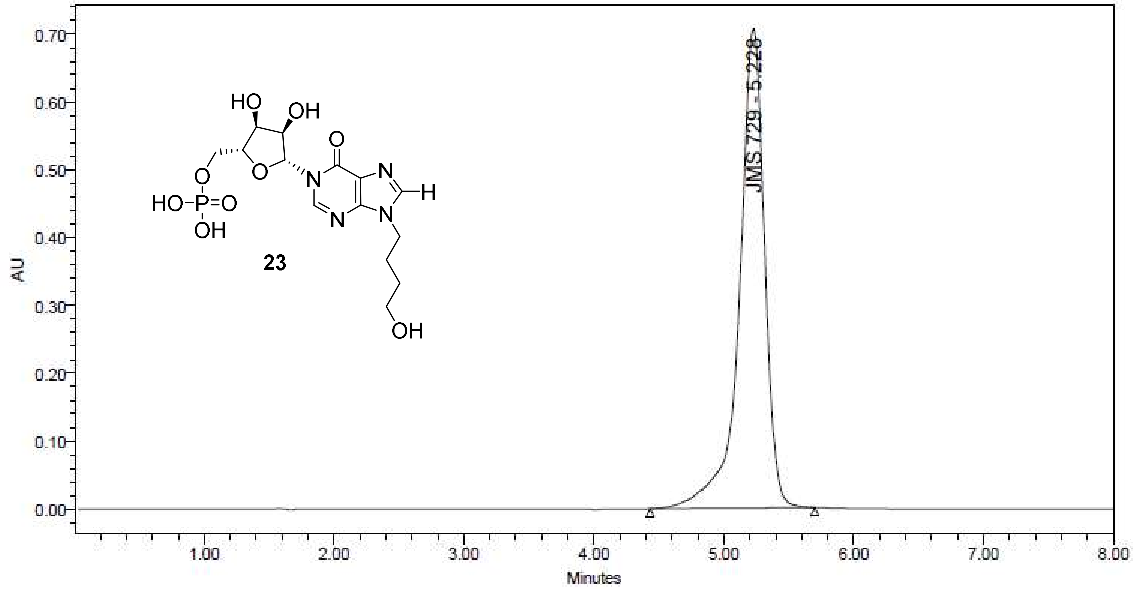

	Peak Name	RT	Area	% Area	Height
1	JMS 729	5.228	9565167	100.00	706064

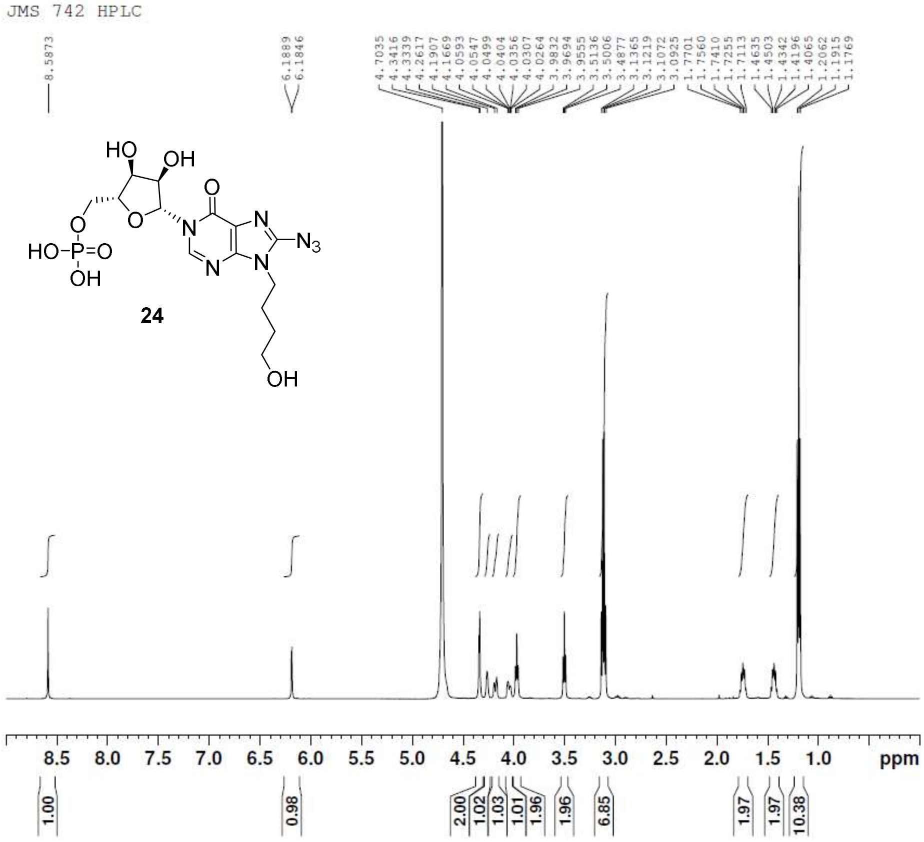

Current Data Parameters
NAME Oct10-2012-JMS742HPLC
EXPNO 10
PROCNO 1

F2 - Acquisition Parameters
Date_ 20121010
Time 17.17
INSTRUM spect
PROBHD 5 mm PABBO BB-
PULPROG zg30
TD 65536
SOLVENT D2O
NS 64
DS 2
SWH 10330.578 Hz
FIDRES 0.157632 Hz
AQ 3.1719425 sec
RG 114
DW 48.400 usec
DE 13.94 usec
TE 298.0 K
D1 1.00000000 sec
TD0 1

======== CHANNEL f1 ========
NUC1 1H
P1 10.30 usec
PL1 -0.12 dB
PL1W 19.35150909 W
SFO1 500.1330885 MHz

F2 - Processing parameters
SI 32768
SF 500.1300000 MHz
WDW EM
SSB 0
LB 0.30 Hz
GB 0
PC 1.00

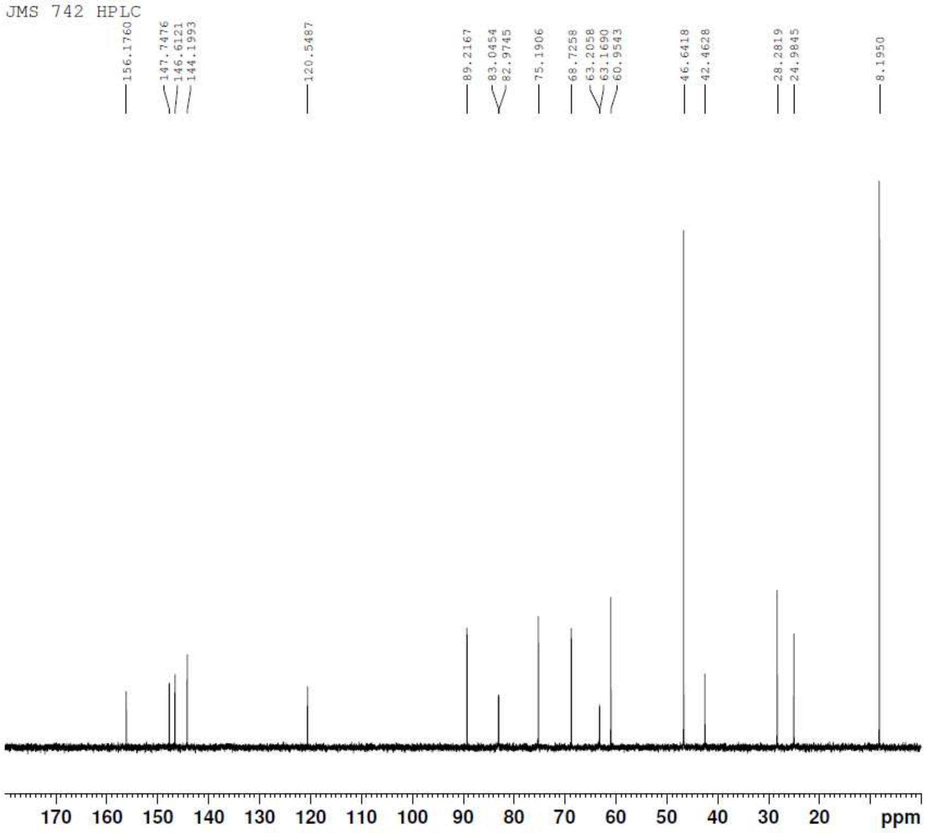

Current Data Parameters
NAME Oct10-2012-JMS742HPLC
EXPNO 12
PROCNO 1

F2 - Acquisition Parameters
Date_ 20121010
Time 21.48
INSTRUM spect
PROBHD 5 mm PABBO BB-
PULPROG zgpg30
TD 65536
SOLVENT D2O
NS 5000
DS 4
SWH 29761.904 Hz
FIDRES 0.454131 Hz
AQ 1.1010048 sec
RG 2050
DW 16.800 usec
DE 8.43 usec
TE 298.0 K
D1 2.00000000 sec
D11 0.03000000 sec
TD0 1

======== CHANNEL f1 ========
NUC1 13C
P1 9.50 usec
PL1 -0.51 dB
PL1W 99.92730713 W
SFO1 125.7703643 MHz

======== CHANNEL f2 ========
CPDPRG[2 waltz16
NUC2 1H
PCPD2 80.00 usec
PL2 -0.12 dB
PL12 17.94 dB
PL13 21.00 dB
PL2W 19.35150909 W
PL12W 0.30249262 W
PL13W 0.14952536 W
SFO2 500.1320005 MHz

F2 - Processing parameters
SI 32768
SF 125.7577890 MHz
WDW EM
SSB 0
LB 1.00 Hz
GB 0
PC 1.40

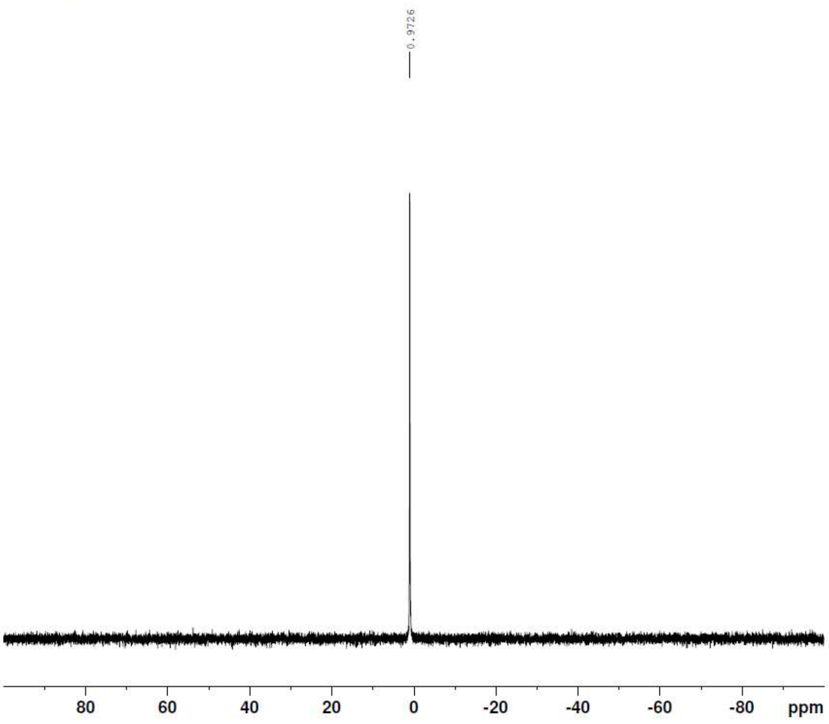


```
Current Data Parameters
NAME       Oct10-2012-JMS742HPLC
EXPNO                 18
PROCNO                 1

F2 - Acquisition Parameters
Date_           20121011
Time               11.25
INSTRUM            spect
PROBHD   5 mm PABBO BB-
PULPROG          zgpg30
TD                 65536
SOLVENT              D2O
NS                   127
DS                     4
SWH            81521.742 Hz
FIDRES          1.243923 Hz
AQ             0.4019541 sec
RG                  2050
DW                 6.133 usec
DE                  6.50 usec
TE                 297.8 K
D1            2.00000000 sec
D11           0.03000000 sec
TD0                    1

======== CHANNEL f1 ========
NUC1                 31P
P1                 12.00 usec
PL1                -0.46 dB
PL1W         90.28126526 W
SFO1         202.4462121 MHz

======== CHANNEL f2 ========
CPDPRG[2         waltz16
NUC2                  1H
PCPD2              80.00 usec
PL2                -0.12 dB
PL12               17.94 dB
PL13               21.00 dB
PL2W         19.35150909 W
PL12W         0.30249262 W
PL13W         0.14952536 W
SFO2         500.1320005 MHz

F2 - Processing parameters
SI                 32768
SF           202.4563350 MHz
WDW                   EM
SSB                    0
LB                  1.00 Hz
GB                     0
PC                  1.40
```

SAMPLE INFORMATION

Sample Name:	JMS 742 C4	Acquired By:	Joanna
Sample Type:	Unknown	Date Acquired:	09/10/2012 7:32:55 PM
Vial:	28	Acq. Method Set:	isocratic new PDA Jo
Injection #:	1	Date Processed:	09/04/2021 3:04:01 PM
Injection Volume:	10.00 ul	Processing Method:	8_N3_butyl_IMP
Run Time:	15.0 Minutes	Channel Name:	Wvln Ch1
Sample Set Name:	JMS 742 C	Proc. Chnl. Descr.:	PDA 254.0 nm

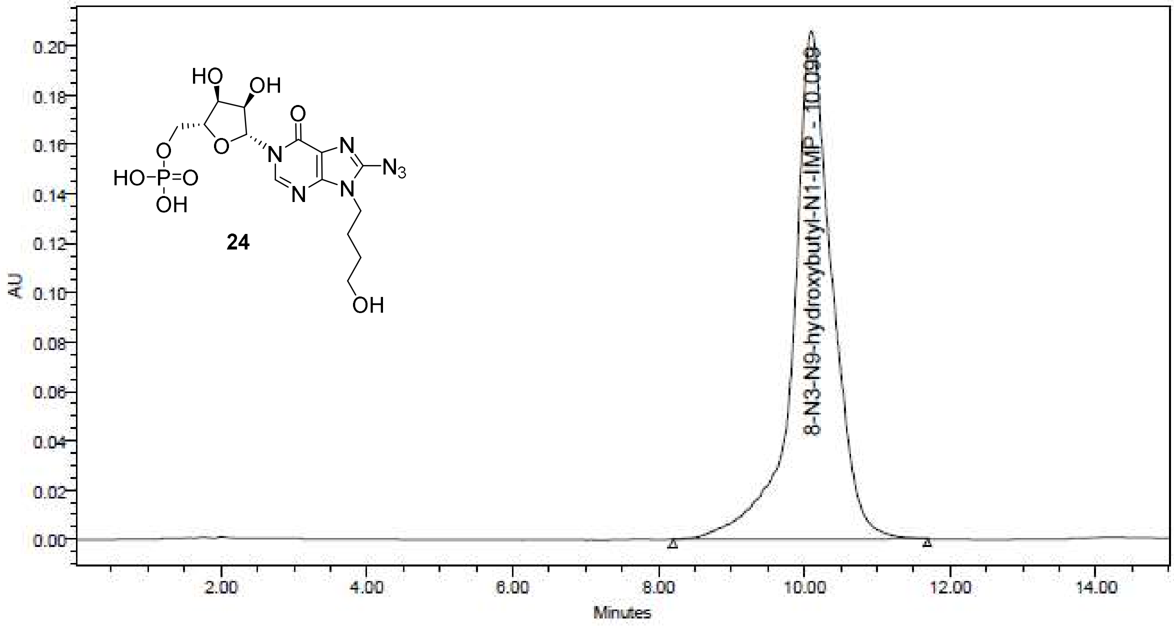

	Peak Name	RT	Area	% Area	Height
1	8-N3-N9-hydroxybutyl-N1-IMP	10.099	7699465	100.00	205493

JMS 741 HPLC

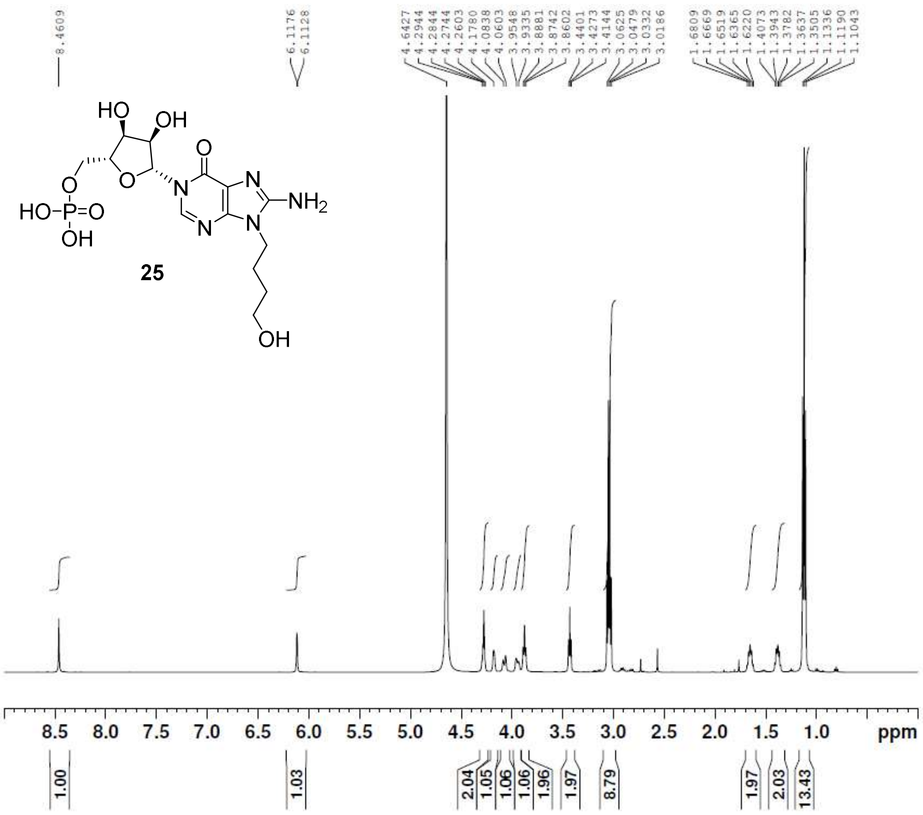


```
Current Data Parameters
NAME       Oct08-2012-JMS741HPLC
EXPNO                 10
PROCNO                 1

F2 - Acquisition Parameters
Date_           20121008
Time               12.11
INSTRUM            spect
PROBHD   5 mm PABBO BB-
PULPROG             zg30
TD                 65536
SOLVENT              D2O
NS                   100
DS                     2
SWH            10330.578 Hz
FIDRES          0.157632 Hz
AQ             3.1719425 sec
RG                  71.8
DW                48.400 usec
DE                 13.94 usec
TE                 298.0 K
D1            1.00000000 sec
TD0                    1

======== CHANNEL f1 ========
NUC1                  1H
P1                 10.30 usec
PL1                -0.12 dB
PL1W         19.35150909 W
SFO1         500.1330885 MHz

F2 - Processing parameters
SI                 32768
SF           500.1300305 MHz
WDW                   EM
SSB                    0
LB                  0.30 Hz
GB                     0
PC                  1.00
```

JMS 741 HPLC

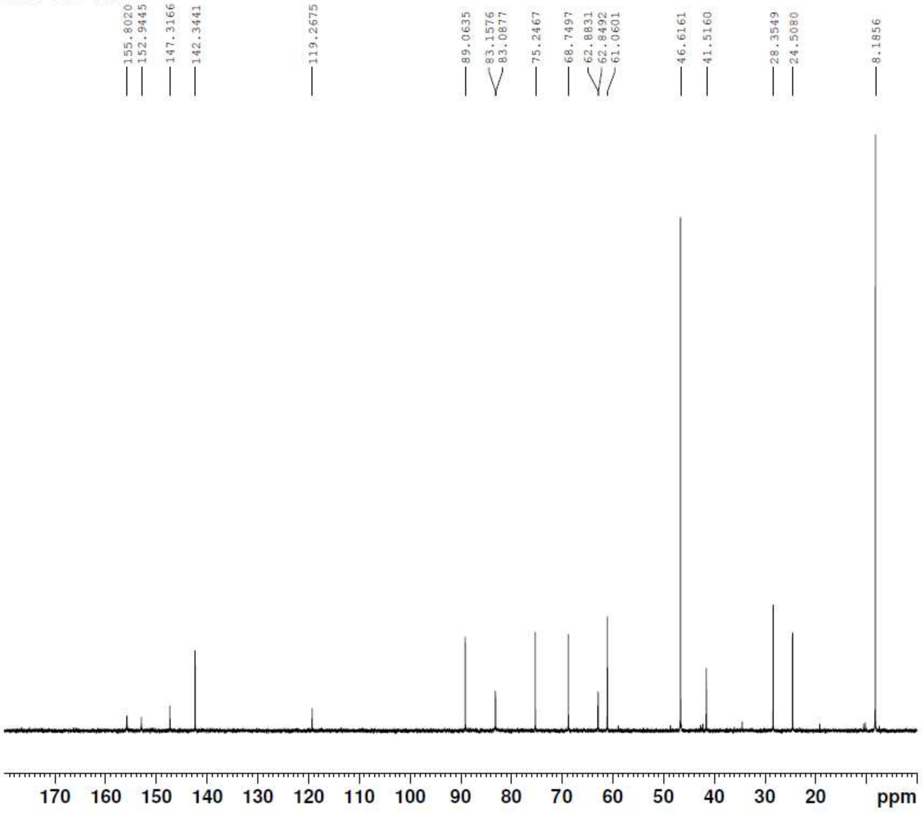


```
Current Data Parameters
NAME       Oct08-2012-JMS741HPLC
EXPNO                 12
PROCNO                 1

F2 - Acquisition Parameters
Date_           20121008
Time               16.55
INSTRUM            spect
PROBHD   5 mm PABBO BB-
PULPROG           zgpg30
TD                 65536
SOLVENT              D2O
NS                  5000
DS                     4
SWH            29761.904 Hz
FIDRES          0.454131 Hz
AQ             1.1010048 sec
RG                  2050
DW                16.800 usec
DE                  8.43 usec
TE                 298.0 K
D1            2.00000000 sec
D11           0.03000000 sec
TD0                    1

======== CHANNEL f1 ========
NUC1                 13C
P1                  9.50 usec
PL1                -0.51 dB
PL1W         99.92730713 W
SFO1         125.7703643 MHz

======== CHANNEL f2 ========
CPDPRG[2         waltz16
NUC2                  1H
PCPD2              80.00 usec
PL2                -0.12 dB
PL12               17.94 dB
PL13               21.00 dB
PL2W         19.35150909 W
PL12W         0.30249262 W
PL13W         0.14952536 W
SFO2         500.1320005 MHz

F2 - Processing parameters
SI                 32768
SF           125.7577890 MHz
WDW                   EM
SSB                    0
LB                  1.00 Hz
GB                     0
PC                  1.40
```

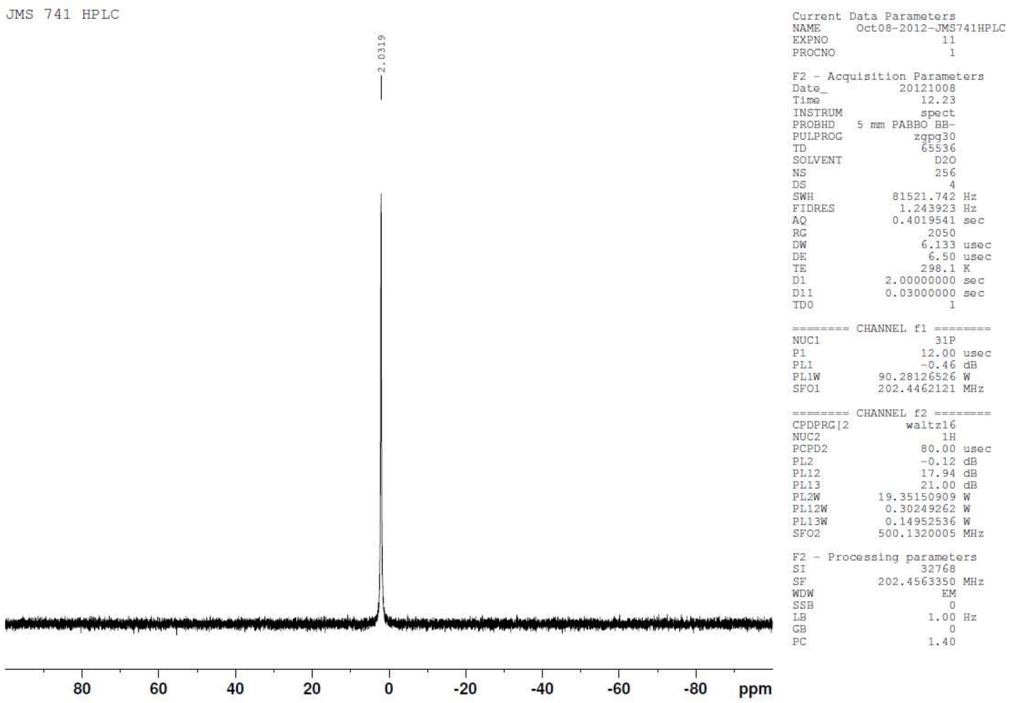

SAMPLE INFORMATION

Sample Name:	JMS 743 E4	Acquired By:	Joanna
Sample Type:	Unknown	Date Acquired:	05/10/2012 10:41:28 AM
Vial:	39	Acq. Method Set:	isocratic new PDA Jo
Injection #:	1	Date Processed:	05/10/2012 12:33:23 PM
Injection Volume:	10.00 ul	Processing Method:	8NH2 N9butyl IMP
Run Time:	10.0 Minutes	Channel Name:	Wvln Ch1
Sample Set Name:	JMS 743 E	Proc. Chnl. Descr.:	PDA 254.0 nm

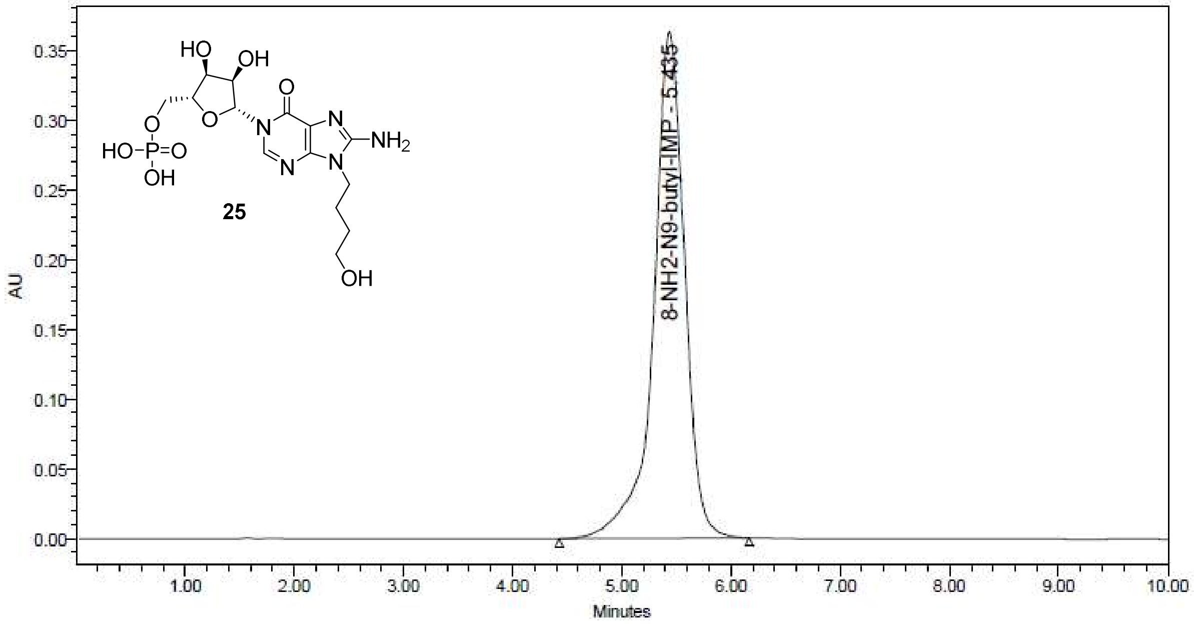

	Peak Name	RT	Area	% Area	Height
1	8-NH2-N9-butyl-IMP	5.435	7152416	100.00	362896

Total synthesis of L-*N*1-IMP analogues (26-27)

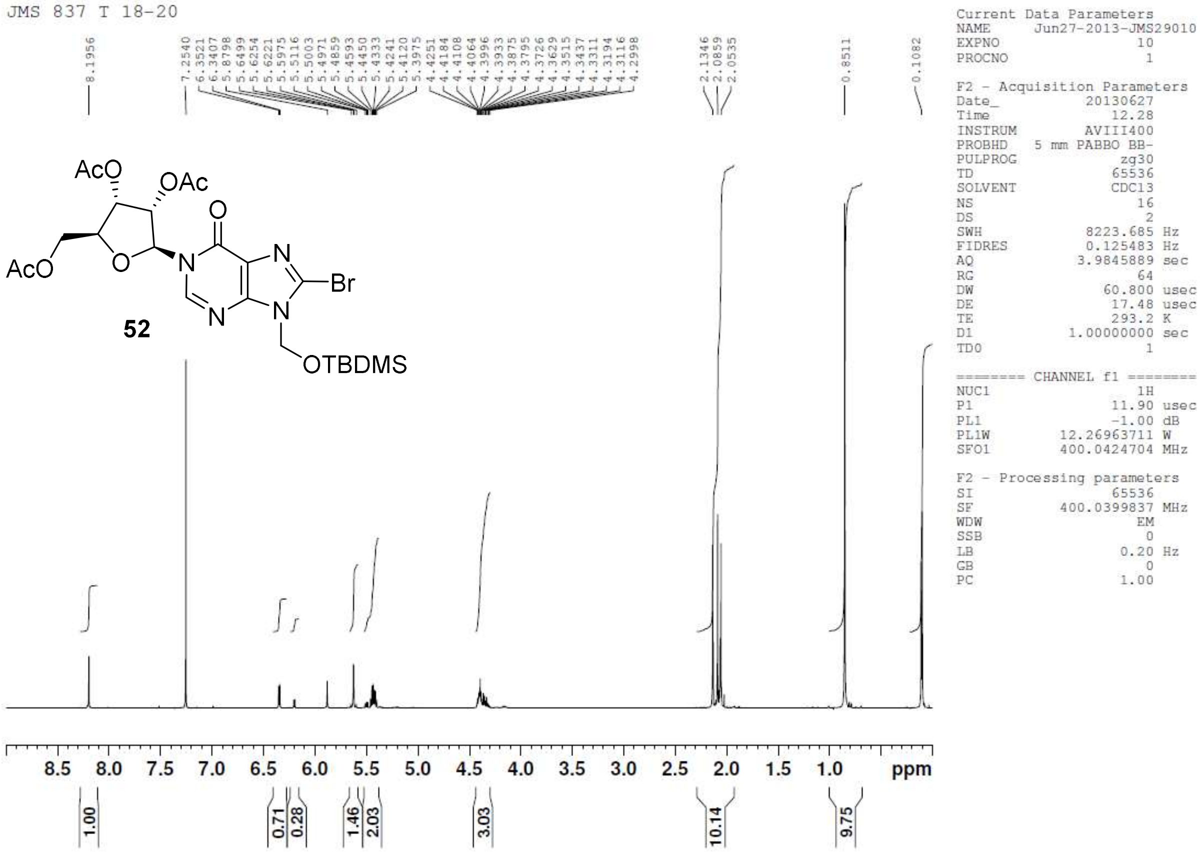

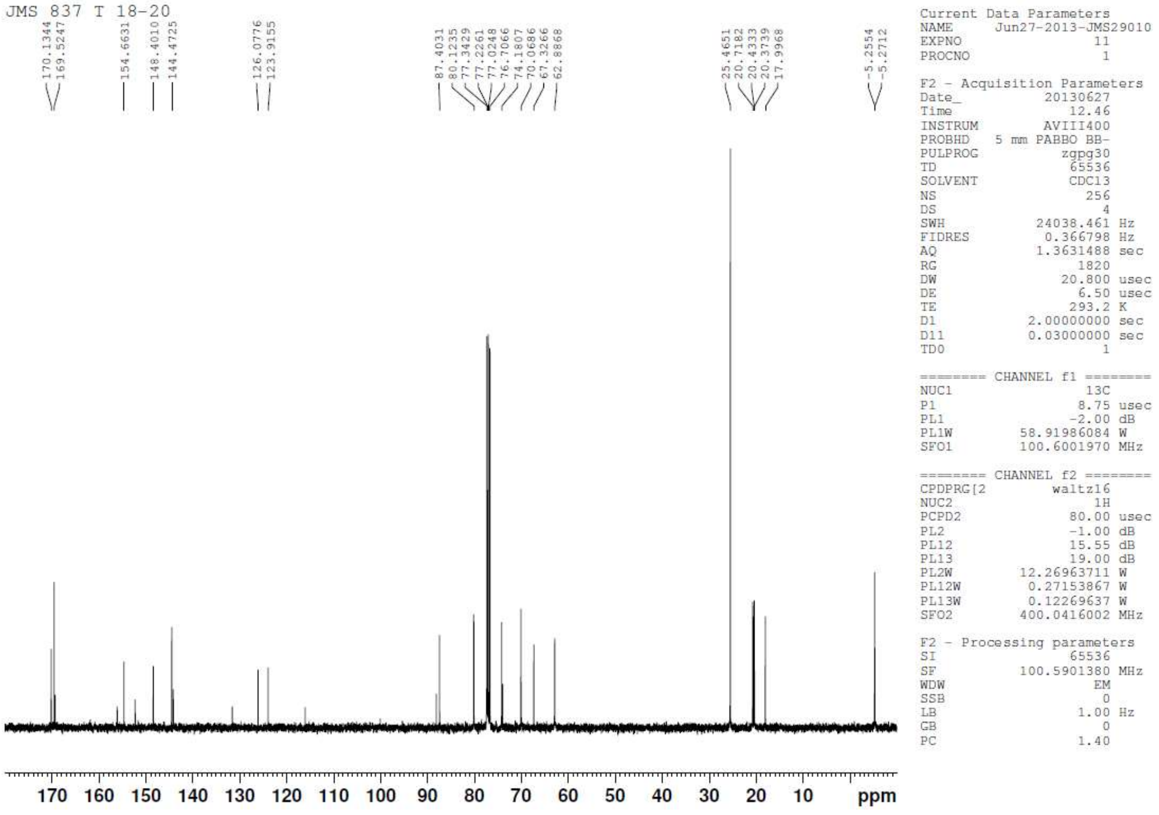

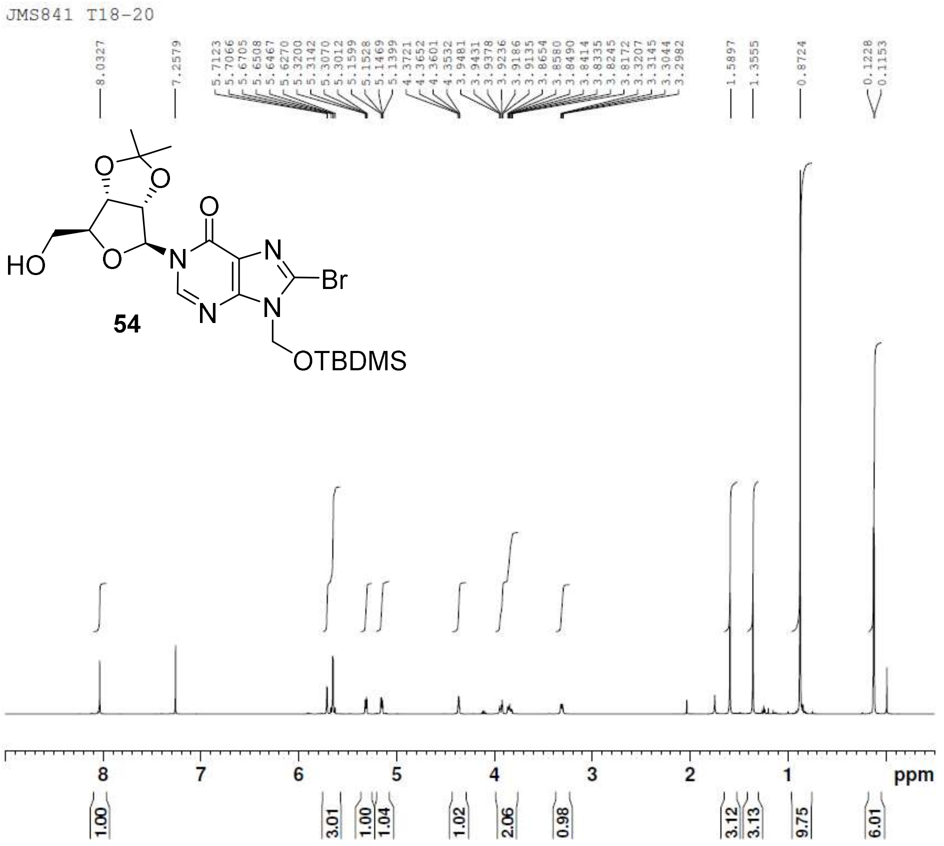

Current Data Parameters
NAME JMS841 T18-20
EXPNO 10
PROCNO 1

F2 - Acquisition Parameters
Date_ 20130704
Time 15.52
INSTRUM spect
PROBHD 5 mm PABBO BB-
PULPROG zg30
TD 65536
SOLVENT CDCl3
NS 16
DS 2
SWH 10330.578 Hz
FIDRES 0.157632 Hz
AQ 3.1719425 sec
RG 101
DW 48.400 usec
DE 14.00 usec
TE 298.2 K
D1 1.00000000 sec
TD0 1

======== CHANNEL f1 ========
NUC1 1H
P1 10.00 usec
PL1 -0.12 dB
PL1W 19.35150909 W
SFO1 500.1330885 MHz

F2 - Processing parameters
SI 32768
SF 500.1300140 MHz
WDW EM
SSB 0
LB 0.30 Hz
GB 0
PC 1.00

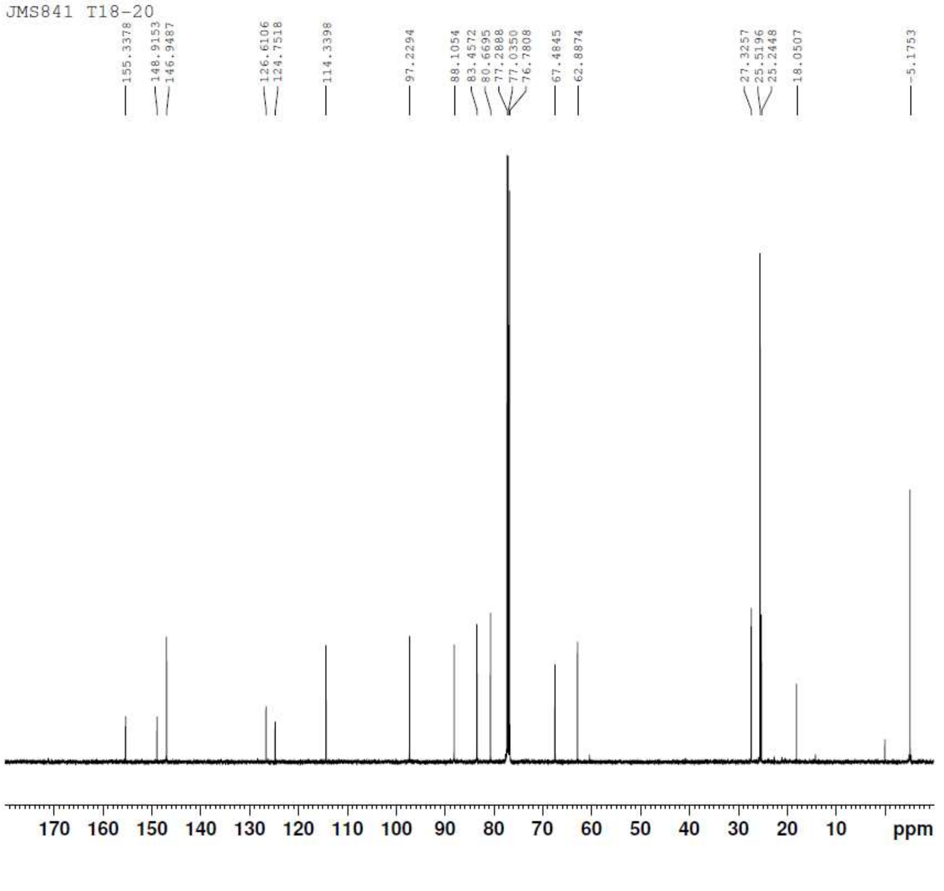

Current Data Parameters
NAME JMS841 T18-20
EXPNO 12
PROCNO 1

F2 - Acquisition Parameters
Date_ 20130704
Time 16.08
INSTRUM spect
PROBHD 5 mm PABBO BB-
PULPROG zgpg30
TD 65536
SOLVENT CDCl3
NS 1024
DS 4
SWH 29761.904 Hz
FIDRES 0.454131 Hz
AQ 1.1010048 sec
RG 2050
DW 16.800 usec
DE 8.45 usec
TE 298.2 K
D1 2.00000000 sec
D11 0.03000000 sec
TD0 1

======== CHANNEL f1 ========
NUC1 13C
P1 9.40 usec
PL1 -0.51 dB
PL1W 99.92730713 W
SFO1 125.7703643 MHz

======== CHANNEL f2 ========
CPDPRG[2 waltz16
NUC2 1H
PCPD2 80.00 usec
PL2 -0.12 dB
PL12 17.94 dB
PL13 21.00 dB
PL2W 19.35150909 W
PL12W 0.30249262 W
PL13W 0.14952536 W
SFO2 500.1320005 MHz

F2 - Processing parameters
SI 32768
SF 125.7577895 MHz
WDW EM
SSB 0
LB 1.00 Hz
GB 0
PC 1.40

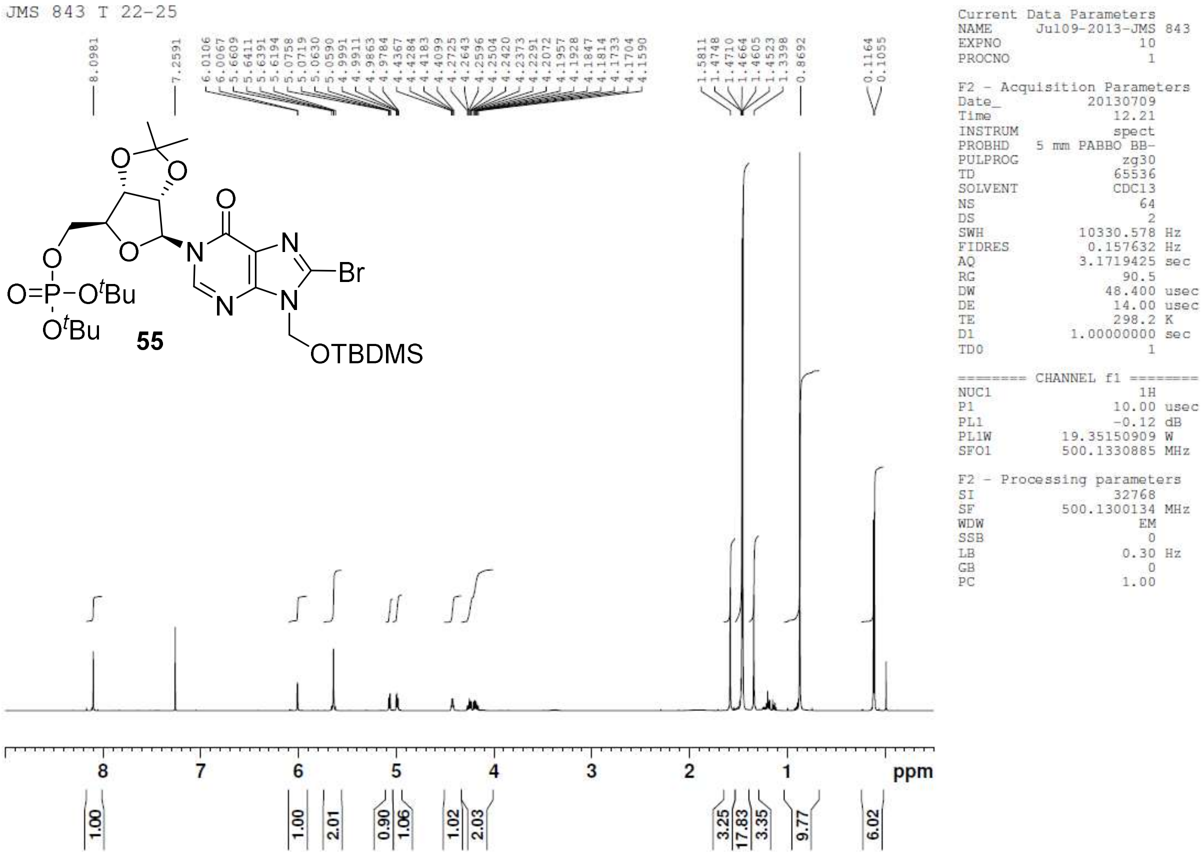
JMS 843 T 22-25
8.0981
7.2591
6.0106 6.0067 5.6609 5.6411 5.6391 5.6194 5.0758 5.0719 5.0630 5.0590 4.9991 4.9911 4.9863 4.9784 4.4367 4.4284 4.4183 4.4099 4.2725 4.2643 4.2596 4.2504 4.2420 4.2373 4.2291 4.2072 4.1957 4.1928 4.1847 4.1814 4.1733 4.1704 4.1590
1.5811 1.4748 1.4710 1.4664 1.4605 1.4523 1.3398 0.8692
0.1164 0.1055
O=P−O^{t}Bu
O^{t}Bu
55
Br
OTBDMS
1.00 1.00 2.01 0.90 1.06 1.02 2.03 3.25 17.83 3.35 9.77 6.02
ppm
Current Data Parameters
NAME Jul09-2013-JMS 843
EXPNO 10
PROCNO 1
F2 - Acquisition Parameters
Date_ 20130709
Time 12.21
INSTRUM spect
PROBHD 5 mm PABBO BB-
PULPROG zg30
TD 65536
SOLVENT CDCl3
NS 64
DS 2
SWH 10330.578 Hz
FIDRES 0.157632 Hz
AQ 3.1719425 sec
RG 90.5
DW 48.400 usec
DE 14.00 usec
TE 298.2 K
D1 1.00000000 sec
TD0 1
======== CHANNEL f1 ========
NUC1 1H
P1 10.00 usec
PL1 -0.12 dB
PL1W 19.35150909 W
SFO1 500.1330885 MHz
F2 - Processing parameters
SI 32768
SF 500.1300134 MHz
WDW EM
SSB 0
LB 0.30 Hz
GB 0
PC 1.00

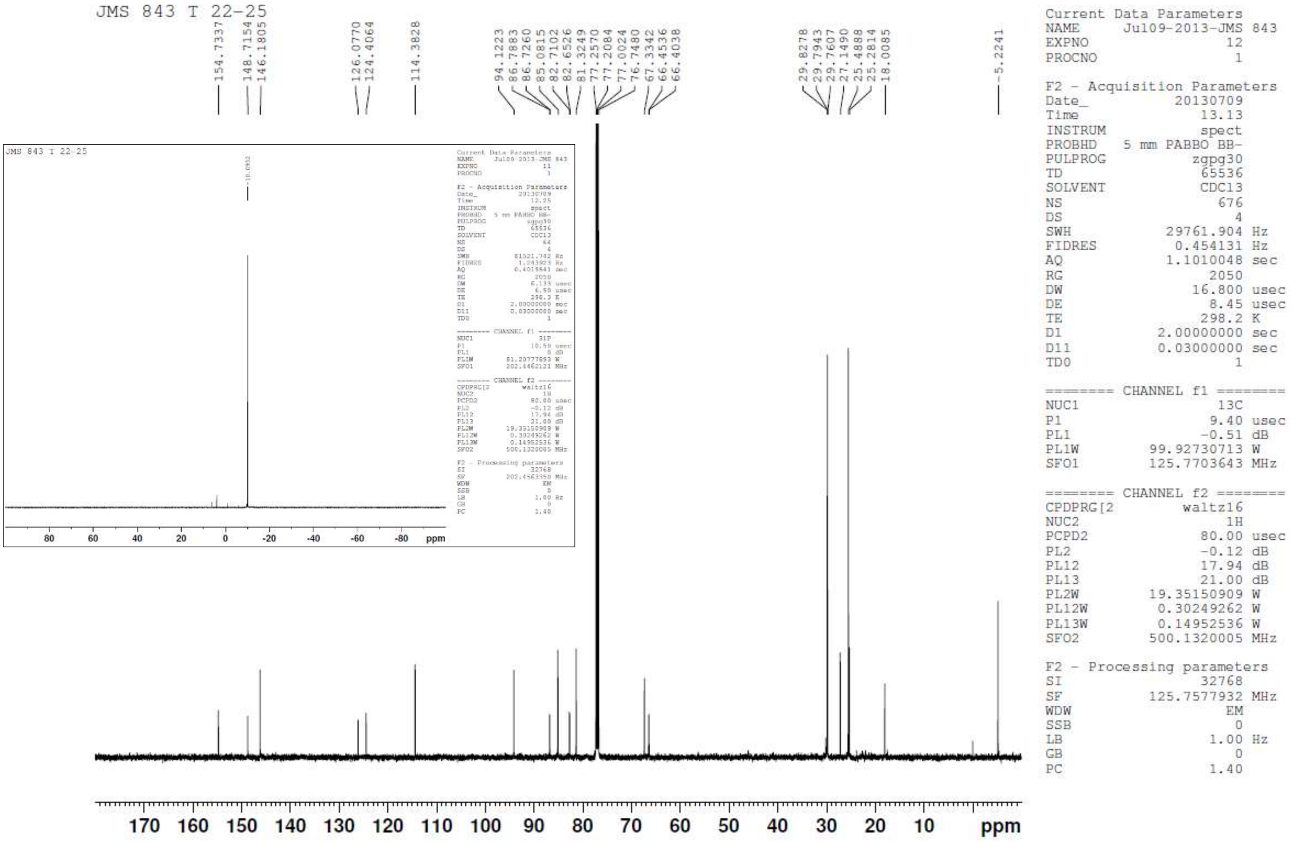
JMS 843 T 22-25
154.7337
148.7154 146.1805
126.0770 124.4064
114.3828
94.1223 86.7883 86.7260 85.0835 82.7102 82.6526 81.3249 77.2570 77.2084 77.0024 76.7480 67.3342 66.4536 66.4038
29.8278 29.7943 29.7607 27.1490 25.4888 25.2814 18.0085
-5.2241
ppm
Current Data Parameters
NAME Jul09-2013-JMS 843
EXPNO 12
PROCNO 1
F2 - Acquisition Parameters
Date_ 20130709
Time 13.13
INSTRUM spect
PROBHD 5 mm PABBO BB-
PULPROG zgpg30
TD 65536
SOLVENT CDCl3
NS 676
DS 4
SWH 29761.904 Hz
FIDRES 0.454131 Hz
AQ 1.1010048 sec
RG 2050
DW 16.800 usec
DE 8.45 usec
TE 298.2 K
D1 2.00000000 sec
D11 0.03000000 sec
TD0 1
======== CHANNEL f1 ========
NUC1 13C
P1 9.40 usec
PL1 -0.51 dB
PL1W 99.92730713 W
SFO1 125.7703643 MHz
======== CHANNEL f2 ========
CPDPRG[2 waltz16
NUC2 1H
PCPD2 80.00 usec
PL2 -0.12 dB
PL12 17.94 dB
PL13 21.00 dB
PL2W 19.35150909 W
PL12W 0.30249262 W
PL13W 0.14952536 W
SFO2 500.1320005 MHz
F2 - Processing parameters
SI 32768
SF 125.7577932 MHz
WDW EM
SSB 0
LB 1.00 Hz
GB 0
PC 1.40

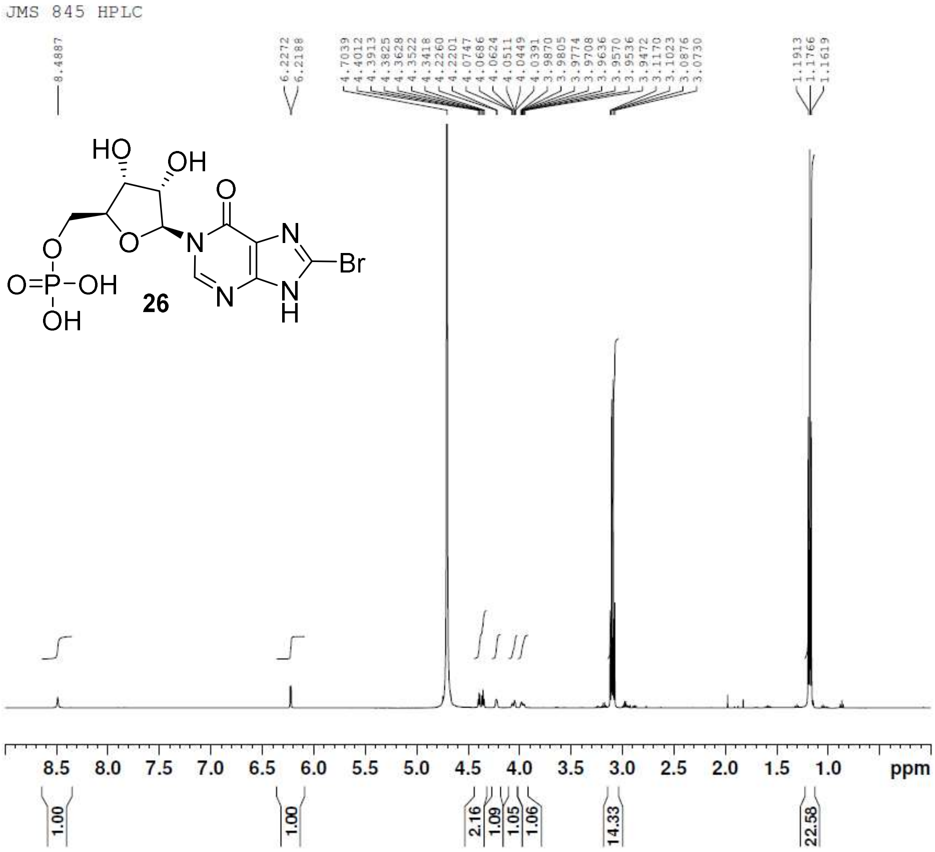


```
Current Data Parameters
NAME          JMS 845 HPLC
EXPNO                   10
PROCNO                   1

F2 - Acquisition Parameters
Date_             20130718
Time                 15.38
INSTRUM              spect
PROBHD    5 mm PABBO BB-
PULPROG               zg30
TD                   65536
SOLVENT                D2O
NS                     128
DS                       0
SWH              10330.578 Hz
FIDRES            0.157632 Hz
AQ               3.1719425 sec
RG                    80.6
DW                  48.400 usec
DE                    6.50 usec
TE                   298.2 K
D1              2.00000000 sec
TD0                      1

======== CHANNEL f1 ========
NUC1                    1H
P1                   10.00 usec
PL1                  -0.12 dB
PL1W           19.35150909 W
SFO1           500.1330885 MHz

F2 - Processing parameters
SI                   32768
SF             500.1300000 MHz
WDW                     EM
SSB                      0
LB                    0.30 Hz
GB                       0
PC                    1.00
```

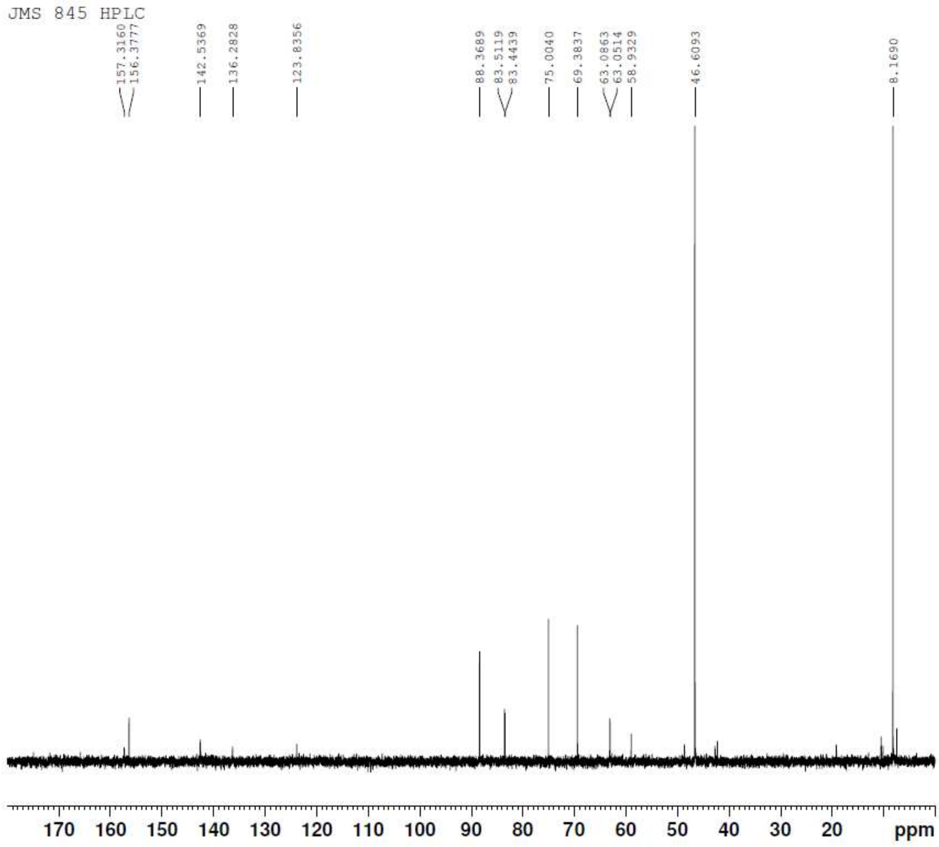


```
Current Data Parameters
NAME          JMS 845 HPLC
EXPNO                   19
PROCNO                   1

F2 - Acquisition Parameters
Date_             20130719
Time                  5.36
INSTRUM              spect
PROBHD    5 mm PABBO BB-
PULPROG             zgpg30
TD                   65536
SOLVENT                D2O
NS                   10240
DS                       4
SWH              29761.904 Hz
FIDRES            0.454131 Hz
AQ               1.1010048 sec
RG                    2050
DW                  16.800 usec
DE                    8.45 usec
TE                   298.2 K
D1              2.00000000 sec
D11             0.03000000 sec
TD0                      1

======== CHANNEL f1 ========
NUC1                   13C
P1                    9.40 usec
PL1                  -0.51 dB
PL1W           99.92730713 W
SFO1           125.7703643 MHz

======== CHANNEL f2 ========
CPDPRG[2           waltz16
NUC2                    1H
PCPD2                80.00 usec
PL2                  -0.12 dB
PL12                 17.94 dB
PL13                 21.00 dB
PL2W           19.35150909 W
PL12W           0.30249262 W
PL13W           0.14952536 W
SFO2           500.1320005 MHz

F2 - Processing parameters
SI                   32768
SF             125.7577890 MHz
WDW                     EM
SSB                      0
LB                    1.00 Hz
GB                       0
PC                    1.40
```

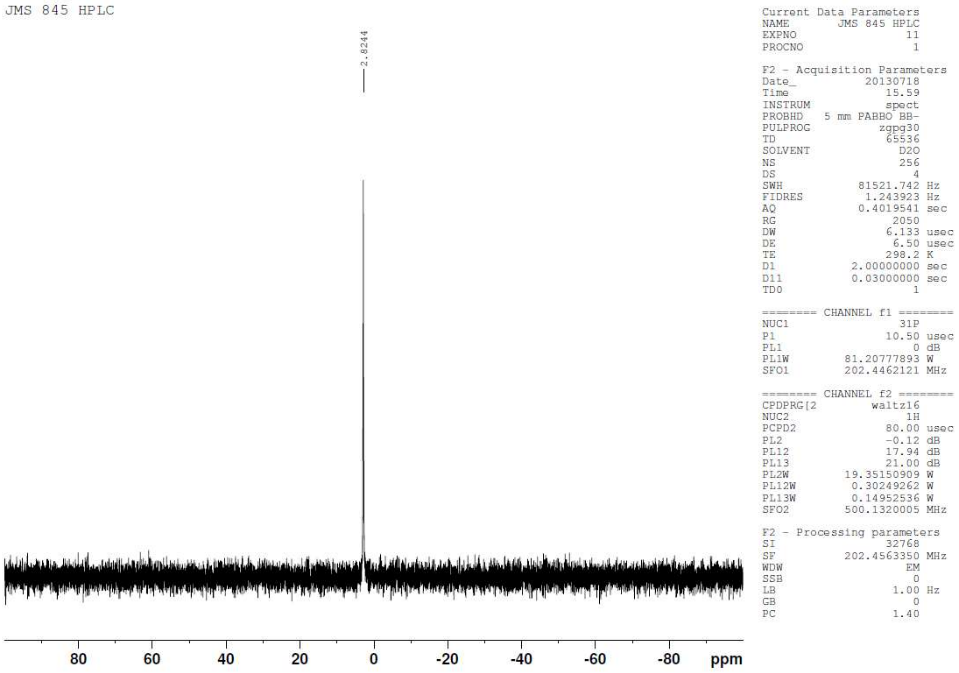

SAMPLE INFORMATION

Sample Name:	JMS 845 Batch 1 from UV	Acquired By:	Joanna
Sample Type:	Unknown	Date Acquired:	26/09/2013 3:32:14 PM
Vial:	56	Acq. Method Set:	isocratic new PDA Jo
Injection #:	1	Date Processed:	27/09/2013 3:44:26 PM
Injection Volume:	10.00 ul	Processing Method:	8 Br L N1 IMP
Run Time:	30.0 Minutes	Channel Name:	Wvln Ch1
Sample Set Name:	JMS 845 Batch 1	Proc. Chnl. Descr.:	PDA 254.0 nm

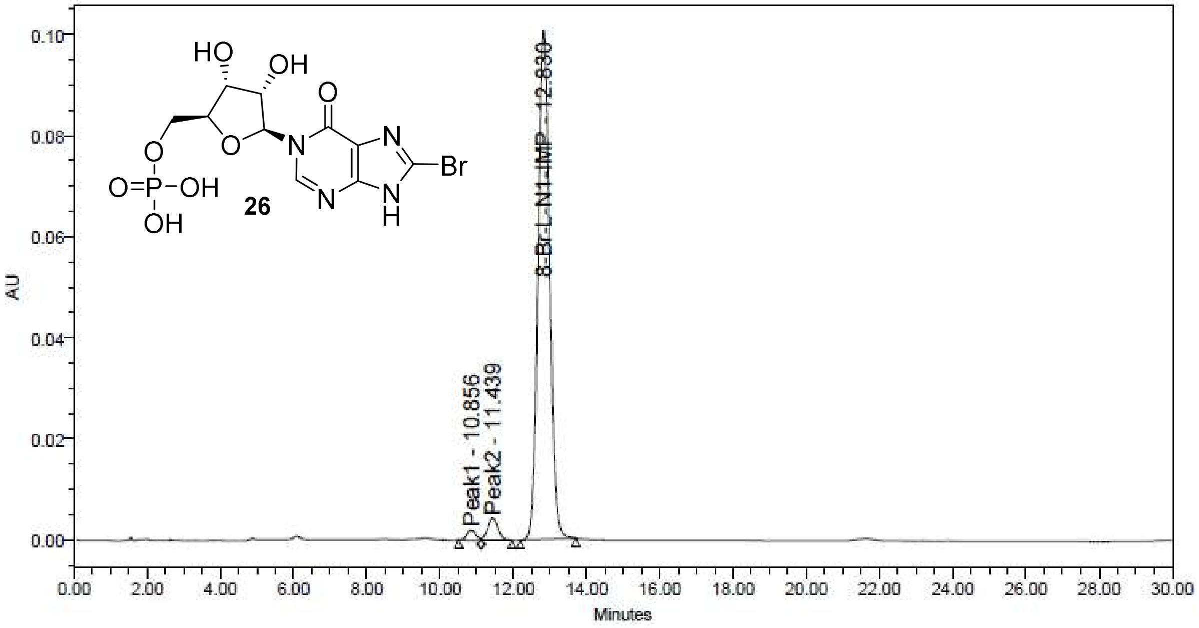

	Peak Name	RT	Area	% Area	Height
1	Peak1	10.856	32649	1.41	2001
2	Peak2	11.439	84320	3.64	4337
3	8-Br-L-N1-IMP	12.830	2197357	94.95	100598

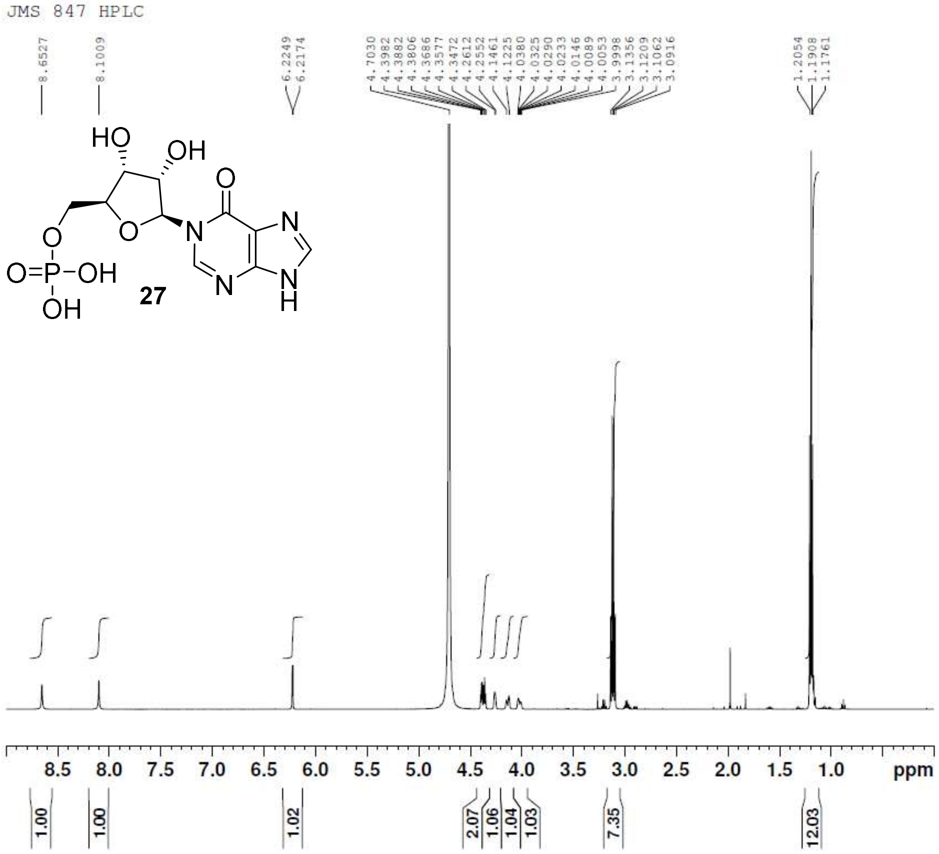


```
Current Data Parameters
NAME          JMS 847 HPLC
EXPNO                   10
PROCNO                   1

F2 - Acquisition Parameters
Date_             20130716
Time                 15.41
INSTRUM              spect
PROBHD   5 mm PABBO BB-
PULPROG               zg30
TD                   65536
SOLVENT                D2O
NS                     128
DS                       0
SWH              10330.578 Hz
FIDRES            0.157632 Hz
AQ               3.1719425 sec
RG                    80.6
DW                  48.400 usec
DE                    6.50 usec
TE                   298.2 K
D1              2.00000000 sec
TD0                      1

======== CHANNEL f1 ========
NUC1                    1H
P1                   10.00 usec
PL1                  -0.12 dB
PL1W           19.35150909 W
SFO1           500.1330885 MHz

F2 - Processing parameters
SI                   32768
SF             500.1300000 MHz
WDW                     EM
SSB                      0
LB                    0.30 Hz
GB                       0
PC                    1.00
```

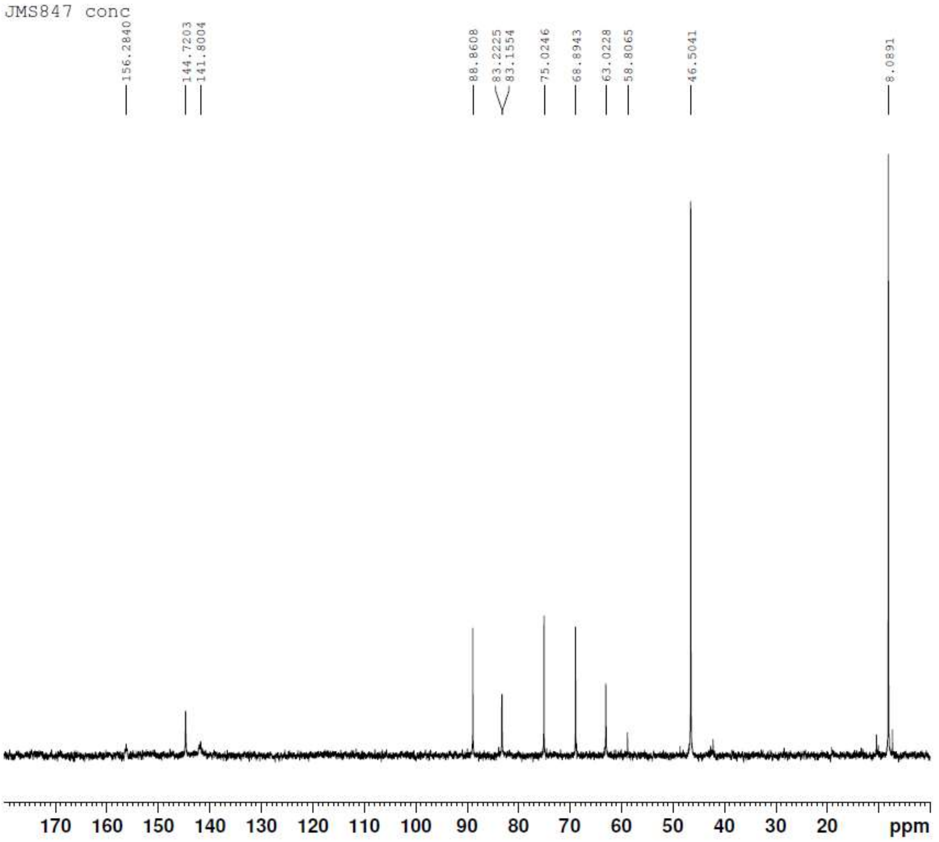


```
Current Data Parameters
NAME           JMS847 conc
EXPNO                   11
PROCNO                   1

F2 - Acquisition Parameters
Date_             20130725
Time                 17.00
INSTRUM              spect
PROBHD   5 mm PABBO BB-
PULPROG            zgpg30
TD                   65536
SOLVENT                D2O
NS                   16384
DS                       4
SWH              29761.904 Hz
FIDRES            0.454131 Hz
AQ               1.1010048 sec
RG                    2050
DW                  16.800 usec
DE                    8.45 usec
TE                   293.5 K
D1              2.00000000 sec
D11             0.03000000 sec
TD0                      1

======== CHANNEL f1 ========
NUC1                   13C
P1                    9.40 usec
PL1                  -0.51 dB
PL1W           99.92730713 W
SFO1           125.7703643 MHz

======== CHANNEL f2 ========
CPDPRG[2           waltz16
NUC2                    1H
PCPD2                80.00 usec
PL2                  -0.12 dB
PL12                 17.94 dB
PL13                 21.00 dB
PL2W           19.35150909 W
PL12W           0.30249262 W
PL13W           0.14952536 W
SFO2           500.1320005 MHz

F2 - Processing parameters
SI                   32768
SF             125.7577890 MHz
WDW                     EM
SSB                      0
LB                    3.00 Hz
GB                       0
PC                    1.40
```

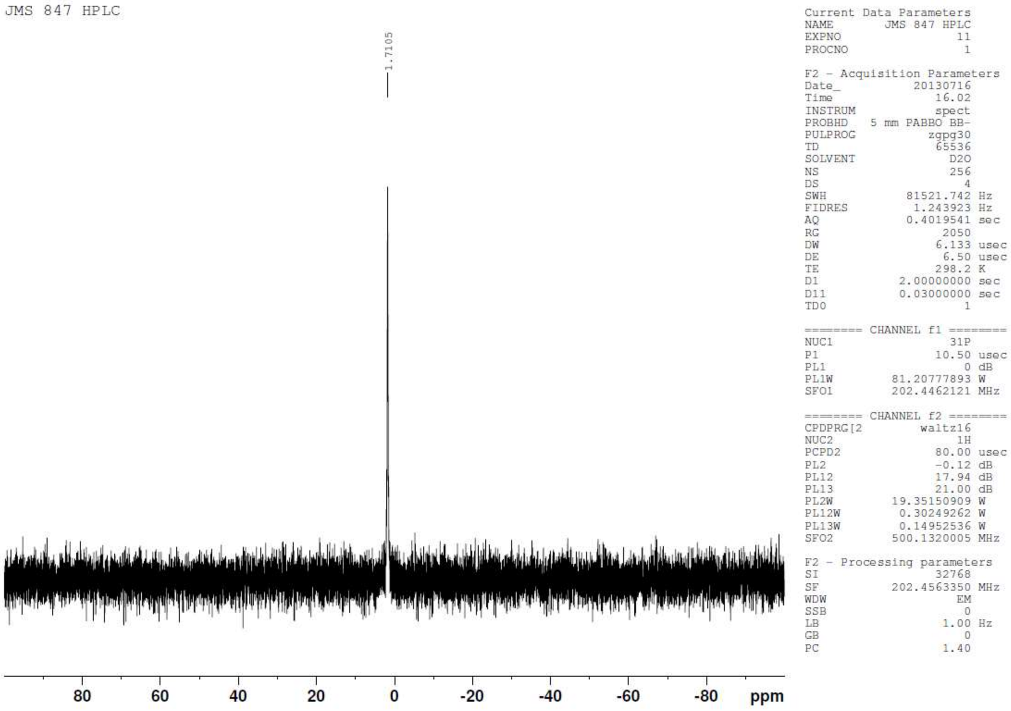

SAMPLE INFORMATION

Sample Name:	JMS 847 uv	Acquired By:	Joanna
Sample Type:	Unknown	Date Acquired:	24/07/2013 3:10:17 PM
Vial:	2	Acq. Method Set:	isocratic new PDA Jo
Injection #:	1	Date Processed:	27/09/2013 3:48:56 PM
Injection Volume:	10.00 ul	Processing Method:	L N1 IMP
Run Time:	15.0 Minutes	Channel Name:	Wvln Ch1
Sample Set Name:	JMS 847 and 845 uv	Proc. Chnl. Descr.:	PDA 254.0 nm

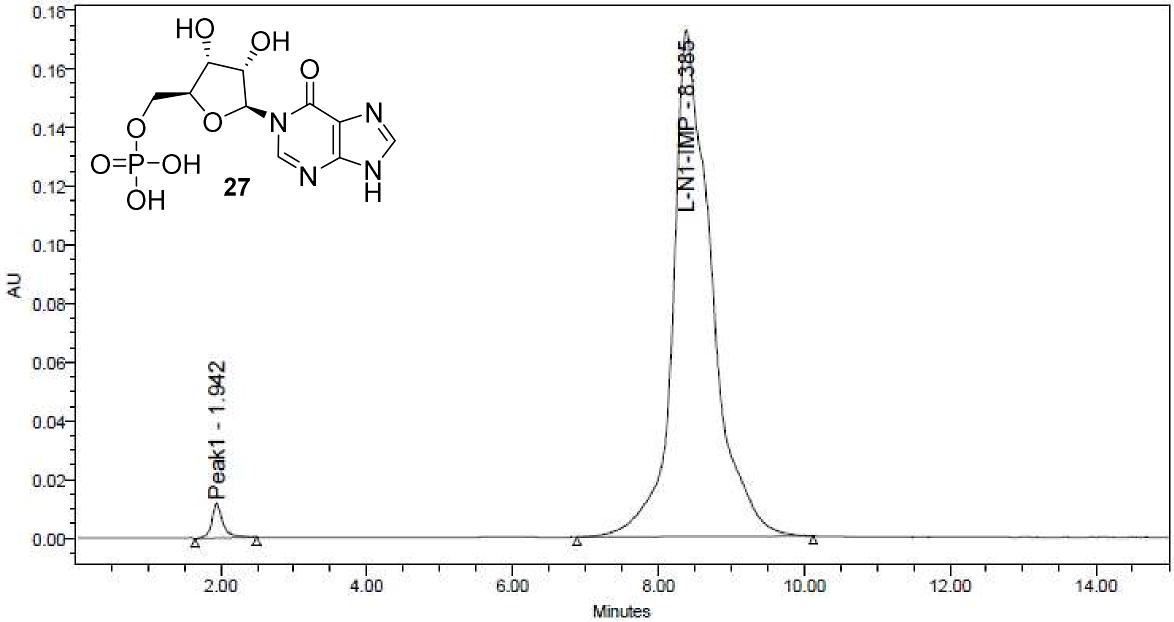

	Peak Name	RT	Area	% Area	Height
1	Peak1	1.942	129676	2.00	11947
2	L-N1-IMP	8.385	6365515	98.00	172494

Total synthesis of pyrophosphate bioisostere analogues (28-29)

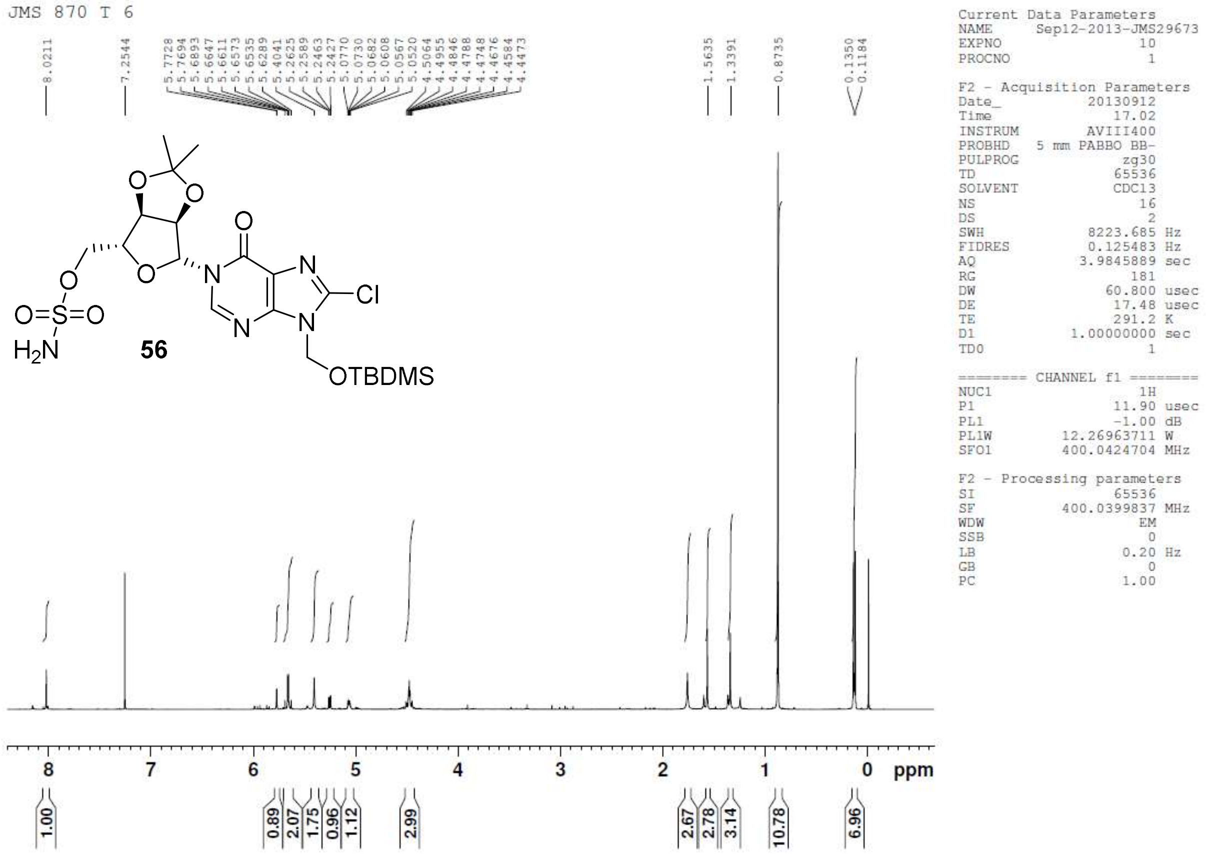

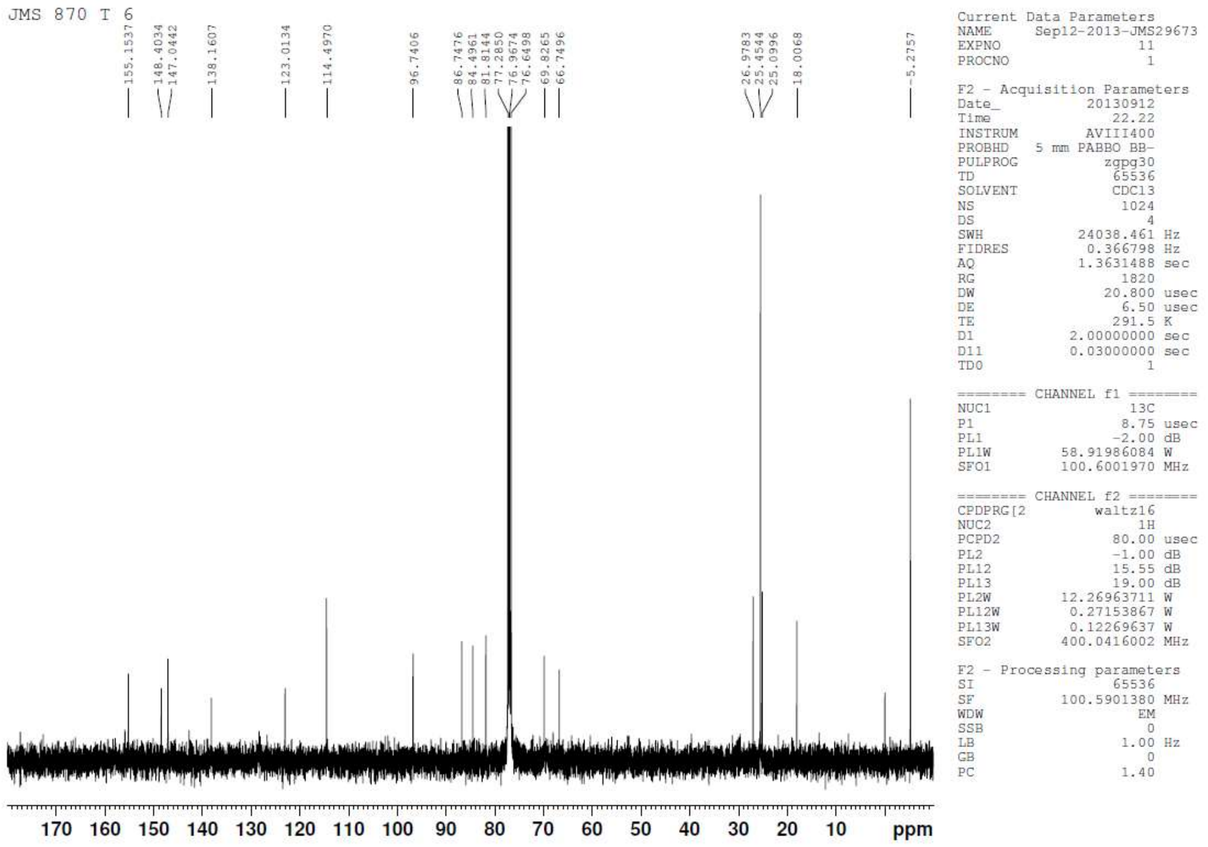

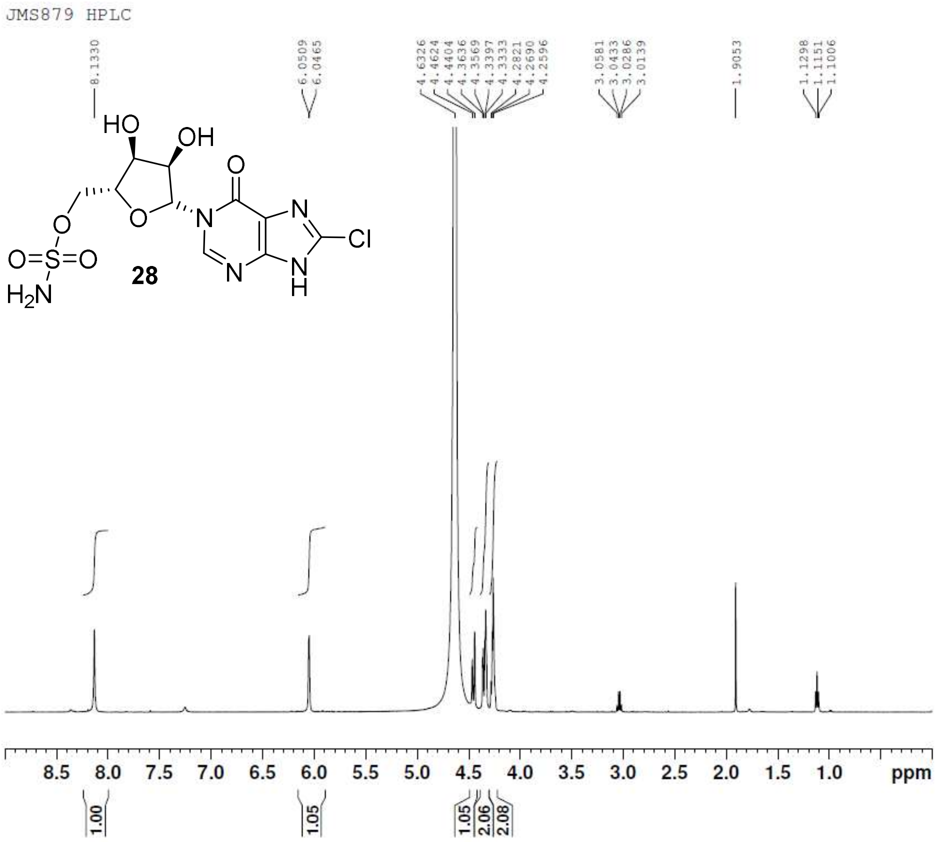

Current Data Parameters
NAME JMS879 HPLC
EXPNO 10
PROCNO 1

F2 - Acquisition Parameters
Date_ 20131129
Time 5.53
INSTRUM spect
PROBHD 5 mm PABBO BB-
PULPROG zg30
TD 65536
SOLVENT D2O
NS 64
DS 2
SWH 10330.578 Hz
FIDRES 0.157632 Hz
AQ 3.1719425 sec
RG 228
DW 48.400 usec
DE 14.00 usec
TE 298.2 K
D1 1.00000000 sec
TD0 1

======== CHANNEL f1 ========
NUC1 1H
P1 10.00 usec
PL1 -0.12 dB
PL1W 19.35150909 W
SFO1 500.1330885 MHz

F2 - Processing parameters
SI 32768
SF 500.1300350 MHz
WDW EM
SSB 0
LB 0.30 Hz
GB 0
PC 1.00

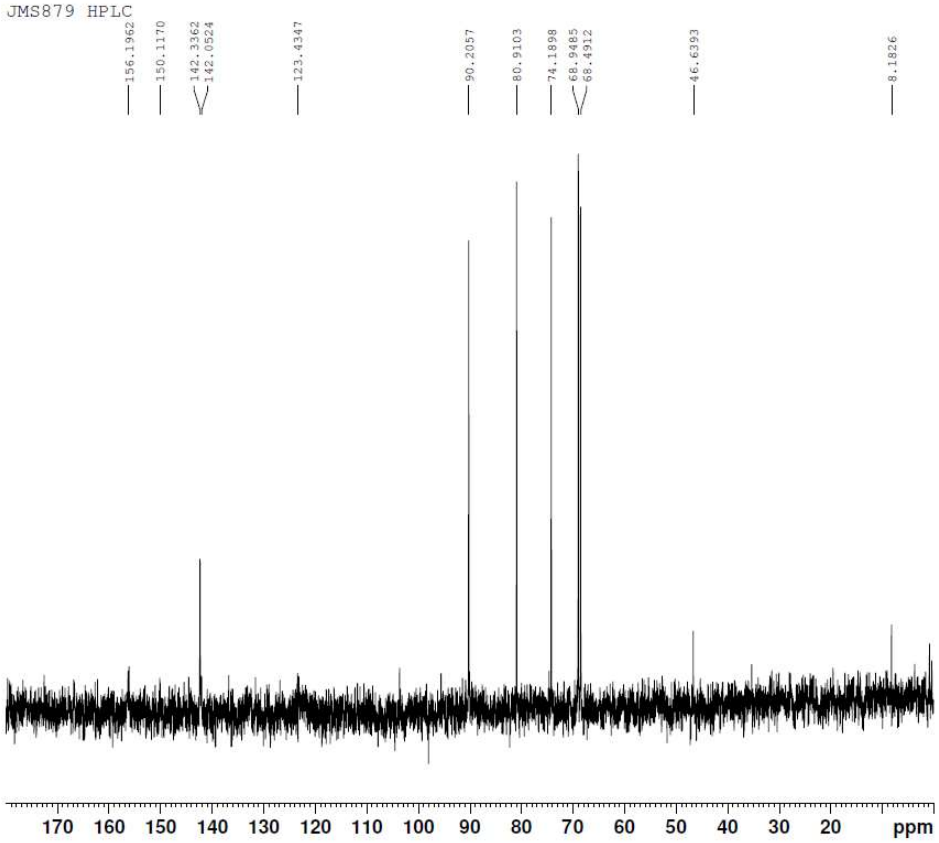

Current Data Parameters
NAME JMS879 HPLC
EXPNO 17
PROCNO 1

F2 - Acquisition Parameters
Date_ 20131201
Time 23.06
INSTRUM spect
PROBHD 5 mm PABBO BB-
PULPROG zgpg30
TD 65536
SOLVENT D2O
NS 10240
DS 4
SWH 29761.904 Hz
FIDRES 0.454131 Hz
AQ 1.1010048 sec
RG 2050
DW 16.800 usec
DE 8.45 usec
TE 298.2 K
D1 2.00000000 sec
D11 0.03000000 sec
TD0 1

======== CHANNEL f1 ========
NUC1 13C
P1 9.40 usec
PL1 -0.51 dB
PL1W 99.92730713 W
SFO1 125.7703643 MHz

======== CHANNEL f2 ========
CPDPRG[2 waltz16
NUC2 1H
PCPD2 80.00 usec
PL2 -0.12 dB
PL12 17.94 dB
PL13 21.00 dB
PL2W 19.35150909 W
PL12W 0.30249262 W
PL13W 0.14952536 W
SFO2 500.1320005 MHz

F2 - Processing parameters
SI 32768
SF 125.7577890 MHz
WDW EM
SSB 0
LB 3.00 Hz
GB 0
PC 1.40

SAMPLE INFORMATION

Sample Name:	JMS 879 B9	Acquired By:	Joanna
Sample Type:	Unknown	Date Acquired:	27/11/2013 3:35:18 AM
Vial:	94	Acq. Method Set:	RP18 LC
Injection #:	1	Date Processed:	18/12/2013 11:48:24 AM
Injection Volume:	10.00 ul	Processing Method:	8CI_N1_IMS in MeCN_H20
Run Time:	20.0 Minutes	Channel Name:	WvlnCh1
Sample Set Name:	JMS 879 HPLC B	Proc. Chnl. Descr.:	PDA 262.1 nm

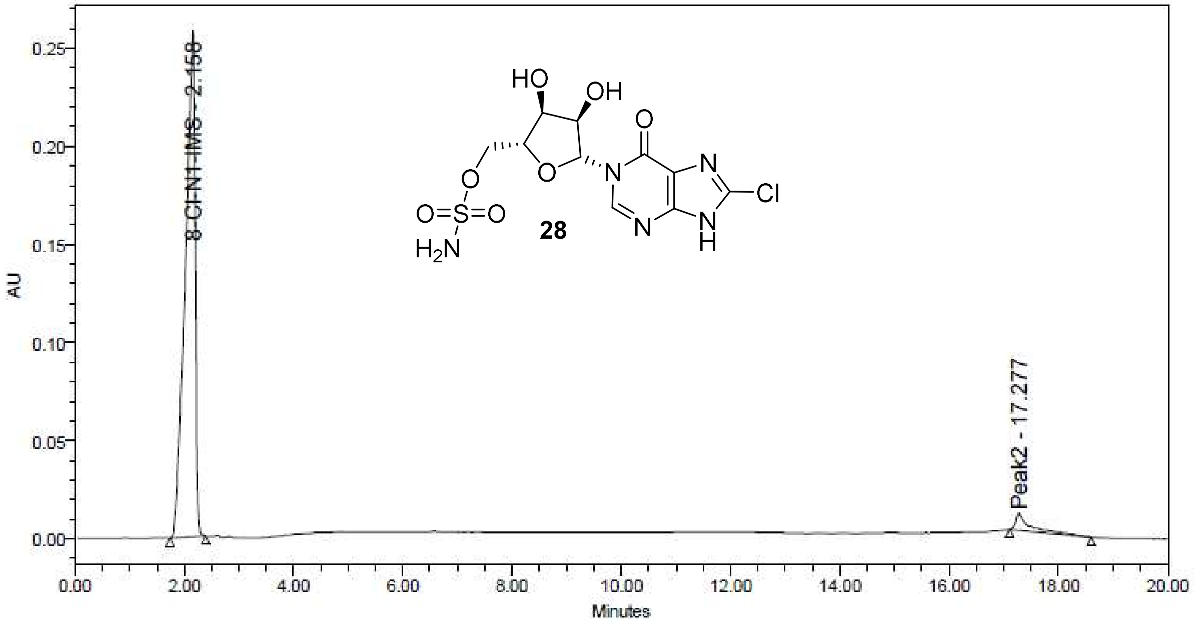

	Peak Name	RT	Area	% Area	Height
1	8-Cl-N1-IMS	2.158	3256505	95.31	257699
2	Peak2	17.277	160233	4.69	8449

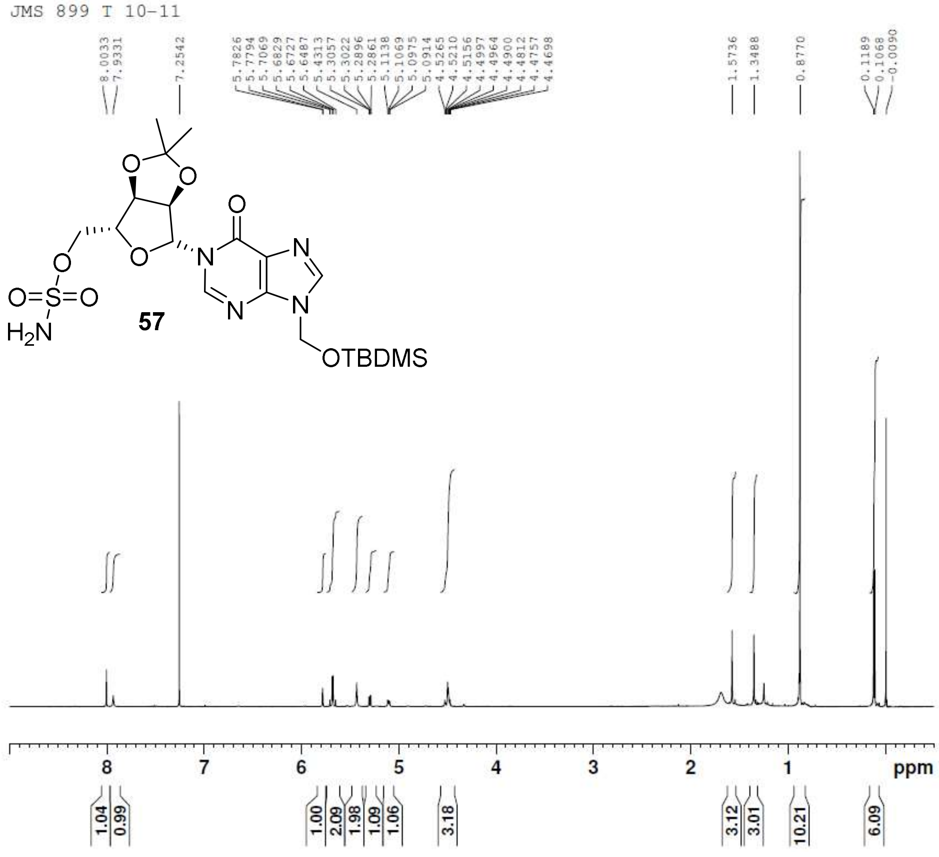

Current Data Parameters
NAME Oct21-2013-JMS30022
EXPNO 10
PROCNO 1

F2 - Acquisition Parameters
Date_ 20131021
Time 15.50
INSTRUM AVIII400
PROBHD 5 mm PABBO BB-
PULPROG zg30
TD 65536
SOLVENT CDCl3
NS 16
DS 2
SWH 8223.685 Hz
FIDRES 0.125483 Hz
AQ 3.9845889 sec
RG 228
DW 60.800 usec
DE 17.48 usec
TE 293.2 K
D1 1.00000000 sec
TD0 1

======== CHANNEL f1 ========
NUC1 1H
P1 11.90 usec
PL1 -1.00 dB
PL1W 12.26963711 W
SFO1 400.0424704 MHz

F2 - Processing parameters
SI 65536
SF 400.0399837 MHz
WDW EM
SSB 0
LB 0.20 Hz
GB 0
PC 1.00

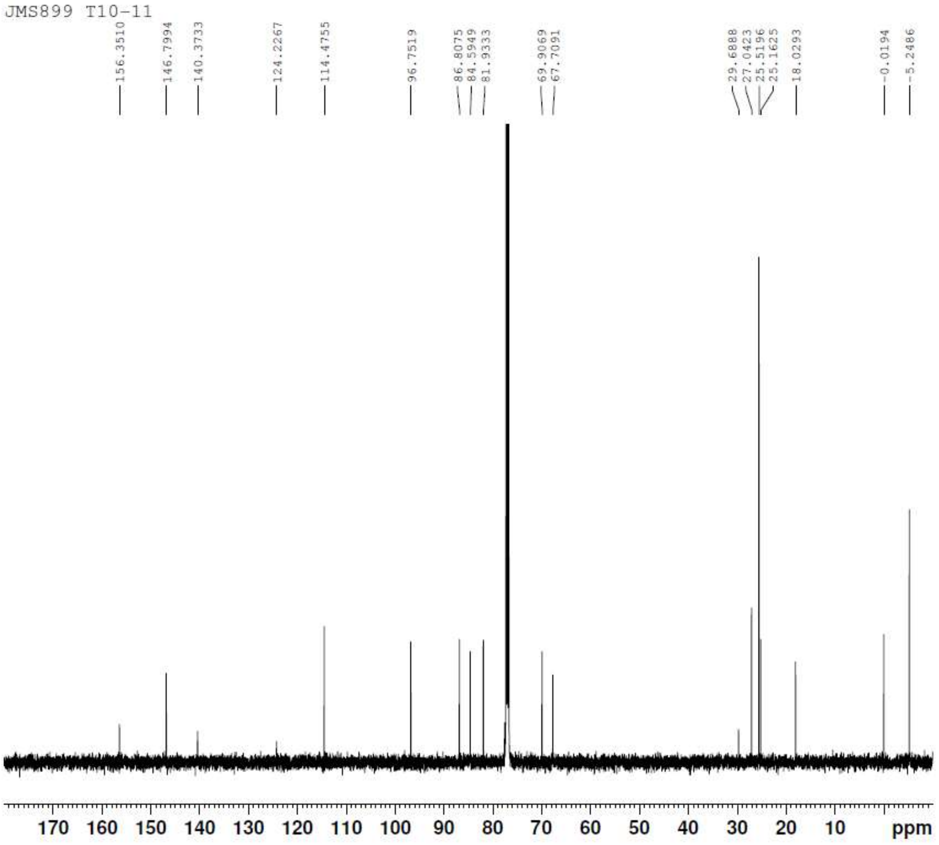

Current Data Parameters
NAME JMS899 T10-11
EXPNO 16
PROCNO 1

F2 - Acquisition Parameters
Date_ 20131022
Time 14.48
INSTRUM spect
PROBHD 5 mm PABBO BB-
PULPROG zgpg30
TD 65536
SOLVENT CDCl3
NS 4096
DS 4
SWH 29761.904 Hz
FIDRES 0.454131 Hz
AQ 1.1010048 sec
RG 2050
DW 16.800 usec
DE 8.45 usec
TE 298.2 K
D1 2.00000000 sec
D11 0.03000000 sec
TD0 1

======== CHANNEL f1 ========
NUC1 13C
P1 9.40 usec
PL1 -0.51 dB
PL1W 99.92730713 W
SFO1 125.7703643 MHz

======== CHANNEL f2 ========
CPDPRG[2 waltz16
NUC2 1H
PCPD2 80.00 usec
PL2 -0.12 dB
PL12 17.94 dB
PL13 21.00 dB
PL2W 19.35150909 W
PL12W 0.30249262 W
PL13W 0.14952536 W
SFO2 500.1320005 MHz

F2 - Processing parameters
SI 32768
SF 125.7577914 MHz
WDW EM
SSB 0
LB 1.00 Hz
GB 0
PC 1.40

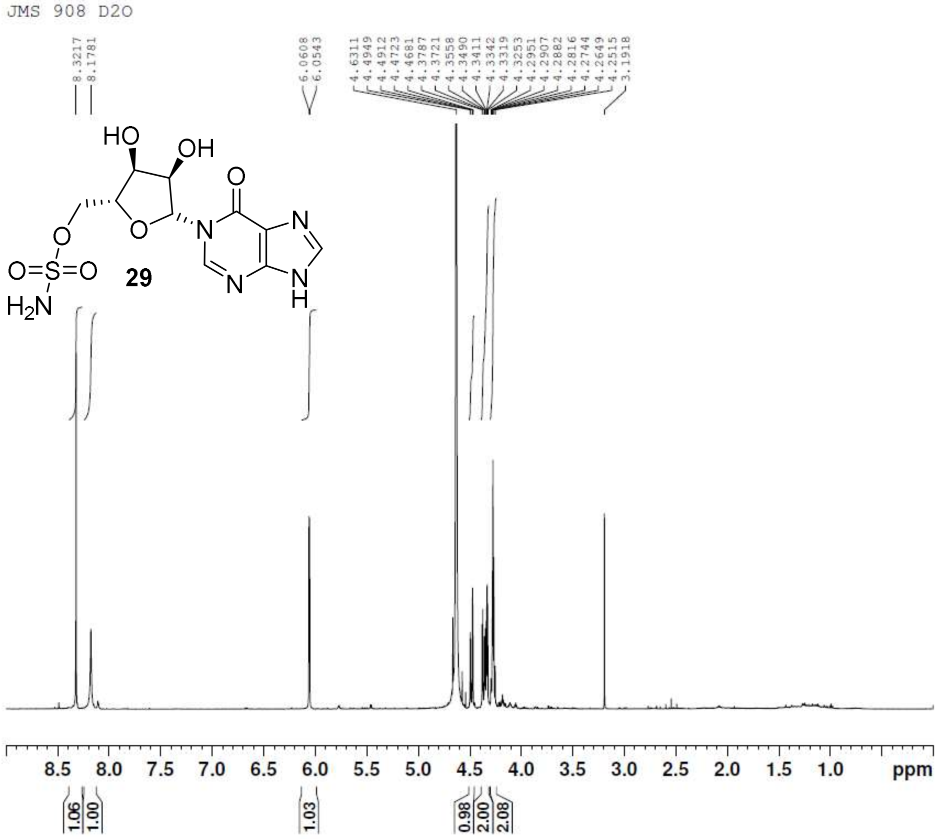

Current Data Parameters
NAME JMS 908-2
EXPNO 11
PROCNO 1

F2 - Acquisition Parameters
Date_ 20131101
Time 16.42
INSTRUM spect
PROBHD 5 mm PABBO BB-
PULPROG zg30
TD 65536
SOLVENT D2O
NS 64
DS 2
SWH 10330.578 Hz
FIDRES 0.157632 Hz
AQ 3.1719425 sec
RG 228
DW 48.400 usec
DE 14.00 usec
TE 298.2 K
D1 1.00000000 sec
TD0 1

======== CHANNEL f1 ========
NUC1 1H
P1 10.00 usec
PL1 -0.12 dB
PL1W 19.35150909 W
SFO1 500.1330885 MHz

F2 - Processing parameters
SI 32768
SF 500.1300364 MHz
WDW EM
SSB 0
LB 0.30 Hz
GB 0
PC 1.00

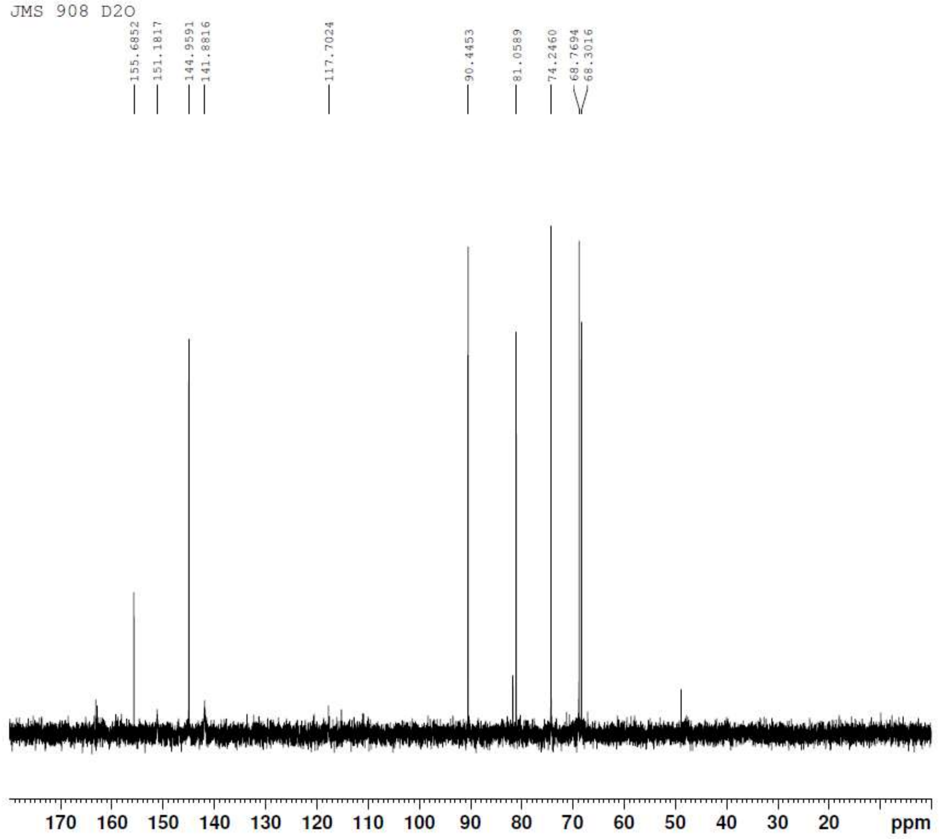

Current Data Parameters
NAME JMS 908-2
EXPNO 17
PROCNO 1

F2 - Acquisition Parameters
Date_ 20131102
Time 22.14
INSTRUM spect
PROBHD 5 mm PABBO BB-
PULPROG zgpg30
TD 65536
SOLVENT D2O
NS 20480
DS 4
SWH 29761.904 Hz
FIDRES 0.454131 Hz
AQ 1.1010048 sec
RG 2050
DW 16.800 usec
DE 8.45 usec
TE 298.2 K
D1 2.00000000 sec
D11 0.03000000 sec
TD0 1

======== CHANNEL f1 ========
NUC1 13C
P1 9.40 usec
PL1 -0.51 dB
PL1W 99.92730713 W
SFO1 125.7703643 MHz

======== CHANNEL f2 ========
CPDPRG[2 waltz16
NUC2 1H
PCPD2 80.00 usec
PL2 -0.12 dB
PL12 17.94 dB
PL13 21.00 dB
PL2W 19.35150909 W
PL12W 0.30249262 W
PL13W 0.14952536 W
SFO2 500.1320005 MHz

F2 - Processing parameters
SI 32768
SF 125.7577890 MHz
WDW EM
SSB 0
LB 1.00 Hz
GB 0
PC 1.40

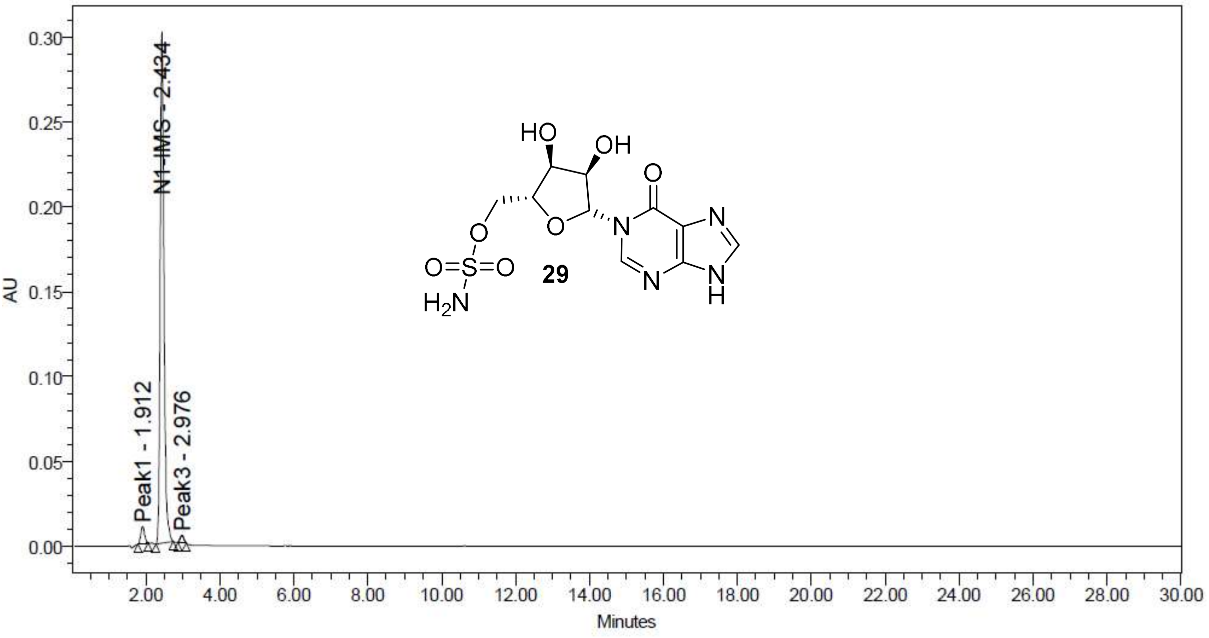

	Peak Name	RT	Area	% Area	Height
1	Peak1	1.912	73083	3.25	10284
2	N1-IMS	2.434	2148227	95.41	301144
3	Peak3	2.976	30303	1.35	4335
